# Effects of vitamin and mineral supplementation during pregnancy on maternal, birth, child health and development outcomes in low‐ and middle‐income countries: A systematic review

**DOI:** 10.1002/cl2.1127

**Published:** 2021-06-26

**Authors:** Emily C. Keats, Christina Oh, Tamara Chau, Dina S. Khalifa, Aamer Imdad, Zulfiqar A. Bhutta

**Affiliations:** ^1^ Centre for Global Child Health The Hospital for Sick Children Toronto Canada; ^2^ Pediatrics Upstate Medical University, Syracuse New York USA

## Abstract

**Background:**

Almost two billion people who are deficient in vitamins and minerals are women and children in low‐ and middle‐income countries (LMIC). These deficiencies are worsened during pregnancy due to increased energy and nutritional demands, causing adverse outcomes in mother and child. To reduce micronutrient deficiencies, several strategies have been implemented, including diet diversification, large‐scale and targeted fortification, staple crop bio‐fortification and micronutrient supplementation.

**Objectives:**

To evaluate and summarize the available evidence on the effects of micronutrient supplementation during pregnancy in LMIC on maternal, fetal, child health and child development outcomes. This review will assess the impact of single micronutrient supplementation (calcium, vitamin A, iron, vitamin D, iodine, zinc, vitamin B12), iron‐folic acid (IFA) supplementation, multiple micronutrient (MMN) supplementation, and lipid‐based nutrient supplementation (LNS) during pregnancy.

**Search Methods:**

We searched papers published from 1995 to 31 October 2019 (related programmes and good quality studies pre‐1995 were limited) in CAB Abstracts, CINAHL, Cochrane Central Register of Controlled Trials, Embase, International Initiative for Impact Evaluations, LILACS, Medline, POPLINE, Web of Science, WHOLIS, ProQuest Dissertations & Theses Global, R4D, WHO International Clinical Trials Registry Platform. Non‐indexed grey literature searches were conducted using Google, Google Scholar, and web pages of key international nutrition agencies.

**Selection Criteria:**

We included randomized controlled trials (individual and cluster‐randomized) and quasi‐experimental studies that evaluated micronutrient supplementation in healthy, pregnant women of any age and parity living in a LMIC. LMIC were defined by the World Bank Group at the time of the search for this review. While the aim was to include healthy pregnant women, it is likely that these populations had one or more micronutrient deficiencies at baseline; women were not excluded on this basis.

**Data Collection and Analysis:**

Two authors independently assessed studies for inclusion and risk of bias, and conducted data extraction. Data were matched to check for accuracy. Quality of evidence was assessed using the GRADE approach.

**Main Results:**

A total of 314 papers across 72 studies (451,723 women) were eligible for inclusion, of which 64 studies (439,649 women) contributed to meta‐analyses. Seven studies assessed iron‐folic acid (IFA) supplementation versus folic acid; 34 studies assessed MMN vs. IFA; 4 studies assessed LNS vs. MMN; 13 evaluated iron; 13 assessed zinc; 9 evaluated vitamin A; 11 assessed vitamin D; and 6 assessed calcium. Several studies were eligible for inclusion in multiple types of supplementation. IFA compared to folic acid showed a large and significant (48%) reduction in the risk of maternal anaemia (average risk ratio (RR) 0.52, 95% CI 0.41 to 0.66; studies = 5; participants = 15,540; moderate‐quality evidence). As well, IFA supplementation demonstrated a smaller but significant, 12% reduction in risk of low birthweight (LBW) babies (average RR 0.88, 95% CI 0.78 to 0.99; studies = 4; participants = 17,257; high‐quality evidence). MMN supplementation was defined as any supplement that contained at least 3 micronutrients. Post‐hoc analyses were conducted, where possible, comparing the differences in effect of MMN with 4+ components and MMN with 3 or 4 components. When compared to iron with or without FA, MMN supplementation reduced the risk of LBW by 15% (average RR 0.85, 95% CI 0.77 to 0.93; studies = 28; participants = 79,972); this effect was greater in MMN with >4 micronutrients (average RR 0.79, 95% CI 0.71 to 0.88; studies = 19; participants = 68,138 versus average RR 1.01, 95% CI 0.92 to 1.11; studies = 9; participants = 11,834). There was a small and significant reduction in the risk of stillbirths (average RR 0.91; 95% CI 0.86 to 0.98; studies = 22; participants = 96,772) and a small and significant effect on the risk of small‐for‐gestational age (SGA) (average RR 0.93; 95% CI 0.88 to 0.98; studies = 19; participants = 52,965). For stillbirths and SGA, the effects were greater among those provided MMN with 4+ micronutrients. Children whose mothers had been supplemented with MMN, compared to IFA, demonstrated a 16% reduced risk of diarrhea (average RR 0.84; 95% CI 0.76 to 0.92; studies = 4; participants = 3,142). LNS supplementation, compared to MMN, made no difference to any outcome; however, the evidence is limited. Iron supplementation, when compared to no iron or placebo, showed a large and significant effect on maternal anaemia, a reduction of 47% (average RR 0.53, 95% CI 0.43 to 0.65; studies = 6; participants = 15,737; moderate‐quality evidence) and a small and significant effect on LBW (average RR 0.88, 95% CI 0.78 to 0.99; studies = 4; participants = 17,257; high‐quality evidence). Zinc and vitamin A supplementation, each both compared to placebo, had no impact on any outcome examined with the exception of potentially improving serum/plasma zinc (mean difference (MD) 0.43 umol/L; 95% CI −0.04 to 0.89; studies = 5; participants = 1,202) and serum/plasma retinol (MD 0.13 umol/L; 95% CI −0.03 to 0.30; studies = 6; participants = 1,654), respectively. When compared to placebo, vitamin D supplementation may have reduced the risk of preterm births (average RR 0.64; 95% CI 0.40 to 1.04; studies = 7; participants = 1,262), though the upper CI just crosses the line of no effect. Similarly, calcium supplementation versus placebo may have improved rates of pre‐eclampsia/eclampsia (average RR 0.45; 95% CI 0.19 to 1.06; studies = 4; participants = 9,616), though the upper CI just crosses 1.

**Authors' Conclusions:**

The findings suggest that MMN and vitamin supplementation improve maternal and child health outcomes, including maternal anaemia, LBW, preterm birth, SGA, stillbirths, micronutrient deficiencies, and morbidities, including pre‐eclampsia/eclampsia and diarrhea among children. MMN supplementation demonstrated a beneficial impact on the most number of outcomes. In addition, MMN with >4 micronutrients appeared to be more impactful than MMN with only 3 or 4 micronutrients included in the tablet. Very few studies conducted longitudinal analysis on longer‐term health outcomes for the child, such as anthropometric measures and developmental outcomes; this may be an important area for future research. This review may provide some basis to guide continual discourse around replacing IFA supplementation with MMN along with the use of single micronutrient supplementation programs for specific outcomes.

## 1 PLAIN LANGUAGE SUMMARY

### 1.1 Micronutrient and vitamin supplementation during pregnancy improves some maternal and child health outcomes

Micronutrients and vitamins are important for the body's normal functioning, growth and development, but many women and children get too few from their diets.

### 1.2 What is this review about?

Micronutrient deficiencies, the lack of essential vitamins and minerals, are especially a concern during pregnancy, when energy and nutrient demands are greater for both mother and foetus, and can lead to adverse health and development outcomes for the mother and child, such as being born too soon or too small.

A cost‐effective strategy that has been implemented worldwide is to provide micronutrient and vitamin supplementation during pregnancy to achieve better health outcomes in the mother and child. There are different types of supplementation, including provision of a single micronutrient/vitamin (e.g. vitamin D), two micronutrients/vitamins (e.g. iron‐folic acid supplementation), or several micronutrients/vitamins, which can be in the form of tablets, powders, or fat‐based products.

Multiple micronutrient supplementation will help women and children who have more than one type of deficiency. However, it is understood that some micronutrients and vitamins can compete for absorption in the body and, as such, intake of several micronutrients at the same time may not be as effective as one would hope.

It is important to evaluate the various types of supplementation in pregnancy and their effects on maternal and child health outcomes to determine the best strategy for reducing poor outcomes.

### 1.3 What is the aim of this review?

This Campbell systematic review evaluates the various types of supplementation in pregnancy and their effects on maternal and child health outcomes to determine the best strategy for reducing poor outcomes.

### 1.4 What studies are included?

We searched for papers published between 1995 and 31 October 2019 in a variety of relevant databases and within grey literature. This systematic review included 314 papers across 72 studies (involving 451,723 women), of which 64 studies (involving 439,649 women) contributed to meta‐analysis.

The included studies used the following comparisons: iron‐folic acid supplementation versus folic‐acid only; multiple‐micronutrient supplementation versus iron‐folic acid or iron alone; lipid nutrient‐based supplementation versus multiple micronutrients; and iron, zinc, vitamin A, vitamin D or calcium supplementations, each compared to placebo.

### 1.5 What are the main findings of the review?

Across all comparisons, micronutrient and vitamin supplementation had little to no effect on the number of deaths among mothers and infants. Overall, women who received multiple micronutrient supplementation had fewer babies that were born early (<37 weeks of gestation), fewer babies born too small (<2500 g) and fewer babies who were smaller in size than normal for their gestational age, compared to women who received iron‐folic acid supplementation. Iron or iron‐folic acid supplementation was very good at improving maternal anaemia.

Mothers who received calcium supplementation may have had a decreased risk of pre‐eclampsia and eclampsia during pregnancy, and mothers who received vitamin D compared to mothers who received placebo may have had fewer babies that were born early (<37 weeks of gestation).

Women supplemented with vitamin A compared to mothers given placebo had higher amounts of serum/plasma retinol, while zinc supplementation had no effect on all maternal and child outcomes reported.

Lipid nutrient‐based supplementation showed little to no effect compared to multiple micronutrients; however, there were very few studies included and further research should be conducted.

### 1.6 What do the findings of this review mean?

The findings of this review support the use of micronutrient and vitamin supplementation during pregnancy to improve certain maternal and child health outcomes, which is especially important for populations living in low‐ and middle‐income settings. Supplementation with multiple micronutrients was particularly good at improving several outcomes.

Further research should be done to establish the effects of supplementation on pregnant adolescents, who may have specific needs. Also, more data is needed to understand supplementation effects on long‐term health and development outcomes for mother and child.

### 1.7 How up‐to‐date is this review?

The review authors searched for studies published up to October 2019.

## 2 BACKGROUND

### 2.1 Description of the condition

Close to two billion people today are deficient in key vitamins and minerals; of these individuals, the vast majority are women and children residing in low‐ and middle‐income countries (LMIC) (Black et al., 2013; Development Initiatives, 2018). Among women of reproductive age (WRA) in LMIC, micronutrient deficiencies result from diets that chronically lack diversity and thus do not provide sufficient amounts of essential vitamins and minerals to meet recommended daily allowances (FAO and WHO, 2004). In some cases, infections and/or chronic disease may contribute to micronutrient deficiencies by directly inhibiting nutrient absorption (Bailey et al., 2015).

Because of increased nutritional requirements throughout pregnancy, micronutrient deficiencies are often exacerbated during this time. Additionally, repeated pregnancies and short inter‐pregnancy intervals have been shown to contribute to poor maternal micronutrient status (Darnton‐Hill, & Mkparu, 2015). As such, multiple concurrent deficiencies (in two or more micronutrients) are common among pregnant women, especially in LMIC (Allen & Peerson, 2009; Black, 2003). Though population‐level estimates are mostly lacking, the global prevalence of prenatal iron deficiency anaemia is estimated to be 19.2% (95% confidence interval (CI) 17.1%‐21.5%), while vitamin A deficiency affects 15.3% (95% CI 6.0%‐24.6%) of pregnant women (Black et al., 2013). Restricting to LMIC could produce slightly larger prevalence estimates.

Micronutrient deficiencies are associated with a host of adverse outcomes for both the mother and the baby. Anemia in pregnancy, typically caused by iron deficiency, increases the risk of maternal mortality, perinatal mortality, and low birthweight (Allen 2001; Christian, 2010; Haider et al., 2013). Low folate levels are unequivocally associated with neural tube defects (NTD) (De‐Regil et al., 2015], and severe iodine deficiency affects fetal development, including increasing the risk of mental retardation and cretinism (Dunn, 1993). Low calcium intake during pregnancy is associated with the development of hypertension, and hypertension is one of the leading causes of maternal morbidity, mortality, fetal growth restriction and preterm birth (Bucher et al., 1996; Ortega et al., 1999; Hofmeyr, 2018). Similarly, low vitamin D levels throughout gestation can lead to pre‐eclampsia and, subsequently, increase the risk of preterm birth, small‐for‐gestational age (SGA), and perinatal mortality (De‐Regil et al., 2016; Dror, 2011; MacKay et al., 2001). The effects of maternal zinc deficiency are not well understood, but it has been suggested that zinc supplementation during pregnancy can result in the reduction of preterm birth (Ota et al., 2015). Maternal malnutrition has also been shown to manifest through intergenerational effects, impacting the short‐term and long‐term outcomes of offspring, including growth, neurodevelopment and cognition, and cardiometabolic, pulmonary, and immune function (Gernand et al., 2016). Poor maternal nutrition reduces a newborn's chance to achieve proper growth and development in the short‐term and, together, these early life inputs can establish the trajectory for chronic and other diseases later in life. Evidence has indicated that poor fetal and infant growth can lead to stunting in adulthood, chronic diseases relating to nutrition, lower educational attainment, reduced income, and even decreased birthweight in the next generation (Victora et al., 2008), highlighting the immense health and social consequences of maternal malnutrition.

### 2.2 Description of the intervention

Several strategies exist for reducing micronutrient malnutrition among women. These include diet diversification, large‐scale and targeted fortification, bio‐fortification of staple crops, and micronutrient supplementation with tablets or powders (Bhutta et al., 2008). This review will encompass micronutrient supplementation interventions during pregnancy.

Generally, micronutrient supplementation is used as a short term, preventive strategy that is targeted towards specific at‐risk population groups (Bailey et al., 2015). As such, supplementation has been recommended as part of routine antenatal care to overcome the complications associated with micronutrient deficiencies during pregnancy.

Within the context of routine antenatal care for pregnant women, the World Health Organization (WHO) currently recomends daily iron‐folic acid (IFA) supplementation with 30‐60 mg of elemental iron and 400 ug of folic acid (WHO, 2016). In populations where anaemia prevalence is less than 20% or where side effects from daily supplementation are severe, weekly IFA supplementation with 120 mg of elemental iron and 2800 ug folic acid is recommended instead (WHO, 2016). The WHO has issued several context‐specific recommendations as well: i) daily calcium supplementation (1.5‐2.9 grams oral elemental calcium) in populations with low dietary intake of calcium; ii) daily (up to 10,000 IU) or weekly (up to 25,000 IU) vitamin A supplementation where vitamin A deficiency is a severe public health problem (WHO, 2016). Currently, zinc supplementation is recommended only where there is rigorous research to support its provision, and vitamin D supplementation is not recommended for pregnant women to improve maternal and perinatal outcomes (WHO, 2016).

To address the issue of multiple deficiencies, the United Nations Children's Fund (UNICEF), United Nations University (UNU), and the WHO developed a multiple micronutrient (MMN) tablet that provides the daily recommended intake of vitamin A, vitamin B1, vitamin B2, niacin, vitamin B6, vitamin B12, folic acid, vitamin C, vitamin D, vitamin E, copper, selenium, and iodine with 30 mg iron and 15 mg of zinc for pregnant women (UNICEF, WHO, and UNU, 1999). Other such tablets have been developed for supplementation studies on a case‐by‐case basis, typically providing at least three essential micronutrients.

More recently, the use of lipid‐nutrient supplements (LNS) has been proposed to combat the adverse effects of maternal micronutrient deficiencies. Similar to MMN supplements, LNS contain a range of vitamins and minerals, but also provide energy, protein, and essential fatty acids. They are considered lipid‐based because energy from LNS comes in the form of fats, such as vegetable fat, peanut/groundnut paste, milk powder and sugar (Arimond et al., 2015). Lipid‐based products like Plumpy'nut were traditionally used for the treatment of severe acute malnutrition, but have since been adapted to contain a lower dose of energy such that daily supplementation with LNS products could be used as a preventive therapy for undernutrition (Arimond et al., 2015).

Supplementation with MMN is not recommended for pregnant women to improve maternal and perinatal outcomes, as more research is needed (WHO, 2016). The WHO has not yet issued any guidance for LNS (WHO, 2016).

### 2.3 How the intervention might work

Micronutrients, essential vitamins and minerals that are obtained from the diet, are critical for a host of metabolic activities that support tissue growth and functioning. As such, they are fundamental in enabling the healthy development of the fetus and promoting optimal pregnancy outcomes. Antenatal micronutrient supplementation interventions aim to increase circulating levels of vitamins and minerals in pregnant women in order to meet the recommended daily intakes, which are higher than normal due to increased physiological demands during pregnancy. Through tablets or other vehicles (e.g. syrup, drops, powder, or food matrices), the micronutrients are ingested and bioconverted to their active form in order to support maternal health and fetal development throughout gestation.

Through primary studies and meta‐analysis of randomised controlled studies (RCTs), some antenatal micronutrient supplementation interventions have proven to be efficacious in improving congenital/birth outcomes, including lowering the risk of NTD, cretinism, premature rupture of membranes (PROM), low birthweight, and preterm birth (Bougma et al., 2013; De‐Regil et al., 2015; De‐Regil et al., 2016; Haider et al., 2013; Lassi et al., 2013; Ota et al., 2015; Rumbold et al., 2015; Zhou et al., 2013). The duration of exposure needed to produce clinically meaningful results may vary depending on the supplement. For example, it is recommended that folic acid supplementation begin as early as possible, and ideally prior to conception (WHO, 2016), while daily iron supplementation that begins mid‐gestation has been effective at improving some outcomes (Peña‐Rosas et al., 2015).

### 2.4 Why it is important to do this review

There are several existing systematic reviews that examine the impact of single and multiple micronutrient supplementation interventions in pregnancy (Appendix 1), many of which incorporate data from studies conducted in low‐ and middle‐income settings. However, significant heterogeneity in results has been reported (e.g., for antenatal iron supplementation); this has not yet been explained by subgroup analysis. In addition, inconclusive results for several micronutrient supplementation interventions (e.g., folic acid supplementation for maternal health and pregnancy outcomes, calcium supplementation (other than for preventing or treating hypertensive disorders) for pregnancy and infant outcomes, and zinc supplementation for improving pregnancy and birth outcomes) were found, warranting further investigation. Many of the systematic reviews listed (Appendix 1) are several years old, underscoring the need to update the evidence in order to capture newly completed study data. There is the hope that with more power to detect differences, some unanswered questions will be resolved. For example, previous evidence suggests that male infants are at a greater risk for morbidity and mortality relative to their female counterparts, especially in the perinatal, neonatal and early infancy stages (Stevenson et al., 2000; Waldron, 1998; Zhao et al., 2017). As well, sex‐specific differences in infant mortality following MMN supplementation were noted in a study by Smith and colleagues (Smith et al., 2017). Thus, additional exploration is required to confirm the sex‐specific differences in morbidity and mortality amongst infants. Additionally, concerns have been raised regarding the safety of iron supplementation in women with high haemoglobin concentrations, and the potentially negative long‐term consequences that unabsorbed iron may have on child morbidity (Mwangi et al., 2017; Paganini et al., 2016).

In addition to the limitations of existing systematic reviews of RCTs, the effectiveness of antenatal micronutrient supplementation interventions in a real world setting has not been well established. We aim to understand which antenatal supplementation interventions are effective to improve key maternal and child health, nutrition, and mortality outcomes in LMIC. We will include data from large programme evaluations as well as smaller studies. Additionally, we will include adolescent women as a pre‐specified subgroup, which will help to elucidate strategies that can mitigate the risks associated with adolescent pregnancy in LMIC (Bhutta et al., 2017). Lastly, we hope to answer some of the remaining questions outlined above, including potential infant sex‐specific differences and safety concerns following supplementation in pregnancy. Taken together, these results will inform the evidence on which to base policy and programming relating to micronutrient supplementation in pregnancy for women in LMIC. In addition, this review will point to any gaps in the existing evidence.

## 3 OBJECTIVES

This review will summarize the available evidence on antenatal micronutrient supplementation interventions in LMIC. For each intervention, results will be summarized separately.

Specific objectives:

1. What is the impact of single micronutrient supplementation (calcium, vitamin A, vitamin D, iodine, zinc, vitamin B12) during pregnancy on maternal, birth, child health and development outcomes at longest follow‐up?
2. What is the impact of iron folic acid supplementation during pregnancy on maternal, birth, child health and development outcomes?
3. What is the impact of multiple micronutrient supplementation during pregnancy on maternal, birth, child health and development outcomes?
4. What is the impact of lipid‐based nutrient supplementation during pregnancy on maternal, birth, child health and development outcomes?

## 4 METHODS

### 4.1 Criteria for considering studies for this review

#### 4.1.1 Types of studies

We included the following study designs:

- Randomized controlled studies (RCTs), where participants were randomly assigned, individually or in clusters, to intervention and comparison groups. Cross‐over designs were eligible for inclusion.
- Quasi‐experimental designs, which include:
  – Natural experiments: studies where non‐random assignment is determined by factors that are out of the control of the investigator. One common type includes allocation based on exogenous geographical variation.
  – Controlled before‐after studies (CBA), in which measures were taken of an experimental group and a comparable control group both before and after the intervention. We also required that appropriate methods were used to control for confounding, such as statistical matching (e.g., propensity score matching, or covariate matching) or regression adjustment (e.g., difference‐in‐differences, instrumental variables).
  – Regression discontinuity designs; here, allocation to intervention/control is based upon a cut‐off score.
  – Interrupted time series (ITS) studies, in which outcomes were measured in the intervention group at least three time points before the intervention and after the intervention.

Reviews were excluded.

#### 4.1.2 Types of participants

Participants were healthy (i.e. non‐diseased) pregnant women of any age and parity living in LMIC. LMIC will be defined by the World Bank Group at the time of the search for this review. Though our aim was to include healthy pregnant women, the prevalence of micronutrient deficiencies is high in these settings, indicating that women are likely to have one or more micronutrient deficiencies at baseline; women were not excluded on this basis. Studies that include only a subset of eligible participants were retained as eligible, but were only included in analysis where data had been disaggregated appropriately for use.

#### 4.1.3 Types of interventions

The following interventions targeting pregnant women were included, and were analysed separately:

- Single micronutrient supplementation (calcium, vitamin D, iodine, folic acid, iron, vitamin A, zinc, vitamin B12) compared to placebo (Supplementation may take the form of tablets, drops, syrup or powder)
- Iron folic acid supplementation compared to folic acid alone or placebo
- Vitamin D and calcium supplementation compared to placebo
- MMN compared to iron folic acid supplementation or placebo: For MMN, studies that use fewer than 3 micronutrients in its composition were excluded (Haider & Bhutta, 2017; Kawai et al., 2011)
- LNS compared to MMN or placebo

For logistical reasons, we did not include every vitamin and mineral. Interventions were chosen based on relevance (i.e., most prevalent nutritional deficiencies) and data availability when considering the LMIC context.

There were no restrictions regarding: i) the duration of exposure to the intervention, ii) the provider of the intervention, iii) the frequency of the intervention (e.g. daily or intermittent supplementation), or iv) the food vehicle utilized for LNS interventions. We included studies where co‐interventions (e.g. education) were provided for both the intervention and the comparison groups.

#### 4.1.4 Types of outcome measures

To be included within this review, studies measured at least one of the following primary and/or secondary outcomes. We looked at maternal, fetal, neonatal and child health and nutrition outcomes that will help to inform related policy and practice. For simplifying, we grouped all secondary outcomes of interest by these domains. Unless otherwise specified, all outcomes listed were dichotomized (yes/no). We used mean and standard deviation (SD) to report all continuous outcomes (maternal biochemical status, newborn anthropometry, newborn/child biochemical status). Outcome definitions can be found in brackets below. International Units (IU) were used for all maternal outcomes whereas z‐scores were used for child outcomes, because z‐scores are adjusted for age.

#### 4.1.5 Primary outcomes

- Maternal Mortality (death while pregnant or within 42 days of pregnancy termination)
- Anemia/iron deficiency anaemia in third trimester of pregnancy [WHO, 2011]
  – Non‐anemic: > or equal to 110 g/L
  – Anemic: <110 g/L
- low birthweight (<2500 g)
- perinatal mortality (stillbirths and deaths <or equal to 7 days)

#### 4.1.6 Secondary outcomes

##### Maternal Outcomes

Morbidity from study enrolment up to 3 months post‐partum:

- Pre‐eclampsia/eclampsia
- Gestational hypertension
- Antepartum haemorrhage
- Postpartum haemorrhage
- Premature rupture of membranes
- Placental abruption
- Infections during pregnancy
- Bone mineral density
- Night blindness
- Need for blood transfusion

Biochemical status at endline:

- Micronutrient deficiencies
  – Vitamin A (serum/plasma retinol) (continuous)
  – Iron (serum/plasma ferritin, plasma TfR, TIBC) (continuous)
  – Serum/plasma/red blood cell folate (continuous)
  – Serum/plasma zinc (continuous)
  – Serum/plasma alkaline phosphatase (continuous)
  – Serum/plasma copper (continuous)
  – Serum/plasma vitamin D (25‐hydroxyvitamin D) (continuous)
  – Thyroglobulin concentration (continuous)

##### Fetal Outcomes

Mortality:

- Miscarriage (loss of pregnancy before 28 weeks gestation)
- Stillbirth (death at or beyond 28 weeks of gestation)

Morbidity:

- Congenital anomalies

##### Newborn Outcomes

Mortality:

- Neonatal mortality (deaths between 0 and 28 days)

Morbidity:

- Preterm birth (<37 weeks gestation)
- Small‐for‐gestational age (defined by study authors)
- Macrosomia (birthweight >4000 g)

Anthropometry measured from birth up to 14 days:

- Birth weight (z‐scores) (continuous)
- Birth length (z‐scores) (continuous)
- Head circumference (z‐scores) (continuous)

##### Child Outcomes

Mortality:

- Infant mortality (deaths between 0 and 12 months)
- Under‐five mortality (deaths between 0 and 59 months)

Morbidity:

- Stunting (‐2 z‐score or lower) at longest follow up
- Wasting (‐2 z‐score or lower) at longest follow up
- Underweight (‐2 z‐score or lower) at longest follow up
- Bone mineral density (continuous)
- Development outcomes (as defined by study authors)
- Infection

Biochemical status at endline:

- Micronutrient deficiencies
  – Vitamin A (serum/plasma retinol) (continuous)
  – Iron (serum/plasma ferritin, plasma TfR) (continuous)
  – Serum/plasma/red blood cell folate (continuous)
  – Serum/plasma zinc (continuous)
  – Serum/plasma vitamin D (25‐hydroxyvitamin D) (continuous)
- Anaemia
  – Hemoglobin concentration (continuous)
- Iron deficiency anaemia

##### Other Outcomes

- Relevant long‐term outcomes during adolescence or adulthood, as specified by study authors. For example:
  – Anthropometrics (stunting, wasting, underweight) in children >59 months
  – Cognitive and motor development as assessed by study investigators at longest follow up (e.g. Bayley Mental Development Index, Bayley Psychomotor Development Index, Stanford‐Binet test)
  – Educational attainment (completion of primary or secondary school)
- Mode of delivery (vaginal, instrumental vaginal, caesarean)

Adverse outcomes: any reported throughout intervention period (e.g. urinary tract infections, kidney stones, hyperthyroidism, allergic reactions, etc.), including short‐term adverse outcomes (e.g. vomiting, abdominal pain, constipation, diarrhoea, unpleasant tastes).

#### 4.1.7 Duration of follow‐up

There was no minimum duration of follow up.

#### 4.1.8 Types of settings

Other than the LMIC inclusion criteria, there were no restrictions regarding study setting.

Any *post hoc* changes to eligibility criteria or outcomes studied were aligned with the review objectives and were clearly stated with reasons justified.

### 4.2 Search methods for identification of studies

The search strategy was guided by our PICO model (Table 1), but was restricted by outcome in order to retain a broader search. The search was conducted using indexing terms, including medical subject headings (MeSH), keywords, and free text words. Details of the search strategy can be found in Appendix 2. To capture the most relevant evidence, we included articles published from 1995 to the end of June 2018 (related programmes and good quality studies before 1995 were very limited). There were no language or publication restrictions. Manual searches were conducted within reference lists of review articles and included studies, and experts were contacted to obtain any additional relevant maternal that may have been missed. The search process, including month/year of search, was documented to ensure that replication is possible.

**Table 1:** PICO table used for formulating our search strategy

P	Pregnant women of any age and parity, living in a low or middle‐income country
	Micronutrient supplementation interventions
I	Micronutrient supplementation interventions
	– Single and multiple micronutrient supplementation (including micronutrient powders) – Lipid‐nutrient supplementation
C	Author‐defined
O	Primary:
	– Anaemia/iron‐deficiency anaemia in pregnancy – Low birthweight – Perinatal mortality
	Seconday, maternal:
	– Mortality – Morbidity – Micronutrient deficiencies
	Secondary, fetal:
	– Mortality (miscarriage, stillbirth) – Congenital anomalies
	Secondary, newborn:
	– Mortality – Morbidity (preterm, small‐for‐gestational‐age, macrosomia) – Anthropometry (birth weight, birth length, head circumference)
	Secondary, child:
	– Mortality – Morbidity, including nutritional indicators (stunting, wasting, underweight) – Micronutrient deficiencies – Anaemia/iron‐deficiency anaemia
	Other secondary outcomes:
	– Author‐specified long‐term outcomes in adolescence or adulthood – Mode of delivery
	Adverse events

#### 4.2.1 Electronic searches

The search was run in the following databases, selected based on their applicability to the subject material:

- CAB Abstracts
- CINAHL
- Cochrane Central Register of Controlled Trials (CENTRAL)
- Embase
- International Initiative for Impact Evaluations (3ie)
- LILACS (Latin American and Caribbean health sciences literature)
- Medline
- POPLINE
- Web of Science
- WHOLIS (WHO library database)

#### 4.2.2 Searching other resources

##### Unpublished Studies

- ProQuest Dissertations & Theses Global
- R4D (Research for Development) material from UK government's Department for International Development
- WHO International Clinical Trials Registry Platform (ICTRP)

##### Grey Literature

Non‐indexed, grey literature searches was conducted to locate relevant programme evaluations and any additional studies. We searched Google, Google Scholar, and web pages of key international nutrition agencies (listed below) using key words based on PICO methodology. We used advanced search options, where possible. Google results were screened online until no relevant result appeared in 3 consecutive pages.

- Canadian Agency for Drugs and Technologies in Health (CADTH) tool for searching health‐related grey literature (http://www.cadth.ca/resources/finding-evidence/grey-matters)
- Centers for Disease Control and Prevention (CDC)
- Emergency Nutrition Network (ENN
- Global Alliance for Improved Nutrition (GAIN)
- Hellen Keller International
- International Food Policy and Research Institute (IFPRI)
- IZiNCG
- Nutrition International (NI)
- Sight and Life Foundation
- UNICEF
- World Food Programme (WFP)

### 4.3 Data collection and analysis

#### 4.3.1 Description of methods used in primary research

The vast majority of included studies were randomised or cluster‐randomised controlled studies that followed our inclusion/exclusion criteria, as listed above. For example, in a study published by Christian and colleagues (Christian et al., 2003), pregnant Nepalese women were cluster‐randomised to 1 of 5 groups: i) daily supplements of vitamin A (control), ii) daily supplements of vitamin A + folic acid, iii) daily supplements of vitamin A + folic acid + iron, iv) daily supplements of vitamin A + folic acid + iron + zinc, or v) daily multiple micronutrient supplements (including vitamin A) from early pregnancy to 72 hours postpartum. Outcomes assessed included infant anthropometrics (birth weight, length, and head circumference), and low birthweight (<2500 grams).

#### 4.3.2 Criteria for determination of independent findings

In order to take into account potential sources of dependency, we grouped studies in terms of their location, population, the programme that is being evaluated (if applicable), and intervention type to ensure that there is no double counting of evidence when synthesizing results across studies. Where there were multiple papers that described the same study, these papers were combined and coded as a single study.

For studies that include multiple intervention arms, we selected one pair (intervention and control) that satisfied the inclusion criteria of the review and excluded the rest. If >2 intervention groups met the eligibility criteria, then these groups were combined into a single pair‐wise comparison group and data was disaggregated into corresponding subgroups, or these arms were separated into different forest plots to ensure that there was no double counting of participants. Where multiple effect sizes from the same study and intervention‐control comparison were eligible, we chose to include the estimate with the larger sample size, though this occurred rarely.

#### 4.3.3 Selection of studies

Two independent reviewers performed title and abstract screening using specified inclusion/exclusion criteria. Where not enough information could be gleaned from the title alone, then abstracts were screened in order to determine eligibility for full text screening. All full texts were then screened in duplicate, with application of the same inclusion/exclusion criteria. A third reviewer resolved any disagreements. Both title/abstract and full text screening were done using Covidence, a web‐based software platform for systematic reviews. We assessed inter‐reviewer reliability/agreement by checking the number of conflicts in the Resolve Conflicts page following each stage of screening.

##### Examples of included studies

- Mridha MK, Matias SL, Chaparro CM, et al. (2016) Lipid‐based nutrient supplements for pregnant women reduce newborn stunting in a cluster‐randomised controlled effectiveness study in Bangladesh. Am J Clin Nutr; 103(1):236‐49.
- Roberfroid D, Huybregts L, Lamou H et al. (2008). Effects of maternal multiple micronutrient supplementation on fetal growth: a double‐blind, randomised controlled study in rural Burkina Faso. Am J Clin Nutr; 88:1330‐40.

##### Examples of excluded studies

- Harvey LJ, Dainty JR, Hollands WJ, et al. (2007) Effect of high‐dose iron supplements on fractional zinc absorption and status in pregnant women. Am J of Clin Nutr; 85:131‐6.
  – Ineligible population (high‐income setting)
- Boran P, Tokuc G, Vagas E, et al. (2006) Impact of zinc supplementation in children with acute diarrhoea in Turkey. Arch Dis Child; 91(4):296‐99.
  – Ineligible population (children 6 months to 5 years of age)

#### 4.3.4 Data extraction and management

For all included studies, we extracted data into a standardized data abstraction form that was comprised of a general study information sheet and a quantitative outcomes sheet. The data abstraction form was piloted before it was finalized. While all arms of a study were described in the tables of included studies, data was extracted and reported on only for those arms that met review criteria. All data abstraction was performed in duplicate. Coders were trained in systematic review methods, and data abstraction was cross‐checked with primary study data for accuracy by the team lead.

Each general study information sheet contained the following:

- General study information: authors, publication year, language of study, study design
- Study setting: World Bank region, country, World Bank income level, city/town, urban/urban slum/rural/mixed setting, duration of data collection, date of data collection
- Study population: sample size recruited, sample size analysed, male/female/mixed (%), age range of participants, mean/median age of participants, description of participants (i.e. inclusion/exclusion criteria applied to recruitment)
- Intervention characteristics: type of intervention, food vehicle utilized (where applicable), duration of intervention, level of delivery, unit of randomisation (where applicable), dose of micronutrient(s) provided, frequency of provision (i.e. daily, weekly, etc.), duration of follow up, attrition rate
- Programmatic indicators (based on the WHO/CDC logic model (De‐Regil et al., 2014)): policies, production, delivery strategies, quality control, behaviour change communication strategies, access and coverage, knowledge and appropriate use
- Funding source of programme (where applicable)
- Quality assessment (see section below: critical appraisal of studies

Each quantitative outcome sheet contained the following:

- Subgroup (if applicable)
- Subgroup sample size
- Outcome type (based on outcomes listed above)
- Outcome units
- Outcomes:
  – Outcome measure treatment group
  – Outcome measure comparison group
  – Standard deviation
- Effect size:
  – Effect measure (specify type); unadjusted and adjusted
  – 95% confidence interval (CI)
  – P‐value of effect measure
  – Standard error (SE) or standard deviation (SD) or t‐statistic

#### 4.3.5 Assessment of risk of bias in included studies

We critically appraised individual studies using the Cochrane Effective Practice and Organisation of Care (EPOC) guidelines for randomised studies, non‐randomised studies, controlled before‐after studies, and interrupted time series (ITS) studies. EPOC guidelines include the following standardized criteria for assessing bias of randomised, non‐randomised, and controlled before‐after studies [Cochrane Effective Practice and Organisation of Care (EPOC), 2017]:

- Random sequence generation
- Allocation concealment
- Baseline outcome measurements similar
- Baseline characteristics similar
- Incomplete outcome data
- Knowledge of the allocated interventions adequately prevented during study
- Protection against contamination
- Selective outcome reporting
- Other risks of bias (e.g. bias in measurement: validity and reliability of the measures used)

For ITS studies, the following criteria was considered [Cochrane Effective Practice and Organisation of Care (EPOC), 2017]:

- Intervention independent of other changes
- Shape of intervention effect pre‐specified
- Intervention unlikely to affect data collection
- Knowledge of the allocated interventions adequately prevented during study
- Incomplete outcome data
- Selective outcome reporting
- Other risks of bias (e.g. bias in measurement: validity and reliability of the measures used)

For EPOC rating schemes for randomised studies, non‐randomised studies, and controlled before‐after studies please see Table 2 and for interrupted time series studies, see Table 3.

**Table 2:** EPOC criteria for assessing risk of bias in randomised trials, non‐randomised trials, and controlled before‐after studies

Criteria	Rating Scheme
Random sequence generation	Low risk if a random component in the sequence generation process is described
	High risk when a non‐random method is used
	Unclear risk if not specified in the paper
Allocation concealment	Low risk if the unit of allocation was by institution, team or professional and allocation was performed on all units at the start of the study; or if the unit of allocation was by patient or episode of care and there was some form of centralised randomizations scheme, an on‐site computer system or sealed opaque envelopes were used
	Controlled before‐after studies are scored high risk
	Unclear risk if not specified in the paper
Baseline outcome measurements similar	Low risk if performance or patient outcomes were measured prior to the intervention, and no important differences were present across study groups. In randomised trials, score low irks if imbalanced bur appropriate adjusted analysis was performed
	High risk if important differences were present and not adjusted for in analysis
	If randomised trials have no baseline measure of outcome, score unclear risk
Baseline characteristics similar	Low risk if baseline characteristics of the study and control providers are reported and similar
	High risk if there is no report of characteristics in text or tables or if there are differences between control and intervention providers
	Unclear risk if it is not clear in the paper (e.g., characteristics are mentioned in text but no data were provided)
Incomplete outcome data	Low risk if missing outcome measures were unlikely to bias the results (e.g., the proportion of missing data were similar in the intervention and control groups or the proportion of missing data were less than the effect size)
	High risk if missing outcome data were likely to bias the results
	Unclear risk of not specified in the paper (without assuming 100% follow up unless explicitly stated)
Knowledge of the allocated interventions adequately prevented during study	Low risk if authors state explicitly that the primary outcome variables were assessed blindly, or the outcomes are objective
	High risk if the outcomes were not assessed blindly
	Unclear risk if not specified in the paper
Protection against contamination	Low risk if allocation was by community, institution or practice and it is unlikely that the control group received the intervention
	High risk if it is likely that the control group received the intervention
	Unclear risk if professionals were allocated within a clinic or practice and it is possible that communication between intervention and control professionals could have occurred
Selective outcome reporting	Low risk if there is no evidence that outcomes were selectively reported
	High risk if some important outcomes are omitted from the results
	Unclear risk if not specified in the paper
Other risks of bias (e.g. bias in measurement: validity and reliability of the measures used)	Low risk if there is no evidence of other risk of biases

**Table 3:** EPOC criteria for assessing risk of bias in interrupted time series studies

Criteria	Rating Scheme
Intervention independent of other changes	Low risk if there are compelling arguments that the intervention occurred independently of other changes over time and the outcome was not influenced by other confounding variables/historic events during study period
	High risk if reported that intervention was not independent of other changes in time
	Unclear risk if not specified in the paper
Shape of intervention effect pre‐specified	Low risk if point of analysis is the point of intervention or a rational explanation for the shape of intervention effect was given by the author(s)
	High risk if it is clear that the condition above is not met
	Unclear risk if not specified in the paper
Intervention unlikely to affect data collection	Low risk if reported that intervention itself was unlikely to affect data collection (for example, sources and methods of data collection were the same before and after the intervention)
	High risk if the intervention itself was likely to affect data collection (for example, any change in source or method of data collection reported)
	Unclear risk if not specified in the paper
Knowledge of the allocated interventions adequately prevented during study	Low risk if the authors state explicitly that the primary outcome variables were assessed blindly, or the outcomes are objective, e.g. length of hospital stay
	High risk if the outcomes were not assessed blindly
	Unclear risk if not specified in the paper
Incomplete outcome data	Low risk if missing outcome measures were unlikely to bias the results (e.g. the proportion of missing data were similar in the pre‐ and post‐intervention periods or the proportion of missing data were less than the effect size i.e. unlikely to overturn the study result)
	High risk if missing outcome data werelikely to bias the results
	Unclear risk if not specified in the paper (Do not assume 100% follow up unless stated explicitly)
Selective outcome reporting	Low risk if there is no evidence that outcomes were selectively reported (e.g. all relevant outcomes in the methods section are reported in the results section)
	High risk if some important outcomes are subsequently omitted from the results
	Unclear risk if not specified in the paper
Other risks of bias (e.g., bias in measurement: validity and reliability of the measures used)	Low risk if there is no evidence of other risk of biases

In addition, the Cochrane risk of bias (ROB) tool (Higgins & Green, 2011) was used for randomised studies, including cluster‐randomised studies and step‐wedge designs. The ROB tool used the following criteria for assessment of bias. Of note, we assessed performance and detection bias separately.

- Selection bias: random sequence generation and allocation concealment
- Performance bias: blinding of participants and personnel
- Detection bias: blinding of outcome assessment
- Attrition bias: incomplete outcome data
- Reporting bias: selective reporting
- Other sources of bias

All risk of bias assessments were performed in duplicate and supportive evidence for all ROB judgements was documented. A third reviewer resolved any disagreements. An overall score was not provided.

#### 4.3.6 Measures of treatment effect

We converted data for each outcome into the same format (e.g. means and standard deviations for continuous data), including appropriate conversion of scales such that an increase/decrease always indicated improvement or deterioration of an indicator. In the case that included studies had data that were reported in a not 'usable' way (i.e., data cannot be pooled with other data), we retained the study as eligible but restricted it from further analysis.

We analyzed dichotomous and continuous outcomes separately. For dichotomous outcomes, results were presented as summary risk ratios (RRs) with 95% CIs, whenever possible, in order to compare risk of the outcome between intervention and control groups. When including incidence data, we combined risk ratios (events per child) and rate ratios (events per child year) because of their similar interpretation and scale. We presented continuous outcome data as either a mean difference (MD), if outcomes were measured on the same scale, or a standardized mean difference (SMD), if outcomes were measured on different scales, with 95% CIs. Both change from baseline scores and final measurements (for RCTs only) were eligible, and were pooled where there is meta‐analysis with MD (i.e., scales are the same and measurements are in the same unit) Higgins & Green, 2011. We carefully considered reporting of the appropriate means and standard deviations (either of final measurements or of changes from baseline) if both change and final values were used in one meta‐analysis. We did not combine final values and change scores as SMDs because the standard deviation in this case reflected differences in measurement reliability. Where it was necessary to combine measures of treatment effects with SMDs (for example, in the case of child development outcomes), we used change scores as opposed to endline values, but only where authors had appropriately reported the SD of the change score.

#### 4.3.7 Unit of analysis issues

All interventions and, within those interventions, outcomes were meta‐analysed separately. We also meta‐analysed RCTs and quasi‐experimental studies separately.

Special attention was given to cluster‐randomised studies; this was to ensure that clustering was appropriately accounted for within the analysis of the primary study, such that study precision was not over or under‐estimated within our analysis. If necessary, we adjusted effect estimates of cluster‐randomised studies using the mean cluster size (M) and the intra‐cluster correlation coefficient (ICC), which quantified the extent to which data from the same cluster were correlated [design effect = 1 + (M‐1) ICC]. The design effect was then used to adjust the study data such that a study was reduced to its effective sample size. We did not make any adjustments if authors appropriately adjusted for cluster design already.

Randomized and non‐randomised studies with contemporaneous comparison groups were analysed separately, but were pooled if differences in findings were not statistically significant. We analyzed and reported findings from controlled before‐after and ITS study designs separately.

#### 4.3.8 Dealing with missing data

Where data was incomplete or in a form that could not be converted with the information available, we contacted the corresponding author for clarification or to obtain missing data. If authors have accounted for missing data (i.e. multiple imputations), we used the adjusted data within our analysis.

#### 4.3.9 Assessment of heterogeneity

Statistical heterogeneity was assessed using Tau^2^, I^2^ and significance of the Chi‐square test; we also assessed heterogeneity visually using forest plots. Based on prior theory and clinical knowledge, we expected clinical and methodological heterogeneity in effect sizes in this literature. Therefore, we attempted to explain any observed statistical heterogeneity using subgroup analysis (see below).

#### 4.3.10 Assessment of reporting biases

If the number of studies was sufficient (>10), funnel plots were used to visually assess publication bias. This kind of bias is unlikely if data forms a symmetric inverted funnel shape around the mean effect estimate. In addition, we performed Egger's test to determine funnel plot asymmetry.

#### 4.3.11 Data synthesis

Statistical analysis was carried out using Review Manager 5.3 and Stata. We followed intention to treat (ITT) analysis for RCTs. We reconstructed the data to create an ITT analysis where authors reported a per protocol analysis.

Random effects meta‐analysis was used to account for any expected heterogeneity in interventions, comparisons, outcomes, or settings within the studies included in a given synthesis. Where meta‐analysis was deemed inappropriate due to substantial methodological or statistical heterogeneity between studies, we summarized the findings of the included studies in narrative or table form.

The generic inverse‐variance approach was used for both dichotomous and continuous outcomes, such that the study weights were adjusted according to the variance of the effect estimate (i.e. the larger studies with smaller standard error were given more weight than smaller studies with larger standard error). For random effects analyses, the DerSimonian and Laird method was applied to incorporate a measure of variation (Tau^2^) among intervention effects from different studies.

We used raw summary estimates to construct meta‐analyses from RCTs and adjusted estimates to construct meta‐analyses from observational studies. We interpreted overall effect estimates that had an associated p‐value <0.05 as statistically significant, but also commented on those effects where the upper or lower confidence interval has just crossed the line of no effect. In the case of the latter, where the confidence intervals fell between the line of no effect and 0.06, the effect estimates were interpreted with caution as possibly significant (i.e. may have or may have not had an effect). Where confidence intervals were >0.06, effect estimates were interpreted as not significant.

We also reported non‐significant findings. Where possible, interaction tests were used to determine if there was a relevant difference in effect across sub‐groups. We based the conclusion that an intervention was effective in one subgroup but not another on a direct test of the mean difference between two groups (i.e., with meta‐regression).

We used the GRADE tool to assess the body of evidence for selected outcomes for which a meta‐analysis was conducted. We chose the following outcomes: maternal mortality, maternal anaemia, low birthweight, and perinatal mortality.We summarized this assessment in a 'Summary of Findings' table, created by the GRADEpro software. We rated the quality of the body of evidence for each selected outcome as high, moderate or low, or very low. Randomized studies were initially rated as high quality evidence but they were downgraded according to the five criteria listed below. Quasi‐experimental studies initially were rated as low quality evidence, but they could be upgraded if they did not have any serious methodological limitations. They could also be downgraded further.

There were five criteria that downgraded evidence (Atkins & GRADE, 2004):

- Risk of bias in individual studies
- Indirectness of evidence
- Unexplained heterogeneity or inconsistency of results
- Imprecision of results
- High probably of publication bias

There were three criteria that upgraded the evidence for quasi‐experimental studies with no serious methodological limitations. (Atkins & GRADE, 2004):

- Large magnitude of effect
- Presence of a dose response relationships
- Effect of plausible residual confounding

Quality ratings, as determined by GRADE, are found in Table 4.

**Table 4:** Quality of evidence, as determined by GRADE criteria

Quality	Description
Very low	Any estimate of effect is uncertain.
Low	Further research is very likely to have an important impact on our confidence in the estimate of effect and is likely to change the estimate.
Moderate	Further research is likely to have an important impact on our confidence in the estimate of effect and may change the estimate.
High	Further research is very unlikely to change our confidence in the estimate of effect.

Subgroup analysis and investigation of heterogeneity

Heterogeneity was assessed based on clinical knowledge and theory and investigation of statistical criteria such as Tau^2^, I^2^ and significance of the Chi‐sqaure test.

Depending on data availability (> or equal to three studies per subgroup of interest), we conducted sub‐group analyses on the primary outcomes for the following variables:

- Age (10‐14 years, 15‐19 years, 20‐29 years, 30‐39 years, 40+)
- Geographical region (based on WHO regions)
- Sex of infant
- Baseline nutritional status
  – Anemic versus non‐anemic
  – Undernutrition versus normal nutrition, based on body mass index (BMI; BMI <18.5)
  – Low stature versus normal stature
- Duration of intervention
  – Women recruited prior to conception versus first trimester versus second trimester versus third trimester of pregnancy
- Frequency of intervention
  – Daily versus intermittent IFA supplementation
- Dose of intervention
  – 30 mg versus 60 mg elemental iron for IFA, MMN, or LNS supplementation
- UNIMMAP versus adapted UNIMMAP versus non‐UNIMMAP formulations for MMN supplementation (UNICEF, WHO, and UNU, 1999)
  – MMN supplements that contained a similar number and type of vitamins and minerals as the UNIMMAP formulation were categorized as 'adapted UNIMMAP' (+/−2 micronutrients, when compared to the standard UNIMMAP formulation)
  – Supplements with the same composition as UNIMMAP but different doses of vitamins and minerals were categorized as 'adapted UNIMMAP'

Variables were selected *a priori*, based on evidence to support their potential to impact the intervention effect. We carefully interpreted results from subgroup analyses. We also used meta‐regression techniques to assess how characteristics of studies (explanatory variables) may influence the size of the effect estimate (outcome variable). Potential variables may include the setting, dosing frequency, dosing form, compound, duration, route, sex of infant, SES status, or nutritional status.

Any subgroup analysis that was conducted *post hoc was* exploratory in nature and was stated as such.

#### 4.3.12 Sensitivity analysis

Sensitivity analyses were conducted to determine whether the removal of studies with high risk of bias or the removal of non‐randomised studies significantly impacted findings. We defined studies as having a high risk of bias if one or more domains have been judged as 'high risk' or two or more domains have been judged as 'unclear risk'.

#### 4.3.13 Treatment of qualitative research

We did not include qualitative research.

## 5 RESULTS

### 5.1 Description of studies

#### 5.1.1 Results of the search

We identified of 27,987 records from our database search and 670 records through hand‐searching and searching grey literature (Figure 3). Following title and abstract screening, full text screening was completed for 1,678 papers (1,246 from the database search and 432 from the grey literature and hand‐search combined). Of these, a total of 72 studies, with 314 associated papers, were identified for inclusion based on our pre‐defined inclusion and exclusion criteria. Eight studies were included in the review but did not contribute data to the meta‐analyses for various reasons: data was in a form that could not be pooled with other studies, no reported outcomes of interest, or insufficient number of studies to pool for meta‐analysis; at minimum, 3 studies were required to conduct a meta‐analysis for a given outcome (Diogenes et al., 2013; Duggan et al., 2014; Gowachirapant et al., 2017; Hambidge et al., 2019; Jarjou et al., 2006; Korkmaz et al., 2014; Prawirohartono et al., 2011; Taherian et al., 2002).

#### 5.1.2 Included studies

We identified a total of 72 studies (number of included papers = 314), involving 451,723 women as eligible for inclusion in this review (Figure 3). Eight trials were included in the review, but did not contribute data to the meta‐analyses (Diogenes et al., 2013; Duggan et al., 2014; Gowachirapant et al., 2017; Hambidge et al., 2019; Jarjou et al., 2006; Korkmaz et al., 2014; Prawirohartono et al., 2011; Taherian et al., 2002). Of these, Gowachirapant et al. (2017) was the only study that examined iodine supplementation versus placebo and Hambidge et al. (2019) was the only study that reported the effects of LNS versus placebo. Diogenes et al. (2013) and Taherian et al. (2002) were excluded from meta‐analyses because they were the only two studies that fit our eligibility criteria and evaluated the impact of calcium plus vitamin D versus placebo supplementation. Jarjou et al. (2006) examined calcium supplementation versus placebo (for which we have conducted meta‐analyses); however, this study reported outcomes that could not be pooled with other studies. Similarly, Korkmaz et al. (2014) was excluded from the iron versus placebo comparison and Prawirohartono et al. (2011) was excluded from the vitamin A versus placebo comparison and the zinc versus placebo comparison due to no common outcomes of interest. Duggan et al. (2014) was the only study that reported effects of vitamin B12 supplementation compared to placebo.

Twelve studies were conducted in the East Asia Pacific region (Dijkhuizen et al., 2001; Gowachirapant et al., 2017; Hanieh et al., 2013; Huy et al., 2009; Liu et al., 2013; Muslimatun et al., 2001; Prawirohartono et al., 2011; Supplementation with Multiple Micronutrients Intervention Trial (SUMMIT) Study Group, 2008; Sunawang et al., 2009; Tanumihardjo, 2002; Zeng et al., 2008; Zhao et al., 2015). Of these, 2 were excluded from analysis (Gowachirapant et al., 2017; Prawirohartono et al., 2011). Countries represented include: China (Liu et al., 2013; Zeng et al., 2008; Zhao et al., 2015), Indonesia (Dijkhuizen et al., 2001; Muslimatun et al., 2001; Prawirohartono et al., 2011; SUMMIT Study Group, 2008; Sunawang et al., 2009; Tanumihardjo, 2002), Vietnam (Hanieh et al., 2013; Huy et al., 2009), and Thailand (Gowachirapant et al., 2017).

Only 1 study was conducted in Europe & Central Asia, in Turkey (Korkmaz et al., 2014).

Seven studies were conducted in Latin America & the Caribbean (Belizán et al., 1997; Castillo‐Durán et al., 2001; Caulfield et al.,^1999^; Diogenes et al., 2013; López‐Jaramillo et al., 1997; Merialdi et al., 2004; Ramakrishnan et al., 2003). Of these, 1 was excluded from analysis (Diogenes et al., 2013). Countries represented include: Argentina (Belizán et al., 1997), Chile (Castillo‐Durán et al., 2001), Peru (Caulfield et al., 1999; Merialdi et al., 2004), Brazil (Diogenes et al., 2013), Ecuador (López‐Jaramillo et al., 1997) and Mexico (Ramakrishnan et al., 2003).

Thirteen studies were conducted in the Middle‐East and North Africa region (Aminisani et al., 2009; Asemi et al., 2013; Asemi et al., 2016; Falahi, 2010; Mohammad‐Alizadeh‐Charandabi et al., 2015; Naghshineh & Sheikhaliyan, 2016; Ouladsahebmadarek et al., 2011; Sabet et al., 2012; Sorouri et al., 2016; Taherian et al., 2002; Vaziri et al., 2016; Ziaei et al., 2007, 2008). Of these, 1 was excluded from analysis (Taherian et al., 2002). All 13 studies were conducted in Iran.

Nineteen studies were conducted in South Asia (Ahmad et al., 2016; Bhutta et al., 2009; Choudhury et al., 2012; Christian et al., 2003; Duggan et al., 2014; Hafeez et al., 2005a, b; Hossain et al., 2012; Khan et al., 2016; Kumar et al., 2009; Osendarp et al., 2000; Osrin et al., 2005; Roth et al., 2013 (AViDD); Roth et al., 2018 (MDIG); Sablok et al., 2015; Sahu et al., 2009; Tofail et al., 2008; West et al., 1999; West et al., 2011; West et al., 2014). Approximately half of the studies (*n*=8) were conducted in Bangladesh (Ahmad et al., 2016; Choudhury et al., 2012; Osendarp et al., 2000; Roth et al., 2013 (AViDD); Roth et al., 2018 (MDIG); Tofail et al., 2008; West et al., 2011; West et al., 2014). Four studies were conducted in India (Duggan et al., 2014; Kumar et al., 2009; Sablok et al., 2015; Sahu et al., 2009), 3 in Nepal (Christian et al., 2003; Osrin et al., 2005; West et al., 1999), and 4 in Pakistan (Bhutta et al., 2009; Hafeez et al.,2005a, 2005b; Hossain et al., 2012; Khan et al., 2016).

Eighteen studies were conducted in Sub‐Saharan Africa (Ashorn et al., 2015; Cox et al., 2005; Dewey, 2009; Darling et al., 2017; Etheredge et al., 2015; Fawzi et al., 2007; Friis et al., 2004; Huybregts et al., 2009; Jarjou et al., 2006; Kæstel et al., 2005; Kirkwood et al., 2010; Menendez et al., 1995; Moore et al., 2012; Preziosi et al., 1997; Roberfroid et al., 2008; Saaka et al., 2009; Semba et al., 2001; Zagré et al., 2007). Of these, Jarjou et al., 2006 was the only study excluded from analysis. Countries represented include Burkina Faso (Huybregts et al., 2009; Roberfroid et al., 2008), The Gambia (Jarjou et al., 2006; Menendez et al., 1995; Moore et al., 2012), Ghana (Cox et al., 2005; Dewey, 2009; Kirkwood et al., 2010; Saaka et al., 2009), Guinea‐Bissau (Kæstel et al., 2005), Malawi (Ashorn et al., 2015; Semba et al., 2001), Niger (Preziosi et al., 1997; Zagré et al., 2007), Tanzania (Darling et al., 2017; Etheredge et al., 2015; Fawzi et al., 2007) and Zimbabwe (Friis et al., 2004).

Two studies, Villar et al. (2006) and Hambidge et al. (2019) were multi‐country studies. Villar et al. (2006) was conducted in India, Peru, South Africa and Vietnam; Hambidge et al. (2019) was conducted in Democratic Republic of Congo, Guatemala, India and Pakistan.

A total of 439,649 women participated in the remaining 64 studies (Ahmad et al., 2016; Aminisani et al., 2009; Asemi et al., 2013; Asemi et al., 2016; Ashorn et al., 2015; Belizán et al., 1997; Bhutta et al., 2009; Castillo‐Durán et al., 2001; Caulfield et al., 1999; Choudhury et al., 2012; Christian et al., 2003; Cox et al., 2005; Mohammad‐Alizadeh‐Charandabi et al., 2015; Dewey, 2009; Dijkhuizen et al., 2001; Etheredge et al., 2015; Falahi, 2010; Fawzi et al., 2007; Friis et al., 2004; Hafeez et al., 2005a, 2005b; Hanieh et al., 2013; Hossain et al., 2012; Huy et al., 2009; Huybregts et al., 2009; Kæstel et al., 2005; Khan et al., 2016; Kirkwood et al., 2010; Kumar et alk., 2009; Liu et al., 2013; López‐Jaramillo et al., 1997; Menendez et al., 1995; Merialdi et al., 2004; Moore et al., 2012; Muslimatun et al., 2001; Naghshineh & Sheikhaliyan, 2016; Osendarp et al., 2000; Osrin et al., 2005; Ouladsahebmadarek et al., 2011; Preziosi et al., 1997; Ramakrishnan et al., 2003; Roberfroid et al., 2008; Roth et al., 2013 (AViDD); Roth et al., 2018 (MDIG); Saaka et al., 2009; Sabet et al., 2012; Sablok et al., 2015; Sahu et al., 2009; Semba et al., 2001; Sorouri et al., 2016; SUMMIT Study Group, 2008; Sunawang et al., 2009; Tanumihardjo, 2002; Tofail et al., 2008; Vaziri et al., 2016; Villar et al., 2006; West et al., 1999; West et al., 2011; West et al., 2014; Zagré et al., 2007; Zeng et al., 2008; Zhao et al., 2015; Ziaei et al., 2007, 2008).

Most of the outcomes were defined in the same way across different studies. The exception to this was in Christian et al., (2003) and Liu et al., 2013, where perinatal mortality was defined as stillbirths (from 28 weeks of gestation to delivery) and early neonatal death from birth to 6 days after delivery; this is compared to all other studies that defined perinatal mortality as stillbirths plus early neonatal deaths from birth to 7 days after delivery. For iron deficiency (measured by serum ferritin levels), Zhao et al., 2015 defined iron deficiency as a serum ferritin of <15ug/L, while the remaining studies (Falahi, 2010; Liu et al., 2013; Preziosi et al., 1997) reported it as a serum ferritin of <12ug/L. Lastly, Villar 2006 combined pre‐eclampsia and eclampsia cases; the other studies only considered pre‐eclampsia cases.

All studies reported sources of funding except: Asemi et al., 2013; Caulfield et al.,^1999^; Dijkhuizen et al., 2001; Falahi, 2010; Huy et al., 2009; Huybregts et al., 2009; Jarjou et al., 2006; Korkmaz et al., 2014; López‐Jaramillo et al., 1997; Menendez et al., 1995; Merialdi et al., 2004; Mohammad‐Alizadeh‐Charandabi et al., 2015; Muslimatun et al., 2001; Osendarp et al., 2000; Ouladsahebmadarek et al., 2011; Preziosi et al.,^1997^; Saaka et al., 2009; Sablok et al., 2015; Tanumihardjo, 2002; Villar et al., 2006; Ziaei et al., 2007, 2008.

Forty‐two studies included a statement of disclosure regarding potential conflicts of interest related to the study (Ahmad et al., 2016; Asemi 2016; Belizán et al., 1997; Castillo‐Durán et al., 2001; Christian et al., 2003; Dijkhuizen et al., 2001; Diogenes et al., 2013; Duggan et al., 2014; Etheredge et al., 2015; Fawzi et al., 2007; Friis et al., 2004; Gowachirapant et al., 2017; Hambidge et al., 2019; Hanieh et al., 2013; Hossain et al., 2012; Jarjou et al., 2006; Kæstel et al., 2005; Khan et al., 2016; Kirkwood et al., 2010; Korkmaz et al., 2014; Liu et al., 2013; Mohammad‐Alizadeh‐Charandabi et al., 2015; Moore et al., 2012; Ramakrishnan et al., 2003; Roberfroid et al., 2008; Roth et al., 2013; Hafeez et al., 2005a, 2005b; (AViDD); Roth et al., 2018 (MDIG); Sabet et al., 2012; Sablok et al., 2015; Sahu et al., 2009; Sorouri et al., 2016; SUMMIT Study Group, 2008; Tofail et al., 2008; Vaziri et al., 2016; West et al., 2011; Zhao et al., 2015; Ashorn et al., 2015; Dewey, 2009; Osrin et al., 2005; West et al., 2014; Zeng et al., 2008).

Of these, 37 studies (Ahmad et al., 2016; Asemi et al., 2016; Belizán et al.,^1997^; Castillo‐Durán et al., 2001; Christian et al., 2003; Dijkhuizen et al., 2001; Diogenes et al., 2013; Duggan et al., 2014; Etheredge et al., 2015; Fawzi et al., 2007; Friis et al., 2004; Gowachirapant et al., 2017; Hafeez et al., 2005a, 2005b; Hambidge et al., 2019; Hanieh et al., 2013; Hossain et al., 2012; Jarjou et al., 2006; Kæstel et al., 2005; Khan et al., 2016; Kirkwood et al., 2010; Korkmaz et al., 2014; Liu et al., 2013; Mohammad‐Alizadeh‐Charandabi et al., 2015; Moore et al., 2012; Ramakrishnan et al., 2003; Roberfroid et al., 2008; Roth et al., 2013 (AViDD); Roth et al., 2018 (MDIG); Sabet et al., 2012; Sablok et al., 2015; Sahu et al., 2009; Sorouri et al., 2016; SUMMIT Study Group, 2008; Tofail et al., 2008; Vaziri et al., 2016; West et al., 2011; Zhao et al., 2015) declared no conflict of interest amongst all authors. The five remaining studies (Ashorn et al., 2015; Dewey, 2009; Osrin et al., 2005; West et al., 2014; Zeng et al., 2008) indicated that one or several of the study's authors had a conflict of interest.

The remaining 30 studies (Aminisani et al., 2009; Asemi et al., 2013; Bhutta et al., 2009; Caulfield et al., 1999; Choudhury et al., 2012; Cox et al., 2005; Darling et al., 2017; Falahi, 2010; Huy et al., 2009; Huybregts et al., 2009; Kumar et al., 2009; López‐Jaramillo et al., 1997; Menendez et al., 1995; Merialdi et al., 2004; Muslimatun et al., 2001; Naghshineh & Sheikhaliyan, 2016; Osendarp et al., 2000; Ouladsahebmadarek et al., 2011; Prawirohartono et al., 2011; Preziosi et al., 1997; Saaka et al., 2009; Semba et al., 2001; Sunawang et al., 2009; Taherian et al., 2002; Tanumihardjo, 2002; Villar et al., 2006; West et al., 1999; Zagré et al., 2007; Ziaei et al., 2007, 2008) did not provide any statement of disclosure, and thus we could not comment on any potential conflicts of interest.

### 5.2 Participants

The 64 studies included in the data analyses involved 439,649 women at varying gestational ages at baseline, ranging from early pregnancy (<20 weeks of gestation) to <37 weeks of gestation. Most participants were enrolled in the studies at <or equal to 20 weeks of gestation. Included participants were healthy and without anaemia (i.e. were not recruited based on anemia status), any chronic or systemic medical condition (e.g. cardiac disease, tuberculosis, human immuno‐deficiency virus (HIV)), or heightened risk of pregnancy complication (e.g. history of pre‐eclampsia/eclampsia). Two studies (Ashorn et al., 2015; Friis et al., 2004) included a subgroup of pregnant women who were HIV‐positive; however, the data for these subgroups were not included in this review. Across all studies, baseline characteristics of participants in the intervention and control groups were comparable, except for the following studies: Christian et al (2003); Friis et al. (2004); Ramakrishnan et al. (2003); Roberfroid et al. (2008); Zagré et al. (2007). In Christian et al., (2003), the control group included more participants who represented a specific ethnic background and owned land compared to the intervention group. In Friis et al. (2004), there were more primigravidae participants in the placebo/control group than in the intervention group. In Ramakrishnan et al. (2003), the intervention group had a higher proportion of single mothers and participants with lower mean BMI than in the control group. In Roberfroid et al. 2008, the serum hemoglobin (Hb) level of participants in the intervention group was lower than in the control group, and maternal BMI was lower in the control group than in the intervention group. Finally, in Zagré et al. (2007) the placebo group included more participants who were less educated and living in poverty than in the intervention group; as well, there were more households and preventive measures against malaria amongst participants in the intervention group.

### 5.3 Intervention

All supplements were given orally and in the form of tablets (except for lipid‐based supplements). Supplements were given to pregnant women throughout the remainder of their pregnancy from the time of enrolment.

### 5.4 IFA Supplementation versus Folic Acid Supplementation

Seven studies assessed IFA supplementation compared to folic acid supplementation or placebo (Christian et al., 2003; Etheredge et al., 2015; Liu et al., 2013; Menendez et al., 1995; Zeng et al., 2008; Zhao et al., 2015; Ziaei et al., 2007). Of these, 5 were included in the iron versus placebo comparison, with folic acid supplementation provided as a co‐intervention (Christian et al., 2003; Etheredge et al., 2015; Liu et al., 2013; Menendez et al., 1995; Ziaei et al., 2007). Three studies had multiple intervention arms and were included in other comparisons: Christian et al., 2003; Liu et al., 2013; Zeng et al., 2008. Studies provided 30 mg to 60 mg of iron, with the majority providing 60 mg of iron, and 400‐500 ug of folic acid in their supplements.

### 5.5 MMN supplementation versus IFA supplementation or placebo

Thirty‐four studies assessed MMN versus IFA supplementation (Aminisani et al., 2009; Asemi et al., 2016; Ashorn et al., 2015; Bhutta et al., 2009; Caulfield et al., 1999; Choudhury et al., 2012; Christian et al. ,^2003^; Dewey, 2009; Dijkhuizen et al., 2001; Fawzi et al., 2007; Friis et al., 2004; Hafeez et al., 2005a, 2005b; Hanieh et al., 2013; Huy et al., 2009; Kæstel et al., 2005; Liu et al., 2013; Merialdi et al., 2004; Moore et al., 2012; Muslimatun et al., 2001; Osrin et al., 2005; Ramakrishnan et al., 2003; Roberfroid et al., 2008; Roth et al., 2013 (AViDD); Roth et al., 2018 (MDIG); Saaka et al., 2009; Semba et al., 2001; Sorouri et al., 2016; SUMMIT Study Group, 2008; Sunawang et al., 2009; Tofail et al., 2008; Villar et al., 2006; West et al., 2014; Zagré et al., 2007; Zeng et al., 2008).

For this review, MMN was defined as the provision of at least 3 micronutrients (e.g. iron, folic acid and vitamin A). Trials could be considered MMN even in situations where iron with or without folic acid was provided in a separate supplement. Thus, it is possible that these studies could be included in other comparisons as well. For example, where a study investigates the effects of vitamin A versus placebo supplementation, but all women are provided IFA as the standard of care, then data from this study would be included in the vitamin A versus placebo and MMN vs. IFA comparisons. There were twelve of these types of studies (Aminisani et al., 2009; Caulfield et al., 1999; Dijkhuizen et al., 2001; Hafeez et al., 2005a, 2005b; Merialdi et al., 2004; Muslimatun et al., 2001; Roth et al., 2013 (AViDD); Roth et al., 2018 (MDIG); Saaka et al., 2009; Semba et al., 2001; Sorouri et al., 2016; and Villar et al., 2006) that were included in other comparisons; mainly zinc versus placebo and vitamin A versus placebo. Asemi et al. (2016) evaluated calcium with vitamin D supplementation versus placebo (plus IFA), and was included in the MMN versus IFA comparison; however, this study was not included in any other comparison given the insufficient number of studies to undertake meta‐analyses for calcium with vitamin D supplementation. Five studies had multiple intervention arms and were included in other comparisons: Ashorn et al., 2015; Christian et al., 2003; Dewey, 2009; Liu et al., 2013; Moore et al., 2012; Zeng et al., 2008.

The composition of MMN supplement varied across all studies. Eleven studies used the UNIMMAP formulation, developed by UNICEF, WHO and United Nations University (Bhutta et al., 2009; Huy et al., 2009; Kæstel et al., 2005; Liu et al., 2013; Osrin et al., 2005; Roberfroid et al., 2008; SUMMIT Study Group, 2008; Sunawang et al., 2009; Tofail et al., 2008; Zagré et al., 2007; Zeng et al., 2008). Another 3 studies used an adapted UNIMMAP formulation which contained the exact same combination of vitamins and minerals, but in different dosages (Hanieh et al., 2013; Moore et al., 2012; West et al., 2014). The remaining 20 studies used non‐UNIMMAP formulations for their MMN supplements (Aminisani et al., 2009; Asemi et al., 2016; Ashorn et al., 2015; Caulfield et al., 1999; Choudhury et al., 2012; Christian et al., 2003; Dewey, 2009; Dijkhuizen et al., 2001; Fawzi et al., 2007; Friis et al., 2004; Hafeez et al., 2005a, 2005b; Merialdi et al., 2004; Muslimatun et al., 2001; Ramakrishnan et al., 2003; Roth et al., 2013 (AViDD); Roth et al., 2018 (MDIG); Saaka et al., 2009; Semba et al., 2001; Sorouri et al., 2016 and Villar et al., 2006).

The dose of iron in the MMN supplement differed across all studies. The studies that used UNIMMAP all contained 30 mg of elemental iron (Bhutta et al., 2009; Huy et al., 2009; Kæstel et al., 2005; Liu et al., 2013; Osrin et al., 2005; Roberfroid et al., 2008; SUMMIT Study Group, 2008; Sunawang et al., 2009; Tofail et al., 2008; Zagré et al., 2007; Zeng et al., 2008). Of the studies that used an adapted UNIMMAP formulation, 2 studies used 60 mg of iron (Hanieh et al., 2013; Moore et al., 2012) and 1 study used 27 mg of iron (West et al., 2014). Of the studies that used a non‐UNIMMAP formulation for the MMN supplement, 7 studies included 60 mg of iron in their formulation (Caulfield et al., 1999; Choudhury et al., 2012; Christian et al., 2003; Fawzi et al., 2007; Merialdi et al., 2004; Muslimatun et al., 2001; Ramakrishnan et al., 2003). Seven other studies used dosages of iron <60 mg, ranging from 20 mg to 40 mg (Aminisani et al., 2009; Ashorn et al., 2015; Dewey et al., 2009; Dijkhuizen et al., 2001; Saaka et al., 2009; Semba et al., 2001; Sorouri et al., 2016), while 2 studies used 66 mg of iron (Roth et al., 2013 (AViDD); Roth et al., 2018 (MDIG). Two studies (Friis et al., 2004; Hafeez et al., 2005a, 2005b) did not indicate the dose of iron in the MMN supplement.

### 5.6 Lipid‐based nutrient supplementation versus MMN supplementation or placebo (control)

Four studies assessed LNS supplementation versus MMN supplementation or placebo (Ashorn et al., 2015; Dewey et al., 2009; Huybregts et al., 2009; Moore et al., 2012). Ashorn et al. (2015) and Dewey, 2009 utilized the same formulation for the LNS supplement, presented in a powder form: 20 g sachets containing 2.6 g protein, 10 g fat, 4.59 g linoleic acid, 0.59 g linolenic acid. These were given alongside a non‐UNIMMAP formulation of multiple micronutrients. Huybregts et al. (2009) also provided a powder that contained 1.56 MJ of energy: carbohydrates 15.9 g, protein 14.7 g, fat 27.6 g, MUFA 12.1 g, PUFA 7.3 g, Omega‐3 fatty acids 0.4 g, omega‐6 fatty acids 7.0 g), 9.1 g total dietary fibre; this was given alongside a UNIMMAP MMN supplement. Moore et al. (2012) provided LNS that contained energy (746 kcal), protein (20.8 g), lipids (52.6 g), alongside a non‐UNIMMAP MMN supplement.

### 5.7 Vitamin A versus placebo

Nine studies assessed vitamin A supplementation versus placebo (Cox et al., 2005; Darling et al., 2017; Kirkwood et al., 2010; Muslimatun et al., 2001; Prawirohartono et al., 2011; Semba et al., 2001; Tanumihardjo, 2002; West et al., 1999; West et al., 2011). Of these, 1 study was excluded from analysis (Prawirohartono et al., 2011). Studies provided vitamin A in doses ranging from 10,000 IU of retinol weekly to 70,000 IU of retinol weekly, with the majority of studies providing 17,000‐25,000 IU of retinol weekly.

### 5.8 Zinc versus placebo

Thirteen studies assessed zinc supplementation versus placebo (Ahmad et al., 2016; Aminisani et al., 2009; Castillo‐Durán et al., 2001; Caulfield et al., 1999; Christian et al., 2003; Darling et al., 2017; Dijkhuizen et al., 2001; Hafeez et al., 2005a, 2005b; Merialdi et al., 2004; Osendarp et al., 2000; Prawirohartono et al., 2011; Saaka et al., 2009; Sorouri et al., 2016). Of these, 1 was excluded from analysis (Prawirohartono et al., 2011). Studies provided zinc in doses ranging from 15 mg to 50 mg of zinc sulphate daily. The majority of studies provided 20 to 30 mg of zinc daily.

### 5.9 Iron versus placebo

Thirteen studies assessed iron supplementation versus placebo (Christian et al., 2003; Etheredge et al., 2015; Falahi et al., 2010; Korkmaz et al., 2014; Liu et al., 2013; Menendez et al., 1995; Ouladsahebmadarek et al., 2011; Preziosi et al., 1997; Tanumihardjo, 2002; Zeng et al., 2008; Zhao et al., 2015; Ziaei et al., 2007, 2008). Of these, 1 was excluded from analysis (Korkmaz et al., 2014). Studies provided iron in doses ranging from 30 mg to 100 mg. The majority of studies provided 50 or 60 mg of iron; one study (Ouladsahebmadarek et al., 2011) provided 30 mg of iron and Preziosi et al., 1997 provided 100 mg.

### 5.10 Vitamin D versus placebo

Eleven studies assessed vitamin D supplementation versus placebo (Asemi et al., 2013; Hossain et al., 2012; Khan et al., 2016; Mohammad‐Alizadeh‐Charandabi et al., 2015; Naghshineh & Sheikhaliyan, 2016; Roth et al., 2013 (AViDD); Roth et al., 2018 (MDIG); Sabet et al., 2012; Sablok et al., 2015; Sahu et al., 2009; Vaziri et al., 2016). Studies provided doses of vitamin D ranging from 2800 IU to 75,000 IU of vitamin D weekly. The majority of studies provided vitamin D in doses ranging from 10,000 to 25,000 IU per week.

### 5.11 Calcium versus placebo

Five studies assessed calcium supplementation versus placebo (Belizán et al., 1997; Jarjou et al., 2006; Kumar et al., 2009; López‐Jaramillo et al., 1997; Villar et al., 2006). Of these, 1 study was excluded from being pooled with other studies in meta‐analysis (Jarjou et al., 2006). Studies provided calcium supplementation in doses ranging from 500 mg daily to 2000 mg of calcium daily.

### 5.12 Excluded studies

We excluded 1,364 studies from this review. Of these, 434 studies were excluded based on an ineligible intervention; 466 were of the wrong study design; 353 involved the wrong population (e.g. high‐income country or child population); 75 reported ineligible outcomes; 17 involved the wrong comparison; 5 were abstracts; and 14 studies were inaccessible. Please see the Characteristics of excluded studies for the excluded studies found through our grey literature and hand‐searches, and their respective reasons for exclusion (n = 295). For the remaining excluded studies (n = 1069) found through the various database searches and their respective reasons for exclusion, please refer to Tables 5, 6, 7, 8, 9, 10, 11, 12, 13, 14, 15, 16.

**Table 5:** Characteristics Table of Excluded Studies (Database Searches)

Authors	Title	Published Year	Journal	Exclusion Reason
Aalaii, M. R., Kermaniyan, M., Moosavi, F. S., Kermaniyan, M.	Iron supplemention and the prevalence and anaemia in pregnant women in Ardakan city	2016	Toloo e Behdasht	Exclusion reason: Wrong study design
Imdad A., Bhutta, Z. A.	Nutritional management of the low birth weight/preterm infant in community settings: a perspective from the developing world	2013	Journal of Pediatrics	Exclusion reason: Wrong study design
Aaron, G. J., Dror, D. K., Yang, Z.	Multiple‐micronutrient fortified non‐dairy beverage interventions reduce the risk of anaemia and iron deficiency in school‐aged children in low‐middle income countries: A systematic review and meta‐analysis(i‐iv)	2015	Nutrients	Exclusion reason: Wrong intervention
Aaron, G. J., Sodani, P. R., Rajan, Sankar, Fairhurst, J., Siling, K., Guevarra, E., Norris, A., Myatt, M.	Household coverage of fortified staple food commodities in Rajasthan, India	2016	PLoS ONE [Electronic Resource]	Exclusion reason: Wrong study design
Aaron, G. J., Dror, D. K., Yang, Z.	Multiple‐Micronutrient Fortified Non‐Dairy Beverage Interventions Reduce the Risk of Anemia and Iron Deficiency in School‐Aged Children in Low‐Middle Income Countries: A Systematic Review and Meta‐Analysis (i‐iv)	2015	Nutrients	Exclusion reason: Wrong study design
Ababiya, T., Gabriel, T.	Prevalence of anaemia among pregnant women in Ethiopia and its management: A review	2014	International Research Journal of Pharmacy	Exclusion reason: Wrong study design
Abdollahi, Z., Elmadfa, I., Djazayeri, A., Sadeghian, S., Freisling, H., Salehi Mazandarani, F., Mohamed, K.	Folate, vitamin B_12_ and homocysteine status in women of childbearing age: Baseline data of folic acid wheat flour fortification in Iran	2008	Annals of Nutrition and Metabolism	Exclusion reason: Wrong patient population
Abdullahi, H., Gasim, G. I., Saeed, A., Imam, A. M., Adam, I.	Antenatal iron and folic acid supplementation use by pregnant women in Khartoum, Sudan	2014	BMC Research Notes	Exclusion reason: Wrong intervention
Abdul‐Rahman, A. M.	Adherence to folic acid supplements during peri‐conceptional period	2015	International Journal of Current Microbiology and Applied Sciences	Exclusion reason: Wrong study design
Abe, S. K., Balogun, O. O., Ota, E.,Takahashi, K.,; Mori, R.	Supplementation with multiple micronutrients for breastfeeding women for improving outcomes for the mother and baby	2016	Cochrane Database of Systematic Reviews	Exclusion reason: Wrong study design
Abebe, Y., Stoecker, B. J., Hinds, M. J., Gates, G. E.	Nutritive value and sensory acceptability of corn‐ and kocho‐based foods supplemented with legumes for infant feeding in Southern Ethiopia	2006	African Journal of Food, Agriculture, Nutrition and Development	Exclusion reason: Wrong intervention
Abebe, Z., Haki, G. D., Baye, K.	Simulated effects of home fortification of complementary foods with micronutrient powders on risk of inadequate and excessive intakes in West Gojjam, Ethiopia	2018	Maternal & Child Nutrition	Exclusion reason: Wrong patient population
Abedi, P., Mohaghegh, Z., Afshary, P., Latifi, M.	The relationship of serum vitamin D with pre‐eclampsia in the Iranian women	2014	Maternal and Child Nutrition	Exclusion reason: Wrong intervention
Abiaka, C., Machado, L., Mathew, M., Rao, K.	Erythrocyte indices, microminerals and ratios, antioxidants and lipids in centrum materna diet‐supplemented Omani mothers	2008	Biological Trace Element Research	Exclusion reason: Wrong patient population
Abir, T., Ogbo, F. A., Stevens, G. J., Page, A. N., Milton, A. H., Agho, K. E.	The impact of antenatal care, iron‐folic acid supplementation and tetanus toxoid vaccination during pregnancy on child mortality in Bangladesh	2017	PLoS ONE	Exclusion reason: Wrong study design
Abizari, A.‐R., Azupogo, F., Brouwer, I. D.	Subclinical inflammation influences the association between vitamin A‐ and iron status among schoolchildren in Ghana	2017	PLoS ONE	Exclusion reason: Wrong patient population
Abizari, A.‐R., Azupogo, F., Nagasu, M., Creemers, N., Brouwer, I. D.	Seasonality affects dietary diversity of school‐age children in northern Ghana	2017	PLoS ONE	Exclusion reason: Wrong study design
Aboud, F. E., Sadika, A.	A cluster‐randomized evaluation of a responsive stimulation and feeding intervention in Bangladesh	2011	Pediatrics	Exclusion reason: Wrong patient population
Abubaker, W. A., Al‐Assaf, A. F., Cleaver, V. L.	Quality assurance and iron deficiency in Egypt	1999	International Journal for Quality in Health Care	Exclusion reason: Wrong intervention
Abu‐Ouf, N. M., Jan, M. M.	The impact of maternal iron deficiency and iron deficiency anaemia on child's health	2015	Saudi Medical Journal	Exclusion reason: Wrong study design
Ackgoz, A., Gunay, T., Ucku, R.	Vitamin D requirements and supplementation during pregnancy	2013	Turk Silahl Kuvvetleri, Koruyucu Hekimlik Bulteni	Exclusion reason: Wrong study design; Article is in Turkish
ACTRN12606000357550, Makrides M., Zhou J.	Can low dose iron be used to treat anaemia in pregnancy effectively?	2005		Exclusion reason: Wrong patient population
ACTRN12610000483055, University of Auckland, University of Otago	Randomised placebo controlled study of vitamin D during pregnancy and infancy	2010		Exclusion reason: Wrong patient population
ACTRN12610000735055, Government body, Women and Children's Health Research Institute	Fish oil supplementation in pregnancy to reduce allergies in early childhood	2010		Exclusion reason: Wrong patient population
ACTRN12610000944033, University of Melbourne, Research and Training Center for Community Development	Which iron supplementation regime for pregnant women provides the best maternal and infant outcomes?	2010		Exclusion reason: Wrong patient population
ACTRN12610001044011, Charities/Societies/Foundations, Newtown Union Health Service, Wellington Medical Research Foundation	Vitamin D deficiency in Pregnancy ‐ A comparison of two treatments	2010		Exclusion reason: Wrong patient population
ACTRN12611001125910, Government body, NH&MRC	Follow‐up of children whose mothers participated in the DOMINO trial: does fish oil supplementation in pregnancy influence child development at 4 years?	2011		Exclusion reason: Wrong patient population
ACTRN12612000196842, Wickens, K., Crane, K.	A maternal probiotic intervention for infant allergic disease prevention	2012		Exclusion reason: Wrong patient population
ACTRN12612000241831, Individual, Brough, L.; Massey University	Dietary intake of micronutrients during pregnancy and breastfeeding after government initiatives to increase iodine intake	2012		Exclusion reason: Wrong patient population
ACTRN12612000588897, University of Sydney, International Centre for Diarrhoeal Disease Research, Bangladesh	A trial to evaluate the impact of an early start to iron/folic acid supplementation in pregnancy on deaths of newborns in rural Bangladesh	2012		Exclusion reason: Wrong patient population
ACTRN12612001145897, Hospital, Westmead Hospital, University of Western Sydney	Does Vitamin D supplementation in pregnancy improve maternal glucose metabolism or prevent gestational diabetes?	2012		Exclusion reason: Wrong patient population
ACTRN12613000853741, Launceston General Hospital, Clifford Craig Medical Research Trust	Evaluation of a single iron infusion versus oral iron tablets in the treatment of pregnancy anaemia among Tasmanian women	2013		Exclusion reason: Wrong patient population
ACTRN12613001142729, South Australian Health and Medical Research Institute	Omega‐3 fats to reduce the incidence of prematurity: the ORIP trial	2013		Exclusion reason: Wrong patient population
ACTRN12613001354774, Benha university hospital	Do nitric oxide donor drugs decrease the incidence of pre‐eclampsia in high risk teenagers pregnant for the first time?	2013		Exclusion reason: Wrong intervention
ACTRN12614000985684, The University of Adelaide	Screening Tests to identify poor Outcomes in Pregnancy (STOP) Study	2014		Exclusion reason: Wrong intervention
ACTRN12614000988651, University of Sydney	Should we treat iron deficiency anaemia of pregnancy with lactoferrin? A randomised controlled trial Lactoferrin Evaluation in Anaemia in Pregnancy	2014		Exclusion reason: Wrong patient population
ACTRN12615000400561, University of Auckland, Counties Manukau Health	A four‐armed randomised controlled demonstration trial of a multifaceted dietary intervention and probiotic capsules in obese pregnant women in the Counties Manukau Health region	2015		Exclusion reason: Wrong patient population
ACTRN12615001075572, Telethon Kids Institute	Investigating the effect of maternal prebiotic fibre supplementation during pregnancy and breastfeeding on the prevention of early childhood allergies	2015		Exclusion reason: Wrong patient population
ACTRN12616000080426, Chowdhury, M.	Promotion of a balanced diet for pregnant women to improve birthweight of infants: a cluster randomised controlled trial in rural Bangladesh	2016		Exclusion reason: Wrong intervention
ACTRN12617000354381, Rucklidge, K., Mulder, R.	A micronutrient intervention for pregnant women experiencing symptoms of depression and anxiety	2017		Exclusion reason: Wrong patient population
ACTRN12617000404325, The University of Adelaide, Women's and Children's Health Research Institute	Does maternal omega‐3 fatty acid supplementation reduce body fat mass at 7 years of age?	2017		Exclusion reason: Wrong patient population
ACTRN12617000442303, Monash University, Research and Training Centre for Community Development	Learning Clubs to improve women's perinatal health and early childhood development	2017		Exclusion reason: Wrong intervention
ACTRN12617001078347, Liggins Institute, University of Auckland	Fish oil in pregnancy for a healthy start to life for the children of overweight mothers	2017		Exclusion reason: Wrong patient population
Adams, K. P., Ayifah, E., Phiri, T. E., Mridha, M. K., Adu‐Afarwuah, S., Arimond, M., Arnold, C. D., Cummins, J., Hussain, S., Kumwenda, C., Matias, S. L., Ashorn, U., Lartey, A., Maleta, K. M., Vosti, S. A., Dewey, K. G.	Maternal and child supplementation with lipid‐based nutrient supplements, but not child supplementation alone, decreases self‐reported household food insecurity in some settings	2017	Journal of Nutrition	Exclusion reason: Wrong outcomes
Adams, K. P., Arimond, M., Dewey, K. G., Vosti, S. A., Ayifah, E., Phiri, T. E., Adu‐Afarwuah, S., Maleta, K., Ashorn, U.	Willingness to pay for small‐quantity lipid‐based nutrient supplements for women and children: Evidence from Ghana and Malawi	2018	Maternal & Child Nutrition	Exclusion reason: Wrong outcomes
Adanikin, A. I., Awoleke, J. O., Olofinbiyi, B. A., Adanikin, P. O., Ogundare, O. R.	Routine iron supplementation and anaemia by third trimester in a Nigerian hospital	2015	Ethiopian Journal of Health Sciences	Exclusion reason: Wrong study design
Adeleye, A. O., Joel‐Medewase, V. I.	Awareness and uptake of measures for preventing CNS birth defects among mothers of affected children in a sub‐Saharan African neurosurgeon's practice	2015	Childs Nervous System	Exclusion reason: Wrong intervention
Adewuya, T. O.	Impact of a newly designed food complement (food multimix) on nutritional status and birth outcomes of pregnant women in the gauteng province of south africa	2009		Exclusion reason: Wrong intervention
Adu‐Afarwuah, S., Lartey, A., Dewey, K. G.	Meeting nutritional needs in the first 1000 days: a place for small‐quantity lipid‐based nutrient supplements	2017	Annals of the New York Academy of Sciences	Exclusion reason: Wrong study design
Adu‐Afarwuah, S., Lartey, A., Okronipa, H., Ashorn, P., Arimond, M., Dewey, K. G.	Prenatal supplementation with small‐quantity lipid‐based nutrient supplements or multiple micronutrients increases urinary iodine concentration in semi‐urban Ghana: A randomized controlled trial	2017	Annals of Nutrition and Metabolism	Exclusion reason: Wrong study design
Afam‐Anene, O. C., Uwaegbute, A. C.	Socio‐economic variables associated with anaemia during pregnancy in Owerri IMO State, Nigeria	2016	FASEB Journal. Conference: Experimental Biology	Exclusion reason: Wrong intervention
Agarwal, A. K., Sen, A. K., Kalra, N. K., Gupta, N.	Prevalence of anaemia during pregnancy in district Burdwan, West Bengal	1999	Indian Journal of Public Health	Exclusion reason: Wrong intervention
Agarwal, K.	Eliminating vitamin A deficiency through early supplementation	2007	Indian Journal of Pediatrics	Exclusion reason: Wrong study design
Agbozo, F., Abubakari, A., Jahn, A.	Does gestational intake of adequate diets using the FAO women's dietary diversity indicator affect haemoglobin levels at delivery and newborn health outcomes? Preliminary findings from a prospective cohort study in Volta region, Ghana	2017	Tropical Medicine and International Health	Exclusion reason: Wrong study design
Aggarwal, A., Kumhar, G. D., Harit, D., Faridi, M. M.	Role of folic acid supplementation in prevention of neural tube defects: physicians yet unaware!	2010	Journal of Preventive Medicine & Hygiene	Exclusion reason: Wrong study design
Aggarwal, D., Sachdev, H. P. S., Nagpal, J, Singh. T., Mallika, V.	Haematological effect of iron supplementation in breast fed term low birth weight infants	2005	Archives of Disease in Childhood	Exclusion reason: Wrong patient population
Agrawal, S.	Is intravenous iron sucrose an alternative to the oral iron‐folate supplementation for treating iron deficiency anaemia in pregnant and post natal women	2012	International Journal of Gynecology and Obstetrics	Exclusion reason: Wrong study design
Agrawal, S., Fledderjohann, J., Vellakkal, S., Stuckler, D.	Adequately diversified dietary intake and iron and folic acid supplementation during pregnancy is associated with reduced occurrence of symptoms suggestive of pre‐eclampsia or eclampsia in indian women	2015	PLoS ONE	Exclusion reason: Wrong study design
Aguayo, V. M., Kone, D., Bamba, S. I., Diallo, B., Sidibe, Y., Traore, D., Signe, P., Baker, S. K.	Acceptability of multiple micronutrient supplements by pregnant and lactating women in Mali	2005	Public Health Nutrition	Exclusion reason: Wrong outcomes
Ahmed, T., Choudhury, N. Hossain, M. I., Islam, M. M., Schumacher, B., De Pee, S., Tangsuphoom, N., Muiruri, J., Fuli, R., Parveen, M., Sarker, S.;, West Jr, K. P., Christian, P.	Development and acceptability of locally developed ready‐to‐use complementary‐food‐supplements (RUCFS) in urban slum settings of dhaka, bangladesh	2013	Annals of Nutrition and Metabolism	Exclusion reason: Paediatric population
Ahmed, T., Choudhury, N., Hossain, M. I., Tangsuphoom, N., Islam, M. M., de Pee, S., Steiger, G., Fuli, R., Sarker, S. A. M., Parveen, M., West Jr, K. P., Christian, P.	Development and acceptability testing of ready‐to‐use supplementary food made from locally available food ingredients in Bangladesh	2014	BMC Pediatrics	Exclusion reason: Paediatric population
Aidam, B. A., Edward, A., Paden, A. C., Wong, R. Y. S., Chege, J.	Addressing anaemia in women and children in rural communities of Cambodia and Kenya: Experiences from an integrated program	2016	FASEB Journal. Conference: Experimental Biology	Exclusion reason: Wrong study design
Aikawa, R., Khan, N. C., Sasaki, S., Binns, C. W.	Risk factors for iron‐deficiency anaemia among pregnant women living in rural Vietnam	2006	Public Health Nutrition	Exclusion reason: Wrong study design
Ain, Q., Aslam, S., Mushtaq, S., Sohail, H.	A survey of knowledge and use of folic acid among women of child bearing age in rawalpindi(Pakistan)	2013	Journal of Perinatal Medicine. Conference: 11th World Congress of Perinatal Medicine	Exclusion reason: Wrong study design
Ajayi, G. O., Fadiran, E. O.	The effect of 61 days of combined iron (Chemiron) and single iron therapy on haemoglobin, packed cell volume, platelets and reticulocytes during pregnancy. Preliminary report	1998	Clinical and Experimental Obstetrics and Gynecology	Exclusion reason: Wrong study design
Akinloye, O., Oyewale, O. J., Oguntibeju, O. O.	Evaluation of trace elements in pregnant women with pre‐eclampsia	2010	African Journal of Biotechnology	Exclusion reason: Wrong outcomes
Akinwande, B. A., Ade‐Omowaye, B. I. O., Olaniyan, S. A., Akintaro, O. O.	Quality evaluation of ginger‐flavoured soy‐cassava biscuit. (Food allergies and intolerances.)	2008	Nutrition & Food Science	Exclusion reason: Wrong outcomes
Al Badawi, M., Hammoud, M. S., Molla, A. M., Shukkur, M., Thalib, L., Eliwa, M. S.	Vitamin D status of mothers and their neonates in Kuwait	2005	Pediatrics International	Exclusion reason: Wrong patient population
Alam, A., Rasheed, S., Khan, N. U. Z., Sharmin, T., Huda, T. M., Arifeen, S. E., Dibley, M. J.	How can formative research inform the design of an iron‐folic acid supplementation intervention starting in first trimester of pregnancy in Bangladesh?	2015	BMC Public Health	Exclusion reason: Wrong study design
Alam, D. S.	Maternal and child nutrition in rural Bangladesh: special reference to the effect of dietary fat supplementation on vitamin A status	2001	Maternal and child nutrition in rural Bangladesh: special reference to the effect of dietary fat supplementation on vitamin A status	Exclusion reason: Wrong study design
Alam, D. S.	Prevention of low birthweight	2009	Nestle Nutrition workshop series	Exclusion reason: Wrong study design
Alam, D. S.	Maternal and child nutrition in rural Bangladesh: special reference to effect of dietary fat supplementation on vitamin A status	2001		Exclusion reason: Wrong study design
Alfawaz, H. A., Khan, N., Al‐Oteabi, N., Hussain, S. D., Al‐Daghri, N. M.	Factors associated with dietary supplement use in Saudi pregnant women	2017	Reproductive Health	Exclusion reason: Wrong study design
Ali, A. A. A., Elgessim, M. E., Taha, E., Adam, G. K.	Factors associated with perinatal mortality in Kassala, eastern Sudan: a community‐based study 2010‐2011	2014	Journal of Tropical Pediatrics	Exclusion reason: Wrong study design
Ali, T., Khan, J.	Prevalence of vitamin D deficiency among postpartum women and their newborns: A cross‐sectional study in Karachi, Pakistan	2013	Intensive Care Medicine	Exclusion reason: Wrong intervention
Al‐Jaroudi, D., Al‐Banyan, N., Aljohani, N. J., Kaddour, O., Al‐Tannir, M.	Vitamin D deficiency among subfertile women: case‐control study	2016	Gynecol Endocrinol	Exclusion reason: Wrong intervention
Allen, L. H.	Interventions for micronutrient deficiency control in developing countries: past, present and future	2003	Journal of Nutrition	Exclusion reason: Wrong study design
Allen, L. H., Peerson, J. M.	Impact of multiple micronutrient versus iron‐folic acid supplements on maternal anaemia and micronutrient status in pregnancy. (Special Issue: Multiple micronutrient supplementation during pregnancy in developing country settings)	2009	Food and nutrition bulletin	Exclusion reason: Wrong study design
Allen, L. H., Peerson, J. M., Olney, D. K.	Provision of multiple rather than two or fewer micronutrients more effectively improves growth and other outcomes in micronutrient‐deficient children and adults	2009	Journal of Nutrition	Exclusion reason: Wrong patient population
Alramdhan, A. M., El‐Zubair, A. G.	Poor vitamin D supplementation in infants. Cross‐sectional study of maternal practices and awareness of vitamin D supplementation in infants in Al‐Ahsa, Eastern Saudi Arabia	2014	Saudi Med J	Exclusion reason: Wrong study design
Alsammani, M. A., Abdelillah, K., Adam, E. M.	Factors associated with folic acid knowledge and intake among pregnant women in Sudan	2017	Eastern Mediterranean Health Journal	Exclusion reason: Wrong study design
Alzate, A., Herrera‐Medina, R., Pineda, L. M.	Preeclampsia prevention: a case‐control study nested in a cohort	2015	Colombia Medica	Exclusion reason: Wrong study design
Amarin, Z. O., Obeidat, A. Z.	Effect of folic acid fortification on the incidence of neural tube defects	2010	Paediatric and Perinatal Epidemiology	Exclusion reason: Wrong study design
Aminee, A., Safi, S. A., Anwari, E.	Calcium supplementation among targeted pregnancies: A life saving strategy to reduce the risk of eclampsia and maternal and newborn deaths	2017	Annals of Nutrition and Metabolism	Exclusion reason: Wrong study design
Amini, E., Dalili, H., Niknafs, N., Shariat, M., Nakhostin, M., Jedari‐Attari, S.	The effect of probiotics in prevention of necrotising enterocolitis in preterm neonates in comparison with control group	2017	Iranian Journal of Pediatrics	Exclusion reason: Paediatric population
Amtuz, Z., Abdullah, S. M. S., Muhammad, S., Moinuddin.	Effect of intravenous iron supplementation on hepcidin levels in iron deficient pregnant females in second and third trimester	2017	Indian Journal of Hematology & Blood Transfusion	Exclusion reason: Wrong intervention
Amuna, P., Zotor, F. B., Adewuya, T.	Impact of locally made food multimix on maternal weight gain and outcome of pregnancy in gauteng province, South Africa	2012	Proceedings of the Nutrition Society. Conference: Summer Meeting of the Nutrition Society Hosted by the Irish Section	Exclusion reason: Wrong intervention
Anam, M., Farkhanda, R., Maham, M., Tanvir, J.	Effectiveness of vitamin C in preventing pre‐labour rupture of chorio‐amniotic membranes in pregnancy in women having history of PROM in previous pregnancies	2015	Journal of Postgraduate Medical Institute	Exclusion reason: Wrong study design
Andersen, L. T., Thilsted, S. H., Nielsen, B. B., Suguna, R.	Food and nutrient intakes among pregnant women in rural Tamil Nadu, South India	2003	Public Health Nutrition	Exclusion reason: Wrong study design
Angeles‐Agdeppa, I., Paulino, L. S., Ramos, A. C., Etorma, U. M., Cavalli‐Sforza, T., Milani, S.	Government‐industry partnership in weekly iron‐folic acid supplementation for women of reproductive age in the Philippines: impact on iron status	2005	Nutrition Reviews	Exclusion reason: Wrong outcomes
Angkasa, D., Tambunan, V., Khusun, H., Witjaksono, F, Agustina, R.	Inadequate dietary α‐linolenic acid intake among Indonesian pregnant women is associated with lower newborn weights in urban Jakarta	2017	Asia Pacific Journal of Clinical Nutrition	Exclusion reason: Wrong study design
Anonymous.	Canadian Society for Epidemiology and Biostatics 2012 National Student Conference	2012	American Journal of Epidemiology Conference	Exclusion reason: Wrong study design
Anonymous.	2nd International Conference on Clinical Neonatology	2011	Early Human Development. Conference: 2nd International Conference on Clinical Neonatology. Torino Italy. Conference Publication.	Exclusion reason: Wrong study design
Anonymous.	Vitamin supplementation in pregnancy	2016	Drug & Therapeutics Bulletin	Exclusion reason: Wrong study design
Anonymous.	Abstracts of the XXII European Congress of Perinatal Medicine, 2010	2010	Journal of Maternal Fetal and Neonatal Medicine. Conference: 22nd European Congress of Perinatal Medicine	Exclusion reason: Wrong study design
Anonymous.	Abstracts of the RCOG World Congress 2014	2014	BJOG: An International Journal of Obstetrics and Gynaecology. Conference: RCOG World Congress	Exclusion reason: Wrong study design
Antal, M., Griffiths, M., Schaetzel, T.	Critical pathways affecting maternal anaemia: A road map for integrated programming for nutrition, health and agriculture	2013	Annals of Nutrition and Metabolism	Exclusion reason: Wrong intervention
Antartani, R., Ashok, K.	Effect of lycopene in prevention of preeclampsia in high risk pregnant women. [Turkish]	2011	Journal of the Turkish German Gynecology Association	Exclusion reason: Wrong intervention
Anwaar, Ahmed, Asif, Ahmad, Khalid, N., David, A., Sandhu, M. A., Randhawa, M. A., Suleria, H. A. R.	A question mark on iron deficiency in 185 million people of Pakistan: its outcomes and prevention	2014	Critical Reviews in Food Science and Nutrition	Exclusion reason: Wrong study design

**Table 6:** Characteristics table of excluded studies (Database Searches)

Authors	Title	Published Year	Journal	Exclusion Reason
Anwar, F, Mudjajanto, E. S., Martianto, D., Hakimi.	High protein and iron‐folate crackers supplementation on the iron status of pregnant women	2003	Medical Journal of Indonesia	Exclusion reason: Wrong intervention
Anwar, U. S.	Effects of a single dose of oral iodised oil during pregnancy, on thyroid & iodide status of women & children, pregnancy outcome and infant mortality in a goitrous area of Bangladesh	2001		Exclusion reason: Wrong intervention
Arguello, M. A., Schulze, K. J., Wu, L. S. F., Dreyfuss, M. L., Christian, P., West, K. P.	Circulating insulin‐like growth Factor‐1 (IGF‐1) is associated with vitamin A (VA) status and increased circulating hemoglobin (Hb) during pregnancy in Nepalese Women	2012	FASEB Journal. Conference: Experimental Biology	Exclusion reason: Wrong intervention
Arimond, M., Zeilani, M., Jungjohann, S., Brown, K. H., Ashorn, P., Allen, L. H., Dewey, K. G.	Considerations in developing lipid‐based nutrient supplements for prevention of undernutrition: experience from the International Lipid‐Based Nutrient Supplements (iLiNS) Project	2015	Maternal & Child Nutrition	Exclusion reason: Wrong study design
Ariyo, O., Keshinro, O. O.	Serum ascorbic acid levels in the third trimester of pregnancy and ascorbate content of maternal breast milk	2012	Continental Journal of Medical Research	Exclusion reason: Wrong outcomes
Arora, C. P., Kacerovsky, M., Zinner, B., Ceausu, I., Lancz, K., Shurpyak, S., Sandhu, S. S., Hobel, C. J., Vari, S. G.	Iron supplementation during pregnancy: Friend or foe	2013	Reproductive Sciences	Exclusion reason: Wrong study design
Arora, C. P., Kacerovsky, M., Zinner, B., Ceausu, I., Lancz, K., Shurpyak, S., Sandhu, S. S., Hobel, C. J., Vari, S. G.	Maternal excess iron intake during pregnancy increases oxidative stress and affects birth outcome	2013	Biopolymers and Cell	Exclusion reason: Wrong study design
Arriaga, C. L., Fuentes, D., Diaz‐Ruiz, E., Garcia‐Meza, R., Solomons, N. W., Hirst, D., Bonorden, M.	Acceptability of fortified whey protein concentrate supplements when added to customary thin gruels (atoles) by women in the western highlands of Guatemala	2017	Annals of Nutrition and Metabolism	Exclusion reason: Wrong outcomes
Aşcı, Özlem; Rathfisch, G.	Effect of lifestyle interventions of pregnant women on their dietary habits, lifestyle behaviors, and weight gain: a randomized controlled trial	2016	Journal of Health, Population and Nutrition	Exclusion reason: Wrong intervention
Asemi, Z., Samimi, M., Tabassi, Z., Naghibi Rad, M., Rahimi Foroushani, A., Khorammian, H., Esmaillzadeh, A.	Effect of daily consumption of probiotic yoghurt on insulin resistance in pregnant women: a randomized controlled trial	2013	European Journal of Clinical Nutrition	Exclusion reason: Wrong intervention
Asemi, Z, Hashemi, T, Karamali, M, Samimi, M, Esmaillzadeh, A.	Effects of vitamin D supplementation on glucose metabolism, lipid concentrations, inflammation, and oxidative stress in gestational diabetes: a double‐blind randomized controlled clinical trial	2013	American Journal of Clinical Nutrition	Exclusion reason: Wrong patient population
Assegedech, B., Yared, W., Yared, A., Dieter, R., Getahun, A.	Conjunctival impression cytology and detection of vitamin A deficiency in pregnant women, Gondar, Northwest Ethiopia	2012	Ethiopian Medical Journal	Exclusion reason: Wrong study design
Atalah S, E., Araya B, M., Rosselot P, G., Araya L, H., Vera A, G., Andreu R, R., Barba G, C., Rodriguez, L.	Consumption of a DHA‐enriched milk drink by pregnant and lactating women, on the fatty acid composition of red blood cells, breast milk, and in the newborn	2009	Archivos Latinoamericanos de Nutricion	Exclusion reason: Wrong intervention
Atalhi, N., Choua, G., Elhamdouchi, A., Elhaloui, N., Elmzibri, M., Haskell, M., Aguenaou, H., Mokhtar, N.	Impact of daily consumption of Vitamin A fortified oil on human milk Vitamin A concentration in lactating Moroccan women	2011	Annals of Nutrition and Metabolism	Exclusion reason: Wrong intervention
Ates, S., Sevket, O., Ozcan, P., Ozkal, F., Kaya, M. O., Dane, B.	Vitamin D status in the first‐trimester: effects of vitamin D deficiency on pregnancy outcomes	2016	African Health Sciences	Exclusion reason: Wrong outcomes
Athiyarath, R., Shaktivel, K., Singh, D., Abraham, V. J., Srivastava, A., Edison, E. S.	Association of single nucleotide polymorphisms with TIBC and erythrocyte count in iron deficiency anaemia of pregnancy in Indian population	2013	American Journal of Hematology	Exclusion reason: Wrong outcomes
Atwell, K., Dresang, L.	Does vitamin A or beta‐carotene supplementation during pregnancy reduce maternal mortality in developing countries where vitamin A deficiency is endemic?	2014	Evidence‐Based Practice	Exclusion reason: Wrong study design
Avula, R., Kim, S., Kohli, N., Chakrabarti, S., Tyagi, P., Singh, K., Van Den Bold, M., Kadiyala, S., Menon, P.	At‐scale delivery of essential nutrition interventions (ENIs) in India is limited by variable gaps in policy, design, implementation and demand	2015	FASEB Journal. Conference: Experimental Biology	Exclusion reason: Wrong patient population
Ayah, R. A. L.	High dose maternal and infant vitamin A supplementation in Bondo District, Kenya: its effects on vitamin A and iron status. (BL: DXN069078)	2002		Exclusion reason: Wrong patient population
Ayoya, M. A., Heidkamp, R., Ngnie‐Teta, I., Mamadoultaibou, A., Daniel, E. F., Durandisse, E. B., Saint‐Fleur, J. E., Beauliere, J. M., Koita, Y., M'Mbakwa, B. E., Stoltzfus, R. J., Pierre, J. M.	Precis of nutrition of children and women in Haiti: Analyses of data from 1995 to 2012	2014	Annals of the New York Academy of Sciences	Exclusion reason: Wrong study design
Babayan, S, Aslanyan, G, Amroyan, E, Gabrielyan, E, Wikman, G,Panossian, A. G.	Comparative study of femineral and floradix in women of child‐bearing age and adolescent girls with iron deficiency anaemia	2008	Scientia pharmaceutica	Exclusion reason: Wrong patient population
Babu, T. A., Sharmila, V.	Vitamin A supplementation in late pregnancy can decrease the incidence of bronchopulmonary dysplasia in newborns	2010	Journal of Maternal‐Fetal & Neonatal Medicine	Exclusion reason: Paediatric population
Bagheri, A., Eskandari, N., Abbaszadeh, F.	Self‐medication and supplement use by pregnant women in kashan rural and urban areas	2014	Journal of Mazandaran University of Medical Sciences	Exclusion reason: Wrong study design
Bahizire, E., D'Alessandro, U., Dramaix, M., Dauby, N., Bahizire, F., Mubagwa, K., Donnen, P.	Malaria and iron load at the first antenatal visit in the rural south kivu, democratic republic of the congo: Is iron supplementation safe or could it be harmful?	2018	American Journal of Tropical Medicine and Hygiene	Exclusion reason: Wrong patient population
Balarajan, Y., Subramanian, S. V., Fawzi, W. W.	Maternal iron and folic acid supplementation is associated with lower risk of low birth weight in India	2013	Journal of Nutrition	Exclusion reason: Wrong intervention
Balogun, O. O, da Silva Lopes, K., Ota, E., Takemoto, Y., Rumbold, A., Takegata, M., Mori, R.	Vitamin supplementation for preventing miscarriage	2016	Cochrane Database of Systematic Reviews	Exclusion reason: Wrong study design
Banjari, I., Kenjeric, D., Mandic, M. L., Koceva Komlenic, D., Jukic, M., Ugarcic, Z., Planinic, M., Hasenay, S.	Cereals and their products as source of energy and nutrients in early pregnancy	2012		Exclusion reason: Wrong intervention
Barbosa, L., Ribeiro, D. de Q., de Faria, F. C., Nobre, L. N., Lessa Ado, C.	Factors associated with folic acid use during pregnancy (Article in Portuguese)	2011	Revista Brasileira de Ginecologia e Obstetricia	Exclusion reason: Wrong intervention
Barnabé, A., Aléssio, A. C. M., Bittar, L. F., de Moraes Mazetto, B., Bicudo, A. M., de Paula, E. V., Höehr, N. F., Annichino‐Bizzacchi, J. M.	Folate, vitamin B12 and Homocysteine status in the post‐folic acid fortification era in different subgroups of the Brazilian population attended to at a public health care center	2015	Nutrition Jornal	Exclusion reason: Wrong intervention
Bath, S. C.	Iodine supplementation in pregnancy in mildly deficient regions	2017	The Lancet Diabetes and Endocrinology	Exclusion reason: Wrong study design
Baxter, J. A. B., Roth, D. E., Abdullah, A.‐M., Tahmeed, A., Munirul, I., Zlotkin, S. H.	Tablets are preferred and more acceptable than powdered prenatal calcium supplements among pregnant women in Dhaka, Bangladesh	2014	Journal of Nutrition	Exclusion reason: Wrong outcomes
Baxter, J. A., Roth, D., Mahmud, A., Islam, M., Ahmed, T., Zlotkin, S.	Preference and acceptability of alternative delivery vehicles for prenatal calcium supplementation among pregnant women in urban Bangladesh	2014	FASEB Journal. Conference: Experimental Biology	Exclusion reason: Wrong intervention
Baxter, Jo‐Anna B., Wasan, Y., Soofi, S. B., Suhag, Z., Bhutta, Z, A.	Effect of life skills building education and micronutrient supplements provided from preconception versus the standard of care on low birth weight births among adolescent and young Pakistani women (15‐24 years): a prospective, population‐based cluster‐randomized trial	2018	Reproductive Health	Exclusion reason: Wrong study design
Beard, J. L.	Effectiveness and strategies of iron supplementation during pregnancy	2000	American Journal of Clinical Nutrition	Exclusion reason: Wrong study design
Begum, K., Ouédraogo, C. T., Wessells, K. R. Young, R. R., Faye, M. T., Wuehler, S. E., Hess, S. Y.	Prevalence of and factors associated with antenatal care seeking and adherence to recommended iron‐folic acid supplementation among pregnant women in Zinder, Niger	2018	Maternal & Child Nutrition	Exclusion reason: Wrong intervention
Beketova, N. A., Sokolnikov, A. A., Kodentsova, V. M., Pereverzeva, O. G., Vrzhesinskaya, O. A., Kosheleva, O. V., Gmoshinskaya, M. V.	The vitamin status of pregnant women in Moscow: effect of multivitamin‐mineral supplements	2016	Voprosy Pitaniya	Exclusion reason: Wrong intervention
Belgnaoui, S., Belahsen, R.	Anaemia and Iron Deficiency Anaemia during pregnancy in an agricultural region of Morocco: effects of dietary intake and iron supplementation	2007	Research Journal of Biological Sciences	Exclusion reason: Wrong intervention
Ben Natan, M., Brandin Rimkus, A., Tseytlin Eryomine, A.	Factors associated with intention of Israeli‐born women and immigrant women from the Former Soviet Union to take folic acid before and during pregnancy	2018	International Journal of Nursing Practice	Exclusion reason: Wrong intervention
Berger, J., Dillon, J. C.	Control of iron deficiency in developing countries (Article in French)	2002	Sante	Exclusion reason: Wrong study design
Berger, J., Thanh, H. T. K., Cavalli‐Sforza, T., Smitasiri, S., Khan, N. C., Milani, S., Hoa, P. T., Quang, N. D., Viteri, F.	Community mobilization and social marketing to promote weekly iron‐folic acid supplementation in women of reproductive age in Vietnam: impact on anaemia and iron status…Preventive weekly iron‐folic acid supplementation can improve iron status of reproductive age women: experience in Cambodia, the Philippines, and Vietnam. World Health Organization Western Pacific Region	2005	Nutrition Reviews	Exclusion reason: Wrong study design
Berry, R. J., Bailey, L., Mulinare, J., Bower, C., Folic Acid Working Group.	Fortification of flour with folic acid	2010	Food & Nutrition Bulletin	Exclusion reason: Wrong study design
Berry, R. J., Li, Z., Erickson, J. D., Li, S., Moore, C. A., Hong, W., Mulinare, J., Ping, Z., Wong, L. Y. C., Gindler, J., Hong, S. Correa, A.	Prevention of neural‐tube defects with folic acid in China	1999	New England Journal of Medicine	Exclusion reason: Wrong study design
Berti, C., Gaffey, M. F., Bhutta, Z. A.,; Cetin, I.	Multiple‐micronutrient supplementation: Evidence from large‐scale prenatal programmes on coverage, compliance and impact	2017	Maternal and Child Nutrition	Exclusion reason: Wrong study design
Berti, P. R., Mildon, A., Siekmans, K., Main, B., Macdonald, C.	An adequacy evaluation of a 10‐year, four‐country nutrition and health programme	2010	International Journal of Epidemiology	Exclusion reason: Wrong outcomes
Bertinato, J., Paniz, C., Lucena, M. R., Da Silva Amorim, P. M., Gomes, G. W., Figueiredo, M. S., Green, R., Shinohara, E. M. G.	High dose (5 mg) daily folic acid supplement in healthy brazilian volunteers increases mononuclear TNF‐alpha expression and reduces NK cell number and activity	2015	Blood	Exclusion reason: Wrong patient population
Bhan, M. K., Sommerfelt, H., Black, R. E., Sandstrom, B., Bhandari, N., Bahl, R.	Micronutrients, maternal and child health. Indo‐European symposium on micronutrients, maternal and child health, Goa, India, 25‐27 April 1999	2001	British Journal of Nutrition	Exclusion reason: Wrong study design
Bharati, K., Christian, P., LeClerq, S. C., Khatry, S. K.	Determinants of compliance to antenatal micronutrient supplementation and women's perceptions of supplement use in rural Nepal	2010	Public Health Nutrition	Exclusion reason: Wrong outcomes
Bhattiprolu, S., Brahmam, G. N. V., Nair, K. M., Ranganathan, S., Rao, M. V., Vijayaraghavan, K., Kamala, K.	Prospects of fortification of salt with iron and iodine. (Micronutrients, maternal and child health)	2001	British Journal of Nutrition	Exclusion reason: Wrong study design
Bhutta, Z. A., Imdad, A., Ramakrishnan, U., Martorell, R.	Is it time to replace iron folate supplements in pregnancy with multiple micronutrients?	2012	Paediatric and Perinatal Epidemiology	Exclusion reason: Wrong study design
Biesalski, H. K., Lambert, C.	Vitamin A deficiency ‐ An overlooked problem? [German]	2014	Gynakologe	Exclusion reason: Wrong study design
Bisanz, J. E., Enos, M. K., PrayGod, G., Seney, S., Macklaim, J. M., Chilton, S., Willner, D., Knight, R., Fusch, C., Fusch, G., Gloor, G. B., Burton, J. P., Reid, G.	Microbiota at multiple body sites during pregnancy in a rural Tanzanian population and effects of Moringa‐supplemented probiotic yogurt	2015	Applied and Environmental Microbiology	Exclusion reason: Wrong intervention
Bisratemariam, G., Abel Fekadu, D., Azeb, A.	High adherence to iron/folic acid supplementation during pregnancy time among antenatal and postnatal care attendant mothers in governmental health centers in Akaki kality sub city, Addis Ababa, Ethiopia: hierarchical negative binomial poisson regression	2017	PLoS ONE [Electronic Resource]	Exclusion reason: Wrong intervention
Bitam, A., Belkadi, N.	Prevalence of iron deficiency anaemia during pregnancy in Blida (north of Algeria). [French]	2008	Nutrition Clinique et Metabolisme	Exclusion reason: Wrong outcomes
Black, M. M.	Integrated strategies needed to prevent iron deficiency and to promote early child development	2012	Journal of Trace Elements in Medicine and Biology	Exclusion reason: Wrong study design
Black, R. E.	Zinc deficiency, immune function, and morbidity and mortality from infectious disease among children in developing countries	2001	Food and Nutrition Bulletin	Exclusion reason: Paediatric population
Black, R. E.	Micronutrients in pregnancy. (Micronutrients, maternal and child health)	2001	British Journal of Nutrition	Exclusion reason: Wrong study design
Bond, T. A., Joglekar, C. V., Marley‐Zagar, E., Lubree, H. G., Kumaran, K., Yajnik, C. S., Fall, C. H. D.	Maternal vitamin B12 and folate status during pregnancy and insulin resistance and body composition in the offspring at 12 years in a rural Indian birth cohort: Data from the Pune Maternal Nutrition Study	2013	Proceedings of the Nutrition Society	Exclusion reason: Wrong intervention
Bond, T., Joglekar, C., Marley‐Zagar, E., Lubree, H., Kumaran, K., Yajnik, C., Fall, C.	Maternal vitamin B12 and folate status during pregnancy and offspring insulin resistance and body composition at 12 years	2013	European Journal of Epidemiology	Exclusion reason: Wrong intervention
Bondevik, G. T., Eskeland, B., Ulvik, R. J., Ulstein, M., Lie, R. T., Schneede, J., Kvale, G.	Anaemia in pregnancy: possible causes and risk factors in Nepali women	2000	European Journal of Clinical Nutrition	Exclusion reason: Wrong interventiom
Bonvecchio, A., Pelto, G. H., Escalante, E., Monterrubio, E., Habicht, J. P., Nava, F., Villanueva, M. A., Safdie, M., Rivera, J. A.	Maternal knowledge and use of a micronutrient supplement was improved with a programmatically feasible intervention in Mexico	2007	Journal of Nutrition	Exclusion reason: Wrong intervention
Bopape, M. M., Mbhenyane, X. G., Alberts, M.	The prevalence of anaemia and selected micronutrient status in pregnant teenagers of Polokwane Municipality in the Limpopo Province	2008	SAJCN South African Journal of Clinical Nutrition	Exclusion reason: Wrong intervention
Borras, B. E., Martinez, C., N., Capdevila, B. S., Cortesao, T. V., Roig, G. M. C.	Micronutrient supplementation in preconceptional period and the pregnancy associated with low birth weight	2014	Journal of Maternal‐Fetal and Neonatal Medicine	Exclusion reason: Wrong study design
Bortolozo, E. F. Q., Candido, L. M. B., Colombo, A. O., dos Santos Junior, G.	Protein supplementation effects on human milk protein and A immunoglobin concentrations	2010	Revista do Instituto Adolfo Lutz	Exclusion reason: Wrong patient population; No MNS supplmentation; Wrong population
Botto, L. D., Mulinare, J., Yang, Q. H., Liu, Y. C., Erickson, J. D.	Autosomal trisomy and maternal use of multivitamin supplements	2004	American Journal of Medical Genetics Part A	Exclusion reason: Wrong intervention
Botto, L. D., Olney, R. S., Erickson, J. D.	Vitamin supplements and the risk for congenital anomalies other than neural tube defects	2004	American Journal of Medical Genetics. Part C, Seminars in Medical Genetics	Exclusion reason: Wrong study design
Brancatisano, S., Dibley, M. J.	Prenatal micronutrient and early pregnancy food supplementation in Bangladesh	2012	JAMA	Exclusion reason: Wrong study design
Briend, A.	Highly nutrient‐dense spreads: a new approach to delivering multiple micronutrients to high‐risk groups	2001	British Journal of Nutrition	Exclusion reason: Paediatric population
Britto, J. C., Cancado, R., Guerra‐Shinohara, E. M.	Concentrations of blood folate in Brazilian studies prior to and after fortification of wheat and cornmeal (maize flour) with folic acid: A review	2014	Revista Brasileira de Hematologia e Hemoterapia	Exclusion reason: Wrong intervention
Brough, L., Rees, G. A., Crawford, M. A.	Thiamin status during pregnancy and pregnancy outcome. (Forty Second Annual Conference)	2007	Proceedings of the Nutrition Society of New Zealand	Exclusion reason: Wrong patient population
Brough, L., Rees, G. A., Crawford, M. A., Morton, R. H., Dorman, E. K.	Effect of multiple‐micronutrient supplementation on maternal nutrient status, infant birth weight and gestational age at birth in a low‐income, multi‐ethnic population	2010	British Journal of Nutrition	Exclusion reason: Wrong patient population
Brouwer, I. D., Melse‐Boonstra, A., Mouquet‐Rivier, C., Hurrell, R., Kvalsvig, J., Houhouigan, J., Mwangi, A., Weltzien, E., Diawara, B., Maziya‐Dixon, B., Boy, E., Verhoef, H.	Instapa: Identifying innovative staple food based strategies to alleviate micronutrient deficiencies for health and development of women and children in africa	2013	Annals of Nutrition and Metabolism	Exclusion reason: Wrong intervention
Busby, A., Abramsky, L., Dolk, H., Armstrong, B.	Preventing neural tube defects in Europe: population based study	2005	British Medical Journal	Exclusion reason: Wrong intervention
Butt, M. S., Tahir‐Nadeem, M., Shahid, M.	Vitamin A: deficiency and food‐based combating strategies in Pakistan and other developing countries	2007	Food Reviews International	Exclusion reason: Wrong study design
Buyukuslu, N., Oval, S., Altuntas, S. L., Batrel, S., Yigit, P., Garipagaoglu, M.	Supplementation of docosahexaenoic acid (DHA)/Eicosapentaenoic acid (EPA) in a ratio of 1/1.3 during the last trimester of pregnancy results in EPA accumulation in cord blood	2017	Prostaglandins, Leukotrienes and Essential Fatty Acids	Exclusion reason: Wrong outcomes
Calvo, E. B., Biglieri, A.	[Impact of folic acid fortification on women's nutritional status and on the prevalence of neural tube defects] (Article in Spanish)	2008	Archivos Argentinos de Pediatria	Exclusion reason: Wrong intervention
Cao, H., Wei, X., Guo, X., Song, C., Luo, Y., Cui, Y., Hu, X., Zhang, Y.	Screening high‐risk clusters for developing birth defects in mothers in Shanxi Province, China: application of latent class cluster analysis	2015	BMC Pregnancy Childbirth	Exclusion reason: Wrong intervention
Carrara, V. I., Stuetz, W., Lee, S. J., Sriprawat, K., Po, B., Hanboonkunupakarn, B., Nosten, F. H., McGready, R.	Longer exposure to a new refugee food ration is associated with reduced prevalence of small for gestational age: results from 2 cross‐sectional surveys on the Thailand‐Myanmar border	2017	American Journal of Clinical Nutrition	Exclusion reason: Wrong patient population
Casanueva, E., Ripoll, C., Tolentino, M., Morales, R. M., Pfeffer, F., Vilchis, P., Vadillo‐Ortega, F.	Vitamin C supplementation to prevent premature rupture of the chorioamniotic membranes: a randomized trial	2005	American Journal of Clinical Nutrition	Exclusion reason: Wrong intervention
Caulfield, L. E., Donangelo, C. M., Chen, P., Junco, J., Merialdi, M., Zavaleta, N.	Red blood cell metallothionein as an indicator of zinc status during pregnancy	2008	Nutrition	Exclusion reason: Wrong comparator
Caulfield, L. E., Zavaleta, N., Chen, P., Lazarte, F., Albornoz, C., Putnick, D. L., Bornstein, M. H., DiPietro, J. A.	Maternal zinc supplementation during pregnancy affects autonomic function of Peruvian children assessed at 54 months of age	2011	Journal of Nutrition	Exclusion reason: Wrong comparator
Cavalli‐Sforza, T.	Effectiveness of weekly iron‐folic acid supplementation to prevent and control anaemia among women of reproductive age in three Asian countries: development of the master protocol and implementation plan	2005	Nutrition Reviews	Exclusion reason: Wrong study design
Cavalli‐Sforza, T., Berger, J., Smitasiri, S., Viteri, F.	Summary. Weekly iron‐folic acid supplementation of women of reproductive age: impact overview, lessons learned, expansion plans, and contributions toward achievement of the millennium development goals…Preventive weekly iron‐folic acid supplementation can improve iron status of reproductive age women: experience in Cambodia, the Philippines, and Vietnam. World Health Organization Western Pacific Region [corrected] [published erratum appears in NUTR REV 2006 Feb;64(2 Part 1):92]	2005	Nutrition Reviews	Exclusion reason: Wrong study design
Cawley, S., Mullaney, L., McKeating, A., Farren, M., McCartney, D., Turner, M. J.	A review of European guidelines on periconceptional folic acid supplementation	2016	European Journal of Clinical Nutrition	Exclusion reason: Wrong intervention
Cefalo, R. C.	[Commentary on] Calcium supplements and bone resorption in pregnancy: a randomized crossover trial	2003	Obstetrical & Gynecological Survey	Exclusion reason: Wrong study design
Cesar, J. A., Dumith, S. de C., Chrestani, M. A. D., Mendoza‐Sassi, R. A.	Iron supplementation among pregnant women: results from a population‐based survey study	2013	Revista Brasileira de Epidemiologia	Exclusion reason: Wrong intervention
Chagas, C., Saunders, C., Pereira, S., Silva, J., Saboya, C., Ramalho, A.	Vitamin A status and its relationship with serum zinc concentrations among pregnant women who have previously undergone Roux‐en‐Y gastric bypass	2016	International Journal of Gynecology & Obstetrics	Exclusion reason: Wrong patient population
Chakhtoura, M., El‐Ghandour, S., Shawwa, K., Akl, E. A., Arabi, A., Mahfoud, Z., Habib, R., Hoballah, H., Fuleihan, G. E.	Vitamin D replacement in children, adolescents and pregnant women in the Middle East and North Africa: a systematic review and meta‐analysis of randomized controlled trials	2017	Metabolism, Clinical and Experimental	Exclusion reason: Wrong study design
Chakhtoura, M., Rahme, M., Chamoun, N., El‐Hajj Fuleihan, G.	Vitamin D in the Middle East and North Africa	2018	Bone Reports	Exclusion reason: Wrong study design
Chandyo, R. K., Ulak, M., Kvestad, I., Shrestha, M., Ranjitkar, S., Basnet, S., Hysing, M., Shrestha, L., Strand, T. A.	The effects of vitamin B12 supplementation in pregnancy and postpartum on growth and neurodevelopment in early childhood: study protocol for a Randomized Placebo Controlled Trial	2017	BMJ Open	Exclusion reason: Wrong intervention
Changamire, F. T., Mwiru, R. S., Peterson, K. E., Msamanga, G. I., Spiegelman, D., Petraro, P., Urassa, W., Fawzi, W. W.	Effect of multivitamin supplements on weight gain during pregnancy among HIV‐negative women in Tanzania	2015	Maternal and Child Nutrition	Exclusion reason: Wrong outcomes
Charatcharoenwitthaya, N., Nanthakomon, T., Somprasit, C., Chanthasenanont, A., Chailurkit, L., Ongphiphadhanakul, B.	Maternal vitamin D status during the course of pregnancy in thai women	2012	Endocrine Reviews. Conference: 94th Annual Meeting and Expo of the Endocrine Society, ENDO	Exclusion reason: Wrong intervention
Charatcharoenwitthaya, N., Nanthakomon, T., Somprasit, C., Chanthasenanont, A., Chailurkit, L., Pattaraarchachai, J., Ongphiphadhanakul, B.	Maternal vitamin D status, its associated factors and the course of pregnancy in Thai women	2013	Clinical Endocrinology	Exclusion reason: Wrong intervention
Charles, C. V., Dewey, C. E., Daniell, W. E., Summerlee, A. J. S.	Iron‐deficiency anaemia in rural cambodia: community trial of a novel iron supplementation technique	2011	European Journal of Public Health	Exclusion reason: Wrong patient population
Charles, C. V., Summerlee, A. J., Dewey, C. E.	Anemia in Cambodia: prevalence, etiology and research needs	2012	Asia Pacific Journal of Clinical Nutrition	Exclusion reason: Wrong study design
Chaudhary, A. K., Asha, C., Tiwari, S. C., Dwivedi, R.	Can community‐based, low‐cost antenatal care in the third trimester of pregnancy reduce the incidence of low birth weight newborns?	2012	Journal of Obstetrics and Gynecology of India	Exclusion reason: Wrong outcomes
Cheatham, C. L., Goldman, B. D., Fischer, L. M., Costa, K. A. da, Reznick, J. S., Zeisel, S. H.	Phosphatidylcholine supplementation in pregnant women consuming moderate‐choline diets does not enhance infant cognitive function: a randomized, double‐blind, placebo‐controlled trial	2012	American Journal of Clinical Nutrition	Exclusion reason: Wrong intervention
Chen, X., Jin, B., Xia, J., Tao, X., Huang, X., Sun, L., Yuan, Q.	Effects of Thyroid Peroxidase Antibody on Maternal and Neonatal Outcomes in Pregnant Women in an Iodine‐Sufficient Area in China	2016	International Journal of Endocrinology	Exclusion reason: Wrong intervention
Cheng, T. L., Mistry, K. B., Wang, G., Zuckerman, B., Wang, X.	Folate Nutrition Status in Mothers of the Boston Birth Cohort, Sample of a US Urban Low‐Income Population	2018	American Journal of Public Health	Exclusion reason: Wrong patient population
Cheng, Y., Dibley, M. J., Zhang, X., Zeng, L., Yan, H.	Assessment of dietary intake among pregnant women in a rural area of western China	2009	BMC public health	Exclusion reason: Wrong outcomes
Cheong, M., Xiao, H., Tay, V., Karakochuk, C. D., Liu, Y, Harvey, S., Lamers, Y., Houghton, L. A., Kitts, D. D., Green, T. J.	Folic acid fortified milk increases blood folate to concentrations associated with a very low risk of neural tube defects in Singaporean women of childbearing age	2016	Asia Pacific Journal of Clinical Nutrition	Exclusion reason: Wrong patient population

**Table 7:** Characteristics table of excluded studies (Database Searches)

Authors	Title	Published Year	Journal	Exclusion Reason
ChiCTR‐EOC‐15007525, *National Institute for Nutrition and Health*, Chinese Center for Disease Control and Prevention	Metabolic balance study on urine iodine thresold of Chinese pregnant women which sustain the maternal and neonatal normal thyroid function	2015		Exclusion reason: Wrong patient population
ChiCTR‐TRC‐13003805, First Affiliated Hospital of China Medical University	Screening and Intervention for iodine deficiency, iron deficiency and subclinical thyroid insufficiency in women planning pregnancy and in early pregnant women	2013		Exclusion reason: Wrong intervention
Chima, I., Adebisi, B., Okpara, A., Mukoro, P.	Effective communication channels for community mobilization during vitamin a supplementation campaigns in 3 states of Nigeria	2013	Annals of Nutrition and Metabolism	Exclusion reason: Wrong study design
Chitayat, D., Matsui, D., Amitai, Y., Kennedy, D., Sunita, Vohra, Rieder, M., Koren, G.	Folic acid supplementation for pregnant women and those planning pregnancy: 2015 update	2016	Journal of Clinical Pharmacology	Exclusion reason: Wrong study design
Chomat, A. M., Crowley, C., Montenegro‐Bethancourt, G., Solomons, N., Bermudez, O.	Feeding practices during pregnancy and early lactation among Mam‐speaking women in the western highlands of Quetzaltenango, Guatemala	2011	FASEB Journal. Conference: Experimental Biology	Exclusion reason: Wrong study design
Chong, M. F.‐F., Chia, A.‐R., Colega, M., Tint, M.‐T., Aris, I. M., Chong, Y.‐S., Gluckman, P., Godfrey, K. M., Kwek, K., Saw, S.‐M., Yap, F., van Dam, R. M., Lee, Y. S.	Maternal Protein Intake during Pregnancy Is Not Associated with Offspring Birth Weight in a Multiethnic Asian Population	2015	The Journal of Nutrition	Exclusion reason: Wrong intervention
Christian, A. M., Krishnaveni, G. V., Kehoe, S. H., Veena, S. R., Khanum, R., Marley‐Zagar, E., Edwards, P., Margetts, B. M., Fall, C. H.	Contribution of food sources to the vitamin B12 status of South Indian children from a birth cohort recruited in the city of Mysore	2015	Public Health Nutrition	Exclusion reason: Wrong intervention
Christian, P., Delange, F. M., West, K. P., Jr.	Effects of maternal micronutrient supplementation on newborn size and infant health and survival. (Nestle Nutrition Workshop Series, Pediatric Program, Vol.52)	2003		Exclusion reason: Wrong study design
Christian, P., Khatry, S. K., LeClerq, S. C., Roess, A. A., Wu, L., Yuenger, J. D., Zenilman, J. M.	Prevalence and risk factors of chlamydia and gonorrhea among rural Nepali women	2005	Sexually Transmitted Infections	Exclusion reason: Wrong intervention
Christian, P., Khatry, S. K., Yamini, S., Stallings, R., LeClerq, S. C., Shrestha, S. R., Pradhan, E. K., West, K. P., Jr.	Zinc supplementation might potentiate the effect of vitamin A in restoring night vision in pregnant Nepalese women	2001	American Journal of Clinical Nutrition	Exclusion reason: Wrong patient population
Christian, P., Labrique, A. B., Hasmot, A., Richman, M. J., Wu, L., Mahbubur, R., West, K. P., Jr.	Maternal vitamin A and beta ‐carotene supplementation and risk of bacterial vaginosis: a randomized controlled trial in rural Bangladesh	2011	American Journal of Clinical Nutrition	Exclusion reason: Wrong outcomes
Christian, P., Lammi‐Keefe, C. J., Couch, S. C.; Philipson, E. H.	Nutrition and maternal survival in developing countries. (Nutrition and Health)	2008		Exclusion reason: Wrong study design
Christian, P., Osrin, D., Manandhar, D. S., Khatry, S. K., De, Costello, A. M. de l., West Jr, K. P.	Antenatal micronutrient supplements in Nepal	2005	Lancet	Exclusion reason: Wrong study design
Christian, P., Tielsch, J. M.	Evidence for multiple micronutrient effects based on randomized controlled trials and meta‐analyses in developing countries	2012	Journal of Nutrition	Exclusion reason: Wrong study design
Christian, P., West Jr, K. P.	Nutrition: Vitamin A supplementation‐maternal and neonatal survival	2011	Nature Reviews Endocrinology	Exclusion reason: Wrong study design
Christian, P., West Jr, K. P., Katz, J., Kimbrough‐Pradhan, E., LeClerq, S. C., Khatry, S. K., Shrestha, S. R.	Cigarette smoking during pregnancy in rural Nepal. Risk factors and effects of beta ‐carotene and vitamin A supplementation	2004	European Journal of Clinical Nutrition	Exclusion reason: Wrong intervention
Clarke, R., Bennett, D.	Folate and prevention of neural tube defects	2014	BMJ: British Medical Journal	Exclusion reason: Wrong study design
Clemmons, L., Schaetzel, T., Edwin, T., Antal, M., Griffiths, M.	"Agri‐frying" the critical pathways to maternal anaemia in tanzania: Bringing agriculture into the conceptual framework	2013	Annals of Nutrition and Metabolism	Exclusion reason: Wrong intervention
Cobayashi, F., Augusto, R. A., Lourenço, B. H., Muniz, P. T., Cardoso, M. A.	Factors associated with stunting and overweight in Amazonian children: a population‐based, cross‐sectional study	2014	Public Health Nutr	Exclusion reason: Wrong intervention
Cobra, C., Muhilal, Rusmil, K., Rustama, D., Djatnika, Suwardi, S. S., Permaesih, D., Muherdiyantiningsih, Martuti, S., Semba, R. D.	Infant survival is improved by oral iodine supplementation	1997	Journal of Nutrition	Exclusion reason: Wrong patient population
Coelho, N. de L. P., Cunha, D. B., Esteves, A. P. P., Lacerda, E. M. de A., Theme Filha, M. M.	Dietary patterns in pregnancy and birth weight	2015	Rev Saude Publica	Exclusion reason: Wrong intervention
Collin, S. M., Baggaley, R. F., Pittrof, R., Filippi, V.	Could a simple antenatal package combining micronutritional supplementation with presumptive treatment of infection prevent maternal deaths in sub‐Saharan Africa?	2007	BMC Pregnancy and Childbirth	Exclusion reason: Wrong study design
Compaore, A., Gies, S., Brabin, B. J., Tinto, H., Brabin, L.	A qualitative study of the acceptability of weekly iron supplementation prior to the first pregnancy in Burkina Faso	2017	Tropical Medicine and International Health	Exclusion reason: Wrong patient population
Conlisk, A. J., Barnhart, H. X., Martorell, R., Grajeda, R., Stein, A. D.	Maternal and child nutritional supplementation are inversely associated with fasting plasma glucose concentration in young Guatemalan adults	2004	Journal of Nutrition	Exclusion reason: Wrong intervention
Cooper, M., Shanta, S., Mahmud, A.,Roth, D., Gernand, A.	Availability and intake of foods with naturally occurring or added vitamin d in a setting of high vitamin d deficiency	2015	FASEB Journal. Conference: Experimental Biology	Exclusion reason: Wrong intervention
Correa, L. M. M., Sosa, B. E. P., Cadavid, A. D., Mesa, S. L. R., Lopez, L. P. M.	Iron and folate intake during pregnancy and its relationship with maternal biochemical indicators	2012	Iatreia	Exclusion reason: Wrong intervention
Correia‐Santos, A., Bolognini, P. K., Erthal, S. R., Teles, B. G., Blondet, de A.o V.	Dietary supplements for the lactating adolescent mother: influence on plasma micronutrients	2011	Nutricion Hospitalaria	Exclusion reason: Wrong patient population
Costello, A. M. de L., Osrin, D.	Micronutrient status during pregnancy and outcomes for newborn infants in developing countries	2003	Journal of Nutrition	Exclusion reason: Wrong study design
Costello, A., Osrin, D.	Vitamin A supplementation and maternal mortality	2010	Lancet	Exclusion reason: Wrong study design
Coutinho, G. G. P. L., Goloni‐Bertollo, E. M., Pavarino‐Bertelli, E. C.	Effectiveness of two programs of intermittent ferrous supplementation for treating iron‐deficiency anaemia in infants: randomized clinical trial	2008	Sao Paulo Medical Journal	Exclusion reason: Wrong patient population
Coutsoudis, A., Pillay, K., Spooner, E., Kuhn, L., Coovadia, H. M.	Randomized trial testing the effect of vitamin A supplementation on pregnancy outcomes and early mother‐to‐child HIV‐1 transmission in Durban, South Africa	1999	AIDS	Exclusion reason: Wrong patient population
Coutsoudis, A, Pillay, K, Spooner, E, Kuhn, L, Coovadia, Hm.	Randomized trial testing the effect of vitamin A supplementation on pregnancy outcomes and early mother‐to‐child HIV‐1 transmission in Durban, South Africa. South African Vitamin A Study Group	1999	AIDS (london, england)	Exclusion reason: Wrong patient population
Cox, S. E., Arthur, P., Kirkwood, B. R., Yeboah‐Antwi, K., Riley, E. M.	Vitamin A supplementation increases ratios of proinflammatory to anti‐inflammatory cytokine responses in pregnancy and lactation	2006	Clinical and Experimental Immunology	Exclusion reason: Wrong study design
Crider, K. S., Devine, O., Hao, L., Dowling, N. F., Li, S., Molloy, A. M., Li, Z., Zhu, J., Berry, R. J.	Population red blood cell folate concentrations for prevention of neural tube defects: Bayesian model	2014	BMJ (Online)	Exclusion reason: Wrong intervention
Crider, K. S., Cordero, A. M., Yan Ping, Q., Mulinare, J., Dowling, N. F., Berry, R. J.	Prenatal folic acid and risk of asthma in children: a systematic review and meta‐analysis	2013	American Journal of Clinical Nutrition	Exclusion reason: Wrong patient population
Crivellenti, L., Barbieri, P., Sartorelli, D.	Adequacy of folate intake during pregnancy: The role of flour fortification and dietary supplement	2013	Annals of Nutrition and Metabolism	Exclusion reason: Wrong intervention
Crozier, S. R., Harvey, N. C., Inskip, H. M., Godfrey, K.h M., Cooper, C., Robinson, Siân, M.	Maternal vitamin D status in pregnancy is associated with adiposity in the offspring: findings from the Southampton Women's Survey	2012	Am J Clin Nutr	Exclusion reason: Wrong intervention
CTRI/2010/091/000352; Das, V., Agarwal, A., Bhatia, V.	To find the minimum dose of vitamin D required in pregnancy for good maternal and neonatal health	2010		Exclusion reason: Wrong intervention
CTRI/2010/091/001277	Effect of H. pylori eradication therapy in iron deficiency anaemia of pregnancy‐ a randomized double blind placebo controlled clinical trial	2010		Exclusion reason: Wrong patient population
CTRI/2010/091/001290, Shafi, D.	A clinical trial to compare the rise in hemoglobin and serum ferritin levels after giving either intravenous iron sucrose or oral ferrous ascorbate to anemic pregnant women.	2010		Exclusion reason: Wrong patient population
CTRI/2012/12/003199, K. L. E. University Belgaum	Effect of Iron Supplementation for Treatment of Anemia in Pregnancy on Maternal and Newborn Outcomes in Belgaum District, Karnataka	2012		Exclusion reason: Wrong patient population
CTRI/2012/12/003212, Indian Council of Medical Research	Vitamin B12 and protein supplementation in adolescents and its effect in next generation: Pune Intervention Study	2012		Exclusion reason: Wrong patient population
CTRI/2013/04/003577, Next Gen Pharma India Pvt, Ltd	Effect of oral probiotic supplementation during pregnancy and lactation in modulating immune response in breast milk, fetus and neonate	2013		Exclusion reason: Wrong intervention
CTRI/2014/01/004369, K. L. E. Hospital	Comparison of efficacy and safety of intravenous injection of the drug ferric carboxymaltose vs iron sucrose in the treatment of iron deficiency anaemia during pregnancy. A randomized controlled trial	2014		Exclusion reason: Wrong patient population
CTRI/2014/08/004860, breast feeding network of india	Adminstartion of large dose of Vitamin D to pregnant mothers at time of delivery and its effects on the levels of Vitamin D In the infants at 6 months of age	2014		Exclusion reason: Wrong intervention, supplemenation only occurs before birth, not antenatal, not throughout pregnancy.
CTRI/2015/06/005834, International Atomic Energy Agency	Effectiveness of a maternal food based intervention in the 3rd trimester till one month post partum to increase vitamin A intakes through breast milk in early infancy	2015		Exclusion reason: Wrong intervention
CTRI/2015/06/005920, All India Institute Of Medical, Sciences	Weekly home visit and supervision of iron folic acid tablets intake in pregnant women in rural population in Haryana	2015		Exclusion reason: Wrong outcomes
CTRI/2017/05/008553, Datta Meghe Institute of Medical Sciences	To study the effects of maternal nutrition and parenting program on cognitive development of children from rural India at 2 years of age	2017		Exclusion reason: Wrong intervention
CTRI/2017/09/009720, All India Institute of Medical Sciences	Effect of injection iron in improving haemoglobin level among pregnant women	2017		Exclusion reason: Wrong intervention
CTRI/2018/02/012119, Bill Melinda Gates Foundation, USA; Harvard, T. H. Chan School of Public Health	Non‐inferiority of Lower Dose Calcium Supplementation During Pregnancy	2018		Exclusion reason: Wrong intervention
Czeizel, A. E., Vereczkey, A., Szabo, I.	Folic acid in pregnant women associated with reduced prevalence of severe congenital heart defects in their children: a national population‐based case‐control study	2015	European Journal of Obstetrics & Gynecology and Reproductive Biology	Exclusion reason: Wrong intervention
da Silva, C. L., Saunders, C., Szarfarc, S. C., Fujimori, E., da Veiga, G. V.	Anaemia in pregnant women before and after the mandatory fortification of wheat and corn flours with iron	2012	Public Health Nutrition	Exclusion reason: Wrong intervention
Darlow, Brian A, Graham, P J, Rojas‐Reyes, M. X.	Vitamin A supplementation to prevent mortality and short‐ and long‐term morbidity in very low birth weight infants	2016	Cochrane Database of Systematic Reviews	Exclusion reason: Wrong patient population
Darnton‐Hill, I., Mkparu, U. C.	Micronutrients in pregnancy in low‐ and middle‐income countries	2015	Nutrients	Exclusion reason: Wrong study design
Darnton‐Hill, I., Nalubola, R.	Fortification strategies to meet micronutrient needs: successes and failures	2002	Proceedings of the Nutrition Society	Exclusion reason: Wrong study design
Das, J. K., Kumar, R., Salam, R. A., Bhutta, Z. A.	Systematic review of zinc fortification trials. (Special Issue: Zinc.)	2013	Annals of Nutrition and Metabolism	Exclusion reason: Wrong study design
Das, J. K., Salam, R. A., Kumar, R. Bhutta, Z. A.	Micronutrient fortification of food and its impact on woman and child health: a systematic review	2013	Systematic reviews	Exclusion reason: Wrong study design
Das, S., Narayan, S., Rai, S.	Is 400 IU per day of Vitamin‐D given to healthy well‐nourished mothers antenatally enough to prevent neonatal Vitamin‐D deficiency?	2018	Medical Journal Armed Forces India	Exclusion reason: Wrong intervention
Daud, N. A.	Zinc, IGF‐1, and food intervention in malnourished pregnant women on children body height after 6 years in Indonesia	2012	FASEB Journal. Conference: Experimental Biology	Exclusion reason: Wrong patient population
de Silva, L. D., Atukorala, T. M.	Micronutrient status of plantation workers in Sri Lanka during pregnancy and postpartum	1996	Journal of Obstetrics & Gynaecology Research	Exclusion reason: Wrong intervention
Deb, R., Arora, J., Meitei, S. Y., Gupta, S., Verma, V., Saraswathy, K. N., Saran, S., Kalla, A. K.	Folate supplementation, MTHFR gene polymorphism and neural tube defects: a community based case control study in North India	2011	Metabolic Brain Disease	Exclusion reason: Wrong intervention
Deeb, F., Fatim, P.	Multiple micronutrients (MMN) in infertile woman undergoing ovulation induction: A randomized control trial	2015	Journal of Obstetrics and Gynaecology Research	Exclusion reason: Wrong patient population
Deitchler, M., Mason, J., Mathys, E., Winichagoon, P., Tuazon, M. A.	Lessons from successful micronutrient programs. Part I: program initiation	2004	Food & Nutrition Bulletin	Exclusion reason: Wrong study design
Deitchler, M., Mathys, E., Mason, J., Winichagoon, P., Tuazon, M. A.	Lessons from successful micronutrient programs. Part II: program implementation	2004	Food & Nutrition Bulletin	Exclusion reason: Wrong study design
del Rio Garcia, C., Torres‐Sanchez, L., Chen, J., Schnaas, L., Hernandez, C., Osorio, E., Portillo, M. G., Lopez‐Carrillo, L.	Maternal MTHFR 677 C>T genotype and dietary intake of folate and vitamin B_12_: Their impact on child neurodevelopment	2009	Nutritional Neuroscience	Exclusion reason: Wrong patient population
Deng, J., Xie, L., Liu, G. L., Yang, J. Y.	Meta‐analysis of effect of n‐3 long‐chain polyunsaturated fatty acid supplementation of pregnant women on head circumference of newborn infants [Article in Chinese]	2012	Chung‐Hua Yu Fang i Hsueh Tsa Chih [Chinese Journal of Preventive Medicine]	Exclusion reason: Wrong study design
Deng, J., Li, X., Ding, Z., Wu, Y., Chen, X., Xie, L.	Effect of DHA supplements during pregnancy on the concentration of PUFA in breast milk of Chinese lactating mothers	2017	Journal of Perinatal Medicine	Exclusion reason: Wrong patient population
De‐Regil, L. M., Peña‐Rosas, J. P., Fernández‐Gaxiola, A. C., Rayco‐Solon, P.	Effects and safety of periconceptional oral folate supplementation for preventing birth defects	2015	Cochrane Database of Systematic Reviews	Exclusion reason: Wrong study design
Dessie, M. A., Zeleke, E. G., Workie, S. B., Berihun, A. W.	Folic acid usage and associated factors in the prevention of neural tube defects among pregnant women in Ethiopia: Cross‐sectional study	2017	BMC Pregnancy and Childbirth	Exclusion reason: Wrong intervention
Devakumar, D., Fall, C. H. D., Sachdev, H. S., Margetts, B. M., Osmond, C., Wells, J. C. K., Costello, A., Osrin, D.	Multiple micronutrient supplementation in pregnancy and long‐term health outcomes in children: A systematic review and metaanalysis	2016	Archives of Disease in Childhood	Exclusion reason: Wrong study design
Dewey, K. G.	Heterogeneity in response to nutrition interventions during the first 1000 days: Evidence from randomized controlled trials using lipid‐based nutrient supplements for mothers and infants	2017	Annals of Nutrition and Metabolism	Exclusion reason: Wrong study design
Dewey, K., Ashorn, P.	Introduction to the international lipidbased nutrient supplements (iLiNS) project	2013	Annals of Nutrition and Metabolism	Exclusion reason: Wrong study design
Diallo, D., Tchernia, G., Yvart, J., Sidibe, H., Kodio, B., Diakite, S.	Role of iron deficiency in anaemia in pregnant women in Mali [Article in French]	1995	Revue Francaise de Gynecologie et d Obstetrique	Exclusion reason: Wrong study design
Diamond‐Smith, N. G., Gupta, M., Kaur, M., Kumar, R.	Determinants of persistent anemia in poor, urban pregnant women of chandigarh city, north india: a mixed method approach	2016	Food & Nutrition Bulletin	Exclusion reason: Wrong study design
Dibley, M. J., Titaley, C. R., d'Este, C., Agho, K.	Iron and folic acid supplements in pregnancy improve child survival in Indonesia	2012	American Journal of Clinical Nutrition	Exclusion reason: Wrong intervention;
Dibley, M., Titaley, C., Agho, K., Patel, A., Badhoniya, N., Khambalia, A.	Iron and folic supplementation during pregnancy reduces risk of neonatal mortality in India	2011	FASEB Journal. Conference: Experimental Biology	Exclusion reason: Wrong intervention
Dickerson, T., Crookston, B., Simonsen, S. E., Sheng, X., Samen, A., Nkoy, F.	Pregnancy and village outreach tibet: A descriptive report of a community‐ and home‐based maternal‐newborn outreach program in rural tibet	2010	Journal of Perinatal and Neonatal Nursing	Exclusion reason: Wrong study design
Divakar, H.	Iron‐deficiency anaemia in pregnant women: What preventing practitioners from using IV iron sucrose	2012	International Journal of Infertility and Fetal Medicine	Exclusion reason: Wrong intervention
Djekic‐Ivankovic, M., Weiler, H., Jones, G., Kaufmann, M., Kaludjerovic, J., Aleksic‐Velickovic, V., Mandic, L. M., Glibetic, M.	Vitamin D status in mothers with pre‐eclampsia and their infants: a case‐control study from Serbia, a country without a Vitamin D fortification policy	2017	Public Health Nutrition	Exclusion reason: Wrong intervention
Dong, C., Ge, P., Ren, X., Zhao, X., Fan, H., Yin, S.‐A., Weiderpass, E.	Evaluating the micronutrient status of women of child‐bearing age living in the rural disaster areas one year after Wenchuan Earthquake	2014	Asia Pacific Journal of Clinical Nutrition	Exclusion reason: Wrong intervention
Dorsey, J, Schulze, K, Wu, L, Khatry, S, LeClerq, S, Christian, P, West Jr, K.	Influence of Vitamin a status on serum retinol and beta‐carotene concentrations associated with dark green leafy vegetable (DGLV) intake during pregnancy in Nepal	2015	FASEB Journal	Exclusion reason: Wrong intervention
DRKS00007736, Oxford University, Centre for Tropical Medicine, Global Health, Nuffield Department of Medicine Research Building	Impact of enriched food ration distribution to pregnant women in Maela refugee camp	2015		Exclusion reason: Wrong intervention
Dufour, D. L., Reina, J. C., Spurr, G. B.	Food and macronutrient intake of economically disadvantaged pregnant women in Colombia	1999	American Journal of Human Biology	Exclusion reason: Wrong study design
Duggan, M. B.	Nutritional update: relevance to maternal and child health in East Africa	2003	African Health Sciences	Exclusion reason: Wrong study design
Dutta, A. J., Prakash, P., Bansal, R. K.	Compliance to iron supplementation among pregnant women: a cross sectional study in urban slum	2014	National Journal of Community Medicine	Exclusion reason: Wrong study design
Dutta, H. K., Baruah, M., Borbora, D.	Maternal nutrition and the risk of congenital malformations in the tea garden community of Assam, Northeast India	2016	Clinical Epidemiology and Global Health	Exclusion reason: Wrong study design
Dwarkanath, P., Barzilay, J. R., Thomas, T., Thomas, A., Bhat, S., Kurpad, A. V.	High folate and low vitamin B‐12 intakes during pregnancy are associated with small‐for‐gestational age infants in South Indian women: A prospective observational cohort study1‐4	2013	American Journal of Clinical Nutrition	Exclusion reason: Wrong intervention
Dwarkanath, P., Muthayya, S., Thomas, T., Vaz, M., Parikh, P., Mehra, R., Kurpad, A. V.	Polyunsaturated fatty acid consumption and concentration among South Indian women during pregnancy	2009	Asia Pacific Journal of Clinical Nutrition	Exclusion reason: Wrong study design
Ebrahim, Z.	EPA and DHA: Evidence based health claims, recommended dosages and implementation thereof	2010	South African Journal of Clinical Nutrition	Exclusion reason: Wrong study design
Ebuy, Y., Alemayehu, M., Mitiku, M., Goba, G. K.	Determinants of severe anaemia among laboring mothers in Mekelle city public hospitals, Tigray region, Ethiopia	2017	PLoS ONE	Exclusion reason: Wrong study design
Echeverri, P. E. M., Leguizamo, M. R. H., Manjarres, L. M.	Exploring the effectiveness and safety of ferrous fumarate preparations and folic acid in the prevention of anaemia in pregnant women. [Spanish, English]	2011	Vitae	Exclusion reason: Wrong intervention
Ejidokun, O. O.	Maternal anaemia and morbidity in South‐western Nigeria	1996		Exclusion reason: Wrong intervention
Ekstrom, E.‐C. M.	Iron supplementation during pregnancy in Tanzania: Determinants and hematologic consequences of adherence	1995		Exclusion reason: Wrong study design
El Koumi, M. A., Ali, Y. F., Abd El Rahman, R. N.	Impact of maternal vitamin D status during pregnancy on neonatal vitamin D status	2013	Turkish Journal of Pediatrics	Exclusion reason: Wrong intervention
Elert, V. W., Machado, A. K. F., Pastore, C. A.	Anemia in pregnancy: prevalence and related nutritional aspects in parturients of a public hospital of southern Brazil	2013	Alimentos e Nutricao	Exclusion reason: Wrong intervention
Elhusseini, M. A., Kassab, M., Krawinkel, M., Hussein, L., El‐Shabrawi, M. H. F.	Folic acid intake and neural tube defects (NTDS) in a cohort of Egyptian mothers	2012	International Journal of Gynecology and Obstetrics	Exclusion reason: Wrong intervention
Elsen, C., Rivas‐Echeverria, C., Sahland, K., Sanchez, R., Molma, L., Pahl, L., Wallinger, R., Volz, J., Wacker, J., Fruhauf, J.	Vitamins E, A and Bas possible risk factors for preeclampsia under consideration of the PROPER study ("prevention of preeclampsia by high‐dose riboflavin supplementationo")	2012	Geburtshilfe und Frauenheilkunde	Exclusion reason: Wrong study design
Escamilla‐Nunez, M. C., Barraza‐Villarreal, A., Hernandez‐Cadena, L., Navarro‐Olivos, E., Sly, P. D., Romieu, I.	Omega‐3 fatty acid supplementation during pregnancy and respiratory symptoms in children	2014	Chest	Exclusion reason: Wrong intervention
Faisel, H., Pittrof, R.	Vitamin A and causes of maternal mortality: association and biological plausibility	2000	Public Health Nutrition	Exclusion reason: Wrong study design;
Fajobi, O. A. Schulze, K. West, K. P., Christian, P.	Vitamin D deficiency, risk factors and morbidity in early pregnancy in rural Nepal	2011	FASEB Journal. Conference: Experimental Biology	Exclusion reason: Wrong study design

**Table 8:** Characteristics table of excluded studies (Database Searches)

Authors	Title	Published Year	Journal	Exclusion Reason
Farebrother, J., Naude, C. E., Nicol, L., Sang, Z., Yang, Z., Andersson, M., Jooste, P. L., Zimmermann, M. B.	Systematic review of the effects of iodised salt and iodine supplements on prenatal and postnatal growth: study protocol	2015	BMJ Open	Exclusion reason: Wrong study design
Fatemi, F., Mohammadzadeh, A., Sadeghi, M. R., Akhondi, M. M., Mohammadmoradi, S., Kamali, K., Lackpour, N., Jouhari, S., Zafadoust, S., Mokhtar, S., Giahi, L.	Role of vitamin E and D3 supplementation in Intra‐Cytoplasmic Sperm Injection outcomes of women with polycystic ovarian syndrome: a double blinded randomized placebo‐controlled trial	2017	Clinical Nutrition ESPEN	Exclusion reason: Wrong patient population
Fathi Najafi, T., Latifnejad Roudsari, R.	Iron supplementation protocols for iron deficiency anaemia: A comparative review of iron regimens in three countries of India, Iran and England	2015	International Journal of Gynecology and Obstetrics	Exclusion reason: Wrong intervention
Feldhaus, I., LeFevre, A. E., Rai, C., Bhattarai, J., Russo, D., Rawlins, B., Chaudhary, P., Thapa, K.	Optimizing treatment for the prevention of pre‐eclampsia/eclampsia in Nepal: Is calcium supplementation during pregnancy cost‐effective	2016	Cost Effectiveness and Resource Allocation	Exclusion reason: Wrong study design
Feleke Demissie, E., Bhagwan Singh, C.	Levels of major and trace elements in fennel (Foeniculum vulgari Mill.) fruits cultivated in Ethiopia	2015	SpringerPlus	Exclusion reason: Wrong intervention
Ferguson, S. A.	Why ferritin measurement at 28 weeks ofgestation is advisable	2013	BJOG: An International Journal of Obstetrics and Gynaecology	Exclusion reason: Wrong intervention
Ferlin, M. L. S., Jorge, S. M., Ricco, R. G., Martinez, F. E.	Prematurity anaemia: effect of iron supplementation (Special issue: Celebrating Exciting Nutrition Research in the Next Century)	2001	Nutrition Research	Exclusion reason: Wrong study design
Filteau, S. M., Rice, A. L., Ball, J. J., Chakraborty, J., Stoltzfus, R., Francisco, A. de, Willumsen, J. F.	Breast milk immune factors in Bangladeshi women supplemented postpartum with retinol or beta ‐carotene	1999	American Journal of Clinical Nutrition	Exclusion reason: Wrong patient population
Finkelstein, J. L., O'Brien, K. O., Abrams, S. A., Zavaleta, N.	Infant iron status affects iron absorption in Peruvian breastfed infants at 2 and 5 mo of age	2013	American Journal of Clinical Nutrition	Exclusion reason: Wrong patient population
Finkelstein, J., Duggan, C., Thomas, T., Bose, B., Samuel, T., Srinivasan, K., Kurpad, A.	Maternal anaemia, iron deficiency, and pregnancy outcomes in India	2014	FASEB Journal. Conference: Experimental Biology	Exclusion reason: Wrong intervention
Finkelstein, J., Kurpad, A., Thomas, T., Bose, B., Samuel, T., Srinivasan, K., Duggan, C.	Vitamin B12 status in pregnant women and their children in India	2014	FASEB Journal. Conference: Experimental Biology	Exclusion reason: Wrong intervention
Fracassi, P.	Beyond a conceptual framework: an applied method to assess the potential impact of multi‐sectoral approaches on the reduction of child stunting in yemen (2013‐2014)	2017		Exclusion reason: Wrong study design
Friedrisch, J. R., Friedrisch, B. K.	Prophylactic Iron Supplementation in Pregnancy: A Controversial Issue	2017	Biochemistry Insights	Exclusion reason: Wrong intervention
Frith, A. L., Frongillo, E.	The influence of maternal nutritional and support interventions and stress on maternal ‐infant feeding interactions in Bangladesh	2006		Exclusion reason: Wrong intervention
Fujimori, E., Sato, A. P. S., Szarfarc, S. C., da Veiga, G. V., de Oliveira, V. A., Colli, C., Moreira‐Araujo, R. S. R., de Arruda, I. K. G., Uchimura, T. T., Brunken, G. S., Yuyama, L. K. O., Muniz, P. T., Priore, S. E., Tsunechiro, M. A., Frazao, A. G. F., Passoni, C. R. M. S., Araujo, C. R. M. A.	Anemia in Brazilian pregnant women before and after flour fortification with iron	2011	Revista de Saude Publica	Exclusion reason: Wrong intervention
Fujimori, E., Vianna de Oliveira, I. M., Nunez de Cassana, L. M., Cornbluth Szarfarc, S.	Iron nutritional status in pregnant adolescents, Sao Paulo, Brazil	1999	Archivos Latinoamericanos de Nutricion	Exclusion reason: Wrong intervention
Gadallah, M., Rady, M., Salem, B., Aly, E. M., Anwer, W.	The effect of nutritional intervention program on the prevalence of anaemia among pregnant women in rural areas of Belbis district‐Sharkia Governorate‐Egypt	2002	Journal of the Egyptian Public Health Association	Exclusion reason: Wrong intervention
Gadgil, M., Joshi, K., Pandit, A., Otiv, S., Joshi, R., Brenna, J. T., Patwardhan, B.	Imbalance of folic acid and vitamin B12 is associated with birth outcome: an Indian pregnant women study	2014	European Journal of Clinical Nutrition	Exclusion reason: Wrong intervention
Ganle, J. K.	Ethnic disparities in utilisation of maternal health care services in Ghana: evidence from the 2007 Ghana Maternal Health Survey	2016	Ethnicity & Health	Exclusion reason: Wrong intervention
Garcia‐Solis, P., Solis, S. Jc, Garcia‐Gaytan, A. C., Reyes‐Mendoza, V. A., Robles‐Osorio, L., Hernandez‐Montiel, H. L., Leo‐Amador, G. E.	Iodine nutrition status in pregnant women in Mexico	2011	Thyroid	Exclusion reason: Wrong intervention
Garg, P., Pejaver, R. K., Sukhija, M., Ahuja, A.	Role of DHA, ARA, & phospholipids in brain development: An Indian perspective	2017	Clinical Epidemiology and Global Health	Exclusion reason: Wrong intervention
Gebreamlak, B., Dadi, A. F., Atnafu, A.	High adherence to iron/folic acid supplementation during pregnancy time among antenatal and postnatal care attendant mothers in Governmental Health Centers in Akaki Kality Sub City, Addis Ababa, Ethiopia: Hierarchical negative binomial poisson regression	2017	PLoS ONE	Exclusion reason: Wrong study design
Gebremedhin, S., Samuel, A., Mamo, G., Moges, T., Assefa, T.	Coverage, compliance and factors associated with utilization of iron supplementation during pregnancy in eight rural districts of Ethiopia: a cross‐sectional study	2014	BMC Public Health	Exclusion reason: Wrong intervention
Gedefaw, A., Teklu, S., Tadesse, B. T.	Magnitude of Neural Tube Defects and Associated Risk Factors at Three Teaching Hospitals in Addis Ababa, Ethiopia	2018	BioMed Research International	Exclusion reason: Wrong intervention
Geethanath, R. M., Ramji, S., Thirupuram, S., Rao, Y. N.	Effect of timing of cord clamping on the iron status of infants at 3 months	1997	Indian Pediatrics	Exclusion reason: Wrong patient population
Geller, S. E., Goudar, S. S., Adams, M. G., Naik, V. A., Patel, A., Bellad, M. B., Patted, S. S., Edlavitch, S. A., Moss, N., Kodkany, B. S., Derman, R. J.	Factors associated with acute postpartum hemorrhage in low‐risk women delivering in rural India	2008	International Journal of Gynecology & Obstetrics	Exclusion reason: Wrong patient population
Gewa, C. A., Frankenfeld, C. L., Slavin, M., Omondi, M.	Fish‐enhanced and soybean‐enhanced supplemental snacks are acceptable among pregnant women in rural Kenya	2014	Food and nutrition bulletin	Exclusion reason: Wrong study design
Ghomian, N., Lotfalizade, M., Movahedian, A.	Comparative study of serum level of vitamin D in pregnant women with preeclampsia and normal pregnant women	2015	Iranian Journal of Obstetrics, Gynecology and Infertility	Exclusion reason: Wrong study design
Ghosh, R., Sharma, A. K.	Intra‐ and inter‐household differences in antenatal care, delivery practices and postnatal care between last neonatal deaths and last surviving children in a peri‐urban area of India	2010	Journal of Biosocial Science	Exclusion reason: Wrong outcomes
Gitau, R., Makasa, M., Kasonka, L., Sinkala, M., Chintu, C., Tomkins, A., Filteau, S.	Maternal micronutrient status and decreased growth of Zambian infants born during and after the maize price increases resulting from the southern African drought of 2001‐2002	2005	Public Health Nutrition	Exclusion reason: Wrong patient population
Gonzalez‐Casanova, I., Nguyen, P., Harding, K., Reinhart, G., Nguyen, H., Truong, T., Pham, H., Nguyen, S., Martorell, R., Ramakrishnan, U.	Predictors of adherence to preconception and prenatal micronutrient supplementation in Vietnam	2015	FASEB Journal. Conference: Experimental Biology	Exclusion reason: Wrong intervention
Gonzalez‐Casanova, I., Rzehak, P., Hao, W., Aryeh, S., Barraza‐Villarreal, A., Garcia‐Feregrino, R., Rivera, J., Romieu, I., Villalpando, S., Koletzko, B., Ramakrishnan, U.	Fatty acid desaturase single nucleotide polymorphisms modify the effect of prenatal supplementation with docosahexaenoic acid on birth weight	2015	FASEB Journal. Conference: Experimental Biology	Exclusion reason: Wrong intervention
Gonzalez‐Casanova, I., Rzehak, P., Stein, A. D., Feregrino, R. G., Dommarco, J. A. R., Barraza‐Villarreal, A., Demmelmair, H., Romieu, I., Villalpando, S.,Martorell, R., Koletzko, B., Usha, R.	Maternal single nucleotide polymorphisms in the fatty acid desaturase 1 and 2 coding regions modify the impact of prenatal supplementation with DHA on birth weight	2016	American Journal of Clinical Nutrition	Exclusion reason: Wrong intervention
Gonzalez‐Casanova, I, Stein, Ad, Hao, W, Garcia‐Feregrino, R, Barraza‐Villarreal, A, Romieu, I, Rivera, Ja, Martorell, R, Ramakrishnan, U.	Prenatal Supplementation with Docosahexaenoic Acid Has No Effect on Growth through 60 Months of Age	2015	Journal of Nutrition	Exclusion reason: Wrong intervention
Greiner, T.	Vitamins and minerals for women: recent programs and intervention trials	2011	Nutrition Research and Practice	Exclusion reason: Wrong study design
Grosbois, B., Decaux, O., Cador, B., Cazalets, C., Jego, P.	Human iron deficiency	2005	Bulletin de l'Academie Nationale de Medecine	Exclusion reason: Wrong study design
Gulati, R., Bailey, R., Prentice, A. M., Brabin, B. J., Owens, S.	Haematological effects of multimicronutrient supplementation in non‐pregnant Gambian women	2009	European Journal of Clinical Nutrition	Exclusion reason: Wrong patient population
Gunaratna, N. S., Masanja, H., Mrema, S., Levira, F., Spiegelman, D., Hertzmark, E., Saronga, N., Irema, K., Shuma, M., Elisaria, E., Fawzi, W.	Multivitamin and iron supplementation to prevent periconceptional anaemia in rural Tanzanian women: A randomized, controlled trial	2015	PLoS ONE	Exclusion reason: Wrong patient population
Gunaratne, A. W, Makrides, M., Collins, C. T.	Maternal prenatal and/or postnatal n‐3 long chain polyunsaturated fatty acids (LCPUFA) supplementation for preventing allergies in early childhood	2015	Cochrane Database of Systematic Reviews	Exclusion reason: Wrong study design
Gur, G., Abac, A., Koksoy, A. Y., Ank, A., Catl, G., Kslal, F. M., Akn, K. O., Andran, N.	Incidence of maternal vitamin D deficiency in a region of Ankara, Turkey: a preliminary study	2014	Turkish Journal of Medical Sciences	Exclusion reason: Wrong patient population
Gutierrez‐Gomez, Y., Stein, A. D., Usha, R., Barraza‐Villarreal, A., Moreno‐Macias, H., Aguilar‐Salinas, C., Romieu, I., Rivera, J. A.	Prenatal docosahexaenoic acid supplementation does not affect nonfasting serum lipid and glucose concentrations of offspring at 4 years of age in a follow‐up of a randomized controlled clinical trial in Mexico	2017	Journal of Nutrition	Exclusion reason: Wrong intervention
Guyon, A. B., Quinn, V. J., Hainsworth, M., Ravonimanantsoa, P., Ravelojoana, V., Rambeloson, Z., Martin, L.	Implementing an integrated nutrition package at large scale in Madagascar: the Essential Nutrition Actions framework	2009	Food and nutrition bulletin	Exclusion reason: Wrong study design
Gyorkos, T. W., Gilbert, N. L., Larocque, R., Casapia, M.	Hookworm and Trichuris infections associated with anemia during pregnancy	2010	American Journal of Tropical Medicine and Hygiene	Exclusion reason: Wrong intervention
Haber, J. A., Solomons, N. W., Hampel, D. A., Orozco, M., Allen, L. H.	The short‐term response of breast milk micronutrient concentrations to a lipid‐based nutrient supplement in Guatemalan women	2016	Annals of Global Health	Exclusion reason: Wrong patient population
Habimana, L., Twite, K. E., Daumerie, C., Wallemacq, P., Donnen, P., Kalenga, M. K., Robert, A.	High prevalence of thyroid dysfunction among pregnant women in Lubumbashi, Democratic Republic of Congo	2014	Thyroid	Exclusion reason: Wrong study design
Habimana, L., Twite, K. E., Wallemacq, P., De Nayer, P., Daumerie, C., Donnen, P., Kalenga, M. K., Robert, A.	Iodine and iron status of pregnant women in Lubumbashi, Democratic Republic of Congo	2013	Public Health Nutrition	Exclusion reason: Wrong study design
Haider, B. A., Humayun, Q., Bhutta, Z. A.	Effect of administration of antihelminthics for soil transmitted helminths during pregnancy	2009	Cochrane Database of Systematic Reviews	Exclusion reason: Wrong intervention
Hajifoghaha, M., Keshavarz, T., Parsanejad, M. E., Fard, A. R.	Vitamin C supplementation and PROM	2008	Iranian Journal of Obstetrics, Gynecology and Infertility	Exclusion reason: Wrong intervention
Halicioglu, O., Aksit, S., Koc, F., Akman, S. A., Albudak, E., Yaprak, I., Coker, I., Colak, A., Ozturk, C., Gulec, E. S.	Vitamin D deficiency in pregnant women and their neonates in spring time in western Turkey	2012	Paediatric and Perinatal Epidemiology	Exclusion reason: Wrong intervention
Halicioglu, O., Sutcuoglu, S., Koc, F., Yildiz, O., Akman, S. A., Aksit, S.	Vitamin D status of exclusively breastfed 4‐month‐old infants supplemented during different seasons	2012	Pediatrics	Exclusion reason: Wrong patient population
Hambidge, K. M., Miller, L. V., Mazariegos, M., Westcott, J., Solomons, N. W., Raboy, V. Kemp, J. F., Das, A., Goco, N., Hartwell, T., Wright, L., Krebs, N. F.	Upregulation of Zinc absorption matches increases in physiologic requirements for Zinc in women consuming high‐ or moderate‐phytate diets during late pregnancy and early lactation	2017	Journal of Nutrition	Exclusion reason: Wrong patient population
Han, A., Rotermann, M., Fuller‐Thomson, E., Ray, J. G.	Pre‐conceptional folic acid supplement use according to maternal country of birth	2009	Journal of Obstetrics & Gynaecology Canada: JOGC	Exclusion reason: Wrong patient population
Han, J. H., Li, X. Y., Li, Y. P.	Risk assessment of fortification level for vitamin A in food in China [Article in Chinese]	2012	Chung‐Hua Yu Fang i Hsueh Tsa Chih [Chinese Journal of Preventive Medicine]	Exclusion reason: Wrong intervention
Han, X. X., Sun, Y. Y., Ma, A. G., Yang, F., Zhang, F. Z., Jiang, D. C., Li, Y.	Moderate NaFeEDTA and ferrous sulfate supplementation can improve both hematologic status and oxidative stress in anemic pregnant women	2011	Asia Pacific Journal of Clinical Nutrition	Exclusion reason: Wrong patient population; Women recruited were anemic
Hao, N., Xia, W., Tang, Y., Wu, M., Jiang, H., Lin, X., Liu, J., Zhou, D.	Periconceptional folic acid supplementation among pregnant women with epilepsy in a developing country: a retroprospective survey in China	2015	Epilepsy & Behavior	Exclusion reason: Wrong intervention
Hao, Y. Tian, S., Jiao, X., Mi, N., Zhang, B., Song, T., An, L., Zheng, X., Zhuang, D.	Association of parental environmental exposures and supplementation intake with risk of nonsyndromic orofacial clefts: a case‐control study in Heilongjiang Province, China	2015	Nutrients	Exclusion reason: Wrong intervention
Harding, K. L., Matias, S. L., Mridha, M. K., Moniruzzaman, M., Vosti, S. A., Hussain, S., Dewey, K. G., Stewart, C. P.	Adherence to recommendations on lipid‐based nutrient supplement and iron and folic acid tablet consumption among pregnant and lactating women participating in a community health programme in northwest Bangladesh	2017	Maternal and Child Nutrition	Exclusion reason: Wrong intervention
Harding, K. B; Peña‐Rosas, J. P., Webster, A. C., Yap, C. M., Payne, B. A., Ota, E., De‐Regil, L. M.	Iodine supplementation for women during the preconception, pregnancy and postpartum period	2017	Cochrane Database of Systematic Reviews	Exclusion reason: Wrong study design
Haribalakrishna, B., Nanavati, R. N., Kabra, N. S.	Effect of two different doses of parenteral amino acid supplementation on postnatal growth of very low birth weight neonates ‐ a randomized controlled trial	2013	Indian Pediatrics	Exclusion reason: Wrong patient population
Harika, R., Faber, M., Samuel, F., Kimiywe, J., Afework, Mulugeta, Eilander, A.	Micronutrient status and dietary intake of iron, vitamin A, iodine, folate and zinc in women of reproductive age and pregnant women in Ethiopia, Kenya, Nigeria and South Africa: a systematic review of data from 2005 to 2015	2017	Nutrients	Exclusion reason: Wrong study design
Hartriyanti, Yayuk, Suyoto, Perdana S. T., Muhammad, Harry F. L., Palupi, Ika R.	Nutrient intake of pregnant women in Indonesia: a review	2012	Malaysian Journal of Nutrition	Exclusion reason: Wrong study design
Hasanzadeh, M., Ayatollahi, H., Ahmadi, S., Namaghi, S. R.	Comparison of serum vitamin D levels in women with gestational trophoblastic neoplasia and healthy women	2016	Tehran University Medical Journal	Exclusion reason: Wrong study design
Haug, A., Christophersen, O. A., Kinabo, J., Kaunda, W., Eik, L. O.	Use of dried kapenta (Limnothrissa miodon and Stolothrissa tanganicae) and other products based on whole fish for complementing maize‐based diets	2010	African Journal of Food, Agriculture, Nutrition and Development	Exclusion reason: Wrong intervention
Hawkesworth, S, Prentice, A., Fulford, A., Moore, S.	Maternal protein‐energy supplementation does not affect adolescent blood pressure in the Gambia	2009	International Journal of Epidemiology	Exclusion reason: Wrong patient population
Hazavehei, S. M. M., Etesamifard, T., Moeini, B., Roshanaei, G., Mahboubi, M.	Education based on BASNEF model; an affective education on regular use of nutritional supple‐ments during pregnancy	2014	Journal of Biology and Today's World	Exclusion reason: Wrong intervention
He, S., Zhu, H., Zhu, X., Zhou, D., Chen, L.	Pregnancy and perinatal outcome in Chinese epileptic women: Prospective continuous hospital based registration study	2016	Epilepsia	Exclusion reason: Wrong patient population
He, Y., Pan, A., Hu, F. B., Ma, X.	Folic acid supplementation, birth defects, and adverse pregnancy outcomes in Chinese women: A population‐based mega‐cohort study	2016	The Lancet	Exclusion reason: Wrong intervention
Heckert, J., Richter, S., Iruhiriye, E., Leroy, J., Olney, D., Ruel, M.	Cost and cost‐effectiveness of food‐assisted maternal and child health and nutrition programs in Burundi and Guatemala	2017	Annals of Nutrition and Metabolism	Exclusion reason: Wrong intervention
Heidkamp, R., Clermont, A., Phillips, E.	Modeling the Impact of Nutrition Interventions on Birth Outcomes in the Lives Saved Tool (LiST)	2017	Journal of Nutrition	Exclusion reason: Wrong study design
Hekmat, K., Bagheri, R., Abedi, P., Tabesh, H.	The relationship of fat soluble antioxidants with gestational diabetes in Iran: A case‐control study	2014	Journal of Maternal‐Fetal and Neonatal Medicine	Exclusion reason: Wrong intervention
Gupta, H., Gupta, P.	Neural tube defects and folic acid	2004	Indian Pediatrics	Exclusion reason: Wrong study design
Hernandez‐Herrera, R. J., Alcala‐Galvan, L. G., Flores‐Santos, R.	Neural defect prevalence in 248,352 consecutive newborns. [Article in Spanish]	2008	Revista medica del Instituto Mexicano del Seguro Social	Exclusion reason: Wrong patient population
Hess, S. Y., Bado, L., Aaron, G. J., Ouedraogo, J. B., Zeilani, M., Brown, K. H.	Acceptability of zinc‐fortified, lipid‐based nutrient supplements (LNS) prepared for young children in Burkina Faso	2011	Maternal and Child Nutrition	Exclusion reason: Wrong patient population;
Ho, J. J.	Folic acid in the prevention of neural tube defect‐‐a programme for Malaysia?	2004	Medical Journal of Malaysia	Exclusion reason: Wrong intervention
Hofmeyr, G. J., Lawrie, T. A., Atallah, Álvaro N., Duley, L., Torloni, M. R.	Calcium supplementation during pregnancy for preventing hypertensive disorders and related problems	2014	Cochrane Database of Systematic Reviews	Exclusion reason: Wrong study design
Hofmeyr, G. J., Manyame, S.	Calcium supplementation commencing before or early in pregnancy, or food fortification with calcium, for preventing hypertensive disorders of pregnancy	2017	Cochrane Database of Systematic Reviews	Exclusion reason: Wrong study design
Hofmeyr, G. J., Roodt, A., Atallah, A. N., Duley, L.	Calcium supplementation to prevent preeclampsia ‐ a systematic review	2003	SAMJ South African Medical Journal	Exclusion reason: Wrong study design
Holmes, W., Toole, M.	Micronutrient supplements in pregnant Nepalese women	2005	Lancet	Exclusion reason: Wrong study design
Horjus, P., Aguayo, V. M., Roley, J. A., Pene, M. C., Meershoek, S. P.	School‐based iron and folic acid supplementation for adolescent girls: findings from Manica Province, Mozambique	2005	Food and nutrition bulletin	Exclusion reason: Wrong patient population
Horton, S., Mosha, T., Saleh, N., Belt, J., Ndau, E., Walters, D.	Cost‐effectiveness of using sunflower oil fortified with vitamin A: Results from Tanzania	2017	Annals of Nutrition and Metabolism	Exclusion reason: Wrong intervention
Hovdenak, N., Haram, K.	Influence of mineral and vitamin supplements on pregnancy outcome	2012	European Journal of Obstetrics & Gynecology and Reproductive Biology	Exclusion reason: Wrong study design
Huda, S. N., Grantham‐McGregor, S. M., Tomkins, A.	Cognitive and motor functions of iodine‐deficient but euthyroid children in Bangladesh do not benefit from iodized poppy seed oil (Lipiodol)	2001	Journal of Nutrition	Exclusion reason: Wrong patient population
Huffman, S. L., Harika, R. K., Eilander, A., Osendarp, S. J. M.	Essential fats: how do they affect growth and development of infants and young children in developing countries? A literature review. (Special Issue: Consequences of malnutrition in early life and strategies to improve maternal and child diets through targeted fortified products.)	2011	Maternal and Child Nutrition	Exclusion reason: Wrong study design
Huma, N., Salim Ur, R., Anjum, F. M., Murtaza, M. A., Sheikh, M. A.	Food fortification strategy‐‐preventing iron deficiency anaemia: a review	2007	Critical Reviews in Food Science & Nutrition	Exclusion reason: Wrong study design
Hurt, L., Asbroek, A. ten; Amenga‐Etego, S., Zandoh, C., Danso, S., Edmond, K., Hurt, C., Tawiah, C., Hill, Z., Fenty, J., Owusu‐Agyei, S., Campbell, O. M., Kirkwood, B. R.	Effect of vitamin A supplementation on cause‐specific mortality in women of reproductive age in Ghana: a secondary analysis from the ObaapaVitA trial	2013	Bulletin of the World Health Organization	Exclusion reason: Wrong patient population; Population is mixed and results are not disaggregated by pregnant and non‐pregnant women
Imdad, A., Jabeen, A., Bhutta, Z. A.	Role of calcium supplementation during pregnancy in reducing risk of developing gestational hypertensive disorders: a meta‐analysis of studies from developing countries	2011	BMC Public Health	Exclusion reason: Wrong study design
Imhoff‐Kunsch, B. C., Stein, A. D., Villalpando, S., Martorell, R., Ramakrishnan, U.	Docosahexaenoic acid supplementation from mid‐pregnancy through parturition influenced breast milk fatty acid composition at 1 month post‐partum in a double‐blind randomized controlled trial in Mexico	2009	The FASEB Journal. Conference: Experimental Biology	Exclusion reason: Wrong intervention
Ingram, C. F., Fleming, A. F., Patel, M., Galpin, J. S.	Pregnancy‐and lactation‐related folate deficiency in South Africa ‐ A case for folate food fortification	2001	South African Journal of Obstetrics and Gynaecology	Exclusion reason: Wrong patient population
IRCT138811203312N1, Yazd Diabetes Research Center, Shahid Sadoughi University of Medical Sciences	Effect of different doses of vitamin D on insulin resistance in pregnant women attending in Shahid Sadoughi and Mojibian prenatal clinics	2011		Exclusion reason: Wrong outcomes
IRCT20091114002709N47, Iran University of Medical Sciences	The effect of vitamin C supplementation in the last month of pregnancy on neonatal bilirubin level	2018		Exclusion reason: Wrong intervention
IRCT2012122611717N2, Vice Chancellor for Research, Shiraz University of Medical Sciences	The effect of omega‐3fatty acid capsules on anexiety and quality of life gravida during pregnancy depression	2013		Exclusion reason: Wrong outcomes
IRCT2014022316701N1, Hamadan University of Medical Sciences ‐ Vice Chanellor for Research Technology	The effect of education on prenatal supplements	2014		Exclusion reason: Wrong intervention
IRCT201512011113N3 Barij Esans Company	Effect of Chamomile, Ginger and Vitamin B6 on nausea and vomiting due to pregnancy	2016		Exclusion reason: Wrong intervention
IRCT201601045623N64, Vice Chancellor for Research, Kashan University of Medical Sciences	Effect of supplementation in treatment of pregnant women at risk for intrauterine growth restriction	2016		Exclusion reason: Wrong patient population
IRCT201602035440N5, Research institute for Endocrine, Sciences, Shahid Beheshti University	consumption of iodine during pregnancy	2016		Exclusion reason: Wrong intervention; iodine supplementation
IRCT20170513033941N33, Kashan University of Medical, Sciences	Effect of zinc supplementation in treatment of pregnant women at risk for intrauterine growth restriction	2018		Exclusion reason: Wrong patient population
IRCT2017060611341N5, Fasa University of Medical Sciences	effect of chamomile oil on decrease of headache due to spinal anesthesia in pregnant females	2017		Exclusion reason: Wrong intervention; chamomile oil
IRCT201708026888N19, Vice Chancellor for Research Technology, Hamadan University of Medical Sciences	The Effect of Ondansetron, Vitamin B6 and Rhizome in Nausea and Vomiting of Pregnancy in pregnant women referred to health centers in Hamadan city	2017		Exclusion reason: Wrong intervention
IRCT2017080635529N1, Deputy of Resaerch Tums	Efficacy evaluation of concurrent Zingiber and Vitamin B6 in morning sickness of pregnant women	2017		Exclusion reason: Wrong intervention

**Table 9:** Characteristics table of excluded studies (Database Searches)

Authors	Title	Published Year	Journal	Exclusion Reason
Islam, Md. Z., Shamim, A. A., Akhtaruzzaman, M., Kärkkäinen, M., Lamberg‐Allardt, C.	Effect of vitamin D, calcium and multiple micronutrients supplementation on lipid profile in pre‐menopausal Bangladeshi garment factory workers with hypovitaminosis D	2014	Journal of Health, Population & Nutrition	Exclusion reason: Wrong patient population
ISRCTN13890849, Protina Pharmazeutische GmbH	Does supplementation of extra Magnesium daily to first time pregnant healthy women prevent blood pressure increase during pregnancy?	2018		Exclusion reason: Wrong intervention
ISRCTN15485068 Khyber Medical University	The effect of oral high micro nutrient nutritional supplements on lipid profile in under weight hypertensive pregnant women	2018		Exclusion reason: Wrong patient population
ISRCTN18855042, Record provided by the N. H. S. Trusts Clinical Trials Register Department of Health	Effect of maternal vitamin C supplementation on plasma vitamin C levels in preterm infants during the early neonatal period	2003		Exclusion reason: Wrong intervention
ISRCTN19917787 University of Ulster	To determine whether taking folic acid during the later stages of pregnancy has any beneficial effects of the cognitive development and growth of the offspring	2013		Exclusion reason: Wrong patient population, study Location: Ireland;
ISRCTN23781770, Ottawa Hospital Research Institute	High Dose Folic Acid Supplementation Throughout Pregnancy for Preeclampsia Prevention	2010		Exclusion reason: Wrong patient population
ISRCTN32921044, King Edward Memorial Hospital Research Centre	Supplementation of vitamin B12 in young men and women, pre‐conception, improves the B12 status of their newborns	2012		Exclusion reason: Wrong patient population
ISRCTN37927591, University of Surrey	Selenium in pregnancy intervention	2013		Exclusion reason: Wrong intervention
ISRCTN68645785, Imperial College London	The effects of prenatal vitamin D supplementation on child health	2010		Exclusion reason: Wrong patient population, recruitment: UK
ISRCTN69794527, Commission of the European Communities	In vivo materno‐fetal fatty acid transfer in normal and obese pregnancy	2017		Exclusion reason: Wrong patient population, study Location: Spain and Germany
Jabbari, H., Bakhshian, F., Asgari, M., Sattari, M., Naghavi‐Behzad, M., Mashayekhi, S. O.	Antenatal micronutrient supplementation relationship with children's weight and height from birth up to the age of 18 months	2013	Iranian Journal of Public Health	Exclusion reason: Wrong study design
Jafarbegloo, E., Tehran, H. A., Tehrani, T. D.	Gastrointestinal complications of ferrous sulfate in pregnant women: a randomized double‐blind placebo‐controlled trial	2015	Iranian red crescent medical journal	Exclusion reason: Wrong outcomes
Jafari, F.	Maternal and neonatal outcomes of group prenatal care: A new experience in Iran	2010	Early Human Development	Exclusion reason: Wrong study design
Jaime‐Perez, J. C., Gomez‐Almaguer, D.	Iron stores in low‐income pregnant Mexican women at term	2002	Archives of Medical Research	Exclusion reason: Wrong intervention
Jamilian, M., Afshar, R.	Effects of combined evening primrose oil and vitamin D intake on hs‐CRP, oxidative stress and pregnancy outcomes in women with gestational diabetes	2017	Arak Medical University Journal	Exclusion reason: Wrong patient population
Janjua, N. Z., Delzell, E., Larson, R. R., Meleth, S., Kabagambe, E. K., Kristensen, S., Sathiakumar, N.	Maternal nutritional status during pregnancy and surma use determine cord lead levels in Karachi, Pakistan	2008	Environmental Research	Exclusion reason: Wrong intervention
Jarjou, Lm, Prentice, A, Bennett, J.	Impact of calcium supplementation in the preceding pregnancy on the human milk calcium concentration of Gambian women	2004	Advances in experimental medicine and biology	Exclusion reason: Wrong study design
Jefferds, M. E., Ogange, L., Owuor, M., Cruz, K., Person, B., Obure, A., Suchdev, P. S., Ruth, L. J.	Formative research exploring acceptability, utilization, and promotion in order to develop a micronutrient powder (Sprinkles) intervention among Luo families in western Kenya	2010	Food & Nutrition Bulletin	Exclusion reason: Wrong intervention
Jia Hai, X., Han, J,. Li, H., Liang, D., Deng, T., Chang, S.	Mineral intake in urban pregnant women from base diet, fortified foods, and food supplements: focus on calcium, iron, and zinc	2016	Biomedical and Environmental Sciences	Exclusion reason: Wrong intervention
Jiang, Y., Wang, H., Chen, J., Zhang, G., Chen, L., Dai, W., Zhou, W., Yang, H., Shi, H.	Blood lead levels during different trimesters of pregnancy and the possible influencing factors in Chengdu, China	2011	Biological Trace Element Research	Exclusion reason: Wrong intervention
Jin, J., Zou, Y., Zeng, S.	Risk factors for and expression of immune and inflammatory factors in atopic dermatitis in Chinese population: a birth cohort study	2016	Molecular and Cellular Probes	Exclusion reason: Wrong study design
Jing, H., Jahan, N., Schulze, K., Murray‐Kolb, L., Shamim, A. A., Labrique, A., Christian, P., West, K.	Gestational iodine deficiency, child cognition and motor skills At Age 5 years in rural Bangladesh	2011	FASEB Journal. Conference: Experimental Biology	Exclusion reason: Wrong intervention
Jirakittidul, P., Sirichotiyakul, S., Ruengorn, C., Techatraisak, K., Wiriyasirivaj, B.	Effect of iron supplementation during early pregnancy on the development of gestational hypertension and pre‐eclampsia	2018	Archives of Gynecology & Obstetrics	Exclusion reason: Wrong study design
Jooma, R.	Preventing neural tube defects by folic acid fortification of flour	2004	JPMA, Journal of the Pakistan Medical Association	Exclusion reason: Wrong study design
Joshi, K., Bhatia, V.	Vitamin D deficiency in a tropical country‐treatment and prevention in children	2014	Indian Journal of Pediatrics	Exclusion reason: Wrong study design
JPRN‐UMIN000008354, Oita University	Study on fetal oxidative stress reduction by a vitamin C supplement to pregnant women during intrapartum period	2012		Exclusion reason: Wrong intervention
Juma, M., Oiye, S. O., Konyole, S. O.	Predictors of optimum antenatal iron‐folate supplementation in a low resource rural set‐up in Eastern Kenya	2015	Journal of Public Health and Epidemiology	Exclusion reason: Wrong patient population
Jus'at, I., Achadi, E. L., Galloway, R., Dyanto, A., Zazri, A., Supratikto, G., Zizic, L., Elder, L.	Reaching young Indonesian women through marriage registries: an innovative approach for anaemia control	2000	Journal of Nutrition	Exclusion reason: Wrong outcomes
Kable, J. A., Coles, C. D., Chambers, C. D., Keen, C. L., Uriu‐Adams, J., Jones, K. L., Yevtushok, L., Wertelecki, W.	Preliminary analysis of the impact of micronutrient supplements on neurophysiological encoding and memory in Ukrainian infants 12‐18 months postpartum	2012	Birth Defects Research Part A ‐ Clinical and Molecular Teratology	Exclusion reason: Wrong patient population
Kable, J., Coles, C., Chambers, C., Keen, C., Uriu‐Adams, J., Jones, K., Yevtushok, L., Wertelecki, W.	The impact of micronutrient supplementation in alcohol‐exposed pregnancies on information processing skills in ukrainian infants	2013	Alcohol and Alcoholism	Exclusion reason: Wrong intervention
Kalengada, P. K., Maralusiddappa, P. G. C., Narayana, V. G. M., Srinath, M. K., Sivasambu, G.	Prevalence of Vitamin D deficiency in pregnancy and its association with Vitamin D levels in normal term newborns	2015	Perinatology	Exclusion reason: Wrong intervention
Kalipa, Z., Goon, D. T., Yako, E. M., Okeyo, A.	Factors influencing adherence to folic acid and ferrous sulphate nutritional intake among pregnant teenagers in buffalo city municipality, South Africa	2017	Pakistan Journal of Nutrition	Exclusion reason: Wrong intervention
Kalliokoski, P., Rodhe, N., Bergqvist, Y., Lofvander, M.	Long‐term adherence and effects on grip strength and upper leg performance of prescribed supplemental vitamin D in pregnant and recently pregnant women of Somali and Swedish birth with 25‐hydroxyvitamin D deficiency: A before‐and‐after treatment study	2016	BMC Pregnancy and Childbirth	Exclusion reason: Wrong patient population
Kanal, K., Busch‐Hallen, J., Cavalli‐Sforza, T., Crape, B., Smitasiri, S.	Weekly iron‐folic acid supplements to prevent anaemia among Cambodian women in three settings: process and outcomes of social marketing and community mobilization…Preventive weekly iron‐folic acid supplementation can improve iron status of reproductive age women: experience in Cambodia, the Philippines, and Vietnam. World Health Organization Western Pacific Region	2005	Nutrition Reviews	Exclusion reason: Wrong patient population
Kancherla, V., Ibne Hasan Md, O. S., Hamid, R., Paul, L., Selhub, J., Oakley, G., Quamruzzaman, Q., Mazumdar, M.	Prenatal folic acid use associated with decreased risk of myelomeningocele: A case‐control study offers further support for folic acid fortification in Bangladesh	2017	PLoS ONE	Exclusion reason: Wrong intervention
Kandasamy, V., Subramanian, M., Rajilarajendran, H., Ramanujam, S., Sathiya, S., Renuka, S.	A study on the incidence of neural tube defects in a tertiary care hospital over a period of five years	2015	Journal of Clinical and Diagnostic Research	Exclusion reason: Wrong study design
Kapil, U.	2nd International Workshop on Micronutrients and Child Health, New Delhi, India, 3‐7 November 2014	2014	Indian Journal of Community Health	Exclusion reason: Wrong study design
Kapil, U., Pathak, P., Tandon, M., Singh, C., Pradhan, R., Dwivedi, S. N.	Micronutrient deficiency disorders amongst pregnant women in three urban slum communities of Delhi	1999	Indian Pediatr	Exclusion reason: Wrong intervention
Kapil, U., Pradhan, R.	Integrated Child Development Services scheme (ICDS) and its impact on nutritional status of children in India and recent initiatives	1999	Indian Journal of Public Health	Exclusion reason: Wrong patient population
Karabulut, A., Åževket, O., Acun, A.	Iron, folate and vitamin B12 levels in first trimester pregnancies in the Southwest region of Turkey	2011	Journal of the Turkish‐German Gynecological Association	Exclusion reason: Wrong intervention
Karamali, M., Asemi, Z., Ahmadi‐Dastjerdi, M., Esmaillzadeh, A.	Calcium plus vitamin D supplementation affects pregnancy outcomes in gestational diabetes: randomized, double‐blind, placebo‐controlled trial	2016	Public Health Nutrition	Exclusion reason: Wrong patient population
Karamali, M., Heidarzadeh, Z., Seifati, S. M., Samimi, M., Tabassi, Z., Talaee, N., Bahardoost, H., Asemi, Z.	Zinc supplementation and the effects on pregnancy outcomes in gestational diabetes: a randomized, double‐blind, placebo‐controlled trial	2016	Experimental and Clinical Endocrinology and Diabetes	Exclusion reason: Wrong patient population
Karimi, B., Sabzi, R., Ghorbani, R.	Mothers ' practice about usage of iron supplement for infants and its related factors. [Persian]	2015	Koomesh	Exclusion reason: Wrong patient population
Karkee, R., Jha, N.	Primary health care development: where is Nepal after 30 years of Alma Ata Declaration?	2010	Jnma, Journal of the Nepal Medical Association	Exclusion reason: Wrong outcomes
Katre, P., Bhat, D., Lubree, H., Otiv, S., Joshi, S., Joglekar, C., Rush, E., Yajnik, C.	Vitamin B12 and folic acid supplementation and plasma total homocysteine concentrations in pregnant Indian women with low B12 and high folate status	2010	Asia Pacific Journal of Clinical Nutrition	Exclusion reason: Wrong intervention
Kavle, J., Landry, M., Gottwalt, A.	Community‐based distribution of iron‐folic acid supplementation: A review of evidence and program implications for anaemia programming for women and girls	2017	Annals of Nutrition and Metabolism	Exclusion reason: Wrong study design
Kawai, K., Spiegelman, D., Shankar, A. H., Fawzi, W. W.	Maternal multiple micronutrient supplementation and pregnancy outcomes in developing countries: meta‐analysis and meta‐regression	2011	Bulletin of the World Health Organization	Exclusion reason: Wrong study design
Kawuma, M.	Sugar as a potential vehicle for vitamin A fortification: experience from Kamuli district in Uganda	2002	African Health Sciences	Exclusion reason: Wrong intervention
Kehoe, S. H., Harsha, Chopra, Sahariah, S. A., Dattatray, Bhat, Munshi, R. P., Falguni, Panchal, Young, S., Brown, N., Dnyaneshwar, T., Meera, G., Margetts, B. M., Potdar, R. D., Fall, C. H. D.	Effects of a food‐based intervention on markers of micronutrient status among Indian women of low socio‐economic status	2015	British Journal of Nutrition	Exclusion reason: Wrong intervention
Khader, V., Maheswari, K. U.	Effect of feeding malted foods on the nutritional status of pregnant women, lactating women and preschool children in Lepakshi Mandal of Ananthapur district, Andhra Pradesh, India	2012	International Journal for Biotechnology and Molecular Biology Research	Exclusion reason: Wrong intervention
Khadivzadeh, T., Ghabel, M.	Use of complementary and alternative medicine in pregnancy, Mashad, Iran, 2005‐6	2009	International Journal of Gynecology and Obstetrics	Exclusion reason: Wrong intervention
Khan, A. I., Hawkesworth, S., Ekstrom, E. C., Shams, A., Moore, S. E., Frongillo, E. A., Yunus, M., Persson, L. A., Iqbal, K.	Effects of exclusive breastfeeding intervention on child growth and body composition: the MINIMat trial, Bangladesh	2013	Acta Paediatrica	Exclusion reason: Wrong intervention
Khan, M. H., Khalique, N., Khan, Z., Siddiqui, A. R.	Antenatal care scenario‐a study among pregnant women of peri‐urban area of Aligarh	2014	Indian Journal of Public Health Research and Development	Exclusion reason: Wrong intervention
Khanna, V.	Effect of calcium supplements on bone mineral density in rural population	2014	Osteoporosis International	Exclusion reason: Wrong patient population
Kharb, S.	Vitamin E and C in preeclampsia	2000	European Journal of Obstetrics Gynecology and Reproductive Biology	Exclusion reason: Wrong intervention
Khezri, M. B., Nasseh, N., Soltanian, G.	The comparative preemptive analgesic efficacy of addition of vitamin B complex to gabapentin versus gabapentin alone in women undergoing cesarean section under spinal anesthesia: a prospective randomized double‐blind study	2017	Medicine	Exclusion reason: Wrong intervention
Khor, G. L.	Update on the prevalence of malnutrition among children in Asia	2003	Nepal Med Coll J	Exclusion reason: Wrong study design
Khushboo, J., Najam, K., Athar, A., Anees, A., Haroon, K.	Effect of utilization of antenatal services on pregnancy outcome in Aligarh ‐ a community based study	2016	Indian Journal of Community Health	Exclusion reason: Wrong study design
Kidanto, H. L., Wangwe, P., Kilewo, C. D., Nystrom, L., Lindmark, G.	Improved quality of management of eclampsia patients through criteria based audit at Muhimbili National Hospital, Dar es Salaam, Tanzania. Bridging the quality gap	2012	BMC Pregnancy Childbirth	Exclusion reason: Wrong study design
Kiondo, P., Wamuyu‐Maina, G., Wandabwa, J., Bimenya, G. S., Tumwesigye, N. M., Okong, P.	The effects of vitamin C supplementation on pre‐eclampsia in Mulago Hospital, Kampala, Uganda: A randomized placebo controlled clinical trial	2014	BMC Pregnancy and Childbirth	Exclusion reason: Wrong intervention
Kirkwood, B.	Effect of vitamin A supplementation on maternal survival ‐ Authors' reply	2010	The Lancet	Exclusion reason: Wrong study design
Klemm, R. D. W., West Jr, K. P., Palmer, A. C., Johnson, Q., Randall, P., Ranum, P., Northrop‐Clewes, C.	Vitamin a fortification of wheat flour: Considerations and current recommendations	2010	Food and nutrition bulletin	Exclusion reason: Wrong study design
Kodentsova, V. M., Pogozheva, A. V., Gromova, O. A., Shikh, E. V.	Vitamin‐mineral supplements in nutrition of adults	2015	Voprosy Pitaniya	Exclusion reason: Wrong study design
Kodish, S., Shiff, C. J.	Formative research to understand household utilization of a lipid‐based nutrient supplement in rural Malawi and Mozambique	2015		Exclusion reason: Wrong study design
Koken, G. N., Derbent, A. U., Erol, O., Saygin, N., Ayik, H., Karaca, M.	Awareness and use of folic acid among reproductive age and pregnant women	2013	Journal of the Turkish German Gynecology Association	Exclusion reason: Wrong study design
Korenromp, E. L., Adeosun, O., Adegoke, F., Akerele, A., Anger, C., Ohajinwa, C., Hotz, C., Umunna, L., Aminu, F.	Micronutrient powder distribution through Maternal, Neonatal and Child Health Weeks in Nigeria: process evaluation of feasibility and use	2016	Public Health Nutrition	Exclusion reason: Wrong patient population
Kozuma, S.	Approaches to anaemia in pregnancy	2009	JMAJ Japan Medical Association Journal	Exclusion reason: Wrong study design
Kulkarni, S. S., Desai, A. D., Ranveer, R. C., Sahoo, A. K.	Development of nutrient rich noodles by supplementation with malted ragi flour	2012	International Food Research Journal	Exclusion reason: Wrong patient population
Kulkarni, V. V., Usha, Verghese	An overview of nutritional status of children in India	2014	Review of Research Journal	Exclusion reason: Wrong patient population
Kumbhar, A., Gupta, A., Thakur, H.	Practical strategies for anaemia prevention in registered antenatal women	2014	BJOG: An International Journal of Obstetrics and Gynaecology	Exclusion reason: Wrong intervention
Kumordzie, S., Arimond, M., Adu‐Afarwuah, S., Young, R. R., Ocansey, M. E., Okronipa, H., Prado, E., Oaks, B., Dewey, K. G.	The long‐term effect of maternal and early childhood supplementation on growth and body composition at 4‐6 years of age in Ghanaian children	2017	Annals of Nutrition and Metabolism	Exclusion reason: Wrong patient population
Kunsch, B. I., Stein, A. D., Villalpando, S., Martorell, R., Ramakrishnan, U.	Docosahexaenoic acid supplementation from mid‐pregnancy to parturition influenced breast milk fatty acid concentrations at 1 month postpartum in Mexican women	2011	Journal of Nutrition	Exclusion reason: Wrong intervention
Lachili, B., Hininger, I., Faure, H., Arnaud, J., Richard, M. J., Favier, A., Roussel, A. M.	Increased lipid peroxidation in pregnant women after iron and vitamin C supplementation	2001	Biological Trace Element Research	Exclusion reason: Wrong intervention
Lachowicz, J. I., Nurchi, V. M., Fanni, D., Gerosa, C., Peana, M., Zoroddu, M. A.	Nutritional iron deficiency: the role of oral iron supplementation	2014	Current Medicinal Chemistry	Exclusion reason: Wrong study design
Laillou, A., Panagides, D., Garrett, G. S., Moench‐Pfanner, R.	Vitamin A‐‐fortified vegetable oil exported from Malaysia and Indonesia can significantly contribute to vitamin A intake worldwide	2013	Food & Nutrition Bulletin	Exclusion reason: Wrong study design
Latham, Mc., Ash, D., Ndossi, G., Mehansho, H., Tatala, S.	Micronutrient dietary supplements‐‐a new fourth approach	2001	Archivos Latinoamericanos de Nutricion	Exclusion reason: Wrong patient population
Lawal, T. A., Adeleye, A. O.	Determinants of folic acid intake during preconception and in early pregnancy by mothers in Ibadan, Nigeria	2014	Pan African Medical Journal	Exclusion reason: Wrong intervention
Lawande, A., Gravio, C., Potdar, R. D., Sahariah, S. A., Gandhi, M., Chopra, H., Sane, H., Kehoe, S. H., Marley‐Zagar, E., Margetts, B. M., Jackson, A, A., Fall, C. H. D.	Effect of a micronutrient‐rich snack taken preconceptionally and throughout pregnancy on ultrasound measures of fetal growth: the Mumbai Maternal Nutrition Project (MMNP)	2017	Maternal & Child Nutrition	Exclusion reason: Wrong patient population
Lawande, A., Di Gravio, C., Potdar, R. D., Sahariah, S. A., Gandhi, M., Chopra, H., Sane, H., Kehoe, S. H., Marley‐Zagar, E., Margetts, B. M., Jackson, A. A., Fall, C. H. D.	Effect of a micronutrient‐rich snack taken preconceptionally and throughout pregnancy on ultrasound measures of fetal growth: The Mumbai Maternal Nutrition Project (MMNP)	2018	Maternal & Child Nutrition	Exclusion reason: Wrong patient population
Leyvraz, M., Laillou, A., Sabuktagin, R., Tahmeed, A., Rahman, A. S., Nurul, A., Santhia, I., Panagides, D.	An assessment of the potential impact of fortification of staples and condiments on micronutrient intake of young children and women of reproductive age in Bangladesh	2015	Nutrients	Exclusion reason: Wrong patient population
Li, C., Zhu, N., Zeng, L., Dang, S., Zhou, J., Kang, Y., Yang, Y., Yan, H.	Sex differences in the intellectual functioning of early school‐aged children in rural China	2016	BMC Public Health	Exclusion reason: Wrong patient population
Li, K., Wahlqvist, M. L., Li, D.	Nutrition, one‐carbon metabolism and neural tube defects: A review	2016	Nutrients	Exclusion reason: Wrong study design
Li, W., Qin, J., Rui, D.	Analysis on folate awareness and intake among reproductive aged women in Xinjiang production and construction corps	2011	Maternal and Child Health Care of China	Exclusion reason: Wrong intervention
Li, X., Jiang, J., Xu, M., Xu, M., Yang, Y., Lu, W., Yu, X., Ma, J., Pan, J.	Individualized supplementation of folic acid according to polymorphisms of methylenetetrahydrofolate reductase (MTHFR), methionine synthase reductase (MTRR) reduced pregnant complications	2015	Gynecologic and Obstetric Investigation	Exclusion reason: Wrong intervention
Li, X., Li, S., Mu, D., Liu, Z., Li, Y., Lin, Y., Chen, X., You, F., Li, N., Deng, K., Deng, Y., Wang, Y., Zhu, J.	The association between periconceptional folic acid supplementation and congenital heart defects: a case‐control study in China	2013	Preventive Medicine	Exclusion reason: Wrong intervention
Li, Z., Ren, A., Zhang, L., Ye, R., Li, S., Zheng, J., Hong, S., Wang, T., Li, Z.	Extremely high prevalence of neural tube defects in a 4‐county area in Shanxi Province, China	2006	Birth Defects Research	Exclusion reason: Wrong study design
Liang, Q., Gong, W., Zheng, D., Zhong, R., Wen, Y., Wang, X.	The influence of maternal exposure history to virus and medicine during pregnancy on congenital heart defects of fetus	2017	Environmental science and pollution research international	Exclusion reason: Wrong intervention
Liao, Y., Wang, J., Li, X., Guo, Y., Zheng, X.	Identifying environmental risk factors for human neural tube defects before and after folic acid supplementation	2009	BMC public health	Exclusion reason: Wrong intervention
Lin, Q., Li, F., Hu, X. Y., Deng, J., Shi, J. C., Gong, W. J.	Awareness, knowledge and behavior of using folic acid supplementation to prevent NTDS among rural women in central south china	2013	Annals of Nutrition and Metabolism	Exclusion reason: Wrong intervention
Lin, W., Ma, S., Yang, L.	Effect of folic acid and vitamin supplement on endothelial injury and placental blood perfusion in patients with hypertensive disorder complicating pregnancy	2017	Journal of Hainan Medical University	Exclusion reason: Wrong patient population
Lin, Y., Shu, S., Tang, S.	A case‐control study of environmental exposures for nonsyndromic cleft of the lip and/or palate in eastern Guangdong, China	2014	International Journal of Pediatric Otorhinolaryngology	Exclusion reason: Wrong intervention
Lindström, E, Hossain, M. B., Lönnerdal, B., Raqib, R., Arifeen, S., Ekström, E.‐C.	Prevalence of anaemia and micronutrient deficiencies in early pregnancy in rural Bangladesh, the MINIMat trial	2011	Acta Obstetricia et Gynecologica Scandinavica	Exclusion reason: Wrong study design; Baseline data only
Linhares, A. O., Cesar, J. A.	Folic acid supplementation among pregnant women in southern Brazil: prevalence and factors associated	2017	Ciencia & Saude Coletiva	Exclusion reason: Wrong outcomes
Lips, P.	Worldwide status of vitamin D nutrition	2010	Journal of steroid biochemistry and molecular biology	Exclusion reason: Wrong study design
Lipus, A., Stanhope, K., De Aceituno, A. F., Rebolledo, P. A., Srinath, M., Revollo, R., Iniguez, V., Suchdev, P., Leon, J. S.	Micronutrient supplementation during pregnancy and anaemia in the post‐partum period among women in Bolivia's Andean plateau	2014	American Journal of Tropical Medicine and Hygiene	Exclusion reason: Wrong intervention
Liu, J., Gao, L.,; Zhang, Y., Jin, L., Li, Z., Zhang, L., Meng, Q., Ye, R., Wang, L., Ren, A.	Plasma folate levels in early to mid pregnancy after a nation‐wide folic acid supplementation program in areas with high and low prevalence of neural tube defects in China	2015	Birth Defects Research	Exclusion reason: Wrong intervention
Liu, J., Zhang, L., Li, Z., Jin, L., Zhang, Y., Ye, R., Liu, J., Ren, A.	Prevalence and trend of neural tube defects in five counties in Shanxi province of Northern China, 2000 to 2014	2016	Birth Defects Research	Exclusion reason: Wrong study design
Liu, J., Wang, Q., Gao, F., He, J., Zhao, J.	Maternal antenatal administration of vitamin K1 results in increasing the activities of vitamin K‐dependent coagulation factors in umbilical blood and in decreasing the incidence rate of periventricular‐intraventricular hemorrhage in premature infants	2006	Journal of Perinatal Medicine	Exclusion reason: Wrong intervention
Liu, J., Jin, L., Meng, Q., Gao, L., Zhang, L., Li, Z., Ren, A.	Changes in folic acid supplementation behaviour among women of reproductive age after the implementation of a massive supplementation programme in China	2015	Public Health Nutrition	Exclusion reason: Wrong study design
Liu, M., Chen, J., Liu, J., Zhang, S., Wang, Q., Shen, H., Zhang, Y.	Socioeconomic inequality in periconceptional folic acid supplementation in China: A census of 0.9 million women in their first trimester of pregnancy	2017	BMC Pregnancy and Childbirth	Exclusion reason: Wrong study design

**Table 10:** Characteristics table of excluded studies (Database Searches)

Authors	Title	Published Year	Journal	Exclusion Reason
Liu, Q., Hu, Y., Sun, Q.	Investigation on antenatal care and pregnant outcomes of 26 803 women in Jiangsu	2009	Maternal and Child Health Care of China	Exclusion reason: Wrong intervention
Loffredo, L. C. M., Souza, J. M. P., Freitas, J. A. S., Mossey, P. A.	Oral clefts and vitamin supplementation	2001	Cleft Palate Craniofacial Journal	Exclusion reason: Wrong study design
Lopes, R. E., Ramos, K. da S., Bressani, C. C., de Arruda, I. K., de Souza, A. I.	Anemia and hypovitaminosis in postpartum women seen at the Women's Care Center of the Instituto Materno Infantil Prof. Fernando Figueira, IMIP: a pilot study	2006	Revista Brasileira de Saude Materno Infantil	Exclusion reason: Wrong patient population
Lopez‐Jaramillo, P., Casas, J. P., Serrano, N.	Preeclampsia: from epidemiological observations to molecular mechanisms	2001	Brazilian Journal of Medical and Biological Research	Exclusion reason: Wrong study design
Loudyi, F. M., Kassouati, J.,; Kabiri, M., Chahid, N., Kharbach, A., Aguenaou, H., Barkat, A.	Vitamin D status in moroccan pregnant women and newborns: Reports of 102 cases	2016	Pan African Medical Journal	Exclusion reason: Wrong study design
Lozanov, B., Tzachev, K., Kirilov, E., Aceva, E., Vukov, M.	Serum selenium concentration and thyroid status in pregnant women on the background of systemic iodine supplementation. [Article in Bulgarian]	2008	Endokrinologya	Exclusion reason: Wrong intervention
Lozoff, B.	Iron deficiency and child development	2007	Food & Nutrition Bulletin	Exclusion reason: Wrong patient population
Lu, W., Lu, M., Li, Z., Zhang, C.	Effects of multimicronutrient supplementation during pregnancy on postnatal growth of children under 5 years of age: a meta‐analysis of randomized controlled trials	2014	PLoS ONE [Electronic Resource]	Exclusion reason: Wrong study design
Lucca, L., de., Rodrigues, F., Jantsch, L. B., Neme, W. S., Gallarreta, F. M. P., Goncalves, T. L.	Oxidative profile and delta ‐aminolevulinate dehydratase activity in healthy pregnant women with iron supplementation	2016	International journal of environmental research and public health	Exclusion reason: Wrong outcomes
LuHang, Xu, Y., Wu, Y.	Comprehensive evaluation of primary prevention of birth defects among rural pregnant women in Sichuan Province	2013	Maternal and Child Health Care of China	Exclusion reason: Wrong intervention
Lukose, A., Ramthal, A., Thomas, T., Bosch, R., Kurpad, A. V., Duggan, C., Srinivasan, K.	Nutritional factors associated with antenatal depressive symptoms in the early stage of pregnancy among urban South Indian women	2014	Maternal & Child Health Journal	Exclusion reason: Wrong intervention
Lunardi‐Maia, T., Schuelter‐Trevisol, F., Galato, D.	Medication use during the first trimester of pregnancy: drug safety and adoption of folic acid and ferrous sulphate	2014	Revista Brasileira de Ginecologia e Obstetricia	Exclusion reason: Wrong study design
Lutovac, B., Duborija‐Kovacevic, N., Pravilovic, D., Lopicic, B.	The influence of education level on self‐medication and medicine consumption among pregnant women in montenegro	2011	Basic and Clinical Pharmacology and Toxicology	Exclusion reason: Wrong intervention
Lynch, S. R.	The potential impact of iron supplementation during adolescence on iron status in pregnancy	2000	Journal of Nutrition	Exclusion reason: Wrong study design
Ma, A., Chen, X., Wang, Y., Xu, R., Zheng, M., Li, J.	The multiple vitamin status of Chinese pregnant women with anaemia and nonanaemia in the last trimester	2004	Journal of Nutritional Science and Vitaminology	Exclusion reason: Wrong intervention
MacDonald, A. C., Main, B. J., Namarika, R. H., Yiannakis, M. E., Mildon, A. M., Thompson, B., Amoroso, L.	Small‐animal revolving funds: an innovative programming model to increase access to and consumption of animal‐source foods by rural households in Malawi	2011		Exclusion reason: Wrong intervention
Maged, A., Elsherbini, M., Ramadan, W., Elkomy, R., Helal, O., Hatem, D., Fouad, M., Gaafar, H.	Periconceptional risk factors of spina bifida among Egyptian population: a case‐control study	2016	J Matern Fetal Neonatal Med	Exclusion reason: Wrong intervention
Mahajan, N. N., Mahajan, K. N., Soni, R. N., Gaikwad, N. L.	Justifying the "Folate trap" in folic acid fortification programs	2007	Journal of Perinatal Medicine	Exclusion reason: Wrong study design
Mahmoud, E. R., Ahmed, A. F.,; Abo‐Elfotoh, N. M., Mohamed, E. A., Al‐Hendy, A.	Maternal vitamin D supplementation decreases risk of rickets in children but effect is modified by parity, a case‐control study in Southern Egypt	2011	Reproductive Sciences	Exclusion reason: Wrong intervention
Mahomed, K.	Iron supplementation in pregnancy	2000	Cochrane Database of Systematic Reviews	Exclusion reason: Wrong study design
Mahomed, K.	WITHDRAWN: Iron supplementation in pregnancy	2007	Cochrane Database of Systematic Reviews	Exclusion reason: Wrong study design
Maleki, A., Fard, M. K., Zadeh, D. H., Mamegani, M. A., Abasaizadeh, S., Mazloomzadeh, S.	The relationship between plasma level of Se and preeclampsia	2011	Hypertension in Pregnancy	Exclusion reason: Wrong study design
Maleta, K. M., Kaimila, Y.	Impact of Interventions to Improve Prenatal Nutrition in Developing Countries on Maternal Health: Obstetric Outcomes and Fetal Health	2015	Current Nutrition Reports	Exclusion reason: Wrong study design
Malpeli, A., Apezteguia, M., Mansur, J. L., Armanini, A., Macias Couret, M., Villalobos, R., Kuzminczuk, M., Gonzalez, H. F.	Calcium supplementation, bone mineral density and bone mineral content. Predictors of bone mass changes in adolescent mothers during the 6‐month postpartum period	2012	Archivos Latinoamericanos de Nutricion	Exclusion reason: Wrong patient population
Mamta, S., Shashi, J.	Efficacy assessment of food based iron intervention on hematological parameters of pregnant women	2013	Asian Journal of Dairy and Food Research	Exclusion reason: Wrong patient population
Manisha, N., Vartika, S., Mirza, A. A., Ranjeeta, K., Kapil, S., Jyoti, B	Assessment of folic acid supplementation in pregnant women by estimation of serum levels of tetrahydrofolic acid, dihydrofolate reductase, and homocysteine	2016	Scientifica	Exclusion reason: Wrong intervention
Mansourian, M., Mohammadi, R., Marateb, H. R., Yazdani, A., Goodarzi‐Khoigani, M., Molavi, S.	Comprehensive maternal characteristics associated with birth weight: Bayesian modeling in a prospective cohort study from Iran	2017	Journal of Research in Medical Sciences	Exclusion reason: Wrong study design
Mao, B., Qiu, J., Zhao, N., Shao, Y., Dai, W., He, X., Cui, H., Lin, X., Lv, L., Tang, Z., Xu, S., Huang, H., Zhou, M., Xu, X., Qiu, W., Liu, Q., Zhang, Y.	Maternal folic acid supplementation and dietary folate intake and congenital heart defects	2017	PLoS ONE	Exclusion reason: Wrong intervention
Mao, B., Qiu, W., Wang, Y.	Study on the correlation between folic acid supplementation before and during pregnancy and hypertensive disorder complicating pregnancy	2017	Maternal and Child Health Care of China	Exclusion reason: Wrong study design
Marchetta, C., Hamner, H.	Blood folate concentrations among women of childbearing age by race/ethnicity and acculturation, NHANES 2001‐2010	2014	FASEB Journal. Conference: Experimental Biology	Exclusion reason: Wrong patient population
Margetts, B. M., Fall, C. H. D., Ronsmans, C., Allen, L. H., Fisher, D. J.	Multiple micronutrient supplementation during pregnancy in low‐income countries: review of methods and characteristics of studies included in the meta‐analyses. (Special Issue: Multiple micronutrient supplementation during pregnancy in developing country settings.)	2009	Food and nutrition bulletin	Exclusion reason: Wrong intervention
Marti‐Carvajal, A., Pena‐Marti, G., Comunian, G., Munoz, S.	Prevalence of anaemia during pregnancy: results of Valencia (Venezuela) Anemia During Pregnancy Study	2002	Archivos Latinoamericanos de Nutricion	Exclusion reason: Wrong intervention
Martin, S. L., Omotayo, M. O., Chapleau, G. M., Stoltzfus, R. J., Birhanu, Z., Ortolano, S. E., Pelto, G. H., Dickin, K. L.	Adherence partners are an acceptable behaviour change strategy to support calcium and iron‐folic acid supplementation among pregnant women in Ethiopia and Kenya	2017	Maternal and Child Nutrition	Exclusion reason: Wrong outcomes
Martin, S., Omotayo, M., Chapleau, G., Stoltzfus, R., Birhanu, Z., Ortolano, S., Dickin, K.	Cross‐country comparison of the acceptability of a social support behavior change strategy to improve adherence to antenatal calcium and iron‐folic acid supplementation	2016	FASEB Journal. Conference: Experimental Biology	Exclusion reason: Wrong study design
Martin, Stephanie L., Stoltzfus, Rebecca J., Ortolano, Stephanie E., Dickin, Katherine L., Omotayo, Moshood O., Mwanga, E.	With adaptation, the WHO guidelines on calcium supplementation for prevention of pre‐eclampsia are adopted by pregnant women	2018	Maternal & Child Nutrition	Exclusion reason: Wrong study design
Martinez de Villarreal, L. E., Arredondo, P., Hernandez, R., Villarreal, J. Z.	Weekly administration of folic acid and epidemiology of neural tube defects	2006	Maternal & Child Health Journal	Exclusion reason: Wrong study design
Martinez de Villarreal, L., Villarreal Perez, J. Z., Arredondo Vazquez, P., Hernandez Herrera, R., Velazco Campos, M. del R., Ambriz Lopez, R., Herrera Ramirez, J. L., Yanez Sanchez, J. M., Morales Villarreal, J. J., Trevino Garza, M., Limon, A., Guzman Lopez, A., Barcenas, M., Cepeda Garcia, J. R., Sanchez Dominguez, A., Hernandez Nunez, R., Garcia Ayala, J. L., Garza Martinez, J., Tijerina Gonzalez, M., Garcia Alvarez, C., Negrete Castro, R.	Decline of neural tube defects cases after a folic acid campaign in Nuevo Leon, Mexico	2002	Teratology	Exclusion reason: Wrong study design
Matias, S. L., Mridha, M. K., Fahmida, Tofail, Arnold, C. D., Khan, M. S. A., Zakia, Siddiqui, Ullah, M. B., Dewey, K. G.	Home fortification during the first 1000 d improves child development in Bangladesh: a cluster‐randomized effectiveness trial	2017	American Journal of Clinical Nutrition	Exclusion reason: Wrong patient population
McAlpine, J. M., Scott, R., Scuffham, P. A., Perkins, A. V., Vanderlelie, J. J.	The association between third trimester multivitamin/mineral supplements and gestational length in uncomplicated pregnancies	2016	Women & Birth: Journal of the Australian College of Midwives	Exclusion reason: Wrong study design
McAuley, E., McNulty, H., Hughes, C., Strain, J. J., Ward, M.	Riboflavin status, MTHFR genotype and blood pressure: current evidence and implications for personalised nutrition	2016	Proceedings of the Nutrition Society	Exclusion reason: Wrong study design
McCauley, M. E., van den Broek, N., Dou, L., Othman, M.	Vitamin A supplementation during pregnancy for maternal and newborn outcomes	2015	Cochrane Database of Systematic Reviews	Exclusion reason: Wrong study design
McGready, R., Simpson, J. A., Cho, T., Dubowitz, L., Changbumrung, S., Bohm, V., Munger, R. G., Sauberlich, H. E., White, N. J., Nosten, F.	Postpartum thiamine deficiency in a Karen displaced population	2001	American Journal of Clinical Nutrition	Exclusion reason: Wrong intervention
McKinney, C. M., Chowchuen, B., Pitiphat, W., DeRouen, T., Pisek, A., Godfrey, K.	Micronutrients and oral clefts: a case‐control study	2013	Journal of Dental Research	Exclusion reason: Wrong study design
Megazzini, K. M., Chintu, N, Vermund, S. H., Redden, D. T., Krebs, D. W., Simwenda, M., Tambatamba, B. Sinkala, M, Stringer, J. S.	Predictors of rapid HIV testing acceptance and successful nevirapine administration in Zambian labor wards	2009	Journal of acquired immune deficiency syndromes (1999)	Exclusion reason: Wrong patient population
Mehansho, H.	Iron fortification technology development: new approaches	2006	The Journal of Nutrition	Exclusion reason: Wrong study design
Mehta, S., Dwarkanath, P., Finkelstein, J. L., Joseph, S., Thomas, T., Kurpad, A. V.	High burden of vitamin D deficiency in pregnancy and its association with low birth weight in a Prospective Cohort in South India	2016	FASEB Journal. Conference: Experimental Biology	Exclusion reason: Wrong intervention
Meijer, W. M., de Walle, H. E. K.	Differences in folic‐acid policy and the prevalence of neural‐tube defects in Europe; recommendations for food fortification in a EUROCAT report	2005	Nederlands Tijdschrift voor Geneeskunde	Exclusion reason: Wrong study design
Mello Neto, J., Rondo, P. H. C., Oshiiwa, M., Morgano, M. A., Zacari, C. Z., dos Santos, M. L.	Iron supplementation in pregnancy and breastfeeding and iron, copper and zinc status of lactating women from a human milk bank	2013	Journal of Tropical Pediatrics	Exclusion reason: Wrong study design
Melse‐Boonstra, A., Gowachirapant, S., Nidhi, J., Winichagoon, P., Krishnamachari, S., Zimmermann, M. B.	Iodine supplementation in pregnancy and its effect on child cognition	2012	Journal of trace elements in medicine and biology	Exclusion reason: Wrong study design
Mendu, V., Athe, R., Ku, R. K., M. N., K.	Maternal nutrition and adverse birth outcomes in developing countries: A systematic review and meta‐analysis	2013	Annals of Nutrition and Metabolism	Exclusion reason: Wrong study design
Mensa‐Wilmot, Y., Phillips, R. D., Sefa‐Dedeh, S.	Acceptability of extrusion cooked cereal/legume weaning food supplements to Ghanaian mothers	2001	International journal of food sciences and nutrition	Exclusion reason: Wrong patient population
Merialdi, M., Caulfield, L. E., Zavaleta, N., Figueroa, A., DiPietro, J. A.	Adding zinc to prenatal iron and folate tablets improves fetal neurobehavioral development	1999	American Journal of Obstetrics and Gynecology	Exclusion reason: Wrong outcomes
Merialdi, M., Caulfield, L. E., Zavaleta, N., Figueroa, A., Dominici, F., DiPietro, J. A.	Randomized controlled trial of prenatal zinc supplementation and the development of fetal heart rate	2004	American Journal of Obstetrics and Gynecology	Exclusion reason: Wrong outcomes
Metzgeroth, G., Hastka, J.	Iron deficiency anaemia and anaemia of chronic disorders. [Article in German]	2015	Internist	Exclusion reason: Wrong study design
Mian, C., Vitaliano, P., Pozza, D., Barollo, S., Pitton, M., Callegari, G., di Gianantonio, E.,; Casaro, A., Acamulli, D. N., Busnardo, B., Mantero, F., Girelli, M. E.	Iodine status in pregnancy: role of dietary habits and geographical origin	2009	Clinical Endocrinology	Exclusion reason: Wrong study design
Middleton, P. F., Lassi, Z. S., Tran, T. S., Bhutta, Z., Bubner, T. K., Flenady, V., Crowther, C. A.	Nutrition interventions and programs for reducing mortality and morbidity in pregnant and lactating women and women of reproductive age: A systematic review	2013	Journal of Paediatrics and Child Health	Exclusion reason: Wrong study design
Miller, M.,; Humphrey, J., Johnson, E., Marinda, E., Brookmeyer, R., Katz, J.	Why do children become vitamin A deficient?	2002	Journal of Nutrition	Exclusion reason: Wrong intervention
Miller, M. A. F., Humphrey, J. H.	Vitamin A and anaemia in Zimbabwean infants	2002		Exclusion reason: Wrong intervention
Mireku, M. O., Cot, M., Bodeau‐Livinec, F.	The impact of anaemia during pregnancy and its risk factors on the cognitive development of one‐year‐old children	2016	American Journal of Tropical Medicine and Hygiene	Exclusion reason: Wrong study design
Mireku, M. O., Davidson, L. L., Boivin, M. J., Zoumenou, R., Massougbodji, A., Cot, M., Bodeau‐Livinec, F.	Prenatal iron deficiency, neonatal ferritin, and infant cognitive function	2016	Pediatrics	Exclusion reason: Wrong intervention
Mishra, V., Thapa, S., Retherford, R. D., Dai, X. L.	Effect of iron supplementation during pregnancy on birthweight: evidence from Zimbabwe	2005	Food and nutrition bulletin	Exclusion reason: Wrong intervention
Modjadji, S. E. P., Alberts, M., Mamabolo, R. L.	Folate and iron status of South African non‐pregnant rural women of childbearing, age, before and after fortification of foods	2007	South African Journal of Clinical Nutrition	Exclusion reason: Wrong patient population
Moghadam Banaem, L., Aliyan Moghadam, N.	Calcium supplementation in pregnancy and neonatal outcomes	2011	Iranian Journal of Reproductive Medicine	Exclusion reason: Wrong study design
Molloy, A. M., Kirke, P. N., Brody, L. C., Scott, J. M., Mills, J. L.	Effects of folate and vitamin B12 deficiencies during pregnancy on fetal, infant, and child development	2008	Food and nutrition bulletin	Exclusion reason: Wrong study design
Monalisha, R., Ray, I., Parnamita, B., Somajita, C.	Estimation of serum vitamin D levels at term pregnancy in a tertiary care centre in eastern India ‐ a cross‐sectional study	2017	Journal of Evolution of Medical and Dental Sciences	Exclusion reason: Wrong study design
Monoarfa, Y., Otoluwa, A. S., Widasari, L., Said, R., Habib, H., Handajani, R., Kuntoro, K., Gumilar, E., Wirjatmadi, B., Thaha, A. R.	Acceptance of and compliance with multi‐micronutrient and iron‐folic acid capsules in Banggai District, Indonesia	2017	Annals of Nutrition and Metabolism	Exclusion reason: Wrong outcomes
Mora, J. O.	Iron supplementation: overcoming technical and practical barriers	2002	Journal of Nutrition	Exclusion reason: Wrong outcomes
Mosha, D., Liu, E., Hertzmark, E., Chan, G., Sudfeld, C., Masanja, H., Fawzi, W.	Dietary iron and calcium intakes during pregnancy are associated with lower risk of prematurity, stillbirth and neonatal mortality among women in Tanzania	2017	Public Health Nutrition	Exclusion reason: Wrong study design
Mosha, D., Mazuguni, F., Mrema, S., Abdulla, S., Genton, B.	Medication exposure during pregnancy: a pilot pharmacovigilance system using health and demographic surveillance platform	2014	BMC pregnancy and childbirth	Exclusion reason: Wrong study design
Mostert, D., Steyn, N. P., Temple, N. J., Olwagen, R.	Dietary intake of pregnant women and their infants in a poor black South African community	2005	Curationis	Exclusion reason: Wrong study design
Moulessehoul, S., Demmouche, A., Chafi, Y., Benali, M.	Effect of iron supplementation among pregnant women at mother‐and‐baby clinic of Sidi Bel Abbes, West Algeria. [Article in French]	2004	Cahiers Sante	Exclusion reason: Wrong patient population
Mousavi, M., Heidari, E., Rayman, M. P., Tara, F., Boskabadi, H., Mohammadi, S., Maamouri, G., Tavallaie, S., Shakeri, M. T., Ghayour‐Mobarhan, M., Ferns, G.	Effects of selenium supplementation on soluble FMS‐like tyrosine kinase‐1 and glutathione peroxidase levels and the plasminogen activator inhibitor‐1: plasminogen activator inhibitor‐2 ratio in pregnant women	2015	Shiraz E Medical Journal	Exclusion reason: Wrong patient population
Moya‐Alvarez, V., Cottrell, G., Ouedraogo, S., Accrombessi, M., Massobodgi, A., Cot, M.	High folate levels are not associated to increased risk of malaria but to reduced anaemia rates in the context of high dosed folate supplements and spiptp in Benin	2015	American Journal of Tropical Medicine and Hygiene	Exclusion reason: Wrong study design
Moya‐Alvarez, V., Ouedraogo, S., Accrombessi, M., Cot, M.	High folate levels are not associated with increased malaria risk but with reduced anaemia rates in the context of high‐dosed folate supplements and intermittent preventive treatment against malaria in pregnancy with sulphadoxine‐pyrimethamine in Benin	2018	Tropical Medicine and International Health	Exclusion reason: Wrong patient population
Muhihi, A., Sudfeld, C. R., Smith, E. R., Noor, R. A., Mshamu, S., Briegleb, C., Bakari, M., Masanja, H., Fawzi, W., Chan, G. J.	Risk factors for small‐for‐gestational‐age and preterm births among 19,269 Tanzanian newborns	2016	BMC Pregnancy & Childbirth	Exclusion reason: Wrong patient population
Mujawar, J. R., Patel, S. S.	Circulating biomarkers of oxidative stress in preeclampsia and efficacy of antioxidant Vitamin C supplementation	2016	Research Journal of Pharmaceutical, Biological and Chemical Sciences	Exclusion reason: Wrong patient population
Muller, O., Krawinkel, M.	Malnutrition and health in developing countries	2005	Canadian Medical Association Journal	Exclusion reason: Wrong study design
Muller, S. D.	Zinc deficiency, part 1. Causes, symptoms therapy. [Article in German]	2003	Schweizerische Zeitschrift fur GanzheitsMedizin	Exclusion reason: Wrong study design
Muller, S. D.	Zinc deficiency, part 2. Causes, symptoms, therapy. [Article in German]	2004	Schweizerische Zeitschrift fur GanzheitsMedizin	Exclusion reason: Wrong study design
Muthayya, S.	Maternal nutrition & low birth weight ‐ what is really important?	2009	Indian Journal of Medical Research	Exclusion reason: Wrong study design
Muthayya, S., Kurpad, A. V., Duggan, C. P., Bosch, R. J., Dwarkanath, P., Mhaskar, A., Mhaskar, R., Thomas, A., Vaz, M., Bhat, S., Fawzi, W. W.	Low maternal vitamin B12 status is associated with intrauterine growth retardation in urban South Indians	2006	European Journal of Clinical Nutrition	Exclusion reason: Wrong intervention
Mwangi, M. N., Andang'o, P. E. A., Mwangi, A. M., Verhoef, H., Savelkoul, H. F. J.	Safety and efficacy of fortification versus fortification plus supplementation with iron in african pregnant women: A randomised controlled trial	2013	Annals of Nutrition and Metabolism	Exclusion reason: Wrong intervention
Nadafi, M., Momeni, E., Hosseini, S. M., Hosseini, M., Zoladl, M.	The association of homocysteine and vitamins with preeclampsia in pregnancy	2009	Atherosclerosis. Conference: 15th International Symposium on Atherosclerosis. Boston, MA United States. Conference Publication:	Exclusion reason: Wrong study design
Nainar, A. T. S. A., Malarvizhi, L.	Undiluted iron sucrose administration in pregnant women with iron deficiency anaemia	2017	Journal of Evolution of Medical and Dental Sciences	Exclusion reason: Wrong patient population
Nair, K. M.	Alternate strategies for improving iron nutrition: lessons from recent research. (Micronutrients, maternal and child health)	2001	British Journal of Nutrition	Exclusion reason: Wrong study design
NCT	Bovine Lactoferrin Versus Ferrous Sulphate In The Treatment Of Iron Deficiency Anemia During Pregnancy	2015	https://clinicaltrials.gov/show/nct03202615	Exclusion reason: Wrong patient population
NCT	Efficacy of Vitamin A Fortified Rice in Lactating Thai Women	2017	https://clinicaltrials.gov/show/nct03056625	Exclusion reason: Wrong intervention
NCT	Interventions for Moderate Malnutrition in Pregnancy	2014	https://clinicaltrials.gov/show/nct02120599	Exclusion reason: Wrong patient population
NCT00000543, National Heart, Lung and Blood Institute	Oral Calcium in Pregnant Women With Hypertension	1999		Exclusion reason: Wrong patient population
NCT00135902, The George Washington University Biostatistics Center, Eunice Kennedy Shriver National Institute of Child, Health and Human, Development	Omega‐3 Fatty Acid Supplementation to Prevent Preterm Birth in High Risk Pregnancies	2005		Exclusion reason: Wrong patient population. Study Location: US
NCT00148629, Cornell, University, Bill and Melinda Gates Foundation, Unicef, Johns Hopkins Bloomberg School of Public Health	Treatment and Prevention of Severe Anemia in Pregnant Zanzibari Women	2005		Exclusion reason: Wrong patient population
NCT00167700, University of Turku, Academy of Finland	Pre‐, Peri‐ and Postnatal Programming and Origins of Disease: Early Targeting the Epidemics of Allergy and Overweight	2005		Exclusion reason: Wrong intervention
NCT00292591, Medical University of South Carolina, Eunice Kennedy Shriver National Institute of Child Health and Human Development	Evaluation of Vitamin D Requirements During Pregnancy	2006		Exclusion reason: Wrong patient population,Study Location: US
NCT00300937, Teran, E. M. D., Ecuadorian Foundation for Science and Technology, FUNDACYT, Central University of Ecuador, Jarrow Formulas Inc	Coenzyme Q10 Supplementation and Development of Preeclampsia	2006		Exclusion reason: Wrong intervention
NCT00320125, University of Utah, National Dairy Council, Rosemont Illinois USA	Effects of Dairy Foods on Adolescent Pregnant Mothers and Their Newborns	2006		Exclusion reason: Wrong patient population, Study location: US, Intervention: Fortification,
NCT00332124, University of Colorado Denver	Safety and Effectiveness of Taking Choline Supplements During Pregnancy for Improving Infant Brain Development	2006		Exclusion reason: Wrong patient population
NCT00362089, Technische Universität München, Else Kröner‐Fresenius Foundation, International, Unilever‐Foundation, Danone, Research, E. U. funding by EARNEST consortium, German Ministry of, Education Research, Gf‐Gfgi	Fatty Acids During Pregnancy and Lactation and Body Fat Mass in Newborns	2006		Exclusion reason: Wrong patient population,Study Location: Germany
NCT00388856, Showa, University, Indonesia University	Antioxidant Supplementation in Pregnant Women With Low Antioxidant Status	2006		Exclusion reason: Wrong patient population
NCT00558623, National Institute of Environmental Health Sciences, Mexican National Institute of Public Health, Brigham and Women's, Hospital, University of California	Dietary Calcium Supplementation to Reduce Blood Lead in Pregnancy	2007		Exclusion reason: Wrong outcomes
NCT00610688, Cincinnati Children's Hospital Medical Center,United Arab Emirates University, Thrasher Research Fund	Controlled Trial of Prenatal Vitamin D3 Supplementation to Prevent Vitamin D Deficiency in Mothers and Their Infants	2007		Exclusion reason: Wrong patient population, Study Location: US and UAE

**Table 11:** Characteristics table of excluded studies (Database Searches)

Authors	Title	Published Year	Journal	Exclusion Reason
NCT00620672, University of British, Columbia, Canadian Institutes of Health Research	N‐3 Fatty Acid Requirements for Human Development	2008		Exclusion reason: Wrong patient population, Study Location: Canada
NCT00632476, Oregon Health & Science University, National Heart, Lung and Blood Institute, National Institute of Environmental Health Sciences	Evaluating the Effects of Supplemental Vitamin C on Infant Lung Function in Pregnant Smoking Women	2008		Exclusion reason: Wrong intervention
NCT00742937, Instituto Materno Infantil, Fernando, F.	Impact of Maternal Supplementation With Dual Megadose of Vitamin A	2008		Exclusion reason: Wrong patient population, Post‐partum supplementation with Vit A and E
NCT00789490, University of Toronto, The Hospital for Sick Children, H. J. Heinz Foundation	Relative Bioavailability of Iron and Folic Acid in New Test Supplement	2008		Exclusion reason: Wrong patient population, Study location: Canada
NCT00865683, National Heart, Lung and Blood Institute, Office of Research on Women's, Health, Mead Johnson, Nutrition, Dsm Nutritional Products, Inc	DHA Supplements to Improve Insulin Sensitivity in Obese Pregnant Women (The Omega‐3 Pregnancy Study)	2009		Exclusion reason: Wrong patient population
NCT00894920, University of Arkansas	Biotin Status in Pregnancy	2009		Exclusion reason: Wrong patient population, Study Location: US
NCT00920621, Brigham and Women's, Hospital, National Heart, Lung and Blood Institute	Randomized Trial: Maternal Vitamin D Supplementation to Prevent Childhood Asthma (VDAART)	2009		Exclusion reason: Wrong patient population, Study Location: US
NCT00957476, MetroHealth Medical, Center, Eunice Kennedy Shriver National Institute of Child, Health and Human Development	Omega‐3 Supplementation Decreases Inflammation and Fetal Obesity in Pregnancy	2009		Exclusion reason: Wrong patient population; Study location: US
NCT01038453, University of Aarhus	Effects of Vitamin D Supplement Before and During Pregnancy on Birth Weight	2009		Exclusion reason: Wrong patient population, Study location: Denmark
NCT01047098, University of California Davis	Effects of Taking Prenatal Vitamin‐mineral Supplements During Lactation on Iron Status and Markers of Oxidation	2010		Exclusion reason: Wrong patient population, Study location: US
NCT01060735, Rabin Medical Center	Vitamin D Supplementation During Pregnancy and Bone Status in Children at Birth and at One Year of Age	2010		Exclusion reason: Wrong patient population, Study location: Israel
NCT01073033, Nestlé, University of the Philippines	Oral Supplement for Pregnant and Lactating Mothers	2010		Exclusion reason: Wrong intervention, Milk supplement
NCT01127022, Cornell University, American Egg Board, National Cattlemen's Beef Association, USDA Beltsville Human Nutrition Research Center	Effect of Maternal Choline Intake on Choline Status and Health Biomarkers During Pregnancy and Lactation	2010		Exclusion reason: Wrong patient population, Study Location: US
NCT01158976, University of Chicago, Eunice Kennedy Shriver National Institute of Child, Health and Human Development	Effect of Omega‐3 Supplementation During Pregnancy on Regulation of Stress	2010		Exclusion reason: Wrong patient population
NCT01180933, Ludwig‐Maximilians ‐ University of Munich, European Union	Fish Oil and Folate Supplementation During Pregnancy	2010		Exclusion reason: Wrong patient population, Study Location: Germany, Hungary, Spain
NCT01221844, Clinica Fabia Mater	Bovine Lactoferrin to Prevent and Cure Iron Deficiency and Iron Deficiency Anemia in Complicated Pregnancies	2010		Exclusion reason: Wrong patient population
NCT01235767, Children's Hospital & Research Center, Oakland	Animal Source Food Supplement and Pregnancy in Vietnam	2010		Exclusion reason: Wrong patient population
NCT01264042, Swiss Federal Institute of Technology, University of Zurich	Iron Supplementation During Pregnancy and Non‐Transferrin‐Bound Iron (NTBI)	2010		Exclusion reason: Wrong patient population, Study Location: Switzerland
NCT01302756, Mike O'Callaghan Federal Hospital	The Effect of High Dose Folic Acid Versus Placebo on the Rate of Gestational Diabetes or Gestational Hypertension in Pregnant Women	2011		Exclusion reason: Wrong patient population, Study Location: US
NCT01353807, Centre for Fetal Programming Denmark, National Institutes of Health, The Danish Council for Strategic Research, Danish Council for Independent Research, Lundbech Foundation, EU Commision, D.G.	Impact of Fish Oil Supplementation in 3rd Trimester of Pregnancy on Maternal and Offspring Health	2011		Exclusion reason: Wrong patient population, Study Location: Denmark
NCT01417351, USDA Western Human Nutrition Research Center, University of California Davis	Effects of Vitamin D Supplementation During Pregnancy on Clinical Outcomes and Immune Function	2011		Exclusion reason: Wrong patient population, Study location: US
NCT01510665, University of California, Los Angeles	Magnesium Supplementation in the Second Trimester of Pregnancy to Overweight and Obese Individuals	2011		Exclusion reason: Wrong patient population
NCT01511835, AGUNCO Obstetrics and Gynecology Centre	Efficacy of Myo‐inositol in Preventing Gestational Diabetes in High‐risk Pregnant Women	2011		Exclusion reason: Wrong patient population, Study Location: Italy
NCT01542970, Ostergotland County Council, Sweden	Can Supplementation With Lactobacillus Reuteri and Omega‐3 Fatty Acids During Pregnancy and Lactation Reduce the Risk of Allergic Disease in Infancy?	2012		Exclusion reason: Wrong patient population, Study Location: Sweden
NCT01723696, Oregon Health & Science University, National Heart, Lung and Blood Institute, Office of Dietary Supplements	Vitamin C to Decrease Effects of Smoking in Pregnancy on Infant Lung Function	2012		Exclusion reason: Wrong intervention
NCT01732328, Universidade Federal do Rio de Janeiro, Rio de Janeiro State University	Effect of Calcium Plus Vitamin D Supplementation on Adolescent Mother and Infant Bone Health	2012		Exclusion reason: Wrong intervention
NCT01741077, The Hospital for Sick Children	The Effect of Folic Acid Supplementation and Pregnancy on the Folate Forms in Red Blood Cells	2012		Exclusion reason: Wrong patient population, Study Location: Canada
NCT01795131, International Centre for Diarrhoeal Disease Research, Bangladesh	Vitamin B12 Supplementation During Pregnancy	2013		Exclusion reason: Wrong patient population, Mild to moderate anaemia
NCT01818180, Hopital Foch	Urell and Pregnancy	2013		Exclusion reason: Wrong patient population
NCT01832688, University of California Davis, Helen Keller International Micronutrient Initiative	Assessment of the Nutritional Status of Pregnant Women and Optimization of Prenatal Care Services	2013		Exclusion reason: Wrong intervention
NCT01932788, Medical University of South Carolina, W. K. Kellogg Foundation	Preventing Health Disparities During Pregnancy Through Vitamin D Supplementation	2013		Exclusion reason: Wrong patient population, Study location: US
NCT02005588, Cairo, University	Treatment of Iron Deficiency Anemia With Pregnancy	2013		Exclusion reason: Wrong patient population
NCT02007837, U. M. C. Utrecht, University of Ghana Health Services	Prospects for the Prevention of Pregnancy‐induced Hypertension and Preeclampsia Trial	2013		Exclusion reason: Wrong patient population
NCT02086838, Ain Shams University	Iron Supplementation Using Total Dose Infusion and Oral Routes for Treatment of Iron Deficiency Anemia in Pregnancy	2014		Exclusion reason: Wrong patient population
NCT02124642, University of Georgia, University of Florida, Emory University, Cornell University	Folic Acid Supplementation in Pregnant Women: Dose Response	2014		Exclusion reason: Wrong patient population, Study location: US
NCT02137408, Cincinnati Children's Hospital Medical CenterNationwide Children's Hospital	Docosahexaenoic Acid Supplementation of Women With Hypertension in Pregnancy to Improve Endothelial Health	2014		Exclusion reason: Wrong patient population
NCT02219399, Colorado State University, USDA Beltsville Human Nutrition Research Center	DHA Supplementation and Pregnancy Outcome	2014		Exclusion reason: Wrong patient population, Study location: US
NCT02229526, Sjurdur Frodi Olsen, Rigshospitalet, Denmark	Fish Oil Trials in Pregnancy for the Prevention of Pregnancy Complications ('FOTIP')	2014		Exclusion reason: Wrong patient population, Study location: Denmark, Scotland, Sweden, England, Italy, The Netherlands, Norway, Belgium and Russia. Data from each country is not defined
NCT02238704, Cornell University, Kenya Ministry of Health, University of Nairobi, Micronutrient Initiative	Cornell University‐Micronutrient Initiative Calcium Supplementation Study	2014		Exclusion reason: Wrong patient population, Study location: US
NCT02371460, C.H.U. de Quebec‐Universite Laval, Canadian Institutes of Health Research, Laval University	Maternal Omega‐3 Supplementation to Reduce Bronchopulmonary Dysplasia	2015		Exclusion reason: Wrong patient population, Study Location: Canada
NCT02487719, University of Zurich	Different Iron Supplements for Prevention of Anemia in Pregnancy	2015		Exclusion reason: Wrong patient population, Study location: Switzerland
NCT02506439, Kiely, M., European Commission	Nutritional Requirements for Vitamin D in Pregnant Women	2015		Exclusion reason: Wrong patient population, Study location: Ireland
NCT02536352, Johns Hopkins University	Effect of Supplementation of Fluoride on Maternal Periodontal Health, Preterm Delivery, and Perinatal Well‐Being	2015		Exclusion reason: Wrong patient population, Study location: US
NCT02574767, University of Chile, Fondo Nacional de Desarrollo Científico y Tecnológico, Chile; DSM Nutritional Products, Inc; Corporacion Para Apoyo De La Investigacion Cientifica En Nutricion	Diet and Physical Activity Counseling and n3‐long Chain (PUFA) Supplementation in Obese Pregnant Women	2015		Exclusion reason: Wrong intervention
NCT02647723, University of Chicago, Eunice Kennedy Shriver National Institute of Child, Health and Human, Development, University of Pittsburgh	Improving Maternal and Child Health Through Prenatal Fatty Acid Supplementation	2016		Exclusion reason: Wrong patient population; Study location: US
NCT02705287, Cornell University, University of Rochester	Vitamin D Dynamics in Women	2016		Exclusion reason: Wrong patient population, Wrong setting
NCT02920593, Stony Brook University	A Randomized Control Trial of Vitamin D Prophylaxis in the Prevention of Hypertensive Disorders of Pregnancy	2016		Exclusion reason: Wrong patient population, Study Location: US
NCT02922803, Uppsala University	Vitamin D and Physical Performance Before and After Intervention and Birth Outcome in Pregnant Somali and Swedish Women	2016		Exclusion reason: Wrong patient population, Study Location: Sweden
NCT02925013, Sheba Medical Center	Maternal Dietary Lipids and Omega 3 Essential Fatty Acids Profile in Pregnant Women at Term and Their Fetuses	2016		Exclusion reason: Wrong patient population, Study Location: Israel
NCT02957643, Hippocration General Hospital	Substitution of the Normal Levels of Iron and Hemoglobin in Pregnant Women With Iron Supplement	2016		Exclusion reason: Wrong patient population, Anemic population: Less than 10.5 g/dl
NCT02959125,Semarang Health Polytechnic	NutFish and Nutrient Supplementation in Pregnancy Class to Improve Maternal and Birth Outcomes	2016		Exclusion reason: Wrong patient population, Anemic population
NCT03037593, University of Minnesota Clinical Translational Science Institute	High Dose Cholecalciferol to Reduce the Incidence of Gestational Diabetes in High Risk Pregnant Women	2017		Exclusion reason: Wrong patient population
NCT03101150, King Fahad Medical City	Effect of Vitamin D3 Supplementation in Pregnancy on Risk of Pre‐eclampsia	2017		Exclusion reason: Wrong patient population, Study Location: Saudi Arabia
NCT03117660, University of Washington, National Institute of General Medical Sciences	Effects of Retinoids on CYP2D6 Activity During Pregnancy	2017		Exclusion reason: Wrong outcomes
NCT03202615, Abdelwahed Mai Mahmoud Mohamed M. D.	Bovine Lactoferrin Versus Ferrous Sulphate In The Treatment Of Iron Deficiency Anemia During Pregnancy	2015		Exclusion reason: Wrong patient population, Anemic population
NCT03212781, Assiut University	Iron Dextran Versus Oral Iron for Treating Iron Deficiency Anemia in Pregnant Women	2017		Exclusion reason: Wrong patient population, Anemic population
NCT03215784, Universidade Federal do Rio de Janeiro, Maternidade Escola da UFRJ	Gestational Obesity and Interventions With Probiotics or Fish Oil Trial	2017		Exclusion reason: Wrong patient population
NCT03258385, International Centre for Diarrhoeal Disease Research, Bangladesh, University of California Davis	Vitamin B12 Supplementation to Improve B12 Status and Child Development	2017		Exclusion reason: Wrong intervention, Fortified milk supplementation, Fortified milk supplementation
NCT03265704, Romanian Society of Anesthesia and Intensive Care, University of Medicine, "Victor Babes" University of Medicine and Pharmacy Timisoara, Timişoara County Emergency Clinical Hospital	Maternal High Blood Pressure and Newborn's Blood Profile	2017		Exclusion reason: Wrong intervention
NCT03279536, Woman's Health University Hospital, Egypt	Lactoferrin Versus Total Dose Infusion (TDI) For Treating Iron Deficiency Anemia in Pregnancy: Role of Nursing	2017		Exclusion reason: Wrong patient population; anemic population;
NCT03310983, University of Kansas Medical, Center	Growth and Adiposity in Newborns: The Influence of Prenatal DHA Supplementation	2017		Exclusion reason: Wrong patient population, Study location: US
NCT03377218, Riga Stradins University, Latvian Biomedical, Research and Study Centre, Riga Maternity hospital, Latvian Institute of Organic Synthesis	Potential Preventive Effect of Selenium on Iodine‐induced Thyroid Autoimmunity During Pregnancy	2017		Exclusion reason: Wrong intervention
NCT03378791, Hatem, A. H.	Efficacy of Iron Bisglycinate in Treatment of Iron Deficiency Anemia in Pregnant Women	2017		Exclusion reason: Wrong patient population
NCT03438227, Washington University School of Medicine	Intravenous Iron for Iron‐deficiency Anemia in Pregnancy: a Randomized Controlled Trial	2018		Exclusion reason: Wrong patient population
NCT03562143, Icahn School of Medicine at Mount Sinai	Daily vs Alternate Day Iron Supplementation for Pregnant Women With Iron Deficiency Anemia	2018		Exclusion reason: Wrong outcomes
Neogi, S. B., Singh, S., Pallepogula, D. R., Pant, H., Kolli, S. R., Bharti, P., Datta, V., Gosla, S. R., Bonanthaya, K., Ness, A., Kinra, S., Doyle, P., Gudlavalleti, V. S. M.	Risk factors for orofacial clefts in India: A case‐control study	2017	Birth Defects Research	Exclusion reason: Wrong study design
Nestel, P. S., Jackson, A. A.	The impact of maternal micronutrient supplementation on early neonatal morbidity	2008	Archives of Disease in Childhood	Exclusion reason: Wrong study design
Nestel, P., Nalubola, R., Sivakaneshan, R., Wickramasinghe, A. R., Atukorala, S., Wickramanayake, T., Godowatta, R., Camilus, J., Dharmaselan, J., Ganapathy, Sunamaratne, N.	The Use of Iron‐fortified Wheat Flour to Reduce Anemia among the Estate Population in Sri Lanka	2004	International Journal for Vitamin and Nutrition Research	Exclusion reason: Wrong patient population
Neves, P. A. R., Saunders, C., de Barros, D. C., Ramalho, A.	Vitamin A supplementation in Brazilian pregnant and postpartum women: a systematic review	2015	Revista Brasileira de Epidemiologia	Exclusion reason: Wrong study design
Nguyen, P., Sanghvi, T., Mahmud, Z., Tran, L., Shabnam, S., Aktar, B., Afsana, K., Frongillo, E., Ruel, M., Menon, P.	Integrating nutrition‐focused behavior change communication and community mobilization into existing Maternal, Neonatal and child health platform improved consumption of diversified foods and micronutrients and exclusive breastfeeding practices in Bangladesh: Results of a cluster‐randomized program evaluation	2017	FASEB Journal. Conference: Experimental Biology	Exclusion reason: Wrong study design
Nidhi, J., Melse‐Boonstra, A., Sharma, S. K., Krishnamachari, S., Zimmermann, M. B.	The iodized salt programme in Bangalore, India provides adequate iodine intakes in pregnant women and more‐than‐adequate iodine intakes in their children	2015	Public Health Nutrition	Exclusion reason: Wrong intervention
Nikiema, L., Martin‐Prevel, Y., Moursi, M., Ouedraogo, G., Kossivavi‐Ayassou, I., Lanou, H., Allemand, P., Moura, F.	Prevalence of micronutrient deficiencies and anthropometric status of women and preschool children in rural Burkina Faso	2013	Annals of Nutrition and Metabolism	Exclusion reason: Wrong intervention
Nili, F., Jahangiri, M.	Risk factors for neural tube defects: a study at university‐affiliated hospitals in Tehran	2006	Archives of Iranian medicine	Exclusion reason: Wrong intervention
Nisar, Y. B., Dibley, M. J.	Antenatal iron‐folic acid supplementation reduces risk of low birthweight in Pakistan: secondary analysis of Demographic and Health Survey 2006‐2007	2016	Maternal and Child Nutrition	Exclusion reason: Wrong intervention
Nisar, Y. B., Dibley, M. J.	Iron/folic acid supplementation during pregnancy prevents neonatal and under‐five mortality in Pakistan: propensity score matched sample from two Pakistan Demographic and Health Surveys	2016	Global health action	Exclusion reason: Wrong intervention
Nisar, Y. B., Dibley, M. J.	Earlier initiation and use of a greater number of iron‐folic acid supplements during pregnancy prevents early neonatal deaths in Nepal and Pakistan	2014	PLoS ONE	Exclusion reason: Wrong intervention
Nisar, Y. B., Dibley, M. J., Aguayo, V. M.	Iron‐folic acid supplementation during pregnancy reduces the risk of stunting in children less than 2 years of age: A retrospective cohort study from Nepal	2016	Nutrients	Exclusion reason: Wrong intervention
Nisar, Y., Dibley, M. J.	Earlier initiation and use of a greater number of iron‐folic acid supplements during pregnancy prevents early neonatal deaths in Nepal and Pakistan	2014	PLoS ONE [Electronic Resource]	Exclusion reason: Wrong intervention
Nisar, Y., Dibley, M. J., Saba, M., Naveen, P., Madhu, D.	Antenatal iron‐folic acid supplementation reduces neonatal and under‐5 mortality in Nepal	2015	Journal of Nutrition	Exclusion reason: Wrong intervention
Nogueira, N. do N., Macedo, A. da S., Parente, J. V., Cozzolino, S. M. F.	Nutritional profile of newborns of adolescent mothers supplemented with iron, in different concentrations, zinc and pholic acid	2002	Revista de Nutricao	Exclusion reason: Wrong patient population
NTR4959, University Medical Center Groningen	ZOOG MUM	2014		Exclusion reason: Wrong patient population
Nuzhat, N., Samina, J., Sarwat, J., Sajjad, S., Riffat, S.,Rehana, H.	Use of folic acid among pregnant women with congenital anomalies in a hospital population	2011	HUGO Journal	Exclusion reason: Wrong intervention
Nwagha, U. I., Ejezie, F. E.	Serum ascorbic acid levels during pregnancy in Enugu, Nigeria	2005	Journal of College of Medicine	Exclusion reason: Wrong intervention
Nyarko, K. A., Wehby, G. L.	Implications of the folic acid fortification mandate on infant and child health	2014		Exclusion reason: Wrong intervention
Obeid, R., Murphy, M., Sole‐Navais, P., Yajnik, C.	Cobalamin Status from Pregnancy to Early Childhood: Lessons from Global Experience	2017	Advances in Nutrition	Exclusion reason: Wrong study design
Ogundipe, O., Hoyo, C., Ostbye, T., Oneko, O., Manongi, R., Lie, R. T., Daltveit, A. K.	Factors associated with prenatal folic acid and iron supplementation among 21,889 pregnant women in Northern Tanzania: a cross‐sectional hospital‐based study	2012	BMC Public Health	Exclusion reason: Wrong intervention
Okwara, J. E., Nnabuo, L. C., Nwosu, D. C., Ahaneku, J. E., Anolue, F., Okwara, N. A., Amah, U. K., Meludu, S. C., Dioka, C. E., Okwara, E. C., Egwurugwu, J. N., Ubajaka, C. F., Chukwulebe, A. E.	Iron status of some pregnant women in Orlu town‐‐eastern Nigeria	2013	Nigerian Journal of Medicine: Journal of the National Association of Resident Doctors of Nigeria	Exclusion reason: Wrong intervention
Oliveira, J. M., Rondo, P. H.	Evidence of the impact of vitamin A supplementation on maternal and child health [Article in Portuguese]	2007	Cadernos de Saude Publica	Exclusion reason: Wrong study design
Omotayo, M., Stoltzfus, R., Martin, S., Kung'u, J., Dickin, K.	WHO guidelines on calcium supplementation for prevention of preeclampsia: adoption, feasibility and acceptability in rural Kenya	2016	FASEB journal. Conference: experimental biology 2016, EB. San diego, CA united states. Conference start: 20160402. Conference end: 20160406. Conference publication: (var.pagings)	Exclusion reason: Wrong intervention
Ononge, S., Campbell, O., Mirembe, F.	Haemoglobin status and predictors of anaemia among pregnant women in Mpigi, Uganda	2014	BMC Research Notes	Exclusion reason: Wrong intervention
Oral, E., Kumbak, B., Senturk, L. M., Aksu, F.	Prophylactic oral iron supplementation during pregnancy: Is it necessary? [Article in Turkish]	2002	Jinekoloji ve Obstetrik Dergisi	Exclusion reason: Wrong intervention
Orjuela, M. A., Cabrera‐Munoz, L., Paul, L., Ramirez‐Ortiz, M. A., Liu, X., Chen, J., Mejia‐Rodriguez, F., Medina‐Sanson, A., Diaz‐Carreno, S., Suen, I. H., Selhub, J., Ponce‐Castaneda, M. V.	Risk of retinoblastoma is associated with a maternal polymorphism in dihydrofolatereductase (DHFR) and prenatal folic acid intake	2012	Cancer	Exclusion reason: Wrong intervention
Osei, A., Septiari, A., Suryantan, J., Hossain, M. M., Chiwile, F., Sari, M., Pinto, P., Soares, D., Faillace, S.	Using formative research to inform the design of a home fortification with micronutrient powders (MNP) Program in Aileu District, Timor‐Leste	2014	Food & Nutrition Bulletin	Exclusion reason: Wrong intervention
Osendarp, S. J. M., West, C. E., Black, R. E.	The need for maternal zinc supplementation in developing countries: an unresolved issue	2003	Journal of Nutrition	Exclusion reason: Wrong study design
Ostos, H., Astaiza, G., Garcia, F., Bautista, M., Rojas, F., Bermudez, A.	Low incidence of congenital neural tube defects at the Neiva University Hospital: possible effect of folic acid supplementation	2000	Biomedica	Exclusion reason: Wrong study design
Osungbade, K. O., Ige, O. K.	Public health perspectives of preeclampsia in developing countries: implication for health system strengthening	2011	Journal of Pregnancy	Exclusion reason: Wrong study design
Otoluwa, A., Salam, A., Syauki, Y., Hasan, N., Monoarfa, Y., Hadju, V.	The effect of moringa oleifera leaf extracts supplementation to the pregnant women in preventing maternal DNA damage	2013	Annals of Nutrition and Metabolism	Exclusion reason: Wrong intervention
PACTR201105000267371, Department of Obstetrics Gynaecology	WHO randomized trial of calcium supplementation before pregnancy to reduce recurrent pre‐eclampsia	2010		Exclusion reason: Wrong patient population
Paganini, D., Zimmermann, M. B.	The effects of iron fortification and supplementation on the gut microbiome and diarrhea in infants and children: a review. (Special Issue: Iron screening and supplementation in iron‐replete pregnant women and young children.)	2017	American Journal of Clinical Nutrition	Exclusion reason: Wrong patient population
Palmer, A. C., Schulze, K. J., West, K. P.	Preconceptional through post‐partum vitamin A (VA) supplementation increases natural antibody concentrations of offspring aged 9‐13 years in rural Nepal	2011	FASEB Journal. Conference: Experimental Biology	Exclusion reason: Wrong outcomes

**Table 12:** Characteristics table of excluded studies (Database Searches)

Authors	Title	Published Year	Journal	Exclusion Reason
Palmer, A. C., Stewart, C. P.	Micronutrients, health and development: evidence‐based programs ‐ The 2nd International Meeting of the Micronutrient Forum, 12‐15 May 2009, Beijing, China	2009	Sight and Life Magazine	Exclusion reason: Wrong intervention
Pandey, P., Bajpai, P., Jain, S., Sharma, A.	Maternal empowerment holds the key to reducing stunting during first 1000 days of life: Evidence from a case‐controlled study	2017	Annals of Tropical Medicine and Public Health	Exclusion reason: Wrong study design
Papathakis, P., Schaffner, A., Garcia, P., Eckert, R., Fry, J., Malek, S., Trehan, I., Thakwalakwa, C., Maleta, K., Manary, M.	Provision of supplementary food to pregnant malawian women with moderate acute malnutrition improves gestational weight gain and reduces low birth weight	2017	FASEB Journal. Conference: Experimental Biology	Exclusion reason: Wrong patient population
Pappagallo, S., Bull, D. L.	Operational problems of an iron supplementation programme for pregnant women: an assessment of UNRWA experience	1996		Exclusion reason: Wrong intervention
Pappagallo, S., Bull, D. L.	Operational problems of an iron supplementation programme for pregnant women: an assessment of UNRWA experience	1996	Bulletin of the World Health Organization	Exclusion reason: Wrong study design
Pasricha, S. R., Biggs, B. A., Prashanth, N. S., Sudarshan, H., Moodie, R., Black, J., Shet, A.	Factors influencing receipt of iron supplementation by young children and their mothers in rural India: local and national cross‐sectional studies	2011	BMC Public Health	Exclusion reason: Wrong patient population
Pathak, P., Kapil, U., Kapoor, S. K., Saxena, R., Kumar, A., Gupta, N., Dwivedi, S. N., Singh, R., Singh, P.	Prevalence of multiple micronutrient deficiencies amongst pregnant women in a rural area of Haryana	2004	Indian Journal of Pediatrics	Exclusion reason: Wrong intervention
Patil, S. B., Kodliwadmath, M. V., Kodliwadmath, S. M., Patil, M. B.	Significance of supplementation of antioxidants vitamin‐E and vitamin‐C therapy in third trimester pre‐eclampsia	2016	Indian Journal of Clinical Biochemistry	Exclusion reason: Wrong intervention
Paul, K. H., Muti, M., Chasekwa, B., Mbuya, M. N. N., Madzima, R. C., Humphrey, J. H., Stoltzfus, R. J.	Complementary feeding messages that target cultural barriers enhance both the use of lipid‐based nutrient supplements and underlying feeding practices to improve infant diets in rural Zimbabwe	2012	Maternal and Child Nutrition	Exclusion reason: Wrong patient population
Pehlivan, I., Hatun, S., Aydogan, M., Babaoglu, K., Gokalp, A. S.	Maternal vitamin D deficiency and vitamin D supplementation in healthy infants	2003	Turkish Journal of Pediatrics	Exclusion reason: Wrong patient population
Pei, L. J., Li, Z. W., Zhang, W., Ren, A. G., Zhu, H. P., Hao, L., Zhu, J. H., Li, Z.	Epidemiological study on reduced folate carrier gene(RFC1 A80G) polymorphism and other risk factors of neural tube defects. [Article in Chinese]	2005	Beijing da xue xue bao	Exclusion reason: Wrong intervention
Pei, L., Zhu, H. P., Zhu, J., Ren, A., Finnell, R. H., Li, Z.	Genetic variation of infant reduced folate carrier (A80G) and risk of orofacial defects and congenital heart defects in China	2006	Annals of Epidemiology	Exclusion reason: Wrong intervention
Pena‐Rosas, J. P., De‐Regil, L. M., Dowswell, T., Viteri, F. E.	Intermittent oral iron supplementation during pregnancy	2012	Cochrane Database of Systematic Reviews	Exclusion reason: Wrong study design
Peng, T., Li, X., Wang, L.	Effects of periconceptional folate intake and methylenetetrahydrofolate reductase gene C667T polymorphism of pregnant women on congenital heart diseases in offspring	2009	Journal of Environment and Health	Exclusion reason: Wrong intervention
Perez Guillen, A., Bernal Rivas, J.	Prediction of the nutritional status by anthropometrical variables and food safety at homes of pregnant women from Caracas, Venezuela [Article in Spanish]	2006	Nutricion Hospitalaria	Exclusion reason: Wrong study design
Perez‐Escamilla, R.	Periconceptional folic acid and neural tube defects: public health issues	1995	Bulletin of the Pan American Health Organization	Exclusion reason: Wrong study design
Perry, H., Muita, J. W. G., Omwega, A. M.	Dietary habits, pregnancy weight gain and birthweights in a highland population of Kenya	1996	East African Medical Journal	Exclusion reason: Wrong intervention
Persson, L. A., Arifeen, S., Ekstrom, E. C.	Prenatal micronutrient and early pregnancy food supplementation in Bangladesh ‐ Reply	2012	JAMA ‐ Journal of the American Medical Association	Exclusion reason: Wrong study design
Peyrin‐Biroulet, L., Williet, N., Cacoub, P.	Guidelines on the diagnosis and treatment of iron deficiency across indications: A systematic review	2015	American Journal of Clinical Nutrition	Exclusion reason: Wrong study design
Pezzack, B., Zlotkin, S.,; Abrams, S., Hawthorne, K., Al, M. A., Baxter, J,‐A., Phillips, A. A., Islam, M., Dimitris, M., Ahmed, T., Roth, D.	Bioavailability of enteric‐coated microencapsulated calcium during pregnancy: a randomized crossover trial in Bangladesh	2014	FASEB Journal	Exclusion reason: Wrong study design
Pinheiro, R., Melendez‐Araujo, M., Bastos, A., Arruda, S.	Nutritional implications in pregnancy after gastric bypass surgery: Maternal and fetal aspects	2015	Obesity Surgery	Exclusion reason: Wrong intervention
Poulton, M.	Iodine deficiency in the Northern Punjab of Pakistan	1997		Exclusion reason: Wrong intervention
Prasad, A. S.	Zinc in human health: An update	1998	Journal of Trace Elements in Experimental Medicine	Exclusion reason: Wrong study design
Priyanka, K., Usha, S.	Nutritional composition and acceptability testing of ladoo developed for pregnant women	2005	Journal of Dairying, Foods and Home Sciences	Exclusion reason: Wrong intervention
Qamar, H., Perumal, N., Papp, E., Gernand, A. D., Mahmud, A. A., Roth, D.	Parathyroid hormone and fetal length in a pregnancy cohort in Dhaka, Bangladesh	2017	Annals of Nutrition and Metabolism	Exclusion reason: Wrong intervention
Rabiu, T. B., Tiamiyu, L. O., Awoyinka, B. S.	Awareness of spina bifida and periconceptional use of folic acid among pregnant women in a developing economy	2012	Childs Nervous System	Exclusion reason: Wrong intervention
Rah, J. H., Depee, S., Kraemer, K., Steiger, G., Bloem, M. W., Spiegel, P., Wilkinson, C., Bilukha, O.	Program experience with micronutrient powders and current evidence	2012	Journal of Nutrition	Exclusion reason: Wrong study design
Rah, J. H., de Pee, S., Halati, S., Parveen, M., Mehjabeen, S. S., Steiger, G., Bloem, M. W., Kraemer, K.	Provision of micronutrient powder in response to the Cyclone Sidr emergency in Bangladesh: cross‐sectional assessment at the end of the intervention	2011	Food and nutrition bulletin	Exclusion reason: Wrong intervention
Raheem, N. A. A., Farhood, H. F.	Study the component of nutritional assessment of teenage pregnancy attending primary health care center in Babylon Province 2016	2016	Research Journal of Medical Sciences	Exclusion reason: Wrong intervention
Rahmanian, M., Aghayan, S., Ghorbani, R.	Effect of combined vitamin C and E supplementation for preventing preeclampsia and some outcomes of pregnancy in nulliparous women	2011	Iranian Journal of Obstetrics, Gynecology and Infertility	Exclusion reason: Wrong intervention
Raina, S. K., Vijay, M., Gurdeep, S.	Differentials in iron folic acid supplementation among pregnant women in a rural area of North‐West, India	2013	International Journal of Health and Allied Sciences	Exclusion reason: Wrong intervention
Ramakrishnan, U.	Fatty acid status and maternal mental health	2011	Maternal & Child Nutrition	Exclusion reason: Wrong study design
Ramakrishnan, U.	Fatty acid status and maternal mental health	2011	Maternal and Child Nutrition	Exclusion reason: Wrong study design
Ramakrishnan, U.	Nutrition and low birth weight: from research to practice	2004	American Journal of Clinical Nutrition	Exclusion reason: Wrong study design
Ramakrishnan, U., Darnton‐Hill, I.	Assessment and control of vitamin A deficiency disorders	2002	Journal of Nutrition	Exclusion reason: Wrong study design
Ramakrishnan, U., Imhoff‐Kunsch, B., Digirolamo, A. M.	Role of docosahexaenoic acid in maternal and child mental health	2009	American Journal of Clinical Nutrition	Exclusion reason: Wrong intervention
Ramakrishnan, U., Imhoff‐Kunsch, B., Lammi‐Keefe, C. J., Couch, S. C., Philipson, E. H.	Anemia and iron deficiency in developing countries. (Nutrition and Health)	2008		Exclusion reason: Wrong study design
Ramakrishnan, U., Manjrekar, R., Rivera, J., Gonzales‐Cossio, T., Martorell, R.	Micronutrients and pregnancy outcome: A review of the literature	1999	Nutrition Research	Exclusion reason: Wrong study design
Ramirez, J. O., Cabrera, S. A. S., Hidalgo, H., Cabrera, S. G., Linnebank, M., Bassetti, C., Kallweit, U.	Restless legs syndrome and pregnancy: Frequency and characteristics in a Peruvian population	2012	Journal of Neurology	Exclusion reason: Wrong intervention
Ramirez‐Velez, R., Romero, M., Echeverri, I., Ortega, J. G., Mosquera, M., Salazar, B., Giron, S. L., Saldarriaga, W., Aguilar de Plata, A. C., Mateus, J. C.	A factorial randomized controlled trial to evaluate the effect of micronutrients supplementation and regular aerobic exercise on maternal endothelium‐dependent vasodilatation and oxidative stress of the newborn	2011	Trials	Exclusion reason: Wrong intervention
Rashid, B., Richard, M. M., Gul e, R.	Low serum calcium levels in pre‐eclampsia	2015	Journal of SAFOG	Exclusion reason: Wrong intervention
Rawal, A., Girisha, K. M.	Use of periconceptional folic acid for prevention of neural tube defects‐where are we	2015	Journal of Obstetrics and Gynaecology Research	Exclusion reason: Wrong study design
Razavi, M., Jamilian, M., Kashan, Z. F., Heidar, Z., Mohseni, M., Ghandi, Y., Bagherian, T., Asemi, Z.	Selenium supplementation and the effects on reproductive outcomes, biomarkers of inflammation, and oxidative stress in women with polycystic ovary syndrome	2016	Hormone and Metabolic Research	Exclusion reason: Wrong patient population
RBR‐524z9n, Universidade Federal do Rio de Janeiro ‐ UFRJ ‐ Rio de Janeiro, R. J. Brazil, Conselho Nacional de Desenvolvimento Científico e Tecnológico ‐ Brasilia, D. F. Brazil	Contributions to Prenatal Care	2016		Exclusion reason: Wrong patient population
RBR‐6ys9yp, Universidade Federal do Rio Grande do Norte ‐ UFRN ‐ Natal, R. N. Brazil, Universidade Federal do Rio Grande do Norte ‐ UFRN ‐ Natal, R. N. Brazil	Evaluation of the effect of vitamin E supplementation on vitamin A values of mother and child	2017		Exclusion reason: Wrong intervention
Refsum, H.	Folate, vitamin B12 and homocysteine in relation to birth defects and pregnancy outcome	2001	British Journal of Nutrition	Exclusion reason: Wrong study design
Rekha, A., Kalaiselvi, S., Vinod, A., Singh, D., Bondu, J. D., Chapla, A., George, B., Alok, S., Edison, E. S.	Association of genetic variants with response to iron supplements in pregnancy	2015	Genes & Nutrition	Exclusion reason: Wrong patient population
Ren, A. G., Zhang, L., Li, Z. W., HaoL., Tian, Y. H., Li, Z.	Awareness and use of folic acid, and blood folate concentrations among pregnant women in northern China ‐ an area with a high prevalence of neural tube defects	2006	Reproductive Toxicology	Exclusion reason: Wrong intervention
Rendell, S., Korgo, P.	A fourth delay: Malaria prevention and nutritional supplementation in the first trimester for pregnant women in Burkina Faso	2013	American Journal of Tropical Medicine and Hygiene	Exclusion reason: Wrong intervention
Reveiz, L., Gyte, G. M., Cuervo, L. G., Casasbuenas, A.	Treatments for iron‐deficiency anaemia in pregnancy	2011	Cochrane Database of Systematic Reviews	Exclusion reason: Wrong study design
Rinku, S.	Birth defects in India: hidden truth, need for urgent attention	2013	Indian Journal of Human Genetics	Exclusion reason: Wrong study design
Ritu, P., Boora, P., Vandana	Nutritional evaluation of iron rich matar prepared from using locally available foodstuffs for pregnant women	2009	Annals of Agri Bio Research	Exclusion reason: Wrong intervention
Rodrigues, H. G., Gubert, M. B., Santos, L. M. P.	Folic acid intake by pregnant women from Vale do Jequitinhonha, Brazil, and the contribution of fortified foods	2015	Archivos Latinoamericanos de Nutricion	Exclusion reason: Wrong intervention
Rogers, M. S., Hung, C., Arumanayagam, M.	Platelet angiotensin II receptor status during pregnancy in Chinese women at high‐risk of developing pregnancy‐induced hypertension	1996	Gynecol Obstet Invest	Exclusion reason: Wrong patient population
Rojas‐Higuera, R., Londono‐Cardona, J. G., Arango‐Gomez, F.	Clinical practice in looking after breastfeeding women and the new‐born in some hospitals in Bogota, Colombia. [Article in Spanish]	2006	Revista de Salud Publica	Exclusion reason: Wrong study design
Rojas‐Higuera, R., Londono‐Cardona, J. G., Arango‐Gomez, F.	Clinical practice in looking after breastfeeding women and the new‐born in some hospitals in Bogota, Colombia	2006	Revista de Salud Publica	Exclusion reason: Wrong intervention
Rondo, P. H. C., Fukushima, C. M., Moraes, F.	Vitamin‐mineral supplement use by low‐income Brazilian pregnant adolescents and non‐adolescents and the predictors for non‐use	2006	European Journal of Clinical Nutrition	Exclusion reason: Wrong intervention
Ronsmans, C., Campbell, O.	Quantifying the fall in mortality associated with interventions related to hypertensive diseases of pregnancy	2011	BMC Public Health	Exclusion reason: Wrong outcomes
Ronsmans, C., Fisher, D. J., Osmond, C., Margetts, B. M., Fall, C. H., Maternal Micronutrient Supplementation Study Group	Multiple micronutrient supplementation during pregnancy in low‐income countries: a meta‐analysis of effects on stillbirths and on early and late neonatal mortality	2009	Food & Nutrition Bulletin	Exclusion reason: Wrong intervention
Rosano, A., Smithells, D., Cacciani, L., Botting, B., Castilla, E., Cornel, M., Erickson, D., Goujard, J., Irgens, L., Merlob, P., Robert, E., Siffel, C., Stoll, C., Sumiyoshi, Y.	Time trends in neural tube defects prevalence in relation to preventive strategies: an international study	1999	Journal of Epidemiology and Community Health	Exclusion reason: Wrong intervention
Rostami, M., Ramezani Tehrani, F., Simbar, M., Hosseinpanah, F., Alavi Majd, H.	Rationale and Design of Khuzestan Vitamin D Deficiency Screening Program in Pregnancy: A Stratified Randomized Vitamin D Supplementation Controlled Trial	2017	JMIR Research Protocols	Exclusion reason: Wrong study design
Rotar, I., Breban, A., Stamatian, F., Muresan, D.	Management and outcomes in women with hereditary thrombophilia during pregnancy	2015	European Journal of Clinical Investigation	Exclusion reason: Wrong patient population
Roth, D. E.	Vitamin D replacement in pregnant women in rural north India: a pilot study	2010	European Journal of Clinical Nutrition	Exclusion reason: Wrong study design
Roth, D. E.	Vitamin D replacement in pregnant women in rural north India: a pilot study	2010	European Journal of Clinical Nutrition	Exclusion reason: Wrong study design
Ruel‐Bergeron, J. C., Oemcke, R., Kapadia‐Kundu, N., Hurley, K., Christian, P.	Facilitators and barriers to access and utilization of a comprehensive nutrition program in rural Malawi ‐ A qualitative study	2017	FASEB Journal. Conference: Experimental Biology	Exclusion reason: Wrong study design
Ruel‐Bergeron, J., Hurley, K., Oemcke, R., Buckland, A., Kapadia‐Kundu, N., Kang, Y., Shu Fune Wu, L., Mitra, M., Phuka, J., Klemm, R., West, K., Christian, P.	Growing the evidence for nutrition programming: Perceptions and implementation of a stunting prevention program rural in Malawi	2017	Annals of Nutrition and Metabolism	Exclusion reason: Wrong study design
Rumbold, Alice, Ota, Erika, Hori, Hiroyuki, Miyazaki, Celine, Crowther, C. A.	Vitamin E supplementation in pregnancy	2015	Cochrane Database of Systematic Reviews	Exclusion reason: Wrong intervention
Rusdi, Soeradi, O., Subakir, S. B., Suyatna, F. D.	F2 alpha ‐isoprostane, Na+‐K+ ATPase and membrane fluidity of placental syncytiotrophoblast cell in preeclamptic women with vitamin E supplementation	2012	Medical journal of indonesia	Exclusion reason: Wrong intervention
Sahbaz, Z. A., Yuksel, K. B.	High frequency of vitamin B12 defficiency in pregnant women in Kutahya province of Turkey	2013	Journal of Perinatal Medicine. Conference: 11th World Congress of Perinatal Medicine	Exclusion reason: Wrong intervention
Salam, R. A., Das, J. K., Bhutta, Z. A.	Multiple micronutrient supplementation during pregnancy and lactation in low‐to‐middle‐income developing country settings: impact on pregnancy outcomes	2014	Annals of Nutrition and Metabolism	Exclusion reason: Wrong study design
Salam, R. A., Zuberi, N. F., Bhutta, Z. A.	Pyridoxine (vitamin B6) supplementation during pregnancy or labour for maternal and neonatal outcomes	2015	Cochrane Database of Systematic Reviews	Exclusion reason: Wrong intervention
Samimi, M, Kashi, M, Foroozanfard, F, Karamali, M, Bahmani, F, Asemi, Z, Hamidian, Y, Talari, Hr, Esmaillzadeh, A.	The effects of vitamin D plus calcium supplementation on metabolic profiles, biomarkers of inflammation, oxidative stress and pregnancy outcomes in pregnant women at risk for pre‐eclampsia	2016	Journal of human nutrition and dietetics	Exclusion reason: Wrong patient population
Sanchez, P. A., Idrisa, A., Bobzom, D. N., Airede, A., Hollis, B. W., Liston, D. E., Jones, D. D., Dasgupta, A., Glew, R. H.	Calcium and vitamin D status of pregnant teenagers in Maiduguri, Nigeria	1997	Journal of the National Medical Association	Exclusion reason: Wrong intervention
Sandhya, M., Pratibha, G., Pankaj, B., Beena, S., Srivastav, J. P., Mishra, A. N.	Effect of antenatal services during pregnancy on prevalence of anaemia amongst pregnant women in Lucknow	2016	Indian Journal of Medical Sciences	Exclusion reason: Wrong patient population
Sanghvi, T. G., Harvey, P. W., Wainwright, E.	Maternal iron‐folic acid supplementation programs: evidence of impact and implementation	2010	Food & Nutrition Bulletin	Exclusion reason: Wrong intervention
Sanoussi, S., Gamatie, Y., Kelani, A., Sbai, C., Abarchi, H., Bazira, L.	Neural tube malformations in Niger: a consideration of 387 cases in 10 years. The case for preventive treatment through the use of folic acid supplements at periconception	2001	Medecine d'Afrique Noire	Exclusion reason: Wrong study design
dos Santos, C. S., Kruze, I., Fernandes, T., Andreto, L. M., Figueiroa, J. N., Diniz, A. da S.	The effect of a maternal double megadose of vitamin A supplement on serum levels of retinol in children aged under six months	2013	Journal of Nutrition and Metabolism	Exclusion reason: Wrong patient population
Saravanan, P., Yajnik, C. S.	Role of maternal vitamin B12 on the metabolic health of the offspring: A contributor to the diabetes epidemic?	2010	British Journal of Diabetes and Vascular Disease	Exclusion reason: Wrong intervention
Sarika, C., Bharat, R.	Are we really making motherhood safe? A study of provision of iron supplements and emergency obstetric care in rural Maharashtra	2007	National Medical Journal of India	Exclusion reason: Wrong intervention
Sato, A. P. S., Fujimori, E., Szarfarc, S. C.	Hemoglobin level in pregnant women assisted by public prenatal services in the five regions of Brazil	2012	European Journal of Epidemiology	Exclusion reason: Wrong intervention
Sato, A. P. S., Fujimori, E., Szarfarc, S. C., Borges, A. L. V., Tsunechiro, M. A.	Food consumption and iron intake of pregnant and reproductive aged women	2010	Revista Latino Americana de Enfermagem	Exclusion reason: Wrong intervention
Sazawal, S., Dhingra, P., Vir, S. C.	Zinc ‐ an essential micronutrient for health and development. (Woodhead Publishing India in Food Science, Technology and Nutrition)	2011		Exclusion reason: Wrong study design
Schlossman, N., Brown, C., Batra, P., de Sa, A. B., Balan, I., Balan, A., Gamache, M. G., Wood, L., Pruzensky, W., Saltzman, E., Roberts, S. B., Bale, C.	A randomized controlled trial of two ready‐to‐use supplementary foods demonstrates benefit of the higher dairy supplement for reduced wasting in mothers, and differential impact in infants and children associated with maternal supplement response	2017	Food and nutrition bulletin	Exclusion reason: Wrong patient population
Schlossman, N, Batra, P, Balan, E, Pruzensky, W, Saltzman, E, Roberts, S	Effects of two ready to use supplementary foods (RUSF) containing different levels of dairy protein on mother's nutritional status in rural Guinea‐Bissau	2015	FASEB Journal	Exclusion reason: Wrong intervention
Schmidt, M.	Susceptibility for infections: Therapeutic option with zinc histidine. [Article in German]	2000	Biologische Medizin	Exclusion reason: Wrong intervention
Scholl, T. O.	Iron status during pregnancy: setting the stage for mother and infant	2005	American Journal of Clinical Nutrition	Exclusion reason: Wrong study design
Scott, J.	Folic acid consumption throughout pregnancy ‐ Differentiation between trimesters	2011	Annals of Nutrition and Metabolism	Exclusion reason: Wrong intervention
Seema, S., Alka, S.	Improvement in nutritional properties of product through fortification with aloevera, mushroom and pearl millets	2015	Plant Archives	Exclusion reason: Wrong intervention
Shah, D., Sachdev, H. P.	Maternal micronutrients and fetal outcome	2004	Indian Journal of Pediatrics	Exclusion reason: Wrong study design
Shahraki, Z, Bonjar, Zsh, Forghani, F, Nakhai, R	Comparing neonatal outcome following the use of ondansetron versus vitamin B6 in pregnant females with morning sickness: a randomized clinical trial	2016	Journal of comprehensive pediatrics	Exclusion reason: Wrong intervention
Shapira, N.	Prenatal nutrition: a critical window of opportunity for mother and child	2008	Women's health	Exclusion reason: Wrong study design
Sharma, J. B., Shankar, M.	Anemia in pregnancy	2010	Journal International Medical Sciences Academy	Exclusion reason: Wrong study design
Shatrugna, V., Raman, L., Kailash, U., Balakrishna, N., Rao, K. V.	Effect of dose and formulation on iron tolerance in pregnancy	1999	National Medical Journal of India	Exclusion reason: Wrong outcomes
Shcheplyagina, L. A.	Problems of iodine deficiency prevention under present‐day conditions	2000	Gigiena i Sanitariya	Exclusion reason: Wrong study design
Sheikh, S.	Evidence based interventions to prevent intergenerational stunting	2016	European Journal of Medical Research. Conference: 1st Liaquat University of Medical and Health Sciences, LUMHS PAK International Medical Research Conference. Pakistan.	Exclusion reason: Wrong study design
Shen, X., Yang, Z J., Zhang, L., Wang, L., Hua, X. L.	Analysis of correlation of vitamin D deficiency with the risk of gestational diabetes mellitus in pregnant women	2015	Progress in Modern Biomedicine	Exclusion reason: Wrong intervention
Shi, R., Moser, C.	Education and Nutrition Problems in Rural China	2017		Exclusion reason: Wrong patient population
Shrimpton, R., Schultink, W.	Can supplements help meet the micronutrient needs of the developing world?	2002	Proceedings of the Nutrition Society	Exclusion reason: Wrong study design
Siddiq, S., Apriatni, M., Prado, E., Santyowibowo, S., Lestari, Y., Sitorus, R., Shankar, A., Muadz, H.	The long term impact of maternal multiple micronutrient supplementation, infant nutrition and health on visual acuity of 9‐12 year old children in Indonesia	2015	FASEB Journal. Conference: Experimental Biology	Exclusion reason: Abstract
Siddiqua, T., Islam, S., Ahsan, K., Rashid, M., Hampel, D., Shahab‐Ferdows, S., Allen, L. H., Raqib, R.	Vitamin B12 supplementation of bangladeshi women in pregnancy and lactation: Effects on maternal and infant status, breast milk and immunity	2013	FASEB Journal. Conference: Experimental Biology	Exclusion reason: Wrong intervention

**Table 13:** Characteristics table of excluded studies (Database Searches)

Authors	Title	Published Year	Journal	Exclusion Reason
Sikder, S. S., Labrique, A. B., Shamim, A. A., Ali, H., Mehra, S., Wu, L., Shaikh, S., West, K. P., Christian, P.	Risk factors for reported obstetric complications and near misses in rural northwest Bangladesh: Analysis from a prospective cohort study	2014	BMC Pregnancy and Childbirth	Exclusion reason: Wrong outcomes
Da Silva, C. A. P., da Silva, C. A. P., Atallah, A. N., Sass, N., Mendes, E. T. R., Peixoto, S.	Evaluation of calcium and folic acid supplementation in prenatal care in Sao Paulo	2010	Sao Paulo Medical Journal	Exclusion reason: Wrong patient population
Silva, K. D. R. R., Munasinghe, D. L. L.	Urinary iodine concentration of pregnant women and female adolescents as an indicator of excessive iodine intake in Sri Lanka	2006	Food and nutrition bulletin	Exclusion reason: Wrong intervention
Simpson, J. L., Bailey, L. B., Pietrzik, K., Shane, B., Holzgreve, W.	Micronutrients and women of reproductive potential: Required dietary intake and consequences of dietary deficienty or excess. Part II ‐ vitamin D, vitamin A, iron, zinc, iodine, essential fatty acids	2011	Journal of Maternal‐Fetal and Neonatal Medicine	Exclusion reason: Wrong study design
Singh, P., Singh, R. K., Singh, R. B., Saboo, B., Elkilany, G., Hristova, K., Wilson, D. W., De Meester, F.	Effect of maternal dietary supplementation on complications of pregnancy and infancy and metabolic syndrome in later adult life	2015	World Heart Journal	Exclusion reason: Wrong study design
Smith, E. R., Shankar, A. H., Wu, L. S., Aboud, S., Adu‐Afarwuah, S., Ali, H., Agustina, R., Arifeen, S., Ashorn, P., Bhutta, Z. A., Christian, P., Devakumar, D., Dewey, K. G., Friis, H., Gomo, E., Gupta, P., Kaestel, P., Kolsteren, P., Lanou, H., Maleta, K., Mamadoultaibou, A., Msamanga, G.; Osrin, D., Persson, L. A., Ramakrishnan, U., Rivera, J. A., Rizvi, A., Sachdev, H. P. S., Urassa, W., West, K. P., Jr., Zagre, N., Zeng, L., Zhu, Z., Fawzi, W. W., Sudfeld, C. R.	Modifiers of the effect of maternal multiple micronutrient supplementation on stillbirth, birth outcomes, and infant mortality: a meta‐analysis of individual patient data from 17 randomised trials in low‐income and middle‐income countries	2017	The Lancet Global Health	Exclusion reason: Wrong study design
Soares, N.,; Mattar, R., Camano, L., Torloni, M.	Iron deficiency anaemia and iron deficiency in adult and adolescent women in pregnancy and after delivery	2009	International Journal of Gynecology and Obstetrics	Exclusion reason: Wrong intervention
Sohrabvand, F., Shariat, M., Haghollahi, F., Khezerdoust, S., Rahimi, F. A., Nazemi, L., Chammari, M.	Prevalence of leg cramps during pregnancy and effects of supplemental therapy	2006	Medical Journal of Reproduction & Infertility	Exclusion reason: Wrong outcomes
Soltanmora, S., Moghaddam‐Banaem, L., Amini, M.	Nutritional intakes of zinc, iron, and vitamins relations with serum levels of zinc and iron in early pregnancy	2015	Urmia Medical Journal	Exclusion reason: Wrong intervention
Sorri, G., Mesfin, E.	Patterns of Neural Tube Defects at Two Teaching Hospitals in Addis Ababa, Ethiopia a Three Years Retrospective Study	2015	Ethiopian Medical Journal	Exclusion reason: Wrong intervention
Souza, E. V., Torloni, M. R., Atallah, A. N., Santos, G. M. S. dos, Kulay, L., Jr., Sass, N.	Aspirin plus calcium supplementation to prevent superimposed preeclampsia: a randomized trial	2014	Brazilian Journal of Medical and Biological Research	Exclusion reason: Wrong patient population
Spíndola Garcêz, L., de Sousa Paz Lima, G., de Azevedo Paiva, A., Maria Rebêlo Sampaio da Paz, S., Lázaro Gomes, Erica I., Nunes, V. S., Cotta de Faria, E. de Barros‐Mazon, S.	Serum Retinol Levels in Pregnant Adolescents and Their Relationship with Habitual Food Intake, Infection and Obstetric, Nutritional and Socioeconomic Variables	2016		Exclusion reason: Wrong intervention
Srihareni, D., Lakshmi, U. K.	Effect of supplementation of Cynodon dactylon (Arugampul) juice/powder on anaemic pregnant women (20‐30 years) ‐ Part II	2001	Indian Journal of Nutrition and Dietetics	Exclusion reason: Wrong patient population
Sserunjogi, L., Scheutz, F., Whyte, S. R.	Postnatal anaemia: neglected problems and missed opportunities in Uganda	2003	Health Policy and Planning	Exclusion reason: Wrong intervention
Stamm, R. A., March, K. M., Karakochuk, C. D., Gray, A. R., Brown, R. C., Green, T. J., Houghton, L. A.	Lactating Canadian Women Consuming 1000 micro g Folic Acid Daily Have High Circulating Serum Folic Acid Above a Threshold Concentration of Serum Total Folate	2018	Journal of nutrition	Exclusion reason: Wrong patient population
Stevens, B., Buettner, P., Watt, K., Clough, A., Brimblecombe, J., Judd, J.	The effect of balanced protein energy supplementation in undernourished pregnant women and child physical growth in low‐ and middle‐income countries: a systematic review and meta‐analysis	2015	Maternal and Child Nutrition	Exclusion reason: Wrong study design
Stuetz, W., Carrara, V. I., McGready, R., Lee, S. J., Sriprawat, K., Po, B., Hanboonkunupakarn, B., Grune, T., Biesalski, H. K., Nosten, F. H.	Impact of food rations and supplements on micronutrient status by trimester of pregnancy: Cross‐sectional studies in the maela refugee camp in Thailand	2016	Nutrients	Exclusion reason: Wrong intervention
Stuetz, W., Carrara, V. I., McGready, R., Lee, S., Biesalski, H. K., Nosten, F. H.	Thiamine diphosphate in whole blood, thiamine and thiamine monophosphate in breast‐milk in a refugee population	2012	PLoS ONE [Electronic Resource]	Exclusion reason: Wrong patient population
Su, L.	Observation on effect of Jiangzu Decoction with calcium agent on the change of hemodynamics of hypertensive disorder during pregnancy	2010	Modern Preventive Medicine	Exclusion reason: Wrong patient population
Suarez, L., Hendricks, K., Felkner, M., Gunter, E.	Maternal serum B12 levels and risk for neural tube defects in a Texas‐Mexico border population	2003	Annals of Epidemiology	Exclusion reason: Wrong patient population
Suarez, L., Hendricks, K., Felkner, M., Gunter, E.	Maternal serum B12 levels and risk for neural tube defects in a Texas‐Mexico border population	2003	Annals of Epidemiology	Exclusion reason: Wrong patient population
Subadra, S.	Prevalence of micronutrient deficiency particularly of iron, zinc and folic acid in pregnant women in South East Asia. (Micronutrients, maternal and child health)	2001	British Journal of Nutrition	Exclusion reason: Wrong outcomes
Subba, N. R., Gurung, G.	A study of public health indicators of morang Nepal by lot quality assurance sampling method	2007	Nepal Medical College Journal: NMCJ	Exclusion reason: Wrong intervention
Suchdev, P. S., De‐Regil, L. M., Walleser, S., Vist, G. E., Peña‐Rosas, P.	Multiple micronutrient powders for home (point of use) fortification of foods in pregnant women: a systematic review	2011		Exclusion reason: Wrong study design
Suchdev, P. S, Peña‐Rosas, J. P., De‐Regil, L. M.	Multiple micronutrient powders for home (point‐of‐use) fortification of foods in pregnant women	2015	Cochrane Database of Systematic Reviews	Exclusion reason: Wrong study design
Sudfeld, C. R., McCoy, D. C., Fink, G., Muhihi, A., Bellinger, D. C., Masanja, H., Smith, E. R., Danaei, G., Ezzati, M., Fawzi, W. W.	Malnutrition and its determinants are associated with suboptimal cognitive, communication, and motor development in Tanzanian children	2015	Journal of Nutrition	Exclusion reason: Wrong patient population
Suesirisawad, C., Panamonta, O., Kiatchoosakun, P., Jirapradittha, J., Buppasiri, P., Thinkhamrop, J.	Correlation between maternal and neonatal urine iodine with thyroid‐stimulating hormone (TSH) levels in Srinagarind Hospital, Khon Kaen, Thailand	2013	Asian Biomedicine	Exclusion reason: Wrong intervention
Sukrat, B., Sirichotiyakul, S.	The prevalence and causes of anaemia during pregnancy in Maharaj Nakorn Chiang Mai Hospital	2006	Journal of the Medical Association of Thailand (Chotmaihet thangphaet)	Exclusion reason: Wrong intervention
Sumathi, S., Thomas, T., Kurpad, A. V.	B‐vitamin interventions for women and children in low‐income populations	2015	Current Opinion in Clinical Nutrition and Metabolic Care	Exclusion reason: Wrong setting
Susser, E.	Periconceptional folic acid and neurodevelopmental disorders: Historical context and current research	2013	Neuropsychopharmacology	Exclusion reason: Wrong study design
Sutapa, A., Fledderjohann, J., Sukumar, V., Stuckler, D.	Adequately diversified dietary intake and iron and folic acid supplementation during pregnancy is associated with reduced occurrence of symptoms suggestive of pre‐eclampsia or eclampsia in Indian women	2015	PLoS ONE [Electronic Resource]	Exclusion reason: Wrong intervention
Sutherland, T, Bishai, D. M.	Cost‐effectiveness of misoprostol and prenatal iron supplementation as maternal mortality interventions in home births in rural India (Provisional abstract)	2009	International Journal of Gynecological Cancer	Exclusion reason: Wrong intervention
Svefors, P., Selling, K. E., Shaheen, R. E., Persson, L. A. E., Lindholm, L.	Prenatal food and micronutrient interventions in rural Bangladesh remain cost‐effective when assessing both favorable and unfavorable outcomes: Costeffectiveness analysis of the MINIMat trial on under five‐mortality and stunting	2017	FASEB Journal. Conference: Experimental Biology	Exclusion reason: Wrong intervention
Sylvester, M.	Determination of the nutritional status of high‐risk antenatal women in St. Elizabeth, Jamaica	Exclusion reason: Wrong intervention		
Taghavi, N., Mollaian, M., Alizadeh, P., Moshref, M., Modabernia, S., Akbarzadeh, A. R.	Orofacial clefts and risk factors in Tehran, Iran: a case control study	2012	Iranian Red Crescent Medical Journal	Exclusion reason: Wrong intervention
Takimoto, H., Hayashi, F., Kusama, K., Kato, N., Yoshiike, N., Toba, M., Ishibashi, T., Miyasaka, N., Kubota, T.	Elevated maternal serum folate in the third trimester and reduced fetal growth: a longitudinal study	2011	Journal of Nutritional Science and Vitaminology	Exclusion reason: Wrong intervention
Tang, L., Lee, A. H., Yau, K. K. W., Hui, Y. V., Binns, C. W.	Consumption of dietary supplements by Chinese women during pregnancy and postpartum: A prospective cohort study	2017	Maternal and Child Nutrition	Exclusion reason: Wrong intervention
Tang, L., Lee, A. H., Yau, K. K. W., Hui, Y. V., Binns, C. W.	Consumption of dietary supplements by Chinese women during pregnancy and postpartum: a prospective cohort study	2017	Maternal and Child Nutrition	Exclusion reason: Wrong intervention
Tara, F., Maamouri, G., Rayman, M. P., Ghayour‐Mobarhan, M., Sahebkar, A., Yazarlu, O., Ouladan, S., Tavallaie, S., Azimi‐Nezhad, M., Shaken, M. T., Boskabadi, H., Oladi, M., Sangani, M. T., Razavi, B. S., Ferns, G.	Selenium supplementation and the incidence of preeclampsia in pregnant Iranian women: a randomized, double‐blind, placebo‐controlled pilot trial	2010	Taiwanese Journal of Obstetrics & Gynecology	Exclusion reason: Wrong intervention
Tara, F., Rayman, M. P., Boskabadi, H., Ghayour‐Mobarhan, M., Sahebkar, A., Alamdari, D. H., Razavi, B. S., Tavallaie, S., Azimi‐Nezhad, M., Shakeri, M. T., Oladi, M., Yazarlu, O., Ouladan, S., Sangani, M. T., Omran, F. R., Ferns, G.	Prooxidant‐antioxidant balance in pregnancy: a randomized double‐blind placebo‐controlled trial of selenium supplementation	2010	Journal of Perinatal Medicine	Exclusion reason: Wrong intervention
Tarcan, T., Onol, F. F.,; Tanidir, Y., Alpay, H., Ilker, Y., Simsek, F., Ozek, M.	Are myelodysplastic children receiving sufficient health care in Turkey? An analysis of the problems in primary management and their impact on neuro‐urological outcome	2007	Journal of Pediatric Urology	Exclusion reason: Wrong patient population
Tariku, A., Biks, G. A., Derso, T., Wassie, M. M., Abebe, S. M.	Stunting and its determinant factors among children aged 6‐59 months in Ethiopia	2017	Italian Journal of Pediatrics	Exclusion reason: Wrong patient population
Tatala, S. R., Ash, D., Makola, D., Latham, M., Ndosi, G., Grohn, Y.	Effect of micronutrient fortified beverage on nutritional anaemia during pregnancy	2002	East African Medical Journal	Exclusion reason: Wrong intervention
TCTR20170629006	Combined therapy with low dose aspirin and calcium supplements during second trimester to reduce the risk of superimposed preeclampsia in pregnant women with chronic hypertension: a randomized‐controlled trial	2017		Exclusion reason: Wrong patient population
Terefe, D., Zelalem, A., Amare, T.	Magnitude and associated factors of anaemia among pregnant women in Dera District: a cross‐sectional study in northwest Ethiopia	2017	BMC Research Notes	Exclusion reason: Wrong intervention
Thaler, J., Neugebauer, J., Wolf, J., Dakoure‐Ouedraogo, M., Kohler, H., Wessel, L., Zanre, Y., Wacker, J.	Effects of riboflavin given to pregnant women on the incidence of malaria: Results of a prospective randomized double blind study. [Article in German]	2006	Geburtshilfe und Frauenheilkunde	Exclusion reason: Wrong intervention
Thiruselvam, N., Cheong, S. W., Jagan, M., Paterson, J., Arcot, J.	Micronutrients fortification of rice by parboiling: lab scale and pilot scale studies	2014	Journal of Nutrition and Food Sciences	Exclusion reason: Wrong intervention
Thitasomakul, S., Piwat, S., Thearmontree, A., Chankanka, O., Pithpornchaiyakul, W., Madyusoh, S.	Risks for early childhood caries analyzed by negative binomial models	2009	Journal of dental research	Exclusion reason: Wrong patient population
Thomson, J.	Anaemia in pregnant women in eastern Caprivi, Namibia	1997	South African Medical Journal. Suid‐Afrikaanse Tydskrif Vir Geneeskunde	Exclusion reason: Wrong study design
Thorne‐Lyman, A. L., Fawzi, W. W.	Vitamin A and carotenoids during pregnancy and maternal, neonatal and infant health outcomes: a systematic review and meta‐analysis	2012	Paediatric and Perinatal Epidemiology	Exclusion reason: Wrong study design
Thorne‐Lyman, A., Fawzi, W. W.	Vitamin D during pregnancy and maternal, neonatal and infant health outcomes: a systematic review and meta‐analysis	2012	Paediatric and Perinatal Epidemiology	Exclusion reason: Wrong study design
Titaley, C. R., Dibley, M. J.	Antenatal iron/folic acid supplements, but not postnatal care, prevents neonatal deaths in Indonesia: Analysis of Indonesia Demographic and Health Surveys 2002/2003‐2007 (a retrospective cohort study)	2012	BMJ open	Exclusion reason: Wrong intervention
Titaley, C. R., Dibley, M. J., Roberts, C. L., Agho, K.	Combined iron/folic acid supplements and malaria prophylaxis reduce neonatal mortality in 19 sub‐Saharan African countries	2010	American Journal of Clinical Nutrition	Exclusion reason: Wrong intervention
Toivonen, K. I., Lacroix, E., Flynn, M., Ronksley, P. E., Oinonen, K. A., Metcalfe, A., Campbell, T. S.	Folic acid supplementation during the preconception period: A systematic review and meta‐analysis	2018	Preventive Medicine	Exclusion reason: Wrong study design
Tomkins, A.	Malnutrition, morbidity and mortality in children and their mothers	2000	Proceedings of the Nutrition Society	Exclusion reason: Wrong study design
Tomkins, A.	Nutrition and maternal morbidity and mortality	2001	British Journal of Nutrition	Exclusion reason: Wrong study design
Tondeur, M. C., Salse, U. N., Wilkinson, C., Spiegel, P., Seal, A. J.	Rapid acceptability and adherence testing of a lipid‐based nutrient supplement and a micronutrient powder among refugee children and pregnant and lactating women in Algeria	2016	Public Health Nutrition	Exclusion reason: Wrong patient population
Tran, T., Ho‐Pham, L., Do, M., To, P., Hirst, J., Nguyen, N., Nguyen, T.	Vitamin d insufficiency during pregnancy in vietnamese women	2013	Journal of Paediatrics and Child Health	Exclusion reason: Wrong intervention
Tripp, K., Perrine, C. G., de Campos, P., Knieriemen, M., Hartz, R., Ali, F., Jefferds, M. E. D., Kupka, R.	Formative research for the development of a market‐based home fortification programme for young children in Niger. (Special Issue: Consequences of malnutrition in early life and strategies to improve maternal and child diets through targeted fortified products.)	2011	Maternal and Child Nutrition	Exclusion reason: Wrong intervention
Tripp, K., Perrine, C. G., de Campos, P., Knieriemen, M., Hartz, R., Ali, F., Jefferds, M. E., Kupka, R.	Formative research for the development of a market‐based home fortification programme for young children in Niger	2011	Maternal & Child Nutrition	Exclusion reason: Wrong patient population
Trugo, N. M.	Micronutrient regulation in pregnant and lactating women from Rio de Janeiro	1997	Archivos latinoamericanos de nutricion	Exclusion reason: Wrong study design
Tsai, A. C., Wang, J.‐Y., Chang, T.‐L., Li, T.‐Y.	A comparison of the full Mini Nutritional Assessment, short‐form Mini Nutritional Assessment, and Subjective Global Assessment to predict the risk of protein‐energy malnutrition in patients on peritoneal dialysis: a cross‐sectional study	2013	International Journal of Nursing Studies	Exclusion reason: Wrong patient population
Tshibumbu, D. D., Blitz, J.	Modifiable antenatal risk factors for stillbirth amongst pregnant women in the Omusati Region, Namibia	2016	African Journal of Primary Health Care and Family Medicine	Exclusion reason: Wrong intervention
Tukan, S., Shalbak, M., Takruri, H., Zibdeh, M.	Dietary intake and serum concentrations of zinc, copper and iron in pregnant Jordanian women and their relation with pregnancy outcome	1997	Saudi Medical Journal	Exclusion reason: Wrong intervention
Tuncbilek, E.	The high incidence of neural tube defects in Turkey what should be done for prevention? [Turkish]	2004	Cocuk Sagligi ve Hastaliklari Dergisi	Exclusion reason: Wrong study design
Tuorkey, M. J., Abdul‐Aziz, K. K.	Strategies for diabetes and pathways of vitamin D	2010	Diabetes and Metabolic Syndrome: Clinical Research and Reviews	Exclusion reason: Wrong outcomes
Turk, T., Spohrer, R., Manus, C., Tran Khan, V., Tran Thuy, N., Nguyen, M., Poonawala, A., Garrett, G. S.	Using formative research to increase purchase intention of fortified foods to prevent micronutrient deficiencies in Vietnam	2016	Journal of Nutrition and Food Sciences	Exclusion reason: Wrong intervention
Tzagaraki, E., Sofocleous, C., Tounta, G., Mavrou, A., Kolialexi, A., Kucharski, H., Zajac, J.	The role of vitamin C in human reproduction. (Nutrition and Diet Research Progress Series)	2009		Exclusion reason: Wrong intervention
Ullah, B., Mridha, M., Arnold, C. D., Matias, S., Khan, S., Siddiki, Z., Hossain, M., Dewey, K. G.	Effect of pre‐and postnatal nutritional supplements on childhood illnesses in Bangladesh: A cluster‐randomized effectiveness trial	2017	Annals of Nutrition and Metabolism	Exclusion reason: Wrong patient population
Umesh, K., Neha, S.	Combating iodine deficiency disorders to achieve millennium development goal 4 in India: reduction in infant mortality rate	2012	Journal of Trace Elements in Medicine and Biology	Exclusion reason: Wrong patient population
Underwood, B. A., Arthur, P.	The contribution of vitamin A to public health	1996	FASEB Journal	Exclusion reason: Wrong study design
Underwood, B. A., Smitasiri, S.	Micronutrient malnutrition: policies and programs for control and their implications	1999	Annual Review of Nutrition	Exclusion reason: Wrong study design
UNICEF, World Health Organization,United Nations University workshop	Composition of a multi‐micronutrient supplement to be used in pilot programmes among pregnant women in developing countries: report of a United Nations Children's Fund (UNICEF), World Health Organization (WHO) and United Nations University workshop	1999		Exclusion reason: Wrong study design
Uriu‐Adams, J. Y., Chambers, C. D., Gross, H. B., Ensunsa, J. L., Green, K., Le, A., Yevtushok, L., Zymak‐Zakutnya, N., Wertelecki, W.	Alcohol drinking patterns and nutrient status in Ukrainian pregnant women	2010	Birth Defects Research Part A ‐ Clinical and Molecular Teratology	Exclusion reason: Wrong intervention
Ursu, H., Gheorghiu, M. L., Dumitrescu, I., Stanciu, M., Popescu, D., Delia, C. E., Toma, G. M., Aldea, R., Lichiardopol, C. R., Tudorache, S., Vasile, M., Podia‐Igna, C., Georgescu, C. E., Purice, M.	Preliminary results of a multicentric study of urinary iodine concentration in pregnant women from Romania	2016	European Thyroid Journal	Exclusion reason: Wrong intervention
Utari, D. M., Achadi, E. L., Pujonarti, S. A., Salimar	Impact of weekly versus daily iron‐folic acid supplementation for pregnant women with anaemia on hemoglobin levels, clinical symptoms and subjective complaints	2017	Pakistan Journal of Nutrition	Exclusion reason: Wrong patient population
Vadillo‐Ortega, F., Perichart‐Perera, O., Espino, S., Avila‐Vergara, M. A., Ibarra, I., Ahued, R., Godines, M., Parry, S., Macones, G., Yanow, M., Yanow, E., Strauss, J. F.	Effect of supplementation during pregnancy with L‐arginine and antioxidant vitamins in medical food on pre‐eclampsia in high risk population: Randomised controlled trial	2011	Bmj	Exclusion reason: Wrong patient population
Vaivada, T., Gaffey, M. F., Das, J. K., Bhutta, Z. A.	Evidence‐based interventions for improvement of maternal and child nutrition in low‐income settings: what's new?	2017	Current Opinion in Clinical Nutrition and Metabolic Care	Exclusion reason: Wrong study design
Van Bogaert, L. J.	Anaemia and pregnancy outcomes in a South African rural population	2006	Journal of Obstetrics and Gynaecology	Exclusion reason: Wrong intervention
van den Broek, N., Dou, L., Othman, M., Neilson, J. P., Gates, S., Gulmezoglu, A. M.	Vitamin A supplementation during pregnancy for maternal and newborn outcomes	2010	Cochrane Database of Systematic Reviews	Exclusion reason: Wrong study design
van den Broek, N., Kulier, R., Gulmezoglu, A. M., Villar, J.	WITHDRAWN: Vitamin A supplementation during pregnancy	2010	Cochrane Database of Systematic Reviews	Exclusion reason: Wrong study design
Van Dillen, J., Stekelenburg, J., Van Roosmalen, J.	The UN Millennium Project; especially the prevention and treatment of the HIV‐virus and AIDS in order to reduce child and maternal mortality. [Article in Dutch]	2006	Nederlands Tijdschrift voor Geneeskunde	Exclusion reason: Wrong study design
van Eijsden, M., Hornstra, G., van der Wal, M. F., Bonsel, G. J.	Ethnic differences in early pregnancy maternal n‐3 and n‐6 fatty acid concentrations: an explorative analysis	2009	British Journal of Nutrition	Exclusion reason: Wrong study design
Van, D. E., Kulier, R., Gulmezoglu, A. M., Villar, J.	Vitamin A supplementation during pregnancy	2002	Cochrane Database of Systematic Reviews	Exclusion reason: Wrong study design
Veghari, G.	Iron supplementation during pregnancy and birth weight in Iran: a retrospective study	2009	Pakistan Journal of Biological Sciences	Exclusion reason: Wrong intervention
Veghari, G., Marjani, A., Rahmati, R., Hosseini	The state of birth weight in the north of Iran	2009	Journal of Clinical and Diagnostic Research	Exclusion reason: Wrong intervention
Venkatarao, T., Ramakrishnan, R., Nair, N. G. K., Radhakrishnan, S., Sundaramoorthy, L., Mohammad Koya, P. K., Suresh Kumar, S. K.	Effect of vitamin A supplementation to mother and infant on morbidity in infancy	1996	Indian Pediatrics	Exclusion reason: Wrong patient population
Verguet, S., Jassat, W., Bertram, M. Y., Tollman, S. M., Murray, C. J. L., Jamison, D. T., Hofman, K. J.	Impact of supplemental immunisation activity (SIA) campaigns on health systems: findings from South Africa	2013	Journal of Epidemiology & Community Health	Exclusion reason: Wrong outcomes
Villamor, E., Msamanga, G., Spiegelman, D., Antelman, G., Peterson, K. E., Hunter, D. J., Fawzi, W. W.	Effect of multivitamin and vitamin A supplements on weight gain during pregnancy among HIV‐1‐infected women	2002	American Journal of Clinical Nutrition	Exclusion reason: Wrong patient population
Villamor, E., Saathoff, E., Bosch, R. J., Hertzmark, E., Baylin, A., Manji, K., Msamanga, G., Hunter, D. J., Fawzi, W. W.	Vitamin supplementation of HIV‐infected women improves postnatal child growth	2005	American Journal of Clinical Nutrition	Exclusion reason: Wrong patient population
Villar, J., Abalos, E., Nardin, J. M., Merialdi, M., Carroli, G.	Strategies to prevent and treat preeclampsia: evidence from randomized controlled trials	2004	Seminars in Nephrology	Exclusion reason: Wrong study design
Villar, J., Bergsjo, P.	Scientific basis for the content of routine antenatal care. I. Philosophy, recent studies, and power to eliminate or alleviate adverse maternal outcomes	1997	Acta Obstetricia et Gynecologica Scandinavica	Exclusion reason: Wrong study design
Villar, J., Purwar, M., Merialdi, M., Zavaleta, N., Ngoc, N. T. N., Anthony, J., de Greeff, A., Poston, L., Shennan, A.	World Health Organisation multicentre randomised trial of supplementation with vitamins C and E among pregnant women at high risk for pre‐eclampsia in populations of low nutritional status from developing countries	2009	BJOG: An International Journal of Obstetrics and Gynaecology	Exclusion reason: Wrong patient population
Viswanathan, M., Treiman, K. A., Doto, J. K., Middleton, J. C., Coker‐Schwimmer, E. J. L., Nicholson, W. K.	2017	Agency for Healthcare Research and Quality	Exclusion reason: Wrong study design	
Viteri, F. E.	A new concept in the control of iron deficiency: community‐based preventive supplementation of at‐risk groups by the weekly intake of iron supplements	1998	Biomedical & Environmental Sciences	Exclusion reason: Wrong study design
Viteri, F. E.	Iron supplementation as a strategy for the control of iron deficiency and ferropenic anaemia	1999	Archivos Latinoamericanos de Nutricion	Exclusion reason: Wrong study design
Vrzhesinskaya, O. A., Kodentsova, V. M., Pereverzeva, O. G., Gmoshinskaya, M. V., Pustograev, N. N.	Evaluation of sufficiency with vitamins C, B1 and B2 of newborn infants feeding different types of nutrition, by means of urinary excretion determination	2015	Voprosy Pitaniya	Exclusion reason: Wrong patient population
Vrzhesinskaya, O. A., Pereverzeva, O. G., Gmoshinskaya, M. V., Kodentsova, V. M., Safronova, A. I., Korosteleva, M. M., Aleshina, I. V., Fandeeva, T. A.	Sufficiency with water‐soluble vitamins and state of bone in pregnant women	2015	Voprosy Pitaniya	Exclusion reason: Wrong intervention
Wahed, F., Latif, S., Uddin, M., Mahmud, M.	Fact of low hemoglobin and packed cell volume in pregnant women are at a stand still	2008	Mymensingh Medical Journal: MMJ	Exclusion reason: Wrong study design
Wald, N. J.	Folic Acid and the Prevention of Neural‐Tube Defects	2004	New England Journal of Medicine	Exclusion reason: Wrong study design

**Table 14:** Characteristics table of excluded studies (Database Searches)

Authors	Title	Published Year	Journal	Exclusion Reason
Wald, N. J., Hackshaw, A. D., Stone, R., Sourial, N. A.	Blood folic acid and vitamin B12 in relation to neural tube defects	1996	British Journal of Obstetrics & Gynaecology	Exclusion reason: Wrong intervention
Wang, A., Keen, C. L.	Micronutrient Status in Pregnant Women who are at High Risk for Having Children with Fetal Alcohol Spectrum Disorder	2013		Exclusion reason: Wrong intervention
Wang, B. L., Pang, Q. X., Liu, H., Zhang, J., Li, Y. D., Hou, A. Q.	Correlation analysis between calcium content in pregnant women and gestational hypertension	2013	Progress in Modern Biomedicine	Exclusion reason: Wrong intervention
Wang, H., Fan, Y. F., Cai, L. Q.	Effect of nutritional evaluation and intervention measures during pregnancy on pregnancy outcome	2010	Maternal and Child Health Care of China	Exclusion reason: Wrong intervention
Wang, J. S., Wang, L. Y., An, M. J., Li, Y. H., Yang, N., Wu, J. H., Wang, R. J., Cui, L. H., Pang, S. L., Guan, W. J., Wang, J., Xue, L.	The investigation and analysis of risk factors of children type 1 diabetes mellitus	2016	Modern Preventive Medicine	Exclusion reason: Wrong patient population
Wang, M., Wang, Z. P., Gao, L. J., Gong, R., Zhang, M., Lu, Q. B., Zhao, Z. T.	Periconceptional factors affect the risk of neural tube defects in offspring: a hospital‐based case‐control study in China	2013	Journal of Maternal‐Fetal & Neonatal Medicine	Exclusion reason: Wrong intervention
Wang, M., Wang, Z. P., Gao, L. J., Gong, R. Sun, X. H., Zhao, Z. T.	Maternal body mass index and the association between folic acid supplements and neural tube defects	2013	Acta Paediatrica	Exclusion reason: Wrong intervention
Wang, S., Ge, X., Zhu, B., Xuan, Y., Huang, K., Rutayisire, E., Mao, L., Huang, S., Yan, S., Tao, F.	Maternal continuing folic acid supplementation after the first trimester of pregnancy increased the risk of large‐for‐gestational‐age birth: A population‐based birth cohort study	2016	Nutrients	Exclusion reason: Wrong intervention
Wang, S., Pan, X. J., Yu, Z. L.	A cohort study on the relationship between serum folic acid of pregnant women during the first trimester of pregnancy and onset of fetal birth defects	2012	Maternal and Child Health Care of China	Exclusion reason: Wrong intervention
Wang, S. F., Ge, X., Zhu, B. B., Xuan, Y. J., Huang, K., Rutayisire, E., Mao, L. J., Huang, S. H., Yan, S. Q., Tao, F. B.	Maternal continuing folic acid supplementation after the first trimester of pregnancy increased the risk of large‐for‐gestational‐age birth: a population‐based birth cohort study	2016	Nutrients	Exclusion reason: Wrong intervention
Wang, Y. Z., Ren, W. H., Liao, W. Q., Zhang, G. Y.	Concentrations of antioxidant vitamins in maternal and cord serum and their effect on birth outcomes	2009	Journal of Nutritional Science & Vitaminology	Exclusion reason: Wrong intervention
Wang, Y., Zhao, N., Qiu, J., He, X., Zhou, M., Cui, H., Lv, L., Lin, X., Zhang, C., Zhang, H., Xu, R., Zhu, D., Dang, Y., Han, X., Zhang, H., Bai, H., Chen, Y., Tang, Z., Lin, R., Yao, T., Su, J., Xu, X., Liu, X., Wang, W., Ma, B., Liu, S.	Folic acid supplementation and dietary folate intake, and risk of preeclampsia	2015	European Journal of Clinical Nutrition	Exclusion reason: Wrong intervention
Wani, M. A.	Neural tube defect and folic acid	2000	Jk Practitioner	Exclusion reason: Wrong study design
Weaver, E. H., Gibbons, L., Belizan, J. M., Althabe, F.	The increasing trend in preterm birth in public hospitals in northern Argentina	2015	International Journal of Gynaecology & Obstetrics	Exclusion reason: Wrong intervention
Wen, S. W., Guo, Y. F., Rodger, M., White, R. R., Yang, Q. Y., Smith, G. N., Perkins, S. L., Walker, M. C.	Folic acid supplementation in pregnancy and the risk of pre‐eclampsia ‐ a cohort study	2016	PLoS ONE [Electronic Resource]	Exclusion reason: Wrong patient population
Werler, M. M., Mitchell, A. A.	Neural‐tube defects	2000	The New England journal of medicine	Exclusion reason: Wrong patient population, no abstract
West Jr, K. P.	Vitamin A deficiency as a preventable cause of maternal mortality in undernourished societies: Plausibility and next steps	2004	International Journal of Gynecology and Obstetrics	Exclusion reason: Wrong study design
West Jr, K. P., Christian, P.	Antenatal micronutrients in undernourished people	2008	The Lancet	Exclusion reason: Wrong study design
West, K. P., Jr.	Vitamin A deficiency disorders in children and women	2003	Food & Nutrition Bulletin	Exclusion reason: Wrong study design
West, K. P., Jr.	Vitamin A deficiency disorders in children and women. (Proceedings of the Colloquim "Unlocking the Potential of the World's Children through Sustainable Fortification and Public‐Private Partnership", Cincinnati, Ohio, USA, 10‐11 October, 2002.)	2003	Food and nutrition bulletin	Exclusion reason: Wrong study design
West, K. P., Christian, P., Katz, J., Labrique, A., Klemm, R., Sommer, A.	Effect of vitamin A supplementation on maternal survival	2010	Lancet	Exclusion reason: Wrong study design
Wibowo, N., Bardosono, S., Irwinda, R.	Effects of bifidobacterium animalis lactis HN019 (DR10TM), inulin, and micronutrient fortified milk on faecal DR10TM, immune markers, and maternal micro‐nutrients among Indonesian pregnant women. (Special Issue: Advancing clinical nutrition in Indonesia.)	2016	Asia Pacific Journal of Clinical Nutrition	Exclusion reason: Wrong intervention
Wibowo, N., Irwinda, R.	The effect of multi‐micronutrient and protein supplementation on iron and micronutrients status in pregnant women	2015	Medical Journal Of Indonesia	Exclusion reason: Wrong intervention
Wibowo, N., Purwosunu, Y., Sekizawa, A., Farina, A., Tambunan, V., Bardosono, S.	Vitamin B6 supplementation in pregnant women with nausea and vomiting	2012	International Journal of Gynecology & Obstetrics	Exclusion reason: Wrong intervention
Wilunda, C., Massawe, S., Jackson, C.	Determinants of moderate‐to‐severe anaemia among women of reproductive age in Tanzania: analysis of data from the 2010 Tanzania demographic and health survey	2013	Tropical Medicine and International Health	Exclusion reason: Wrong intervention
Wilunda, C., Tanaka, S., Esamai, F., Kawakami, K.	Prenatal anaemia control and anaemia in children aged 6‐23 months in sub‐Saharan Africa	2017	Maternal and Child Nutrition	Exclusion reason: Wrong patient population
Winje, B. A., Kvestad, I., Krishnamachari, S., Manji, K., Taneja, S., Bellinger, D. C., Bhandari, N., Bisht, S., Darling, A. M., Duggan, C. P., Fawzi, W., Hysing, M., Kumar, T., Kurpad, A. V., Sudfeld, C. R., Svensen, E., Thomas, S., Strand, T. A.	Does early vitamin B12 supplementation improve neurodevelopment and cognitive function in childhood and into school age: a study protocol for extended follow‐ups from randomised controlled trials in India and Tanzania	2018	BMJ open	Exclusion reason: Wrong patient population
Winkvist, A., Habicht, J. P., Rasmussen, K. M.	Linking maternal and infant benefits of a nutritional supplement during pregnancy and lactation	1998	American Journal of Clinical Nutrition	Exclusion reason: Wrong intervention;
Wrottesley, S. V., Lamper, C., Pisa, P. T.	Review of the importance of nutrition during the first 1000 days: maternal nutritional status and its associations with fetal growth and birth, neonatal and infant outcomes among African women	2016	Journal of Developmental Origins of Health and Disease	Exclusion reason: Wrong intervention
Wu, J., Zheng, Q., Huang, Y. Q., Wang, Y., Li, S., Lu, D. W., Shi, B., Chen, H. Q.	Significant evidence of association between polymorphisms in ZNF533, environmental factors, and nonsyndromic orofacial clefts in the western han Chinese population	2011	DNA and Cell Biology	Exclusion reason: Wrong intervention
Wu, Z. C., Chijang, C. C., Lau, B. H., Hwang, B., Sugawara, M., Idota, T.	Crude protein content and amino acid composition in Taiwanese human milk	Exclusion reason: Wrong intervention		
Xing, X. Y., Tao, F. B., Hao, J. H., Huang, K., Huang, Z. H., Zhu, X. M., Xiao, L. M., Cheng, D. J., Su, P. Y., Zhu, P., Xu, Y. Y., Sun, Y.	Periconceptional folic acid supplementation among women attending antenatal clinic in Anhui, China: data from a population‐based cohort study	2012	Midwifery	Exclusion reason: Wrong intervention
Xu, H., Perez‐Cuevas, R., Xiong, X., Reyes, H., Julien, P., Smith, G., Choquette, P., Winsor, S., Leduc, L., Audibert, F., Moutquin, J. M., Wood, S., Benjamin, A., Walker, M., Helewa, M., Dube, J., Tawagi, G., Seaward, G., Ohlsson, A., Von Dadelszen, P., Macgee, L., Olatunbosun, F., Piedboeuf, B., Gratton, R., Shearman, R., Demianczuk, N., Collet, J. P., Roy, C., Fraser, W.	An international trial of vitamins C and E in the prevention of preeclampsia (INTAPP trial)	2009	American Journal of Obstetrics and Gynecology	Exclusion reason: Wrong intervention
Xu, H., Zhong‐Cheng, L., Shatenstein, B., Fraser, W.	Periconceptional nutrient intake and the risk of hypertensive disorder during pregnancy‐a binational perspective	2010	American Journal of Epidemiology	Exclusion reason: Wrong intervention
Xu, H.	Maternal Nutrition and The Risk of Preeclampsia	2011		Exclusion reason: Wrong intervention
Xu, L. F., Zhou, X. L., Wang, Q., Zhou, J. L., Liu, Y. P., Ju, Q., Wang, H., Zhang, J. P., Wu, Q. R., Li, Y. Q., Xia, Y. J., Peng, X., Zhang, M. R., Yu, H. M., Xu, L. C.	A Case‐control Study of Environmental Risk Factors for Nonsyndromic Cleft of the Lip and/or Palate in Xuzhou, China	2015	Biomedical & Environmental Sciences	Exclusion reason: Wrong intervention
Yabes‐Almirante, C.	Calcium supplementation in pregnancy to prevent pregnancy induced hypertension (PIH)	1998	Journal of Perinatal Medicine	Exclusion reason: Wrong study design
Yadav, K., Srivastava, R., Badhal, S., Palanivel, C., Pandav, C. S., Karmarkar, M. G.	Iodine nutrition of pregnant women in India: evidence of significant iodine deficiency	2012	Indian Journal of Medical Specialities	Exclusion reason: Wrong study design
Yajnik, C.	Nutritional control of fetal growth	2006	Nutrition Reviews	Exclusion reason: Wrong study design
Yakoob, M. Y., Bhutta, Z. A.	Effect of routine iron supplementation with or without folic acid on anaemia during pregnancy	2011	BMC Public Health	Exclusion reason: Wrong study design
Yakoob, M. Y., Menezes, E. V., Soomro, T., Haws, R. A., Darmstadt, G. L., Bhutta, Z. A.	Reducing stillbirths: Behavioural and nutritional interventions before and during pregnancy	2009	BMC Pregnancy and Childbirth	Exclusion reason: Wrong study design
Yakoob, M. Y., Theodoratou, E., Afshan, J., Aamer, I., Eisele, T. P., Ferguson, J., Jhass, A., Rudan, I., Campbell, H., Black, R. E., Bhutta, Z. A.	Preventive zinc supplementation in developing countries: impact on mortality and morbidity due to diarrhea, pneumonia and malaria. (Special Issue: Technical inputs, enhancements and applications of the Lives Saved Tool (LiST).)	2011	BMC Public Health	Exclusion reason: Wrong study design
Yakout, S. M., Taha, N., Badawy, A. S., Al‐Salooly, H. A.	Effect of iron supplementation and nutritional education among a group of anemic pregnant women on their perinatal outcome in Riyadh	2014	Journal of Current Research in Science	Exclusion reason: Wrong patient population
Yalçın, S. S., Tezel, B., Yurdakök K., Pekcan, G., Ozbas, S., Köksal, E., Tunç, B., Sahinli, S., Altunsu, A. T., Köse, M. R., Buzgan, T., Akdag, R.	A community‐based iron supplementation program, “Iron‐Like Turkey”, and the following prevalence of anaemia among infants aged 12‐23 months	2013	Turk J Pediatr	Exclusion reason: Wrong patient population
Yan, J., Liu, Y., Cao, L., Zheng, Y., Li, W., Huang, G.	Association between duration of folic acid supplementation during pregnancy and risk of postpartum depression	2017	Nutrients	Exclusion reason: Wrong patient population
Yang, J. M., Cheng, Y., Pei, L. L., Jiang, Y. F., Lei, F. L., Zeng, L. X., Wang, Q. L., Li, Q., Kang, Y. J., Shen, Y., Dang, A. N., Yan, H.	Maternal iron intake during pregnancy and birth outcomes: a cross‐sectional study in Northwest China	2017	British Journal of Nutrition	Exclusion reason: Wrong intervention
Yang, T., Gu, Y., Wei, X., Liang, X., Chen, J., Liu, Y., Zhang, T., Li, T.	Periconceptional folic acid supplementation and Vitamin B_12_ status in a cohort of Chinese early pregnancy women with the risk of adverse pregnancy outcomes	2017	Journal of Clinical Biochemistry and Nutrition	Exclusion reason: Wrong intervention
Yang, Y. X., Chen, X. C., Liu, J. Y., Pan, L. M., Yan, H. C., Xu, Q. M.	Effect of zinc intake on fetal and infant growth among Chinese pregnant and lactating women	Exclusion reason: Wrong intervention		
Yang, Z., Huffman, S. L.	Modelling linoleic acid and Î±‐linolenic acid requirements for infants and young children in developing countries	2013	Maternal & Child Nutrition	Exclusion reason: Wrong patient population
Yang, Z., Huffman, S. L.	Nutrition in pregnancy and early childhood and associations with obesity in developing countries	2013	Maternal & Child Nutrition	Exclusion reason: Wrong study design
Yared, W.	Vitamin B6 status of pregnant women attending antenatal clinic in northwestern Ethiopia	2005	Ethiopian Journal of Health Development	Exclusion reason: Wrong intervention
Yasir, N., Dibley, M. J., Aguayo, V. M.	Iron‐folic acid supplementation during pregnancy reduces the risk of stunting in children less than 2 years of age: a retrospective cohort study from Nepal	2016	Nutrients	Exclusion reason: Wrong patient population
Yasmin, S.	Neonatal outcome of low birth weight infants in Bangladesh	1998		Exclusion reason: Wrong patient population
Ye, R. W., Ren, A. G., Zhang, L., Li, Z. W., Liu, J. M., Pei, L. J., Zheng, X. Y.	Tea drinking as a risk factor for neural tube defects in Northern China	2011	Epidemiology	Exclusion reason: Wrong intervention
Yin, Y., Zhang, T., Dai, Y., Zheng, X., Pei, L.,Lu, X.	Pilot study of association of anembryonic pregnancy with 55 elements in the urine, and serum level of folate, homocysteine and S‐adenosylhomocysteine in Shanxi Province, China	2009	Journal of the American College of Nutrition	Exclusion reason: Wrong intervention
Yin, Z., Xu, W., Xu, C., Zhang, S., Zheng, Y., Wang, W., Zhou, B.	A population‐based case‐control study of risk factors for neural tube defects in Shenyang, China	2011	Childs Nervous System	Exclusion reason: Wrong intervention
Yip, R.	Prevention and control of iron deficiency in developing countries	1996	Current Issues in Public Health	Exclusion reason: Wrong study design; Lit Review
Yirga, E., Mussie, A., Mengistu, M., Goba, G. K.	Determinants of severe anaemia among laboring mothers in Mekelle city public hospitals, Tigray region, Ethiopia	2017	PLoS ONE [Electronic Resource]	Exclusion reason: Wrong intervention
Young, M. W., Lupafya, E., Kapenda, E., Bobrow, E. A.	Folic acid and the prevention of disease. Report of the Committee on Medical Aspects of Food and Nutrition Policy The effectiveness of weekly iron supplementation in pregnant women of rural northern Malawi	Exclusion reason: Wrong study design		
Young, S. L., Blanco, I., Hernandez‐Cordero, S., Pelto, G. H., Neufeld, L. M.	Organoleptic properties, ease of use, and perceived health effects are determinants of acceptability of micronutrient supplements among poor Mexican women	2010	Journal of Nutrition	Exclusion reason: Wrong outcomes
Young, T.	Effects of micronutrient supplementation on morbidity and mortality among HIV‐infected individuals ‐ a summary of the evidence	2006	S Afr Med J	Exclusion reason: Wrong study design
Youssef, M. A., Abdelmoty, H. I., Elashmwi, H, A., Abduljawad, E. M., Elghamary, N., Magdy, A, Mohesen, M. N., Abdella, R. M., Bar, M. A., Gouda, H. M., Ali, A. M., Raslan, A. N., Youssef, D. Sherif, N. A., Ismail, A. I.	Oral antioxidants supplementation for women with unexplained infertility undergoing ICSI/IVF: randomized controlled trial	2015	Human fertility (Cambridge)	Exclusion reason: Wrong patient population
Yu, R., Li, Y. Y.	The clinical observation of the effects of maternal serum vitamin D levels on the developing nervous system in infants	2015	Chongqing Medicine	Exclusion reason: Wrong patient population
Yu, X. D., Wang, W. Y., Wei, Z. Z., Ouyang, F. X., Huang, L. S., Wang, X., Zhao, Y. J., Zhang, H. J., Zhang, J.	Vitamin D status and related factors in newborns in Shanghai, China	2014	Nutrients	Exclusion reason: Wrong intervention
Yuan, Y., Zhang, L., Jin, L., Liu, J., Li, Z., Wang, L., Ren, A.	Markers of macromolecular oxidative damage in maternal serum and risk of neural tube defects in offspring	2015	Free Radic Biol Med	Exclusion reason: Wrong intervention
Yue, H. N., Liu, S., Gao, Y. L., Yuan, Y. H., Ji, J. L., Han, J. Q.	Effect of intervention to the blood lead level of pregnant women on the early intellectual development of infants. [Article in Chinese]	2005	Chinese Journal of Clinical Rehabilitation	Exclusion reason: Wrong patient population
Yun, C. F., Chen, J., He, Y. N., Mao, D. Q., Wang, R., Zhang, Y., Yang, C., Piao, J. H., Yang, X. G.,	Vitamin D deficiency prevalence and risk factors among pregnant Chinese women	2017	Public Health Nutrition	Exclusion reason: Wrong intervention
Yuskiv, N., Honein, M. A., Moore, C. A.	Reported multivitamin consumption and the occurrence of multiple congenital anomalies	2005	American Journal of Medical Genetics Part A	Exclusion reason: Wrong intervention
Zachara, B. A.	Selenium in pregnant women: mini review	2016	Journal of Nutrition and Food Sciences	Exclusion reason: Wrong intervention
Zeisel, S. H.	Nutrition in pregnancy: The argument for including a source of choline	2013	International Journal of Women's Health	Exclusion reason: Wrong study design
Zeng, L., Yan, H., Chen, Z.	Measurement of the living standards of family in rural area and relationship between wealth index and perinatal care status [Article in Chinese]	2008	Wei Sheng Yen Chiu/Journal of Hygiene Research	Exclusion reason: Wrong study design
Zeng, L, Yan, H, Cheng, Y, Dang, S, Dibley, M. J.	Adherence and costs of micronutrient supplementation in pregnancy in a double‐blind, randomized, controlled trial in rural western China	2009	Food and nutrition bulletin	Exclusion reason: Wrong outcomes
Zhang, L., Ren, A. G., Li, Z. W., Hao, L., Tian, Y. H., Li, Z.	Plasma and red blood cell folate levels among women in their first trimester of pregnancy from rural areas with high or low prevalence of neural tube defects, China. [Article in Chinese]	2006	Zhonghua Liu Xing Bing Xue Za Zhi	Exclusion reason: Wrong study design
Zhang, L., Ren, A., Li, Z., Hao, L., Tian, Y., Li, Z.	Folate concentrations and folic acid supplementation among women in their first trimester of pregnancy in a rural area with a high prevalence of neural tube defects in Shanxi, China	2006	Birth Defects Research	Exclusion reason: Wrong intervention
Zhang, Q. Y., Li, Z., Ananth, C. V.	Prevalence and risk factors for anaemia in pregnant women: a population‐based prospective cohort study in China	2009	Paediatric and Perinatal Epidemiology	Exclusion reason: Wrong intervention
Zhang, Y., Lin, L., Cao, Y., Chen, B., Zheng, L., Ge, R. S.	Phthalate levels and low birth weight: a nested case‐control study of Chinese newborns	2009	Journal of Pediatrics	Exclusion reason: Wrong patient population
Zhang, Y., Yang, Y. L., Hasegawa, Y., Yamaguchi, S., Shi, C. Y., Song, J. Q., Sawami, S., Liu, P., Yan, R., Dong, J. H., Qin, J.	Prenatal diagnosis of methylmalonic aciduria by analysis of organic acids and total homocysteine in amniotic fluid	2008	Chinese Medical Journal	Exclusion reason: Wrong intervention
Zhao, L. Y., Yu, D.‐M., Huang, J., Zhao, X.‐F., Li, J. W., Du, W.‐W., Yu, W.‐T., Su, C., Yin, S.‐A.	The nutrition status of special population living in the areas affected by Wenchuan Earthquake after 3 months [Article in Chinese]	2010	Zhonghua Yu Fang Yi Xue Za Zhi	Exclusion reason: Wrong patient population
Zheng, J. S., Guan, Y., Zhao, Y., Zhao, W., Tang, X., Chen, H., Xu, M., Wu, L., Zhu, S., Liu, H., Huang, T., Li, D.	Pre‐conceptional intake of folic acid supplements is inversely associated with risk of preterm birth and small‐for‐gestational‐age birth a prospective cohort study	2015	British Journal of Nutrition	Exclusion reason: Wrong intervention
Zheng, J. S., Guan, Y. H., Zhao, Y. M., Zhao, W., Tang, X. J., Chen, H., Xu, M. L., Wu, L. P., Zhu, S. L., Liu, H. J., Huang, T., Li, D.	Pre‐conceptional intake of folic acid supplements is inversely associated with risk of preterm birth and small‐for‐gestational‐age birth: a prospective cohort study	2016	British Journal of Nutrition	Exclusion reason: Wrong intervention
Zhou, Q., Wang, Q., Shen, H., Tian, W., Dong, X., Zhang, S., Li, X.	Effects of preconception risk intervention for the prevention of fetal congenital heart defect in Chinese rural areas: Community‐based cross sectional study	2015	International Journal of Gynecology and Obstetrics	Exclusion reason: Wrong intervention
Zhou, X. J., Jiang, Q. J., Li, X. N.	Status quo of iron supplementation before pregnancy and during pregnancy and its influence on anaemia in pregnant and lying‐in women	2013	Chongqing Medicine	Exclusion reason: Wrong intervention
Zhu, B. B., Ge, X., Huang, K., Mao, L. J., Yan, A. Q., Xu, Y. Q., Huang, S. H., Hao, J. H., Zhu, P., Niu, Y., Tong, S. L., Tao, F. B.	Folic acid supplement intake in early pregnancy increases risk of gestational diabetes mellitus: evidence from a prospective cohort study	2016	Diabetes Care	Exclusion reason: Wrong study design
Zhu, H., Yang, Z. J., Hui, N., Liu, J. X.	Zinc supplementation during pregnancy against mercury toxicity	2014	Academic Journal of Second Military Medical University	Exclusion reason: Wrong patient population
Zhu, L., Ling, H.	National neural tube defects prevention program in China	2008	Food and nutrition bulletin	Exclusion reason: Wrong study design
Zhu, P., Tong, S.‐L., Hao, J.‐H., Tao, R.‐X., Huang, K., Hu, W.‐B., Zhou, Q.‐F., Jiang, X.‐M., Tao, F.‐B.,	Cord blood vitamin d and neurocognitive development are nonlinearly related in toddlers	2015	Journal of Nutrition	Exclusion reason: Wrong patient population
Zhu, Z., Cheng, Y., Yang, W., Li, D., Yang, X., Liu, D., Zhang, M., Yan, H., Zeng, L.	Who Should Be Targeted for the Prevention of Birth Defects? A Latent Class Analysis Based on a Large, Population‐Based, Cross‐Sectional Study in Shaanxi Province, Western China	2016	PLoS ONE	Exclusion reason: Wrong intervention
Zima, T., Springer, D., Loucky, J.	Recommendations in prenatal screening in the world and connections to other diseases like thyropathy	2011	Clinical Chemistry and Laboratory Medicine	Exclusion reason: Wrong study design
Zimmermann, M. B., Hurrell, R. F.	Nutritional iron deficiency	2007	Lancet	Exclusion reason: Wrong study design
Zylke, J. W., Rivara, F. P., Bauchner, H.	Contrasts in child health care and child health research	2013	JAMA ‐ Journal of the American Medical Association	Exclusion reason: Wrong study design
	Bulletin	2010	Midwifery News	Exclusion reason: Wrong study design
	Clinical Pharmacology and Therapeutics			Exclusion reason: Wrong study design
	Dramatic changes in Kenya	1995	Newsl Macro Syst Inst Resour Dev Demogr Health Surv	Exclusion reason: Wrong study design
	Executive summary	2008	South African Journal of Clinical Nutrition	Exclusion reason: Wrong study design
	From the editor. Facts and ideas from anywhere	2008	Baylor University Medical Center Proceedings	Exclusion reason: Wrong study design
	Guideline: use of multiple micronutrient powders for home fortification of foods consumed by pregnant women. (In IRIS)	2011		Exclusion reason: Wrong study design
	Guideline: vitamin A supplementation in infants and children 6‐59 months of age	2011		Exclusion reason: Wrong study design
	Guideline: vitamin A supplementation in pregnant women	2011		Exclusion reason: Wrong study design
	Health and dietary facts. 2nd International Workshop on Micronutrients and Child Health, New Delhi, India, 3‐7 November 2014	2014	Indian Journal of Community Health	Exclusion reason: Wrong study design
	News	2010	Neonatal Intensive Care	Exclusion reason: Wrong study design

**Table 15:** Characteristics table of excluded studies (Database Searches)

Authors	Title	Published Year	Journal	Exclusion Reason
	New in review. Periodicals	2006	Journal of the American Dietetic Association	Exclusion reason: Wrong study design
	Oral Presentations (pp. 113‐124)	2009	Annals of Nutrition & Metabolism	Exclusion reason: Wrong study design
	Oral Presentations (pp. 94‐112)	2009	Annals of Nutrition & Metabolism	Exclusion reason: Wrong study design
	Poster Presentations Part II (pp. 222‐237)	2009	Annals of Nutrition & Metabolism	Exclusion reason: Wrong study design
	Symposium on iron deficiency in Indonesia	1998	Nutrition Research	Exclusion reason: Wrong study design
	The Centre sets nutrition research agenda	1995	Glimpse	Exclusion reason: Wrong study design
	Feeding frenzy	1998	Media Gend Monit	Exclusion reason: Wrong study design
	International Journal of Radiation Oncology, Biology, Physics		Exclusion reason: Wrong study design	
	ISRHML Abstracts	2010	Journal of Human Lactation	Exclusion reason: Wrong study design
	Journal of the International Association of Physicians in AIDS Care: JIAPAC. [ON EXAM]	Exclusion reason: Wrong study design		
	Nutrition research reviews	1999		Exclusion reason: Wrong study design
	Project boosts chances of infant survival in Vietnam	2011	Australian Nursing Journal	Exclusion reason: Wrong study design
	Randomised trial to assess benefits and safety of vitamin A supplementation linked to immunisation in early infancy	1998	Lancet	Exclusion reason: Wrong patient population
	Reducing mother‐to‐child HIV transmission	1999	AIDS Action	Exclusion reason: Wrong intervention
	SAJOG: the Official Journal of the South African Society of Obstetricians and Gynaecologists	Exclusion reason: Wrong study design		
	The China Study ‐‐ what it means…T. Colin Campbell	2004	Nutrition Health Review: The Consumer's Medical Journal	Exclusion reason: Wrong study design
	Unmet need for family planning	1998	Global Issues	Exclusion reason: Wrong study design
	Vitamin A supplementation and HIV	1996	South African Medical Journal	Exclusion reason: Wrong study design
	Zambia project serving as African model	1997	JOICFP News	Exclusion reason: Wrong study design
	Prise en charge de l'enfant atteint d'infection grave ou de malnutrition sévère: directives de soins pour les centres de transfert de premier niveau dans les pays en développement	2002		Exclusion reason: Wrong study design
	Abstracts from 3rd Symposium on Research in International Health "Research for Better Health", Amsterdam, Netherlands, May 19, 2004	2005	Tropical Medicine and International Health	Exclusion reason: Wrong study design
	Annual Report ‐ Nestle Foundation for the Study of the Problems of Nutrition of the World, 2007	2007		Exclusion reason: Wrong study design
	Management of the child with a serious infection or severe malnutrition: guidelines for care at the first‐referral level in developing countries	2000		Exclusion reason: Wrong study design
	Micronutrients, maternal and child health. Goa, India, 25‐27 April 1999. Symposium proceedings	2001	Br J Nutr	Exclusion reason: Wrong study design
	Midwifery and childbirth news	2007	Midwifery Today	Exclusion reason: Wrong study design
	New ingredients	2009	Functional Ingredients	Exclusion reason: Wrong study design
	Nutrition, health, and child development: research advances and policy recommendations	1998		Exclusion reason: Wrong study design
	Report of the first meeting of the micronutrient forum 16‐18 April 2007, Istanbul, Turkey	2007	Sight and Life Magazine	Exclusion reason: Wrong study design
	ASSA‐SAGES 2002 Congress. Sun City, South Africa, 14‐19 June 2002	2002	SAMJ South African Medical Journal	Exclusion reason: Wrong study design

**Table 16:** Characteristics table of excluded studies (Database Searches)

Authors	Title	Published Year	Exclusion Reason
Widasari et al.	Effects of multi micronutrient and IFA supplementation in preconception period against birth length and birth weight: A randomized, double blind controlled trial in banggai regency, Central Sulawesi	2019	Can't find text
Vahedi et al.	Is fish oil supplementation effective on maternal serum FBS, oral glucose tolerance test, hemoglobin and hematocrit in low risk pregnant women? A triple‐blind randomized controlled trial	2018	Wrong intervention
Ullah et al.	Newborn physical condition and breastfeeding behaviours: Secondary outcomes of a cluser randomized trial of prenatal lipid based nutrient supplements in Bangladesh	2019	Wrong comparison
Ullah et al.	Provision of Pre‐ and Postnatal Nutritional Supplements Generally Did Not Increase or Decrease Common Childhood Illnesses in Bangladesh: A Cluster‐Randomized Effectiveness Trial	2019	Wrong intervention
Tosic et al.	Multivitamins, iron and folic acid supplementation in pregnancy	2019	Abstract
Soldo et al.	Effect of n‐3 long‐chain polyunsaturated fatty acids supplementation in healthy mothers on DHA and EPA profiles in maternal and umbilical blood: a randomized controlled trial	2019	Wrong intervention
Rostami et al.	The supplementary effect of 50,000 units of vitamin D in maternal and infants	2018	Wrong intervention
Rahul et al.	Effect of iron and folic acid tablet versus capsule formulation on treatment compliance and iron status among pregnant women: a randomized controlled trial	2019	Wrong intervention
Quinn et al.	Mediators of the Effect of Multiple Micronutrient Supplementation in Pregnancy on Infant Mortality in Tanzania (P24‐050‐19)	2019	Abstract
Ping et al.	Effect of docosahexenoic acid supplementation on infant's growth and body mass index during maternal pregnancy. [Article in Chinese]	2018	No access to text
Ostadrahimi et al.	The effect of perinatal fish oil supplementation on neurodevelopment and growth of infants: a randomized controlled trial	2018	Wrong intervention
Omotayo et al.	Feasibility of integrating calcium and iron‐folate supplementation to prevent preeclampsia and anemia in pregnancy in primary healthcare facilities in Kenya	2018	Wrong study design
Olsen et al.	Examining the Effect of Fish Oil Supplementation in Chinese Pregnant Women on Gestation Duration and Risk of Preterm Delivery	2019	Wrong intervention
Matias et al.	Prenatal and Postnatal Supplementation with Lipid‐Based Nutrient Supplements Reduces Anemia and Iron Deficiency in 18‐Month‐Old Bangladeshi Children: A Cluster‐Randomized Effectiveness Trial	2018	Wrong comparison
Matias et al.	Daily Maternal Lipid‐Based Nutrient Supplementation with 20 mg Iron, Compared with Iron and Folic Acid with 60 mg Iron, Resulted in Lower Iron Status in Late Pregnancy but Not at 6 Months Postpartum in Either the Mothers or Their Infants in Bangladesh	2018	Wrong Comparison
Ma et al.	Analysis on iodine nutrition and thyroid levels of pregnant women with access to iodine supplement intervention in Xinjiang Uygur Autonomous Region	2018	Wrong Study Design
Liu et al.	A retrospective study of supplemental iron intake in singleton pregnancy women with risk of developing gestational diabetes mellitus	2018	Wrong study design
Khandelwal et al.	The impact of Docosa Hexaenoic Acid supplementation during pregnancy and lactation on Neurodevelopment of the offspring in India (DHANI): trial protocol	2018	Wrong intervention
Jorgensen et al.	Effects of lipid‐based nutrient supplements or multiple micronutrient supplements compared with iron and folic acid supplements during pregnancy on maternal haemoglobin and iron status	2018	Wrong comparison
Huda et al.	A community‐based cluster randomised controlled trial in rural Bangladesh to evaluate the impact of the use of iron‐folic acid supplements early in pregnancy on the risk of neonatal mortality: the Shonjibon trial	2018	Wrong study design
Goonewardene et al.	Randomized control trial comparing effectiveness of weekly versus daily antenatal oral iron supplementation in preventing anemia during pregnancy	2018	Wrong intervention
Garmendia et al.	Effectiveness on maternal and offspring metabolic control of a home‐based dietary counseling intervention and DHA supplementation in obese/overweight pregnant women (MIGHT study): a randomized controlled trial ‐ study protocol	2018	Wrong study design
Friebert et al.	Adolescent pregnancy and nutrition: a subgroup analysis from the Mamachiponde study in Malawi	2018	Wrong intervention
Enkhmaa et al.	Randomized trial of three doses of vitamin D to reduce deficiency in pregnant Mongolian women	2019	Wrong intervention
Dewey et al.	Effects of maternal supplementation with small‐quantity lipid‐based nutrient supplements on breast milk composition and infant outcomes	2018	Abstract
Das et al.	Lipid‐based nutrient supplements for maternal, birth, and infant developmental outcomes	2018	Wrong study design
Clermont et al.	Acceptability and Utilization of Three Nutritional Supplements during Pregnancy: Findings from a Longitudinal, Mixed‐Methods Study in Niger	2018	Wrong outcome
Cheng et al.	Effects of Maternal Prenatal Multi‐Micronutrient Supplementation on Growth and Development until 3 Years of Age	2019	Wrong study design
Chaiopanont et al.	Effectiveness of Modified Iodine Consumption Behavior Model in Pregnant Women by Civil Society Integrated Participation in Khon Kaen Province: a participatory action research	2019	No access to text
Baker et al.	Micronutrient Status of Young Adolescents in Rural Bangladesh: The JiVitA‐1 Birth Cohort (FS01‐04‐19)	2019	Abstract
Asadi et al.	Effects of Prophylactic Iron Supplementation on Outcome of Nonanemic Pregnant Women; A Non‐Randomized Clinical Trial	2019	Wrong study design
Anand et al.	Effects of maternal B12 supplementation on neurophysiological outcomes in children: a study protocol for an extended follow‐up from a placebo randomised control trial in Bangalore, India	2019	Wrong study design
Alizadeh et al.	Effect of iron supplementation in pregnant women with high hemoglobin on neonatal jaundice: a randomized double‐blind clinical trial	2019	No access to text
Ahmed et al.	Effect of routine iron‐folic acid supplementation among rural pregnant women living in low‐ and high‐groundwater‐iron areas in Bangladesh	2019	Wrong study design
Ahamed et al.	Effect of directly observed oral iron supplementation during pregnancy on iron status in a rural population in Haryana: A randomized controlled trial	2018	No access to text
Adaji et al.	Daily versus twice daily dose of ferrous sulphate supplementation in pregnant women: A randomized clinical trial	2019	Wrong intervention
Abbasnezhad et al.	Impact of iodine supplementation on serum TSH and free T4 levels in pregnant women residing in Mahabad City, 2015‐2016	2018	No access to text
Field Exchange	Independent and combined effects of improved WASH and improved complementary feeding on child stunting and anaemia in rural Zimbabwe	2019	Wrong intervention
ENN EN	Impact evaluation of a DFID programme to accelerate improved nutrition for the extreme poor in Bangladesh	2018	Wrong Study Design
Herrera et al.	Calcium and Conjugated Linoleic Acid Reduces Pregnancy‐Induced Hypertension and Decreases Intracellular Calcium in Lymphocytes	2006	Wrong intervention
Herrera et al.	Calcium oral supplementation in adolescent pregnant women [Spanish]	2006	Wrong intervention
Herrera et al.	Calcium plus linoleic acid therapy for pregnancy induced‐hypertension	2005	Wrong intervention

### 5.13 Risk of bias in included studies

**Figure 1:**
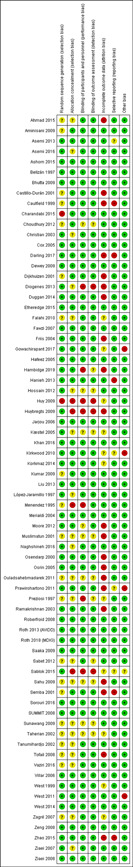
Study flow diagram

**Figure 2:**
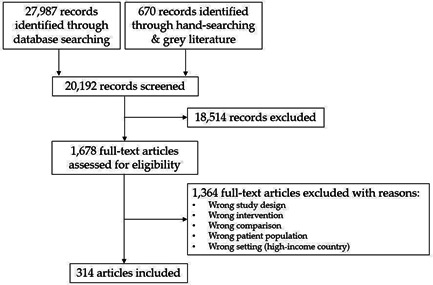
Risk of bias graph: review authors' judgements about each risk of bias item presented as percentages across all included studies

The risk of bias of the included studies was overall low with at least 50% of the judgements at low risk for three domains (random sequence generation, allocation concealment, and incomplete outcome data. For the remaining four domains, at least 75% of the judgements were assessed as low risk. The domain with the highest risk of bias was incomplete outcome data (attrition bias). See Figures 1 and 2, and the Characteristics of included studies tables for further details on the risk of bias for each included study.

**Figure 3:**
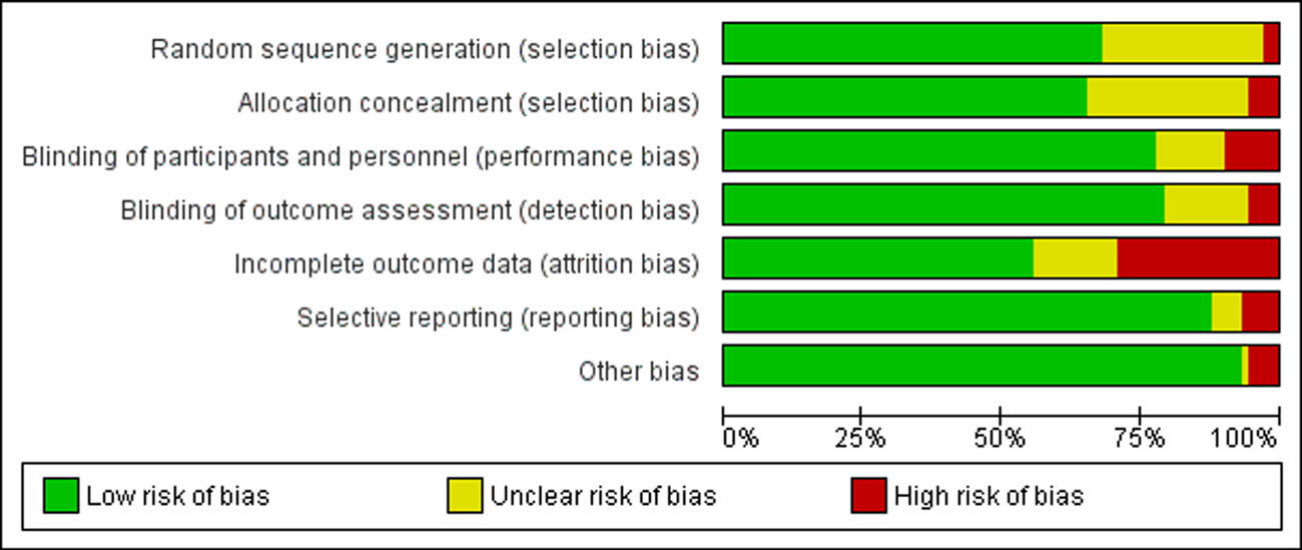
Risk of bias summary: review authors’ judgements about each risk of bias item for each included study

### 5.14 Allocation (selection bias)

#### 5.14.1 Randomisation Sequence Generation

The included studies were of variable risk of bias in terms of randomisation of participants. Of the 73 included studies, 21 did not mention or describe the method used to generate a randomisation sequence in sufficient detail to permit judgement (Ahmad et al., 2016; Aminisani et al., 2009; Castillo‐Durán et al., 2001; Caulfield et al.,^1999^; Choudhury et al., 2012; Dijkhuizen et al., 2001; Falahi 2010; Kumar et al., 2009; Menendez et al., 1995; Muslimatun et al., 2001; Ouladsahebmadarek et al., 2011; Preziosi et al., 1997; Sabet et al., 2012; Sahu et al., 2009; Semba et al., 2001; Sunawang et al., 2009; Taherian et al., 2002; Tanumihardjo et al., 2002; Tofail et al., 2008; Vaziri et al., 2016; Zagré et al., 2007). Two studies were assessed as high risk related to randomisation (Huy et al., 2009; Mohammad‐Alizadeh‐Charandabi et al., 2015). The remaining studies adequately reported randomisation procedures for assigning participants to the intervention and control groups.

#### 5.14.2 Allocation Concealement

The included studies were of variable risk of bias in terms of allocation concealment. Of the included studies, 19 studies did not mention or describe the method used to allocate concealment in sufficient detail to permit judgement Ahmad et al., 2016; Asemi et al., 2016; Christian et al., 2003; Falahi 2010; Hossain et al., 2012; Kæstel et al., 2005; López‐Jaramillo et al., 1997; Muslimatun et al., 2001; Naghshineh & Sheikhaliyan, 2016; Ouladsahebmadarek et al., 2011; Preziosi et al., 1997; Sabet et al., 2012; Sahu et al., 2009; Sunawang et al., 2009; Taherian et al., 2002; Tanumihardjo et al., 2002; Tofail et al., 2008; Vaziri et al., 2016; Zagré et al., 2007). Four studies were assessed as high risk for allocation concealment (Huy et al., 2009; Huybregts et al., 2009; Menendez et al., 1995; Sablok et al., 2015). The remaining studies had adequate allocation concealment of participants to the intervention and control groups.

##### Blinding (performance bias and detection bias)

For several included studies, it was unclear whether participants and personnel were appropriately blinded to the treatment allocation (Choudhury et al., 2012; Hossain et al., 2012; Kæstel et al., 2005; Moore et al., 2012; Muslimatun et al., 2001; Ouladsahebmadarek et al., 2011; Sahu et al., 2009; Sunawang et al., 2009; Taherian et al., 2002). Seven studies were assessed as high risk in terms of blinding (Diogenes et al., 2013; Hambidge et al., 2019; Huy et al., 2009; Huybregts et al., 2009; Menendez et al., 1995; Preziosi et al., 1997; Sablok et al., 2015).

In terms of blinding of outcome assessors, eleven studies did not clearly describe the method used to blind outcome assessors to permit judgement (Choudhury et al., 2012; Hambidge et al., 2019; Hossain et al., 2012; Kæstel et al., 2005; Muslimatun et al., 2001; Ouladsahebmadarek et al., 2011; Preziosi et al., 1997; Sabet et al., 2012; Sahu et al., 2009; Sunawang et al., 2009; et al., Taherian 2002). Four studies did not adequately blind outcome assessors, and were thus assessed as high risk (Diogenes et al., 2013; Huy et al., 2009; Huybregts et al., 2009; Sablok et al., 2015).

##### Incomplete outcome data (attrition bias)

Loss to follow up was >20% in 21 studies (Ahmad et al., 2016; Castillo‐Durán et al., 2001; Caulfield et al., 1999; Darling et al., 2017; Dijkhuizen et al., 2001; Diogenes et al., 2013; Duggan et al., 2014; Friis et al., 2004; Hambidge et al., 2019; Huy et al., 2009; Moore et al., 2012; Muslimatun et al., 2001; Osendarp et al., 2000; Osrin et al., 2005; Ouladsahebmadarek et al., 2011; Prawirohartono et al., 2011; Ramakrishnan et al., 2003; Sahu et al., 2009; Semba et al., 2001; Tofail et al., 2008; Zhao et al., 2015). Thus, these studies were assessed as high‐risk for incomplete outcome data. Zagré et al. (2007) reported an attrition less than 20%; however, the proportion of women who dropped out was significantly higher in the MMN group versus the IFA group. Thus, this study was assessed as unclear risk. Other studies that were deemed unclear risk were: Falahi 2010; Gowachirapant et al., 2017; Huybregts et al., 2009; Kæstel et al., 2005; Kirkwood et al., 2010; Korkmaz et al., 2014; Preziosi et al., 1997; Sablok et al., 2015; Taherian et al., 2002; West et al., 1999).

##### Selective reporting (reporting bias)

In the majority of the included studies, there was no indication of selective reporting. However, in four studies, incidence of selective reporting was unclear (Asemi et al., 2013; Kirkwood et al., 2010; Prawirohartono et al., 2011; Sablok et al., 2015). In five additional studies, the risk of selective reporting was assessed as high, as authors did not report all of the outcomes described in the methods (Caulfield et al., 1999; Hambidge et al., 2019; Hanieh et al., 2013; Semba et al., 2001; Zhao et al., 2015).

##### Other potential sources of bias

In the majority of the included studies we did not identify other possible sources of bias. Sablok 2015 was assessed as unclear risk given that the intervention and control groups were significantly different in size. Four studies were assessed as high risk for other sources of bias (Gowachirapant et al., 2017; Kirkwood et al., 2010; Prawirohartono et al., 2011; West et al., 2011). In Gowachirapant et al. (2017), authors indicated that their iodine concentration measurements were not accurately conducted and that participants may have been mis‐classified. They also stated that iodine nutrition may have influenced participants' behaviours to consume more iodine rich foods. In Kirkwood et al. (2010), the authors revealed that women were switched into different treatment arms if they migrated to a different cluster area. Prawirohartono et al. (2011) began in 1994; however, results of the original study were not published with reasons not provided. The results presented here pertain to follow‐up studies conducted in 2011 and 2013. Lastly, authors of West et al. (2011) indicated that the vitamin A supplementation outcomes may have been biased by women receiving vitamin A to treat for night blindness.

##### Effects of interventions

 

### 5.15 Comparison I: IFA versus Folic Acid

Seven studies contributed to this comparison (Christian et al., 2003; Etheredge et al., 2015; Liu et al., 2013; Menendez et al., 1995; Zeng et al., 2008; Zhao et al., 2015; Ziaei et al., 2007). Of these, 2 studies were conducted in sub‐Saharan Africa (Etheredge et al., 2015; Menendez et al., 1995), 3 in East Asia Pacific, specifically in China (Liu et al., 2013; Zeng et al., 2008; et al., Zhao 2015), 1 in Nepal, South Asia (Christian et al., 2003) and 1 in Iran, Middle East & North Africa (Ziaei et al., 2007). Three studies utilized a similar formulation of IFA supplement; the equivalent of 60 mg elemental iron and 400 ug folic acid (Christian et al., 2003; Zeng et al., 2008; Zhao et al., 2015). Liu et al. 2013 used 30 mg of iron and 400 ug of folic acid, while Etheredge et al. (2015) and Menendez et al. (1995) utilized 60 mg of iron and 5 mg of folic acid, and Ziaei 2007 used 50 mg of iron and 1 mg of folic acid. In one study, women received 1000 ug vitamin A daily with their supplements (Christian et al., 2003), and in another study, women received iron and folic acid in separate capsules (Zhao et al., 2015). For all studies, participants began supplementation at enrolment and continued until delivery. Liu et al. (2013) began supplementation at the women's first prenatal visit until delivery. Etheredge 2015 stated that enrolment was at or before 27 weeks of gestation; while Zhao et al. (2015) stated enrolment began at the first prenatal visit, and Ziaei et al. (2007) stated that women were in their early stage of the second trimester at enrolment. Enrolment was undefined and unclear in 3 studies Christian et al., 2003; Menendez et al., 1995; Zeng et al., 2008.

#### 5.15.1 Primary Outcomes

When comparing IFA to folic acid supplementation, maternal anaemia was reduced by 48% (average risk ratio (RR) 0.52, 95% confidence interval (CI) 0.41 to 0.66; studies = 5; participants = 15,540; random effects; Tau² = 0.07; I² = 88%; moderate quality evidence; Analysis 1.1). IFA supplementation also reduced low birthweight (average RR 0.88, 95% CI 0.78 to 0.99; studies = 4; participants = 17,257; random‐effects; Tau² = 0.00%; I² =0%; high quality evidence; Analysis 1.2); however, IFA probably did not reduce perinatal mortality (average RR 0.88, 95% CI 0.71 to 1.08; studies = 4; participants = 17,464; random‐effects; Tau² = 0.00; I² = 0%; moderate quality evidence; Analysis 1.3) when compared to supplementation with folic acid alone.

#### 5.15.2 Secondary Outcomes

When compared to folic acid alone, IFA supplementation increased maternal hemoglobin concentration (average mean difference (MD) 6.95 g/L; 95% CI 2.80 to 11.1; studies = 7; participants = 16,089; random‐effects; Tau² = 29.46; I² = 98%; Analysis 1.4). Likewise, IFA supplementation increased maternal serum/plasma ferritin (average MD 15.87 ug/L; 95% CI 2.96 to 28.79; studies = 5; participants = 3,894; random‐effects; Tau² = 194.91; I² = 100%; Analysis 1.5), though there was no difference in maternal serum/plasma transferrin receptor (average MD ‐0.16; 95% CI ‐0.96 to 0.65; studies = 3; participants = 2,431; random‐effects; Tau² = 0.46; I² = 99%; Analysis 1.6). Additionally, there was no difference in neonatal mortality (average RR 0.85; 95% CI 0.55 to 1.31; studies = 3; participants = 15,794; Tau² = 0.07; I² = 47%; Analysis 1.7), preterm births (average RR 0.96 95% CI 0.64 to 1.44; studies = 5; participants = 17,637; random‐effects; Tau² = 0.18; I² = 96%; Analysis 1.8), SGA (average RR 1.03; 95% CI 0.87 to 1.23; studies = 4; participants = 6,549; random‐effects; Tau² = 0.02; I² = 69%; Analysis 1.9), or infant mortality (average RR 1.10; 95% CI 0.84 to 1.45; studies = 3; participants = 14,748; random‐effects; Tau² = 0.00; I² = 0%; Analysis 1.10) when comparing IFA and folic acid supplementation.

#### 5.15.3 Subgroup Analysis

Due to insufficient data (this review required > or equal to 3 studies per subgroup of interest), subgroup analyses of the primary outcomes were not conducted based on maternal age, geographical region, sex of infant, baseline nutritional status in mothers (anemic vs. non‐anemic; undernutrition vs. normal nutrition based on body mass index: BMI <18.5; low stature vs. normal stature), duration of intervention, time of recruitment (preconception vs. first trimester vs. second trimester vs. third trimester of pregnancy), daily vs. intermittent IFA supplementation and dose of intervention.

### 5.16 Comparison II: MMN versus IFA

Thirty‐four studies contributed data to this comparison (Aminisani et al., 2009; Asemi et al., 2016; Ashorn et al., 2015; Bhutta et al., 2009; Caulfield et al., 1999; Choudhury et al., 2012; Christian et al., 2003; Dewey et al., 2009; Dijkhuizen et al., 2001; Fawzi et al., 2007; Friis et al., 2004; Hafeez et al., 2005a, 2005b; Hanieh et al., 2013; Huy et al., 2009; Kæstel et al., 2005; Liu et al., 2013; Merialdi et al., 2004; Moore et al., 2012; Muslimatun et al., 2001; Osrin et al., 2005; Ramakrishnan et al., 2003; Roberfroid et al., 2008; Roth et al., 2013 (AViDD); Roth et al., 2018 (MDIG); Saaka et al., 2009; Semba et al., 2001; Sorouri et al., 2016; SUMMIT Study Group, 2008; Sunawang et al., 2009; Tofail et al., 2008; Villar et al., 2006; West et al., 2014; Zagré et al., 2007; Zeng et al., 2008). Of these, 10 studies were conducted in sub‐Saharan Africa; 2 conducted in Ghana (Dewey et al., 2009; Saaka et al., 2009); 2 in Malawi (Ashorn et al. (2015); Semba et al. (2001); 1 each in Burkina Faso (Roberfroid et al., 2008); Gambia (Moore et al., 2012); Guinea‐Bissau (Kæstel et al., 2005); Tanzania (Fawzi et al., 2007); Zimbabwe (Friis et al., (2004); and Niger (Zagré et al., 2007). Eight studies were conducted in the East Asia & Pacific region; 4 were conducted in Indonesia (Dijkhuizen et al., 2001; Muslimatun et al., 2001; SUMMIT Study Group, 2008; Sunawang et al., 2009), 2 in Vietnam Hanieh et al., 2013; Huy et al., 2009) and 2 in China (Liu et al., 2013; Zeng et al., 2008). Nine studies were conducted in the South Asia region; of which 5 were conducted in Bangaldesh (Choudhury et al., 2012; Roth et al., 2013 (AViDD); Roth et al., 2018 (MDIG); Tofail et al., 2008; West et al., 2014), 2 in Nepal (Christian et al., 2003; Osrin et al., 2005), 2 in Pakistan (Bhutta et al., 2009; Hafeez et al., 2005a, b). Three studies were conducted in Latin America & the Caribbean: 2 in Peru (Caulfield et al., 1999; Merialdi et al., 2004) and 1 in Mexico (Ramakrishnan et al., 2003). Three studies were conducted in the Middle East & North Africa; all in Iran (Aminisani et al., 2009; Asemi et al., 2016; Sorouri et al., 2016). One study, Villar et al. (2006), was a multi‐country study, conducted in India, Peru, South Africa and Vietnam.

Eleven studies used the UNIMMAP formulation, developed by UNICEF, WHO and United Nations University (Bhutta et al., 2009; Huy et al., 2009; Kæstel et al., 2005; Liu et al., 2013; Osrin et al., 2005; Roberfroid et al., 2008; SUMMIT Study Group, 2008; Sunawang et al., 2009; Tofail et al., 2008; Zagré et al., 2007; Zeng et al., 2008). The UNIMMAP formulation consists of 800 RE of vitamin A, 200 IU vitamin D, 10 mg of vitamin E, 70 mg of vitamin C, 1.4 mg of thiamin, 1.4 mg of riboflavin, 18 mg of niacin, 1.9 mg of pyridoxine, 2.6 mg of cobalamin, 400 ug of folic acid, 30 mg of iron, 15 mg of zinc, 2 mg of copper, 65 mg of selenium and 150 ug of iodine. Three studies used an adapted UNIMMAP formulation which contained the exact same combination of vitamins and minerals, but in different dosages (Hanieh et al., 2013; Moore et al., 2012; West et al., 2014). Hanieh et al. (2013) used 60 mg of iron and 1500 ug of folic acid; Moore et al. (2012) used a formulation that doubled the volume of every component of the UNIMMAP formulation, along with 60 mg of iron and 400 ug of folic acid. West 2014 changed the volumes of vitamin A (770 retinol equivalents), vitamin E (15 mg), vitamin C (85 mg), folic acid (600 ug), iron (27 mg), zinc (12 mg), copper (1 mg), selenium (60 ug and iodine (220 ug).

The remaining 20 studies used non‐UNIMMAP formulations for their MMN supplements (Aminisani et al., 2009; Asemi et al., 2016; Ashorn et al., 2015; Caulfield et al., 1999; Choudhury et al., 2012; Christian et al., 2003; Dewey et al., 2009; Dijkhuizen et al., 2001; Fawzi et al., 2007; Friis et al., 2004; Hafeez et al., 2005; Merialdi et al., 2004; Muslimatun et al., 2001; Ramakrishnan et al., 2003; Roth et al., 2013 (AViDD); Roth et al., 2018 (MDIG); Saaka et al., 2009; Semba et al., 2001; Sorouri et al., 2016; and Villar et al., 2006). Of this list, Aminisani et al., 2009; Caulfield et al., 1999; Dijkhuizen et al., 2001; Villar et al., 2006; Hafeez et al., 2005; Merialdi et al., 2004; Muslimatun et al., 2001; Roth et al., 2013 (AViDD); Saaka et al., 2009; Semba et al., 2001; Sorouri et al., 2016 used MMN formulations that included iron, folic acid and 1 other micronutrient, typically zinc or vitamin A. Choudhury et al. (2012) and Roth et al. (2018) (MDIG) utilized MMN supplements that contained 4 components; Choudhury et al. (2012) used iron 60 mg, 400 ug of folic acid, 30 mg of vitamin C and 5 mg of zinc, and Roth et al. (2018) (MDIG) used iron 66 mg, folic acid 350 ug, calcium 500 mg and vitamin D (dosages differed across different arms). The remaining studies (Ashorn et al., 2015; Dewey et al., 2009; Fawzi et al., 2007; Friis et al., 2004; and Ramakrishnan et al., 2003) used formulations that had many micronutrient and vitamin components that differed from UNIMMAP. For example, some used the UNIMMAP formulation with the addition of pantothenic acid, vitamin K, and manganese (Ashorn et al., 2015; Dewey et al., 2009). The other studies removed some of the UNIMMAP components and added others into their study formulations (Fawzi et al., 2007; Friis et al., 2004; and Ramakrishnan et al., 2003).

In addition to any pre‐specified, per‐protocol subgroup analyses, an exploratory post‐hoc analysis based on the number of micronutrients and vitamins comprised in each supplement (supplements containing 3 or 4 micronutrients versus supplements containing >4 micronutrients) was conducted for all outcomes. Post‐hoc analyses were conducted for the following outcomes: maternal anaemia, maternal iron‐deficiency anaemia, low birthweight, perinatal mortality, maternal serum/plasma hemoglobin concentration, maternal serum/plasma ferritin concentration, maternal iron deficiency, maternal serum/plasma transferrin receptor concentration, maternal serum/plasma retinol concentration, maternal serum/plasma zinc concentration, maternal serum/plasma folate concentration, maternal serum/plasma vitamin B12 concentration, stillbirths, congenital anomalies, preterm births, SGA, mode of delivery ‐ Caesarean section, neonatal mortality, development outcomes: general intelligence, verbal comprehension and language, and motor function, child diarrhea, child serum/plasma hemoglobin concentration, child serum/plasma ferritin concentration, child serum/plasma retinol concentration, and child serum/plasma zinc concentration.

#### 5.16.1 Primary Outcomes

Compared to iron with or without folic acid supplementation, MMN supplementation made no difference to maternal mortality (average RR 1.04, 95% CI 0.71 to 1.51; studies = 6; participants = 75,051; random‐effects; Tau² = 0.00; I² = 0%; moderate quality evidence; Analysis 2.1) and had no effect on maternal anaemia (average RR 1.02; 95% CI 0.95 to 1.10; studies = 16; participants = 23,356; random‐effects; Tau² = 0.01; I² = 57%; high quality evidence; Analysis 2.2). A post‐hoc analysis comparing studies with MMN containing >4 micronutrients to those with MMN containing 3 or 4 micronutrients showed no difference in the effects on maternal anaemia.

Maternal iron deficiency anaemia was not improved, though the certainty of evidence was very low (average RR 1.12; 95% CI 0.62 to 2.02; studies = 4; participants = 1,595; random‐effects; Tau² = 0.24; I² = 73%; very low quality evidence; Analysis 2.3). Post‐hoc analysis comparing studies based on supplement composition showed no difference in effect.

MMN supplementation had a large effect on low birthweight, reducing the risk by 15% (average RR 0.85; 95% CI 0.77 to 0.93; studies = 28; participants = 79,972; random‐effects; Tau² = 0.03; I² = 70%; high quality evidence; Analysis 2.4). However, post‐hoc analysis comparing the subgroups of studies whose MMN formulation contained >4 micronutrients to those whose formulation contained <or equal to 4 micronutrients, revealed that the former subgroup demonstrated a greater reduction in risk of low birthweight (average RR 0.79, 95% CI 0.71 to 0.88; studies = 19; participants = 68,138; random‐effects; Tau² = 0.03; I² = 74%; Analysis 2.3.1 versus average RR 1.01, 95% CI 0.92 to 1.11; studies = 9; participants = 11,834; random‐effects; Tau^2^ =0.00; I^2^ = 1%; Analysis 2.3.2). There was a significant difference between subgroups (test for subgroup differences: Chi² = 11.51; P = 0.0007).

Compared to IFA, MMN did not demonstrate any important effect on perinatal mortality (average RR 1.00; 95% CI 0.90 to 1.11; studies = 16; participants = 92,769; random‐effects; Tau² = 0.01; I² = 43%; high quality evidence; Analysis 2.5). Post hoc‐analysis showed no difference in perinatal mortality between studies with MMN containing >4 micronutrients (average RR 0.97; 95% CI 0.88 to 1.07; studies = 14; participants = 90,959; random effects; Tau² = 0.01; I² = 33%; Analysis 2.5.1) compared to MMN with <or equal to 4 micronutrients (average RR 1.79; 95% CI 0.56 to 5.76; studies = 2; participants = 1,810; random effects; Tau² = 0.38; I² = 25%; Analysis 2.5.2). The test for sub‐group differences was non‐significant (test for subgroup differences: Chi² = 1.06, P = 0.30).

#### 5.16.2 Secondary Outcomes

Compared to iron with or without folic acid supplementation, MMN supplementation made no difference to maternal hemoglobin (average MD ‐0.34 g/L, 95% CI ‐1.53 to 0.86; studies = 16; participants = 26,312; random‐effects; Tau² = 4.61; I² = 92%; Analysis 2.6) and ferritin concentrations (average MD ‐2.37 ug/L, 95% CI ‐7.84 to 3.10; studies = 11; participants = 5,149; random‐effects; Tau² = 79.82; I² = 100%; Analysis 2.7). Similarly, MMN supplementation had no effect on maternal iron deficiency (average RR 1.39; 95% CI 0.88 to 2.20; studies = 3; participants = 1,182; random‐effects; Tau² = 0.14; I² = 86%; Analysis 2.8). Post‐hoc analyses for maternal hemoglobin and ferritin concentrations, and maternal iron deficiency all showed no differences in the effects between studies that provided supplements with >4 micronutrients and studies whose formulations contained 3 or 4 micronutrients. MMN supplementation may have slightly improved maternal serum transferrin receptor concentration, although the confidence interval of the pooled estimate just crossed the line of no effect (average MD 0.12 mg/L; 95% CI ‐0.03 to 0.27; studies = 7, participants = 2,550; random‐effects; Tau² = 0.02; I² = 79%; Analysis 2.9).

Similarly, MMN supplementation slightly improved maternal serum retinol concentration (average MD 0.11 umol/L; 95% CI 0.05 to 0.17; studies = 7; participants = 3,111; random‐effects; Tau² = 0.00; I² = 60%; Analysis 2.10), maternal serum zinc concentration (average MD 0.40 umol/L; 95% CI 0.18 to 0.62; studies = 5; participants = 3028; random‐effects; Tau² = 0.07; I² = 49%, Analysis 2.11), and maternal serum vitamin B12 concentration (average MD 14.77 pmol/L; 95% CI 5.13 to 24.42; studies = 3; participants = 962; random‐effects; Tau² = 0.00; I² = 0%; Analysis 2.12), but had no effect on maternal serum folate concentration (average MD ‐1.66 nmol/L; 95% CI ‐4.75 to 1.08; studies = 5; participants = 2,614; random‐effects; Tau² = 10.28; I² = 79%; Analysis 2.13).

MMN supplementation probably did not improve the risk of miscarriage (average RR 0.99; 95% CI 0.94 to 1.04; studies = 13; participants = 88,971; random‐effects; Tau² = 0.00; I² = 0%; Analysis 2.14). However, MMN supplementation did reduce the risk of stillbirths (average RR 0.91; 95% CI 0.86 to 0.98; studies = 22; participants = 96,772; random‐effects; Tau² = 0.00; I² = 0%; Analysis 2.15) when compared to IFA. In a post‐hoc analysis, no significant differences in stillbirths were observed between MMN formulation groups (test for subgroup differences: Chi² = 0.05; P = 0.82).

MMN supplementation, compared to IFA, did not reduce congenital anomalies (average RR 0.73; 95% CI 0.41 to 1.29; studies =7; participants = 4,195; random‐effects; I² = 0%; Analysis 2.16), but may have slightly reduced births that were considered preterm, although the line of no effect just crosses one (average RR 0.96, 95% CI 0.91 to 1.01; studies = 29; participants = 99,855; random‐effects; Tau² = 0.00; I² = 37%; Analysis 2.17). In post‐hoc analysis, no significant differences were observed between studies that provided MMN comprised of of 3 or 4 micronutrients versus MMN comprised of >4 micronutrients (test for subgroup differences: Chi² = 0.69; P = 0.41). MMN supplementation showed a small 7% improvement in the risk of babies considered SGA overall (average RR 0.93; 95% CI 0.88 to 0.98; studies = 19; participants = 52,965; random‐effects; Tau² = 0.00; I² = 38%; Analysis 2.18). However, when comparing MMN supplement formulations, there were significant differences in SGA between groups (test for subgroup differences: Chi² = 10.49; P = 0.001). Women who took MMN supplements comprised of >4 micronutrients showed greater reduction in risk of SGA when compared to women who took MMN with <or equal to 4 micronutrients (average RR 0.90; 95% CI 0.85 to 0.96; studies = 16; participants = 50,788; random‐effects, Tau² = 0.00; I² = 33%; Analysis 2.19.1 compared to average RR 1.07; 95% CI 0.98 to 1.16; studies = 3; participants = 2,177; random‐effects; Tau² = 0.00; I² = 0%; Analysis 2.19).

MMN supplementation during pregnancy overall had no effect on reducing the risk of Caesarean section as a mode of delivery (average RR 1.00; 95% CI 0.94 to 1.07; studies = 11; random‐effects; Tau² = 0.00; I² = 0%; Analysis 2.19). Post‐hoc analysis showed no significant difference between studies which provided MMN with 3 or 4 micronutrients (average RR 0.97; 95% CI 0.90 to 1.04; studies = 6; participants = 10,527; random‐effects; Tau² = 0.00; I² = 0%; Analysis 2.19.2) when compared to and studies that provided MMN with >4 micronutrients (average RR 1.13; 95% CI 0.99 to 1.29; studies = 5; participants = 13,217; random‐effects; Tau² = 0.00; I² = 0%; Analysis 2.19.1) (test for subgroup differences: Chi² = 3.86, P = 0.05).

For outcomes pertaining to the child, MMN supplementation probably did not reduce the risk of neonatal mortality overall (average RR 0.98; 95% CI 0.90 to 1.06; studies = 17; participants = 82,293; random‐effects; I² = 14%; Analysis 2.20). No significant differences were observed between studies that used MMN supplements comprised of >4 micronutrients and studies using MMN supplements containing <or equal to 4 micronutrients (test for subgroup differences: Chi² = 1.65, P = 0.20).

Compared to IFA, MMN supplementation during pregnancy probably had no effect on reducing infant mortality (average RR 0.99; 95% CI 0.92 to 1.08; studies = 10; participants = 55,595; random‐effects; Tau² = 0.00; I² = 0%; Analysis 2.21). In terms of nutritional indicators, MMN supplementation had no effect on the risk of child wasting (average RR 1.02; 95% CI 0.88 to 1.18; studies = 5; participants = 9,671; random‐effects; Tau² = 0.00; I² = 7%; Analysis 2.22), stunting (average RR 0.99; 95% CI 0.92 to 1.07; studies = 7; participants = 11,264; random‐effects; Tau² = 0.00; I² = 30%; Analysis 2.23), or underweight (average RR 0.95; 95% CI 0.84 to 1.07; studies = 4; participants = 931; random‐effects; Tau² = 0.01; I² = 45%; Analysis 2.24).

There were four types of child developmental outcomes that were assessed in this comparison group: general intelligence, verbal comprehension and language, motor function, and executive function scores. Compared to IFA, MMN supplementation showed no effect on general intelligence (standard MD 0.00; 95% CI ‐0.06 to 0.07; studies = 8; participants = 12,172; Tau² = 0.00; I² = 36%; Analysis 2.25), and showed no effect on motor function (standard MD ‐0.02; 95% CI ‐0.17 to 0.13; studies = 7; participants = 12,057; random‐effects, Tau² = 0.03; I² = 89%; Analysis 2.26). There was also no difference in effect of MMN supplementation compared to IFA on verbal comprehension and language (standard MD 0.02; 95% CI ‐0.13 to 0.16; studies = 4; participants = 10,781; random‐effects, Tau² = 0.02; I² = 84%; Analysis 2.27). However, MMN supplementation compared to IFA did have a positive effect on executive function scores (standard MD 0.09; 95% CI 0.01 to 0.17; studies = 3; participants = 2,511; random‐effects, Tau² = 0.00; I² = 0%; Analysis 2.28).

Compared to IFA, MMN supplementation during pregnancy reduced the occurrence of diarrhea in children (average RR 0.84; 95% CI 0.76 to 0.92; studies = 4; participants = 3142; random‐effects; I² = 0%; Analysis 2.29). Maternal MMN supplementation had no impact on child hemoglobin concentration (average MD 0.01 g/L; 95% CI ‐0.63 to 0.66; studies = 7; participants = 13,067; random‐effects; Tau² = 0.25; I² = 61%; Analysis 2.30) or on ferritin levels (average MD 1.85 ug/L; 95% CI ‐0.81 to 4.50; studies = 4; participants = 1,443; random‐effects; Tau² = 0.00; I² = 0%; Analysis 2.32). MMN supplementation, when compared to IFA, demonstrated slight improvements in child serum retinol concentrations (average MD 0.06 umol/L; 95% CI 0.02 to 0.09; studies = 3; participants = 868; random‐effects; Tau² = 0.00; I² = 69%; Analysis 2.31). MMN supplementation did not have any major impact on zinc concentrations in children (average MD 0.04 umol/L; 95% CI ‐0.21 to 0.30; studies = 3; participants = 944; random‐effects; Tau² = 0.00; I² = 0%; Analysis 2.33), nor on anaemia among children (average RR 0.83; 95% CI 0.54 to 1.27; studies = 3; participants = 1,458; random‐effects; Tau² = 0.13; I² = 92%; Analysis 2.34).

#### 5.16.3 Subgroup Analysis

Subgroup analyses were conducted for the MMN vs. IFA comparison for maternal mortality, maternal anaemia, perinatal mortality and low birthweight based on MMN formulation (UNIMMAP vs. adapted UNIMMAP vs. non‐UNIMMAP formulations), geographical region, duration of intervention and dose of iron. Please see below for results under heading 'Subgroup Analysis I: MMN supplementation versus IFA'.

### 5.17 Comparison III: LNS versus MMN

Four randomized controlled trials that assessed LNS supplementation against MMN reported eligible outcomes (Ashorn et al., 2015; Dewey et al., 2009; Huybregts et al., 2009; Moore et al., 2012). Each study was conducted in Africa and in a rural setting, except Dewey et al. (2009) which was conducted in a peri‐urban setting. In 2 studies, women were provided supplements throughout pregnancy only (Huybregts et al., 2009; Moore et al., 2012), while in Ashorn et al. (2015) and Dewey et al. (2009), supplementation was continued up to 6 months postpartum. LNS supplementation was daily, and came packaged within a 20 g foil sachet in 2 studies (Ashorn et al., 2015; Dewey et al., 2009). Moore et al. (2012) and Huybregts et al. (2009) provided participants with daily LNS in the form of a fatty spread. Daily MMN supplementation was provided in the form of tablets to the control groups across all studies, but with variable composition. Three studies utilized a non‐UNIMMAP formulation (Dewey et al., 2009; Ashorn et al., 2015; Moore et al., 2012), while the fourth used UNIMMAP tablets. In Dewey et al. (2009) and Ashorn et al., 2015, the iron concentration in the MMN tablet was 20 mg while in Moore et al., 2012, it was 60 mg. Folic acid in the MMN tablets was 400 ug across all studies. Three studies provided participants with intermittent preventive treatment of malaria (Ashorn et al., 2015; Dewey et al., 2009; Huybregts et al., 2009).

#### 5.17.1 Primary outcomes

Compared to MMN, LNS supplementation had no effect on the risk of LBW (average RR 0.92; 95% CI 0.75 to 1.13; studies = 4; participants = 2,727; random‐effects*;* Tau² = 0.0, I²= 0.0%; moderate quality evidence; Analysis 3.1) and made no difference to perinatal mortality (average RR 1.01; 95% CI 0.65 to 1.65; studies = 3; participants = 2,771; random‐effects; Tau² = 0.04, I²= 21%; low quality evidence; Analysis 3.2).

#### 5.17.2 Secondary Outcomes

There was no difference in the risk of miscarriage between participants supplemented with LNS compared to those supplemented with MMN (average RR 1.12, 95% CI 0.69 to 1.80; studies = 3; participants = 2,865; random‐effects; Tau² = 0.0, I²= 0.0%; Analysis 3.3). Compared to MMN, LNS supplementation made no difference in the risk of stillbirths (average RR 0.47; 95% CI 0.12 to 1.81; studies = 3; participants = 2,481; random‐effects; Tau² = 0.0, I²= 0.0%; Analysis 3.4). Similarly, there were no effects of LNS on neonatal mortality (average RR 0.81; 95% CI 0.45 to 1.45; studies = 3; participants = 2,727; random‐effects; Tau² = 0.0, I²= 0.0%; Analysis 3.5) or on preterm birth (average RR 1.15; 95% CI 0.93 to 1.42; studies = 4; participants = 2,953; random‐effects, Tau² = 0.0, I²= 0.0%; Analysis 3.6). LNS supplementation did not reduce the risk of SGA infants (average RR 0.96; 95% CI 0.86 to 1.07; studies = 4; participants = 2,716; random‐effects, Tau² = 0.0, I²= 0.0%; Analysis 3.7).

#### 5.17.3 Subgroup Analysis

Due to insufficient data (this review required > or equal to three studies per subgroup of interest), subgroup analyses of the primary outcomes were not conducted based on maternal age, geographical region, sex of infant, baseline nutritional status in mothers (anemic vs. non‐anemic; undernutrition vs. normal nutrition based on body mass index: BMI <18.5; low stature vs. normal stature), duration of intervention, time of recruitment (preconception vs. first trimester vs. second trimester vs. third trimester of pregnancy), daily vs. intermittent IFA supplementation and dose of intervention.

### 5.18 Comparison IV: Vitamin A versus Placebo

Nine studies evaluated vitamin A supplementation versus placebo (Cox et al., 2005; Darling et al., 2017; Kirkwood et al., 2010; Muslimatun et al., 2001; Semba et al., 2001; Tanumihardjo et al., 2002; Prawirohartono et al., 2011; West et al., 1999; West et al., 2011). Of these, 4 studies were conducted in sub‐Saharan Africa, with 2 in Ghana and 1 each in Malawi and Tanzania (Cox 2005 et al.,; Darling et al., 2017; Kirkwood et al., 2010; Semba et al., 2001). Three studies were conducted in East Asia & Pacific, specifically in Indonesia (Muslimatun et al., 2001; Tanumihardjo et al., 2002; Prawirohartono et al., 2011), and 2 studies were conducted in Nepal and Bangladesh, South Asia (West et al., 1999; West et al., 2011). Four studies gave participants supplementation from enrolment to the end of pregnancy or delivery (Darling et al., 2017; Muslimatun et al., 2001; Semba et al., 2001; West et al., 1999). Cox et al. (2005) provided supplementation from enrolment until 6 weeks postpartum and in West et al. (2011), participants received supplements from enrolment to 12 weeks postpartum. One study did not explicitly state when the intervention ended (Kirkwood et al., 2010).

Of these 9 studies, one was excluded from meta‐analysis because no common outcomes of interest were reported (Prawirohartono et al., 2011). For a narrative synthesis of this study, see below under 'Other studies and comparisons not included in meta‐analysis'.

Eight studies were included in the meta‐analysis of vitamin A supplementation versus placebo (Cox et al., 2005; Darling et al., 2017; Kirkwood et al., 2010; Muslimatun et al., 2001; Semba et al., 2001; Tanumihardjo et al., 2002; West et al., 1999; West et al., 2011).

#### 5.18.1 Primary outcomes

Compared to placebo, vitamin A supplementation did not affect maternal mortality (average RR 0.90; 95% CI 0.68 to 1.18; studies = 3; participants = 124,002; random‐effects; Tau² = 0.02; I² = 35%; low quality evidence; Analysis 4.1).

#### 5.18.2 Secondary outcomes

Vitamin A supplementation, when compared to placebo, had no effect on maternal hemoglobin concentration (average MD 0.51 g/L; 95% CI ‐2.42 to 3.43; studies = 5; participants = 1,683; random‐effects; Tau² = 4.23; I² = 41%; Analysis 4.2), but may have slightly improved maternal serum retinol (average MD 0.13 umol/L; 95% CI ‐0.03 to 0.30; studies = 6; participants = 1,654; random‐effects; Tau² = 0.04; I² = 92%; Analysis 4.3), although the lower CI of the pooled estimate effect just crossed the line of no effect. Supplementation with vitamin A had effect on the risk of stillbirths (average RR 1.01; 95% CI 0.96 to 1.07; studies = 3; participants = 115,223; random‐effects; Tau² = 0.00; I² = 0%; Analysis 4.4).

#### 5.18.3 Subgroup Analysis

Due to insufficient data (this review required > or equal to three studies per subgroup of interest), subgroup analyses of the primary outcomes were not conducted based on maternal age, geographical region, sex of infant, baseline nutritional status in mothers (anemic vs. non‐anemic; undernutrition vs. normal nutrition based on body mass index: BMI <18.5; low stature vs. normal stature), duration of intervention, time of recruitment (preconception vs. first trimester vs. second trimester vs. third trimester of pregnancy), daily vs. intermittent IFA supplementation and dose of intervention.

### 5.19 Comparison V: Zinc versus Placebo

Thirteen studies evaluated prenatal zinc supplementation compared to placebo (Ahmad et al., 2016; Aminisani et al., 2009; Castillo‐Durán et al., 2001; Caulfield et al., 1999; Christian et al., 2003; Darling et al., 2017; Dijkhuizen et al., 2001; Hafeez et al., 2005; Merialdi et al., 2004; Osendarp et al., 2000; Prawirohartono et al., 2011; Saaka et al., 2009; Sorouri et al., 2016). Of these, 3 were conducted in Latin America and the Caribbean; 2 in Peru (Caulfield et al., 1999; Merialdi et al., 2004) and 1 in Chile (Castillo‐Durán et al., 2001). Two were conducted in the Middle East & North Africa; both conducted in Iran (Aminisani et al., 2009; Sorouri et al., 2016). Four were conducted in South Asia, with 2 in Bangladesh (Ahmad et al., 2016; et al., Osendarp et al., 2000); 1 in Pakistan (Hafeez et al., 2005) and 1 in Nepal (Christian et al., 2003).Two were conducted in East Asia and Pacific (Indonesia) Dijkhuizen et al., 2001; Prawirohartono et al., 2011, and two in sub‐Saharan Africa, in Ghana and Tanzania (Saaka et al., 2009 and Darling et al., 2017, respectively).

Three studies provided supplementation to the participants from enrolment until past delivery; Caulfield et al. (1999) and Merialdi et al. (2004) provided supplementation until 1 month postpartum; Ahmad et al., 2016 provided it until 6 months postpartum. Eight studies provided supplementation until delivery (Aminisani et al., 2009; Christian et al., 2003; Darling et al., 2017; Dijkhuizen et al., 2001; Hafeez et al., 2005; Osendarp et al., 2000; Saaka et al., 2009 and Sorouri et al., 2016). One study did not indicate until when supplementation was given (Castillo‐Durán et al., 2001).

One study was excluded from meta‐analysis because no common outcomes of interest were reported in this study (Prawirohartono et al., 2011) and thus data could not be pooled with the other studies. For a narrative synthesis of this study, see below under 'Other studies and comparisons not included in meta‐analysis'. Twelve studies contributed data to the various meta‐analyses for this comparison (Ahmad et al., 2016; Aminisani et al., 2009; Castillo‐Durán et al., 2001; Caulfield et al., 1999; Christian et al., 2003; Darling et al., 2017; Dijkhuizen et al., 2001; Hafeez et al., 2005; Merialdi et al., 2004; Osendarp et al., 2000; Saaka et al., 2009; Sorouri et al., 2016).

In addition to any pre‐specified, per‐protocol subgroup analyses, an exploratory post‐hoc analysis based on whether studies provided strictly zinc supplements vs. studies that provided zinc supplements along with additional micronutrients (e.g. iron folic acid as prenatal standard of care. Post‐hoc analyses were conducted for low birthweight, preterm births, small‐for‐gestational age and maternal serum/plasma zinc concentration.

#### 5.19.1 Primary outcomes

When compared to placebo, zinc supplementation probably made no difference to the risk of low birthweight infants (average RR 1.08; 95% CI 0.94 to 1.25; studies = 10; participants = 4,633; random‐effects; Tau² = 0.01; I² = 13%; moderate‐quality evidence; Analysis 5.1). In a post‐hoc analysis, there was no significant difference in the pooled effect estimates for low birthweight observed between studies that provided zinc supplements with additional micronutrients (mainly iron and folic acid, as prenatal standard of care) (average RR 1.02; 95% CI 0.80 to 1.31; studies = 8; participants = 4,176; random‐effects; Tau² = 0.03; I² = 28%; Analysis 5.1.1) and studies that that provided only zinc supplements compared to placebo (average RR 1.15; 95% CI 0.92 to 1.44; studies = 2; participants = 457; random‐effects; Tau² = 0.00; I² = 0%; Analysis 5.1.2) (test for subgroup differences: Chi² = 0.49, P = 0.48).

#### 5.19.2 Secondary outcomes

When compared to placebo, zinc supplementation in pregnancy had no effect on the risk of pre‐eclampsia/eclampsia (average RR 1.01; 95% CI 0.53 to 1.93; studies = 3; participants = 1,226; random‐effects; Tau² = 0.00; I² = 0%; Analysis 5.3), nor on the risk of preterm births (average RR 0.97; 95% CI 0.80 to 1.17; studies = 11; participants = 5,017; random‐effects, Tau² = 0.02; I² = 22%; Analysis 5.4). In a post‐hoc analysis, no difference was observed between studies that provided zinc supplements along with additional micronutrients (mainly iron folic acid supplementation as prenatal standard of care) and studies that provided only zinc supplementation compared to placebo.

Zinc supplementation compared to placebo also had no effect on reducing the risk of having an infant born SGA (average RR 1.05; 95% CI 0.97 to 1.13; studies = 3; participants = 2,174; random‐effects; Tau² = 0.00; I² = 0%; Analysis 5.5). No significant differences in effect were observed between studies that provided additional micronutrients (average RR 1.08; 95% CI 0.98 to 1.20; studies = 2; participants = 1,764; Analysis 5.5.1) and studies that gave strictly zinc (average RR 1.00; 95% CI 0.90 to 1.12; studies = 1; participants = 410; Analysis 5.5.2) (test for subgroup differences: Chi² = 0.99, P = 0.32).

Zinc supplementation may have slightly improved maternal serum zinc concentration (average MD 0.43 umol/L; 95% CI ‐0.04 to 0.89; studies = 5; participants = 1,202; random‐effects; Tau² = 0.16; I² = 79%; Analysis 5.6), although the lower confidence interval just crossed the line of no effect. Studies that provided strictly zinc supplements did not show significant differences in maternal serum zinc concentration (average MD 0.86 umol/L; 95% CI 0.67 to 1.05; studies = 2; participants = 485) compared to studies that provided additional micronutrients (average MD 0.01 umol/L; 95% CI ‐0.70 to 0.72; studies = 3; participants = 717) (test for subgroup differences: Chi² = 5.18, P = 0.02).

#### 5.19.3 Subgroup Analysis

Subgroup analyses were conducted for low birthweight based on geographical region. Please see below for results under heading 'Subgroup Analysis II: Zinc supplementation versus Placebo'.

### 5.20 Comparison VI: Iron versus Placebo

Thirteen studies assessed iron supplementation (Christian et al., 2003; Etheredge et al., 2015; Falahi 2010; Liu et al., 2013; Menendez et al., 1995; Ouladsahebmadarek et al., 2011; Preziosi et al., 1997; Tanumihardjo et al., 2002; Zeng et al., 2008; Zhao et al., 2015; Ziaei et al., 2007; Ziaei et al., 2008 and Korkmaz et al., 2014). Of these, 4 studies were conducted in the Middle East and North Africa, specifically in Iran (Falahi, 2010; Ouladsahebmadarek et al., 2011; Ziaei et al., 2007; Ziaei et al., 2008), while 3 studies were conducted in sub‐Saharan Africa (Etheredge et al., 2015; Menendez et al., 1995; Preziosi et al., 1997), representing Tanzania, The Gambia and Niger, respectively. Four studies were conducted in East Asia and Pacific; 3 were in China and 1 was in Indonesia (Liu et al., 2013; Tanumihardjo et al., 2002; Zeng et al., 2008; Zhao et al., 2015). One study was conducted in Nepal, in the South Asia region (Christian et al., 2003).

One study was excluded from meta‐analysis because no common outcomes of interest were reported (Korkmaz et al., 2014), and thus data could not be pooled with the other studies. For a narrative synthesis of this study, see below under 'Other studies and comparisons not included in meta‐analysis'. Of these, 12 studies contributed data to this comparison (Christian et al., 2003; Etheredge et al., 2015; Falahi, 2010; Liu et al., 2013; Menendez et al., 1995; Ouladsahebmadarek et al. 2011; Preziosi et al., 1997; Tanumihardjo et al. 2002; Zeng et al., 2008; Zhao et al. 2015; Ziaei et al., 2007; Ziaei et al., 2008).

The majority of included studies utilized supplements containing 60 mg of elemental iron (Etheredge et al., 2015; Falahi, 2010; Menendez et al., 1995; Tanumihardjo et al. 2002; Zeng et al., 2008; Zhao et al. 2015). Two studies gave participants 50 mg of iron (Ziaei et al., 2007; Ziaei et al., 2008), 3 studies gave participants 30 mg of iron (Christian et al., 2003; Liu et al., 2013; Ouladsahebmadarek et al. 2011), and 1 study gave 100 mg of iron (Preziosi et al., 1997).

In Tanumihardjo 2002 participants received 8.4 umol of vitamin A daily and in Menendez et al. (1995) and Etheredge et al. (2015) participants received 5 mg of folic acid daily. All women received intermittent preventative treatment for malaria in Etheredge et al. (2015).

In terms of duration of the intervention, the majority of included studies gave supplementation from enrolment to delivery (Christian et al., 2003; Etheredge et al., 2015; Menendez et al., 1995; Preziosi et al., 1997; Zeng et al., 2008; Zhao et al. 2015; Ziaei et al., 2007). Liu et al. (2013) gave supplementation from the first prenatal visit to delivery, Ouladsahebmadarek et al. (2011) gave supplementation from 13 weeks of gestation until delivery, and Tanumihardjo et al. (2002) gave supplementation for 8 weeks following enrolment. One study (Ziaei et al., 2008) gave iron supplementation from enrolment to 6 weeks postpartum.

In addition to any pre‐specified, per‐protocol subgroup analyses, an exploratory post‐hoc analysis based whether studies provided strictly iron supplementation compared to placebo vs. studies that provided iron supplements along with additional micronutrients (typically folic acid as prenatal standard of care). Post hoc analyses were conducted for maternal anaemia, maternal serum/plasma hemoglobin, maternal serum/plasma ferritin, and maternal iron deficiency.

#### 5.20.1 Primary Outcomes

When compared to placebo, iron supplementation in pregnancy improved maternal anaemia (average RR 0.53; 95% CI 0.43, to 0.65; studies = 6; participants = 15,737; random‐effects; Tau² = 0.06; I² = 84%; high quality evidence; Analysis 6.1). For maternal anaemia, we conducted a post‐hoc analysis of studies that gave strictly iron supplements versus placebo compared to studies where iron supplementation was combined with other supplements (most commonly folic acid as prenatal standard of care or vitamin A). There were no significant difference between subgroups, though only one study contributed to the analysis of strictly iron versus placebo (Analysis 6.1.1 and 6.1.2) (test for subgroup differences: Chi² = 0.44, P = 0.51).

When compared to placebo, iron supplementation demonstrated a moderate 12% reduction in the risk of low birthweight (average RR 0.88; 95% CI 0.78 to 0.99; studies = 4; participants = 17,257; random‐effects; Tau² = 0.00; I² = 0%; high quality evidence; Analysis 6.3) but no difference in perinatal mortality (average RR 0.88; 95% CI 0.71 to 1.08; studies =4; participants = 17,464; random‐effects; Tau² = 0.00; I² = 0%; high quality evidence; Analysis 6.4).

#### 5.20.2 Secondary outcomes

Iron supplementation, when compared to placebo, made no difference in reducing the risk of pre‐eclampsia/eclampsia during pregnancy (average RR 1.55; 95% CI 0.91 to 2.63; studies = 3; participants = 2,773; random‐effects; Tau² = 0.00; I² = 0%; Analysis 6.5). However, it did improve maternal hemoglobin concentration (average MD 7.80 g/L; 95% CI 4.08 to 11.52; studies = 11; participants = 25,916; random‐effects; Tau² = 36.63; I² = 99%; Analysis 6.6), and it improved maternal ferritin concentration (average MD 24.14 ug/L; 95% CI 10.83 to 37.45; studies = 9; participants = 5,045; random‐effects; Tau² = 370.94; I² = 100%; Analysis 6.7).

There were no significant differences between subgroups for maternal hemoglobin concentration (test for subgroup differences: Chi^2^ = 0.03, P = 0.85; Analysis 6.6.1 and 6.6.2). However, post‐hoc analysis showed that mothers who took strictly iron versus placebo had significantly lower serum ferritin (average MD of 7.09 ug/L; 95% CI 4.45 to 9.72; studies = 3; participants = 365; random‐effects; Tau² = 0.00; I² = 0%; Analysis 6.7.2) compared to mothers who took iron supplements plus additional micronutrients (average MD 32.87 ug/L; 95% CI 15.39 to 50.34; studies = 6; participants = 4,680; random‐effects; Tau² = 526.07; I² = 100%; Analysis 6.7.1) (test for subgroup differences: Chi² = 8.17, P = 0.004).

There was no impact of iron supplementation on maternal transferrin receptor concentration (average MD ‐0.16; 95% CI ‐0.96 to 0.65; studies = 3; participants = 2,431; random‐effects; Tau² = 0.46; I² = 99%; Analysis 6.8); however, iron supplementation improved iron deficiency when compared to placebo (average RR 0.54; 95% CI 0.40 to 0.74; studies = 4; participants = 2,522; random‐effects; Tau² = 0.06; I² = 82%; Analysis 6.9). In a post‐hoc analysis, the effect was significantly larger in studies that strictly gave iron (average RR of 0.34; 95% CI 0.23 to 0.51; studies = 2; participants = 2,177; random‐effects; Tau² = 0.00; I² = 0%; Analysis 4.9.2) when compared to studies that gave other supplements along with iron (average RR 0.67; 95% CI 0.54 to 0.83; studies = 2; participants = 345; random‐effects; Tau² = 0.02; I² = 78%; Analysis 4.9.1) (test for subgroup differences: Chi² = 8.32, P = 0.004).

When compared to placebo, iron supplementation did not have an effect on neonatal mortality (average RR 0.85; 95% CI 0.55 to 1.31; studies = 3; participants = 15,524; random‐effects; Tau² = 0.07; I² = 47%; Analysis 6.10), nor on the risk of preterm births (average RR 0.94; 95% CI 0.63 to 1.41; studies = 6; participants = 18,419; random‐effects; Tau² = 0.21; I² = 96%; Analysis 6.11). Iron supplementation also did not improve the risk of having babies considered SGA (average RR 1.04; 95% CI 0.87 to 1.24; studies = 4; participants = 6,549; random‐effects; Tau² = 0.02; I² = 71%; Analysis 6.12), nor did it improve the risk of infant mortality (average RR 1.10; 95% CI 0.84 to 1.45; studies = 3; participants = 14,748; random‐effects; Tau² = 0.00; I² = 0%; Analysis 6.13).

#### 5.20.3 Subgroup Analysis

Subgroup analyses were conducted for maternal anaemia based on geographical region and dose of iron. Please see below for results under heading 'Subgroup Analysis III: Iron supplementation versus Placebo'.

### 5.21 Comparison VII: Vitamin D versus Placebo

Eleven studies contributed data to this comparison (Asemi et al., 2013; Hossain et al., 2012; Khan et al., 2016; Mohammad‐Alizadeh‐Charandabi et al., 2015; Naghshineh & Sheikhaliyan, 2016; Roth et al., 2013 (AViDD); Roth et al., 2018 (MDIG); Sabet et al., 2012; Sablok et al., 2015; Sahu et al., 2009; Vaziri et al. 2016). Approximately half of the studies (n = 5) were conducted in the Middle East and North Africa region, specifically in Iran (Asemi et al., 2013; Mohammad‐Alizadeh‐Charandabi et al., 2015; Naghshineh & Sheikhaliyan, 2016; Sabet et al., 2012; Vaziri et al. 2016). The other half of the studies (n = 6) were conducted in the South Asia region; 2 were conducted in Bangladesh (Roth et al., 2013 (AViDD); Roth et al., 2018 (MDIG)), 2 in Pakistan (Hossain et al., 2012; Khan et al., 2016) and 2 in India (Sablok et al., 2015; Sahu et al., 2009). Six studies indicated that they gave participants supplementation from enrolment to delivery; enrolment was at various gestational ages, ranging from 12 weeks to 28 weeks of gestation (Hossain et al., 2012; Khan et al., 2016; Naghshineh & Sheikhaliyan, 2016; Roth et al., 2018 (MDIG); Sabet et al., 2012; Vaziri et al. 2016). Three studies were specific in their duration of intervention: Asemi et al. (2013) indicated that supplementation was given for nine weeks after enrolment; Mohammad‐Alizadeh‐Charandabi et al., 2015 stated supplementation was given for 60 days after enrolment and Roth et al., 2013 (AViDD) indicated that on average, women received supplementation for approximately 10 weeks after enrolment. Sablok et al. (2015) did not indicate the end of the supplementation period.

In addition to any pre‐specified, per‐protocol subgroup analyses, an exploratory post‐hoc analysis based whether studies provided strictly vitamin D supplementation compared to placebo vs. studies that provided vitamin D along with additional micronutrients (typically iron and folic acid as prenatal standard of care). Post hoc analyses were conducted for preterm births, SGA, maternal serum/plasma vitamin D, and mode of delivery ‐ caesarean section.

#### 5.21.1 Primary outcomes

There were no primary outcomes that were measured by studies included in this comparison.

#### 5.21.2 Secondary outcomes

Compared to placebo, vitamin D supplementation in pregnancy may have reduced the risk of preterm births (average RR 0.64; 95% CI 0.40 to 1.04; studies = 7; participants = 1,262; random‐effects; Tau² = 0.17; I² = 45%; Analysis 7.1), although the upper limit of the CI just crosses 1. In a post‐hoc analysis, mothers who took strictly vitamin D had a significantly reduced risk of preterm births (average RR 0.33; 95% CI 0.17 to 0.62; studies = 2; participants = 303; random‐effects; Tau² = 0.00; I² = 0%; Analysis 7.1.2) compared to mothers who took vitamin D plus additional micronutrients, typically iron‐folic acid as the prenatal standard of care (average RR 0.94; 95% CI 0.64 to 1.36; studies = 5, participants = 959; random‐effects; Tau² = 0.00; I² = 0%; Analysis 7.1.1) (test for subgroup differences: Chi² = 7.71, P = 0.006).

Vitamin D supplementation showed no effect on the risk of having SGA infants overall (average RR 0.93; 95% CI 0.57 to 1.53; studies = 3; participants = 851; random‐effects; Tau² = 0.12; I² = 63%; Analysis 7.2). Post‐hoc analysis indicated no significant differences in births considered SGA between studies that strictly gave vitamin D supplementation and studies that provided vitamin D plus additional micronutrients (test for subgroup differences: Chi² = 5.32, P = 0.02).

Vitamin D supplementation, when compared with placebo, improved maternal serum vitamin D concentrations (average MD 44.70 nmol/L; 95% CI 21.94 to 67.45; studies = 9; participants = 1,092; random‐effects, Tau² = 1149.40; I² = 99%; Analysis 7.3). No significant differences in maternal serum vitamin D concentration were noted when comparing studies that provided strictly vitamin D and studies that provided additional micronutrients (test for subgroup differences: Chi² = 0.16, P = 0.69).

Vitamin D supplementation demonstrated no effect on maternal serum calcium concentrations (average MD ‐0.06 mg/dL; 95% CI ‐0.21 to 0.09; studies = 5; participants = 759; random‐effects; Tau² = 0.02; I² = 84%; Analysis 7.4). Lastly, vitamin D supplementation, compared to placebo, had no effect on reducing the risk of caesarean section as a mode of delivery (average RR 1.05; 95% CI 0.94 to 1.18; studies = 5; participants = 1,063; random effects; Tau² = 0.00; I² = 0%; Analysis 7.5). Our post‐hoc analysis revealed no significant differences between groups for caesarean sections (test for subgroup differences: Chi² = 0.26, P = 0.61).

#### 5.21.3 Subgroup Analysis

Due to insufficient data (this review required more than or equal to three studies per subgroup of interest), subgroup analyses of the primary outcomes were not conducted based on maternal age, geographical region, sex of infant, baseline nutritional status in mothers (anemic vs. non‐anemic; undernutrition vs. normal nutrition based on body mass index: BMI <18.5; low stature vs. normal stature), duration of intervention, time of recruitment (preconception vs. first trimester vs. second trimester vs. third trimester of pregnancy), daily vs. intermittent IFA supplementation and dose of intervention.

### 5.22 Comparison VIII: Calcium vs. Placebo

Five studies assessed calcium supplementation versus placebo (Belizán et al.,^1997^; Jarjou et al., 2006; Kumar et al., 2009; López‐Jaramillo et al., 1997; Villar et al., 2006). One study was excluded from meta‐analysis because no common outcomes of interest were reported (Jarjou et al., 2006). For a narrative synthesis of this study, see below under 'Other studies and comparisons not included in meta‐analysis' section. Thus, 4 studies contributed data to this comparison (Belizán et al., 1997; Kumar et al., 2009; López‐Jaramillo et al., 1997; Villar et al., 2006). Two studies were conducted in Latin America and the Caribbean (Belizán et al., 1997; López‐Jaramillo et al., 1997) and 1 study was conducted in South Asia (Kumar et al., (2009). Villar et al. (2006) is a multi‐country study that was conducted in Peru, South Africa, Vietnam and India.

In addition to any pre‐specified, per‐protocol subgroup analyses, an exploratory post‐hoc analysis based whether studies provided strictly calcium supplementation compared to placebo vs. studies that provided calcium along with additional micronutrients (typically iron and folic acid as prenatal standard of care). Post‐hoc analyses were conducted for low birthweight, pre‐eclampsia/eclampsia, stillbirths, preterm births, and mode of delivery ‐ Caesarean section.

#### 5.22.1 Primary Outcomes

Compared to placebo, calcium supplementation in pregnancy did not improve the risk of low birthweight infants (average RR 0.99; 95% CI 0.95 to 1.04; studies = 3; participants = 9,498; random‐effects; Tau² = 0.00; I² = 0%; high quality evidence; Analysis 8.1). No significant difference in effect was noted between studies that provided strictly calcium and those that provided additional micronutrients (test for subgroup differences: Chi² = 0.12, P = 0.73).

#### 5.22.2 Secondary outcomes

Calcium supplementation, when compared to placebo, may have improved the risk of pre‐eclampsia/eclampsia (average RR 0.45; 95% CI 0.19 to 1.06; studies = 4; participants = 9,616; random‐effects; Tau² = 0.52; I² = 80%; Analysis 8.2), although the upper CI for the pooled effect estimate just crossed the line of no effect. In a post‐hoc analysis, compared to studies that provided additional nutrients along with the calcium supplement (mainly iron and folic acid as prenatal standard of care), women who received strictly calcium had a much greater reduction in the risk of pre‐eclampsia/eclampsia (average RR 0.30; 95% CI 0.17 to 0.52; studies = 3; participants = 1,304; random‐effects, Tau² = 0.00; I² = 0%; Analysis 8.2.2 compared to average RR 0.92; 95% CI 0.75 to 1.13; study = 1; participants = 8,312; random‐effects; Analysis 8.2.1), though only one study contributed to Analysis 8.2.1. Effects between subgroups were significantly different (test for subgroup differences: Chi² = 14.17, P = 0.0002).

Calcium supplementation probably did not reduce the risk of stillbirths (average RR 0.87; 95% CI 0.70 to 1.07; studies = 4; participants = 10,287; random‐effects; Tau² = 0.00; I² = 0%; Analysis 8.3). A post‐hoc analysis revealed no significant difference in stillbirths when comparing mothers who took calcium plus additional nutrients (average RR 0.86; 95% CI 0.69 to 1.07; studies = 1; participants = 8,378; Analysis 8.2.1) and those who took strictly calcium (average RR 0.97; 95% CI 0.44 to 2.15; studies = 3; participants = 1,909; Analysis 8.2.2) (test for subgroup differences: Chi² = 0.09, P = 0.77).

Calcium supplementation probably did not reduce the risk of preterm births (average RR 0.84; 95% CI 0.65 to 1.08; studies = 4; participants = 9,933; random‐effects; Tau² = 0.02; I² = 40%; Analysis 8.4), and there was no significant difference in effect between sub‐groups when conducting our post‐hoc analysis (test for subgroup differences: Chi² = 0.77, P = 0.38).

Lastly, calcium supplementation versus placebo had no effect on reducing the risk of Caesarean section as a mode of delivery (average RR 0.99, 95% CI 0.84 to 1.15; studies = 3; participants = 10,000; random‐effects; Tau² = 0.01; I² = 26%; Analysis 8.5). In a post‐hoc analysis, no significant differences in Caesarean sections were observed between studies that gave mothers strictly calcium and studies that provided additional nutrients alongside calcium (test for subgroup differences: Chi² = 0.50, P = 0.48).

##### Subgroup Analyses

Due to insufficient data (this review required > or equal to three studies per subgroup of interest), subgroup analyses of the primary outcomes were not conducted based on maternal age, geographical region, sex of infant, baseline nutritional status in mothers (anemic vs. non‐anemic; undernutrition vs. normal nutrition based on body mass index: BMI <18.5; low stature vs. normal stature), duration of intervention, time of recruitment (preconception vs. first trimester vs. second trimester vs. third trimester of pregnancy), daily vs. intermittent IFA supplementation and dose of intervention.

### 5.23 Subgroup Analysis I: MMN supplementation versus IFA

For studies comparing MMN supplementation versus IFA supplementation, we conducted subgroup analyses based on MMN formulation, duration of intervention, dose of iron in the intervention, and geographical region.

For maternal mortality, there were no significant differences between groups when comparing UNIMMAP plus adapted UNIMMAP to non‐UNIMMAP formulations of supplements (P for subgroup differences = 0.87; Analysis 9.1). In addition, there were no significant differences based on geographical region (P for subgroup differences = 0.61; Analysis 9.2), duration of intervention (P for subgroup differences = 1.00; Analysis 9.3), or dose of iron (P for subgroup differences = 1.00; Analysis 9.4).

Similarly, for the outcome maternal anaemia, there were no significant differences between groups when considering supplement formulation (P for subgroup differences = 0.65; Analysis 9.5), geographical region (P for subgroup differences = 0.51; Analysis 9.6), or dose of iron (P for subgroup differences = 0.83; Analysis 9.7).

For perinatal mortality, we found no significant differences between groups when examining MMN formulation (P for subgroup differences = 0.88; Analysis 9.8), geographical region (P for subgroup differences = 0.68; Analysis 9.9), or dose of iron (P for subgroup differences = 0.68; Analysis 9.10).

No significant difference was observed between studies that used the UNIMMAP formulation (average RR 0.74; 95% CI 0.61 to 0.90; studies = 11), compared to studies that used adapted UNIMMAP (average RR 0.88; 95% CI 0.85 to 0.91; studies = 3) and non‐UNIMMAP supplements (average RR 0.92; 95% CI 0.81 to 1.05; studies = 12; P for subgroup differences = 0.18; Analysis 9.11).

We observed a greater reduction in risk of low birthweight in women in studies conducted in Western Pacific (average RR 0.46; 95% CI 0.38 to 0.56; studies = 4), compared to studies conducted South Asia, Americas and Eastern Mediterranean regions (P for subgroup differences <0.00001; Analysis 9.12). Villar et al. (2006) was excluded from this subgroup analysis because it was a multi‐country study and the data was not disaggregated by country.

No significant difference in the reduction of low birthweight risk was observed between studies that utilized <60 mg of iron in their MMN supplements (average RR 0.79; 95% CI 0.69 to 0.89; studies = 18) and studies that utilized 60 mg of iron in their supplements (average RR 0.96; 95% CI 0.83 to 1.12; studies = 7; P for subgroup differences = 0.05; Analysis 9.13). Villar et al. (2006) was excluded from this subgroup analysis because the dosage of iron was not indicated in the methods.

### 5.24 Subgroup Analysis II: Zinc supplementation versus Placebo

For studies comparing zinc supplementation versus placebo, we conducted a subgroup analysis for the outcome low birthweight based on geographical region. We found no significant differences by region (P for subgroup differences = 0.39; Analysis 11.2).

### 5.25 Subgroup Analysis II: Iron supplementation versus Placebo

For studies examining iron supplementation versus placebo, we conducted subgroup analysis for the outcome maternal anaemia based on geographical region and dose of iron in the intervention supplement. For both region (P for subgroup differences = 0.49; Analysis 10.1) and dose (P for subgroup differences = 0.69; Analysis 10.2), we found no significant differences by subgroup.

Descriptive Summary of Additional Studies (not included in meta‐analyses):

### 5.26 Calcium plus vitamin D versus placebo supplementation

Diogenes et al. (2013) evaluated prenatal supplementation of calcium with vitamin D compared to placebo. Diogenes et al. (2013) was not included in any comparison for analysis in this review given that there was insufficient number of studies to conduct pooled analysis for calcium with vitamin D prenatal supplementation. The primary outcomes reported by Diogenes et al. (2013) were bone measurements in adolescent mothers at 5 and 20 weeks postpartum, including total bone mineral content (BMC) and total bone mineral density (BMD), and serum vitamin D concentration measured at baseline and postpartum. Serum vitamin D concentration levels in mothers who received prenatal supplementation were higher than those in mothers of the placebo group at 5 weeks and 20 weeks postpartum. In terms of bone measurements, the authors postulated that there may be an effect of increased calcium intake during pregnancy on reduced postpartum bone loss in adolescent mothers.

Taherian et al. (2002) also evaluated calcium plus vitamin D vs. placebo supplementation; however, it could not be pooled with the other two studies (Diogenes et al., 2013 and Asemi et al., 2016) given that they shared no common reported outcomes. The primary outcome was the prevalence of preeclampsia, whereby significant differences were observed in the frequency reduction of preeclampsia in women receiving only low‐dose aspirin daily or only calcium‐vitamin D supplements daily compared to placebo. No significant difference in the reduction of preeclampsia was observed between the aspirin and calcium‐vitamin D groups.

Note: Asemi et al. (2016) was included in the MMN vs. IFA comparison; however could not be pooled with Diogenes et al. (2013) or Taherian et al. (2002) to form a comparison group for calcium plus vitamin D supplementation versus placebo.

### 5.27 Iodine versus placebo

Gowachirapant et al. (2017) was the sole study that evaluated this type of supplementation. Participants were provided iodine or placebo tablets; there was no indication of provision of iron folic acid or multiple micronutrient supplements as additional prenatal supplementation. Primary outcomes reported were developmental outcomes in children, specifically verbal and performance IQ scores on WPPSI‐III and the global executive composite score from BRIEF‐P in children aged 5 to 6 years. Secondary outcomes included child's auditory performance, weight and height, maternal urinary iodine concentration, maternal thryoid stimulating hormone (TSH) concentration, maternal total thyroxine (T4) concentration. There were no significant differences between the iodine and placebo groups in terms of any child development outcomes. Maternal urinary iodine concentration was higher in the iodine group compared to the placebo group during pregnancy, but not at 6 weeks postpartum. In terms of maternal TSH and T4 concentrations, there were no significant differences between the intervention and control groups reported in by the authors.

### 5.28 LNS vs. placebo

Hambidge et al. (2019) was the only study that reported the effects of LNS vs. placebo; there was no report of iron‐folic acid or other multiple micronutrient given to both intervention arms and the control arm. The first intervention arm received the LNS supplement from the time of random assignment until delivery (Arm 1) while the second intervention arm received the LNS supplement during trimester 2 or 3 until delivery (Arm 2). Placebo group was Arm 3. The primary outcome of this study was newborn length‐for‐age z score (LAZ). Secondary outcomes included weight, head circumference (HC), BMI, and WAZ, HC‐for‐age (HCAZ) and BMI for age (BMIAZ) z scores, prevalence of low birthweight, small‐for‐gestational age and preterm infants. For all sites, there was no significant difference in LAZ between the two intervention arms (Arm 1 and Arm 2); however, small positive effects in LAZ were observed in Arm 1 compared to Arm 3 for the sites in Guatemala, Pakistan, India and Democratic Republic of Congo. Similar effects were noticed comparing Arms 2 and 3 for combined sites and Pakistan. Effect sizes for WAZ were same or lower than LAZ but followed similar patterns in which Arms 1 and 2 were greater than Arm 3.

### 5.29 Calcium vs. placebo

Jarjou et al. (2006) was excluded from meta‐analysis because the study did not report outcomes reported by other studies in the same comparison. Outcomes reported by Jarjou et al. (2006) include: maternal vitamin D serum concentration, bone mineral accretion in infants, birthweight (grams) and other growth measures, blood pressure, urine biomarkers (urinary calcium, phosphours and titratable acid) and calcium concentration in breast milk. No significant differences were noted by the authors between the intervention and control groups in terms of breastmilk concentrations of calcium, infant birth weight, infant growth or bone mineral status during the first year of life.

### 5.30 Iron vs. placebo

Korkmaz et al. (2014) was excluded from the iron vs. placebo comparison because it reported no common outcomes reported by other studies that evaluated the same type of supplementation. Outcomes reported by the study's authors include: maternal weight and weight gain during pregnancy, serum albumin levels, and oxidative stress markers such as y‐glutamyl transferase (GGT) levels. Oxidative stress markers, mainly GGT serum levels, were significantly higher in the iron intervention group compared to placebo (P < 0.001).

### 5.31 Vitamin A vs. placebo and Zinc vs. placebo

Prawirohartono et al. (2011): Although vitamin A vs. placebo and zinc vs. placebo are comparisons that have been meta‐analysed in this review, Prawirohartono et al. (2011) reported outcomes that were not common with reported outcomes in the other studies. The primary outcomes were postnatal child growth in terms of weight‐for‐age (WAZ), height‐for‐age (HAZ), and weight‐for‐height (WHZ) scores. The study found no effect on WAZ, HAZ and WHZ of prenatal supplementation of vitamin A, a combination of vitamin A and zinc, or zinc alone, compared to placebo. Other outcomes included prevalence of low birthweight infants, small‐for‐gestational age infants, and preterm births; no significant differences were noted between the vitamin A supplementation group, the zinc only supplementation group, and the placebo group for these outcomes.

### 5.32 Vitamin B12 vs. placebo

Duggan et al. (2014) was the only study that reported the effects of vitamin B12 supplementation vs. placebo. This study primarily measured vitamin B12 concentrations (pmol/L) in maternal plasma and breast milk. Secondary outcomes included mode of delivery ‐ Caesarean section, prevalence of low birthweight infants, mean birth weight and incidence of delivering an infant with intrauterine growth retardation (IUGR). Findings showed that women supplemented with vitamin B12 had higher vitamin B12 plasma concentrations, as well as, higher concentrations of vitamin B12 in their breastmilk. More mothers in the vitamin B12 arm underwent elective Caesarean section (18%) than mothers in the placebo group (10%) (P = 0.06). The frequency of LBW infants was not significantly different between the two groups. As well, mean birth weight did not differ significantly between the intervention and control arms. 25% of mothers in the vitamin B12 group delivered an infant with IUGR, compared to 34% of mothers in the placebo group (P = 0.11).

#### 5.32.1 Sensitivity Analyses

A sensitivity analysis was conducted to examine if any of the meta‐analyses were especially affected by the risk of bias assessments. Included studies that were assesed with a high risk of bias for one or more domain or studies that were assessed as unclear risk in two or more domains were removed from the meta‐analyses to see if this had any effect on the effect estimates of the outcomes. Diogenes et al. (2013), Duggan et al. (2014); Hambidge et al. (2019), Gowachirapant et al. (2017), Prawirohartono et al. (2011) and Taherian et al. (2002) were not included in any meta‐analyses, and thus sensitivity analysis of these studies rendered no effects on any outcomes.

For Comparison I: IFA vs. FA supplementation, two studies (Menendez et al., 1995; Zhao et al., 2015) were excluded; however, this exclusion did not significantly affect the findings for the outcomes.

For Comparison II: MMN vs. IFA supplementation, thirteen studies were excluded (Caulfield et al., 1999; Choudhury et al., 2012; Dijkhuizen et al., 2001; Friis et al., 2004; Hanieh et al., 2013; Huy et al., 2009; Kæstel et al., 2005; Moore et al., 2012; Muslimatun et al., 2001; Osrin et al., 2005; Ramakrishnan et al., 2003; Sunawang et al., 2009; Tofail et al., 2008). With the exclusion of Caulfield et al. (1999), Hanieh et al. (2013), Ramakrishnan et al. (2003), and Tofail et al. (2008), maternal serum/plasma folate concentration showed greater benefit with iron‐folic acid supplementation compared to MMN (average RR ‐3.60; 95% CI ‐5.62 to ‐1.58), although this sensitivity analysis includes only one study. With the exclusion of Friis et al. (2004), Hanieh et al. (2013), Kæstel et al. (2005), Osrin et al. (2005), Ramakrishnan et al. (2003), Sunawang et al. (2009), Tofail et al. (2008) and Zagré et al. (2007), there is minimal change to the effect of MMN supplementation on the risk reduction of stillbirths, although the upper limit of the confidence interval just crossed the line of no effect (average RR 0.95; 95% CI 0.96 to 1.01; compared to the original analysis, average RR 0.91; 95% CI 0.86 to 0.98). Similarly, with the removal of Friis et al. (2004), Kæstel et al. (2005), Moore et al. (2012), Osrin et al. (2005), Ramakrishnan et al. (2003), Sunawang et al. (2009), Tofail et al. (2008) and Zagré et al. (2007), there is minimal change to the effect of MMN supplementation on the reduced risk of SGA infants, although the upper limit of the confidence interval just crossed the line of no effect (average RR 0.95; 95% CI 0.96 to 1.01; compared to the original analysis, average RR 0.93; 95% CI 0.96 to 1.01). With the exclusion of Huy et al. (2009) and Osrin et al. (2005), the average risk ratio for underweight status in children changes from average RR 0.95 (95% CI 0.84 to 1.07) to average RR 1.01 (95% CI 0.97 to 1.06). Although the confidence intervals for the effect estimates both before and after the sensitivity analysis crosses the line of no effect, exclusion of these studies seemed to show no effect of MMN supplementation on child underweight status. Removal of Hanieh et al. (2013) seemed to show no effect of MMN supplementation for child development outcome: verbal comprehension and language (standard mean difference ‐0.01; 95% CI ‐0.19 to 0.16).

For Comparison III: LNS vs. MMN supplementation, two studies (Huybregts et al., 2009; Moore et al., 2012) were excluded. With the removal of Huybregts et al., 2009, a 75% reduction in the risk of stillbirths was reported (average RR 0.25; 95% CI 0.08 to 0.78) by LNS supplementation compared to MMN. The original analysis showed no effect of LNS supplements on the risk reduction of stillbirths (average RR 0.47; 95% CI 0.12 to 1.81).

For Comparison IV: vitamin A vs. placebo, six studies were excluded (Darling et al., 2017; Kirkwood et al., 2010; Muslimatun et al., 2001; Semba et al., 2001; Tanumihardjo et al., 2002; West et al., 2011). With the removal of Kirkwood et al. (2010) and West et al. (2011), a greater effect of vitamin A supplementation on maternal mortality was observed (average RR 0.62, 9% CI 0.37 to 1.04) although the confidence interval crosses one. The original analysis showed no observed effect of vitamin A on maternal mortality (average RR 0.90; 95% CI 0.68 to 1.18). Similarly, with the removal of Kirkwood et al. (2010), Muslimatun et al. (2001) Semba et al. (2001) Tanumihardjo et al. (2002) and West et al. (2011), maternal serum/plasma retinol concentration showed greater improvement with vitamin A supplementation (average MD 0.38 umol/L; 95% CI 0.14 to 0.62), although this analysis comprised of only one study, compared to the original analysis (average MD 0.13 umol/L; 95% CI ‐0.03 to 0.30).

For Comparison V: zinc vs. placebo, exclusion of six studies (Ahmad et al., 2016; Castillo‐Durán et al., 2001; Caulfield et al., 1999; Darling et al., 2017; Dijkhuizen et al., 2001; and Osendarp et al., 2000), showed no change in the findings for the outcomes.

For Comparison VI: iron vs. placebo, exclusion of six studies (Falahi 2010; Menendez et al., 1995; Ouladsahebmadarek et al., 2011; Tanumihardjo et al., 2002; Preziosi et al., 1997 and Zhao et al., 2015) showed no change in the findings for the outcomes.

For Comparison VII: vitamin D vs. placebo, exclusion of six studies (Hossain et al., 2012; Mohammad‐Alizadeh‐Charandabi et al., 2015; Sabet et al., 2012; Sablok et al., 2015; Sahu et al., 2009; Vaziri et al., 2016) showed no change in the findings for the outcomes.

For Comparison VIII: calcium vs. placebo, there were no studies to remove in the sensitivity analysis.

### 5.33 Post‐Hoc Analysis based on declarations of conflict of interest

A post‐hoc analysis was conducted to examine if any effect estimates were influenced by studies where a conflict of interest was declared. Five studies reported a conflict of interest (Ashorn et al., 2015; Dewey et al., 2009; Osrin et al., 2005; West et al., 2014; Zeng et al., 2008), and exclusion of these studies did not significant affect the findings for the reported outcomes.

## 6 DISCUSSION

### 6.1 Summary of main results

We identified 314 included papers across 72 studies (involving 451,723 women) as eligible for inclusion in this review; however, 64 studies (involving 439,649 women) contributed data to the analyses. This review summarizes the current evidence on the effect of several micronutrient and vitamin supplementations during pregnancy on fetal, infant, maternal and child outcomes. Of eligible studies, seven assessed IFA versus folic acid supplementation; thirty‐four studies assessed MMN supplementation versus IFA supplementation or placebo; four studies assessed LNS supplementation versus MMN supplementation or placebo; thirteen studies assessed iron supplementation versus no iron or placebo; thirteen studies assessed zinc supplementation versus no zinc or placebo; nine studies assessed vitamin A supplementation versus no vitamin A or placebo; eleven studies assessed vitamin D supplementation versus no vitamin D or placebo; and six studies assessed calcium supplementation versus no calcium or placebo.

Supplementation with IFA compared to folic acid showed a large and significant effect on the risk of maternal anaemia in the third trimester of pregnancy, reducing the risk by 48% (average RR 0.52, 95% CI 0.41 to 0.66; studies = 5; participants = 15,540; moderate‐quality evidence); and a smaller but significant, 12% reduction in the risk of low birthweight babies (average RR 0.88, 95% CI 0.78 to 0.99; studies = 4; participants = 17,257; high‐quality evidence).

MMN supplementation compared to iron with or without folic acid supplementation probably made no difference on maternal mortality (average RR 1.04 95% CI 0.71 to 1.51; studies = 7; participants = 75,051; moderate quality of evidence) and demonstrated no significant effect on perinatal mortality (average RR 1.00, 95% CI 0.90 to 1.11; studies = 16; participants = 92,769; high‐quality evidence). MMN supplementation also did not have an important effect on maternal anaemia (average RR 0.95, 95% CI 0.82 to 1.10; studies = 16; participants = 23,556; high quality evidence) and had little effect on iron deficiency anaemia as well (average RR 1.12, 95% CI 0.62 to 2.02; studies = 4; participants = 1595; low‐quality evidence). However, MMN supplementation in pregnancy, when compared to iron with or without folic acid, demonstrated a significant effect, a 15% reduction, on the risk of having a low birthweight baby (average RR 0.85, 95% CI 0.77 to 0.93; studies = 28; participants = 79,972). MMN supplementation also demonstrated a small and significant effect on the risk of stillbirths (average RR 0.91, 95% CI 0.86 to 0.98; studies = 22; participants = 96,772), and a smaller but significant effect on the risk of SGA infants (average RR 0.93; 95% CI 0.88 to 0.98; studies = 19; participants = 52,965). The risk of preterm births may have improved with MMN supplementation (average RR 0.96; 95% CI 0.91 to 1.01; studies = 29; participants = 99,855), although the confidence interval just crossed the line of no effect.

Regarding child outcomes, children of mothers who had been supplemented with MMN showed a significant effect on the risk of diarrhea in children (average RR 0.84; 95% CI 0.76 to 0.92; studies = 4; participants = 2,556) and a positive effect on executive function (standard MD 0.09; 95% CI 0.01 to 0.17; studies = 3; participants = 2,511).

Our subgroup analyses that compared studies based on MMN formulation (UNIMMAP vs. Adapted UNIMMAP vs. non‐UNIMMAP formulations) showed that MMN supplementation had a greater effect in reducing the risk of low birthweight among women in the subgroup of studies that used the UNIMMAP formulation (average RR 0.74; 95% CI 0.61 to 0.90; studies = 11), compared to studies that used adapted UNIMMAP (average RR 0.88; 95% CI 0.85 to 0.91; studies = 3) and non‐UNIMMAP supplements (average RR 0.92; 95% CI 0.81 to 1.05; studies = 12; P for subgroup differences = 0.18). We also conducted a post‐hoc exploratory analysis based on the number of components provided in the MMN supplement, specifically comparing studies that used MMN supplements containing >4 micronutrients to studies whose MMN formulation contained <or equal to 4 micronutrients. MMN supplements with more than 4 components showed a large significant effect on reducing the risk of having a low birthweight baby (average RR 0.79; 95% CI 0.71 to 0.88; studies = 19; participants = 68,138); whereas, MMN supplements with far fewer components indicated little to no effect (average RR 1.02; 95% CI 0.88 to 1.18; studies = 7; participants = 3,669) (Test for subgroup differences: Chi² = 11.51; P = 0.0007).

When compared to MMN, LNS supplementation made no difference to perinatal mortality (average RR 1.01, 95% CI 0.62 to 1.65; studies = 3; participants = 2771; low‐quality evidence) and probably did not impact the risk of low birthweight (average RR 0.92, 95% CI 0.74 to 1.13; studies = 4; participants 2727; moderate‐quality evidence) or SGA (average RR 0.96, 95% CI 0.86 to 1.07; studies = 4; participants = 859).

Vitamin A supplementation compared to placebo made no difference to any outcome examined.

Zinc supplementation compared to placebo had no major effect on any outcome examined, with the exception of serum/plasma zinc levels, which were probably improved among mothers (average MD 0.43 umol/L; 95% CI ‐0.04 to 0.89; studies = 5; participants = 1,202), although the confidence interval crossed the line of no effect.

Iron supplementation, when compared to no iron or placebo, showed a large significant effect on maternal anaemia, reducing the risk by 47% (average RR 0.53, 95% CI 0.43 to 0.65; studies = 6; participants = 15,737; moderate‐quality evidence) and a significant effect on low birthweight, reducing the risk by 12% (average RR 0.88, 95% CI 0.78 to 0.99; studies = 4; participants = 17,257; high‐quality evidence), but had no important effect on perinatal mortality (average RR 0.88, 95% CI 0.71 to 1.08; studies = 4; participants = 17,464; moderate quality evidence).

When compared to placebo, supplementation with vitamin D may have reduced the risk of preterm births (average RR 0.64; 95% CI 0.40 to 1.04; studies = 7; participants = 1,262); however, it had no impact on SGA.

When compared to placebo, calcium supplementation probably improved rates of pre‐eclampsia/eclampsia among mothers (average RR 0.45; 95% CI 0.19 to 1.06; studies = 4; participants = 9,616), but made no difference to other outcomes that were assessed in this review.

### 6.2 Overall completeness and applicability of evidence

This review included 72 studies that evaluated all forms of micronutrient and vitamin supplementation during pregnancy on maternal and child outcomes, of which 64 trials contributed data to pooled analyses. The remaining eight studies that could not be pooled with other studies were narratively synthesized. To our knowledge, this is the first systematic review that has compiled all evidence from 1995, across all low‐ and middle‐income countries, regarding multiple forms of micronutrient and vitamin supplementation in one report, covering a wide breadth of outcomes, including maternal, fetal, infant outcomes pertaining to morbidity, mortality, nutrition and biochemical statuses, and child outcomes such as longitudinal developmental outcomes. Certain forms of supplementation could not be evaluated in this review given the insufficient number of studies per reported outcome; a minimum of three studies was required. While comparisons involving iodine, folic acid, vitamin B12 and vitamin D plus calcium supplementation could not be evaluated, this review comprehensively synthesized eight other important and common forms of supplementation, involving 439,649 pregnant women. It is important to note that while data regarding LNS supplementation was captured, there were still not enough studies to reliably evaluate its benefits compared to MMN supplementation; further research is highly recommended.

This review also aimed to assess effectiveness along with efficacy. Despite the numerous antenatal and maternal health programmes that exist in , the majority of programme evaluations did not fit our inclusion criteria related to study design (RCTs or quasi experimental studies). Thus, only two effectiveness trials were included: Friis et al. (2004) and Huy et al. (2009). A recent systematic review by Hossain et al. (2017) reviewed the evidence on health and nutrition programs aimed at reducing stunting in low‐ and middle‐income countries. The authors found that while nutrition focused interventions are necessary, a combination of nutrition‐specific interventions (e.g. maternal supplementation during pregnancy) and nutrition‐sensitive interventions (e.g. social safety nets, maternal mental health and women's empowerment, to name a few) is most successful at reducing stunting in the child population. They listed health and nutrition during preconception, pregnancy and lactation, and micronutrient supplementation for mothers and children as important nutrition specific interventions, under which the interventions of this review fall. Given that stunting is an indicator of chronic malnutrition, and has long term, generational effects on maternal and child health, this highlights the importance of maternal supplementation interventions to improve the nutrition of mothers and children generally. The authors also noted that all of the studies included in this review were cross‐sectional or observational in nature, which limits causality of findings. None of the programmes were designed with a true control area or group in which there was no intervention. As evaluation of micronutrient and vitamin supplementation continues, it is important to consider the design of these effectiveness studies and programmes so as to be included in future meta‐analyses.

While we included longer‐term outcomes in this review, such as those commonly reported in the under‐five child and developmental outcomes, very few studies examine these outcomes in terms of maternal supplementation. Despite the few number of studies, our findings showed that MMN supplementation, compared to iron with or without folic acid, did improve diarrhea in children (studies = 4), child serum retinol concentrations (studies = 3), and executive function (studies = 3). A number of studies did report other child development outcomes, such as general intelligence (studies = 8) and motor function (studies = 7); however, there is significant diversity in the tests and scores used to measure, age of participants and time of assessment. While MMN supplementation showed little to no effect, the wide diversity of scores utilized by researchers and the range of ages evaluated may lend to difficulty in pooling results effectively. Apart from MMN supplementation compared to iron with or without folic acid, no other comparison in this review included studies that reported on long‐term child outcomes.

According to protocol, subgroup analyses were conducted for primary outcomes permitting data availability. Subgroup analyses were conducted for the MMN vs. IFA, zinc vs. placebo and iron vs. placebo comparisons for geographical region, dose of iron (mg) in the MMN or IFA supplement, and MMN supplement composition. However, subgroup analyses could not be conducted based on a variety of other descriptive measures including maternal age, sex of infant, baseline nutritional status of the mother, duration of intervention and dose of intervention, with the exception of the subgroup analysis based on MMN composition (UNIMMAP vs. adapted UNIMMAP vs. non‐UNIMMAP formulations). As such, conclusions could not be drawn regarding the optimal doses and especially, duration of supplementation. Given that the majority of all included studies in this review recruited only pregnant women who began supplementation mostly during the first and second trimester, and not those in the preconception period, this review cannot comment on the importance of preconception supplementation, and its short and long‐term effects on maternal, fetal, infant, and child health outcomes. This is a notable limitation given the breadth of evidence supporting the importance of preconception health in mothers to reduce maternal and child morbidity and mortality related to pregnancy (Dean et al., 2014, Lassi et al., 2014, Mason et al., 2014). A systematic review by Dean et al. (2014) examined the role of preconception and peri‐conception interventions in mothers in all economic settings, including micronutrient and vitamin supplementation, and concluded that greater positive impacts and benefits for mother and child may be noticed if preconception supplementation was implemented for all women of reproductive age, compared to periconception supplementation. It is possible that the lack of effect of supplementation on mortality outcomes such as maternal, infant, and perinatal mortality, and remaining uncertainty of the effects of micronutrient supplementation on preterm births, miscarriage, and congenital anomalies, might be due to supplementation beginning too late. Especially in low‐ and middle‐income settings where the majority of women have poor nutritional status and health prior to conception, preconception care and supplementation may have great impact and benefit on maternal, infant and child health outcomes in these populations.

Post‐hoc exploratory analyses were also conducted, comparing studies that provided strictly the micronutrient in question to studies who provided additional micronutrients along with the supplement (mainly iron and folic acid as prenatal standard of care). The aim was to dissociate possible differences in the true effects of the micronutrient itself, without the possible modifier effects of other micronutrients. This data disaggregation is unique to this review. Post‐hoc analyses were conducted for the following comparisons: zinc vs. placebo, iron vs. placebo, vitamin D vs. placebo and calcium vs. placebo. These analyses did demonstrate differences in the true effects of the micronutrients itself for certain outcomes, such as zinc serum/plasma concentration, maternal iron deficiency, and pre‐eclampsia/eclampsia, where provision of strictly zinc, iron and calcium, respectively noted greater improvements compared to supplementation with additional micronutrients. The calcium vs. placebo comparison showed that additional micronutrients had greater benefit for stillbirths, preterm births and mode of delivery ‐ Caesarean section; which further substantiates the support for multiple micronutrient supplementation during pregnancy. For the MMN vs. IFA comparison, post‐hoc analyses were conducted for certain outcomes to dissociate possible differences in MMN supplements containing many components, the common definition of a MMN supplement, compared to the review's definition of a MMN supplement, which was any supplement containing at least three micronutrient/vitamin components. Generally, MMN supplements with a greater number of micronutrients performed better in terms of improving maternal and child health outcomes, especially for outcomes like low birthweight, stillbirth, and SGA.

Another limitation of this review is our exclusion of certain populations, including women who were anaemic, women at high risk of pregnancy disorders, or those who had chronic illnesses, because many of these conditions are quite prevalent in maternal populations living in low‐ and middle‐income countries. As a point of distinction, we excluded studies that had specifically recruited these groups of women, though understand that many of these conditions (e.g. anaemia) are present in the women included within these analyses. As a result, for some of our outcomes, we may not have seen similar effects on maternal and infant outcomes as reported by other reviews, who mostly did include these populations. For example, a recent systematic review, Smith et al. (2017) disaggregated data by maternal anaemia status, and reported a larger effect by MMN supplementation on birth outcomes in women with poorer nutritional status than women who were non‐anemic. Given our exclusion criteria, our review did not have enough studies to comment and disaggregate data by factors such as maternal nutritional status at baseline, and thus this review is unable to identify specific groups that may benefit the most from different types of supplementation. Similarly, our review is also unable to examine data and comment on the adolescent population due to data limitations. Our inclusion criteria included healthy pregnant women of any age, which would certainly capture pregnant adolescents given adolescent pregnancies are common in low‐ and middle‐income countries. However, none of our studies, though they included adolescent participants, disaggregated data by age group. Thus, this review is unable to identify if specific effects of maternal supplementation exist in this particular demographic. These may be considerations for future updates and research.

Another limitation was our lack of ability to disaggregate birth outcomes by sex. Recent evidence highlights differences for certain outcomes between female and male infants. Smith et al. (2017) noted that MMN supplementation consistently reduced mortality in female infants by 15% compared to their male counterparts. Similarly, Christian et al. (2005), also reported a significant 17% reduction in infant mortality amongst female infants compared to their male counterparts who saw no reduction. Biological mechanisms are still unclear, but some studies (Lee et al., 2009, West et al., 2014) postulate that the difference in mortality might be caused by difference in birth size between sexes. This might be an important consideration in a future update of this review, to consider the sex differences for important outcomes, especially mortality, across all forms of micronutrient and vitamin supplementation; Smith et al. (2017) and Christian et al. (2005) solely evaluated MMN versus IFA supplementation.

### 6.3 Quality of the evidence

Risk of bias assessment was conducted for individual studies based on the following criteria: random sequence generation (selection bias), allocation concealment (selection bias), blinding of participants, personnel and outcome assessment (performance bias and detection bias), incomplete outcome data (attrition bias), selective reporting (reporting bias) and other potential forms of bias. Each of these categories were assessed as high or low risk, or unclear risk, if there was insufficient information to make any judgement. To evaluate the quality of the available evidence in this review, assessment by outcome was conducted using the GRADE methodology, as outlined in the methods. A 'Summary of Findings' table was created for the primary outcomes of maternal anaemia, maternal mortality, perinatal mortality and low birthweight for Comparisons I to VIII. For Comparison I (IFA vs. FA), we graded low birthweight as high quality, and down‐graded maternal anaemia and perinatal mortality to moderate‐quality for funnel plot asymmetry, possibly indicating publication bias.

For Comparison II (MMN vs. IFA), maternal anaemia, low birthweight and perinatal mortality were assessed as high‐quality, while maternal mortality was downgraded by one to moderate‐quality because the confidence interval of the point estimate fails to exclude important harm. Maternal iron deficiency anaemia was graded very low for large heterogeneity, the small number of events and a potential publication bias as indicated by funnel plot asymmetry. For Comparison III (LNS vs. MMN), low birthweight was downgraded to moderate quality, and perinatal mortality was downgraded to low‐quality because the risk of bias assessment was high or unclear for a substantial proportion of studies in the outcomes. Perinatal mortality was further downgraded due to the small number of events and because the confidence interval of the point estimate failed to exclude important harm. For Comparison IV (Vitamin A vs. placebo), maternal mortality was downgraded by two to low‐quality because a substantial proportion of studies were assessed as high or unclear risk of bias, low number of events, and the confidence interval crossed the threshold for decision making. For Comparison V (Zinc vs. placebo) was downgraded to moderate quality for the outcome low birthweight because the confidence interval of the point estimate does not exclude important harm. For Comparison VI (Iron vs. Placebo), both low birthweight and perinatal mortality were graded high quality while maternal anaemia was downgraded by one to moderate‐quality due to funnel plot asymmetry, indicating possible publication bias. Comparison VII (vitamin D vs. placebo) reported no primary outcomes. For Comparison VIII (calcium vs. placebo), the outcome low birthweight was graded as high quality.

A sensitivity analysis was conducted to examine if any of the meta‐analyses were especially affected by the risk of bias assessments. Included studies that were assessed with a high risk of bias for one or more domain or studies that were assessed as unclear risk in two or more domains were removed from the meta‐analyses to see if this had any effect on the effect estimates of the outcomes. Diogenes et al. (2013), Duggan et al. (2014), Hambidge et al. (2019), Gowachirapant et al. (2017), Prawirohartono et al. (2011) and Taherian et al. (2002) were not included in any meta‐analyses, and thus sensitivity analysis of these studies rendered no effects on any outcomes. Overall, sensitivity analyses did not significantly affect the findings for the majority of outcomes across all comparisons. An additional post‐hoc analysis was conducted to examine if any effect estimates were influenced by studies where a conflict of interest was declared. Five studies reported a conflict of interest (Ashorn et al., 2015; Dewey et al., 2009; Osrin et al., 2005; West et al., 2014; Zeng et al., 2008), and exclusion of these studies did not significant affect the findings for the reported outcomes.

### 6.4 Potential biases in the review process

As per systematic review guidelines, all results screening, data extraction, and risk of bias assessments were completed in duplicate by two independent review authors. Given that the Cochrane methodology was closely followed, it is unlikely that this review is affected by biases in the review process. One potential bias may be the selection of pre‐1995 as a cut‐off date. For certain micronutrients, such as vitamin A, a number of studies evaluating their supplementation and effects on maternal, infant and child health outcomes were conducted pre‐1995.

### 6.5 Agreements and disagreements with other studies or reviews

To better support our explanations within this section, we have provided a table that compares our findings to those of recent and related Cochrane reviews. Please see Table 17.

**Table 17:** Comparison table of systematic reviews

Authors	Year	Title	Type of Review	Comparison/Intervention	Number of Included Studies	Search Date	Outcomes	Reported Relative Risk [95% Confidence Interval] (# of studies)	Our Review ‐ Relative Risk [95% Confidence Interval] (# of studies)
Hofmeyr, G.J., Lawrie, T. A., Atallah Á. N., Torloni, M. R.	2018	Calciumsupplementation during pregnancy for preventing hypertensive disorders and related problems (Review)	Cochrane	1) high‐dose calcium supplementation (at least 1 g daily of elementa calcium) during pregancy with placebo 2) low‐dose calcium supplementation during pregnancy with placebo	27	18 Sept. 2017	Low birth weight	0.85 [0.72, 1.01] (9)	0.99 [0.95, 1.04] (3)
							Pre‐eclampsia/Eclampsia	0.45 [0.31, 0.65] (12)	0.45 [0.19, 1.06] (4)
							Stillbirths	0.90 [0.74, 1.09] (6)	0.87 [0.70, 1.07] (4)
							Preterm births	0.76 [0.60, 0.97] (9)	0.84 [0.65, 1.08] (4)
Das, J. K., Hoodbhoy, Z., Salam, R. A., Bhutta, A. Z., Valenzuela‐Rubio, N. G., Weise Prinzo, Z., Bhutta, Z. A.	2018	Lipid‐based nutrient supplements formaternal, birth, and infant developmental outcomes (Review)	Cochrane	LNS supplementation to IFA or MMN	4	May 2018	Low birth weight	0.92 [0.74, 1.13] (3)	0.92 [0.75, 1.13] (4)
							Neonatal mortality	0.88 [0.36, 2.15] (1)	0.81 [0.45, 1.45] (3)
							Preterm births	1.15 [0.93, 1.42] (3)	1.15 [0.93, 1.42] (4)
							SGA	0.95 [0.84, 1.07] (3)	0.96 [0.86, 1.07] (4)
Palacios, C., Kostiuk, L. K., Peña‐Rosas, J. P.	2019	Vitamin D supplementation for women during pregnancy	Cochrane	Vitamin D vs placebo/no intervention	22	12 July 2018	Preterm births	0.66 [0.31, 1.30 (7)	0.64 [0.40, 1.04] (7)
							Caesarean section	0.98 [0.80, 1.21] (10)	1.05 [0.94, 1.18] (5)
Roth, D., Leung, M., Mesfin, E., Qamar, H., Watterworth, J., Papp, E.	2017	Vitamin D supplementation during pregnancy: state of the evidence from a systematic review of randomised trials	Non‐Cochrane	Vitamin D supplementation versus placebo or no treatment	43	Sept. 2017	Preterm births	0.52 [0.26, 1.05] (4)	0.64 [0.40, 1.04] (7)
							SGA	0.60 [0.40, 0.90] (5)	0.93 [0.57, 1.53] (3)
							Caesarean section	1.02 [0.93, 1.12] (17)	1.05 [0.94, 1.18] (5)
Peña‐Rosas, J. P., De‐Regil, L. M., Garcia‐Casal, M. N., Dowswell, T.	2015	Daily oral iron supplementation during pregnancy	Cochrane	Supplementation with iron versus no treatment/placebo	33	26 Feb. 2015	Low birth weight	0.63 [0.30, 1.32] ( 6)	0.88 [0.78, 0.99] (4)
							Preterm births	0.82 [0.58, 1.14] (6)	0.94 [0.63, 1.41] (6)
							Maternal Anemia	0.29 [0.19, 0.47] (14)	0.53 [0.43, 0.65] (6)
							Pre‐eclampsia/Eclampsia	0.96 [0.06, 14.43] (1)	1.55 [0.91, 2.63] (3)
Peña‐Rosas, J. P., De‐Regil, L. M., Garcia‐Casal, M. N., Dowswell, T.	2015	Daily oral iron supplementation during pregnancy	Cochrane	Iron‐folic acid supplementation versus folic acid only supplementation	5	26 Feb. 2015	Low birth weight	0.88 [0.78, 1.00] (4)	0.88 [0.78, 0.99] (4)
							Preterm births	0.97 [0.87, 1.08] (4)	0.96 [0.64, 1.44] (5)
							Neonatal mortality	0.91 [0.71, 1.18] (4)	0.85 [0.55, 1.31] (3)
							Maternal Anemia	0.34 [0.21, 0.55] (2)	0.52 [0.41, 0.66] (5)
Ota, E., Mori, R., Middleton, P., Tobe‐Gai, R., Mahomed, K., Miyazaki, C., Bhutta, Z. A.	2015	Zinc supplementation for improving pregnancy and infant outcome	Cochrane	Zinc supplementation versus no zinc or placebo	20	31 Oct 2014	Preterm births	0.86 [0.76, 0.97] (16)	0.97 [0.80, 1.17] (11)
							Low birth weight	0.93 [0.78, 1.12] (14)	1.08 [0.94, 1.25] (10)
							SGA	1.02 [0.94, 1.11] (8)	1.05 [0.97, 1.13] (n = 3)
							Pre‐eclampsia/Eclampsia *included pregnancy hypertension	0.83 [0.64, 1.08] (7)	1.01 [0.53, 1.93] (n = 3)
McCauley, M. E., van den Broek, N., Dou, L., Othman, M.	2015	Vitamin A supplementation during pregnancy formaternal and newborn outcomes	Cochrane	Vitamin A supplementation alone versus placebo or no treatment	10	30 March 2015	Maternal mortality	0.88 [0.65 to 1.20] (4)	0.90 [0.67 to 1.18] (3)
							Stillbirths	1.04 [ 0.98, 1.10] (2)	1.01 [0.96, 1.07] (3)
Bhutta, Z. A., Imdad, A., Ramakrishnan, U., Martorell, R.	2012	Is it time to replace iron folate supplements in pregnancy with multiple micronutrients?	Non‐Cochrane	Multiple micronutrients (5+ micronutrients) versus multiple micronutrients (<3 micronutrients; mainly iron folate)	7	15 Sept 2011	Low birth weight	0.86 [ 0.81, 0.91] (16)	0.82 [0.74, 0.90] (26)
							SGA	0.83 [0.73, 0.95] (8)	0.92 [0.88, 0.97] (18)
							Maternal Anemia	1.03 [0.94, 1.12] (7)	1.02 [0.82, 1.10] (16)
Ramakrishnan, U., Grant, F. K., Goldenberg, T., Bui, V., Imdad, A., Bhutt, Z. A.	2012	Effect of Multiple Micronutrient Supplementation on Pregnancy and Infant Outcomes: A Systematic Review	Non‐Cochrane	Multiple micronutrients (5+ micronutrients) versus multiple micronutrients (<3 micronutrients; mainly iron folate)	46	April 2011	Low birth weight	0.86 [ 0.81, 0.91] (16)	0.82 [0.74, 0.90] (26)
							SGA	0.83 [0.73, 0.95] (8)	0.92 [0.88, 0.97] (18)
							Preterm births	0.99 [0.96, 1.03] (9)	0.97 [0.91, 1.02] (27)
							Stillbirths	1.00 [0.84, 1.21]) (10)	0.92 [0.86, 0.99] (20)
							Neonatal mortality	0.97 [0.87, 1.09] (9)	0.98 [0.90, 1.06] (16)
Christian, P.	2005	Evidence of Multiple Micronutrient Supplementation in Pregnancy	Non‐Cochrane	Multiple micronutrient versus iron‐folic acid or Iron or Placebo	15	Not reported	Low birth weight	0.88 [0.86, 0.90] (15)	0.82 [0.74, 0.90] (26)
							Preterm births	0.90 [0.84, 0.96](10)	0.97 [0.91, 1.02] (27)
							Stillbirths	0.94 [0.87, 1.01] (12)	0.92 [0.86, 0.99] (20)
							SGA	0.91 [0.84, 1.00] (7)	0.92 [0.88, 0.97] (18)
							Neonatal mortality	0.98 [0.91, 1.05] (12)	0.98 [0.90, 1.06] (16)
Kawai, K., Spiege, D., Shankar, A. H., Fawzi, W. W.	2011	Maternal multiple micronutrient supplementation and pregnancy outcomes in developing countries: meta‐analysis and meta‐regression	Non‐Cochrane	Multiple micronutrients versus iron‐folic acid supplementation	17	1 Aug 2010	Low birth weight	0.86 [0.79, 0.93] (15)	0.82 [0.74, 0.90] (26)
							preterm birth	0.99 [0.95, 1.03] (14)	0.97 [0.91, 1.02] (27)
							SGA	0.85 [0.78, 0.93] (15)	0.82 [0.74, 0.90] (26)
							Perinatal mortality	1.05 [0.90, 1.22] (11)	1.00 [0.90, 1.11] (16)
							Neonatal mortality	1.08 [0.92, 1.26] (11)	0.98 [0.90, 1.06] (16)
Keats, E. C., Haider, B. A., Tam, E., Bhutta, Z. A.	2019	Multiple‐micronutrient supplementation for women during pregnancy (Review)	Cochrane	1) Multiple micronutrient with iron and folic acid versus iron, with or without folic acid 2) Multiple micronutrient versus placebo	20	23 Feb 2018	Preterm births	0.95 [0.90, 1.01] (18)	0.97 [0.91, 1.02] (27)
							SGA	0.92 [0.88, 0.97] (17)	0.92 [0.88, 0.97] (18)
							Low birth weight	0.88 [0.85, 0.91] (18)	0.82 [0.74, 0.90] (26)
							Perinatal mortality	1.00 [0.90, 1.11] (15)	1.00 [0.90, 1.11] (16)
							Stillbirths	0.95 [0.86, 1.04] (17)	0.92 [0.86, 0.99] (20)
							Neonatal mortality	1.00 [0.89, 1.12] (14)	0.98 [0.90, 1.06] (16)
							Maternal anaemia	1.04 [0.94, 1.15] (9)	1.02 [0.95, 1.10] (16)
							Maternal mortality	1.06 [0.72, 1.54] (6)	1.06 [0.72, 1.54] (6)
							Miscarriage	0.99 [0.94, 1.04] (12)	0.99 [0.94, 1.04] (13)
							Caesarean section	1.13 [0.99, 1.29] (5)	1.08 [0.97, 1.22] (9)
							Congenital anomalies	1.34 [0.25, 7.12] (2)	1.02 [0.46, 2.24] (6)
Smith, E. R., Shankar, A. H., Wu, S. F., Aboud, S., et al.	2017	Modifiers of the effect of maternal multiple micronutrient supplementation on stillbirth, birth outcomes, and infant mortality: a meta‐analysis of individual patient data from 17 randomised trials in low‐income and middle‐income countries	Non‐Cochrane	Multiple micronutrient supplementation versus iron‐folic acid supplementation	17	20 July 2015	Stillbirths	0.97 [0.85, 1.11] (16)	0.92 [0.86, 0.99] (20)
							Neonatal mortality	0.99 [0.89, 1.09] (12)	0.98 [0.90, 1.06] (16)
							Infant mortality	0.97 [0.88, 1.06] (8)	1.18 [0.96, 1.44] (5)
							Low birth weight	0.86 [0.81, 0.92] (17)	0.82 [0.74, 0.90] (26)
							Preterm births	0.93 [0.87, 0.98] (16)	0.97 [0.91, 1.02] (27)
							SGA	0.94 [0.90, 0.98] (16)	0.92 [0.88, 0.97] (18)

#### 6.5.1 MMN vs. IFA

The findings of this review on the reduction of LBW as a result of MMN supplementation corroborate those of other systematic reviews (Bhutta et al., 2012; Christian et al., 2005; Kawai et al., 2011; Keats et al., 2019; Margetts et al., 2009; Ramakrishnan et al., 2012). In comparison to the most recent Cochrane update, Keats 2019 found that MMN supplementation led to a 12% reduction (average RR 0.88; 95% CI 0.8 to 0.91) in the risk of LBW across 19 studies; our findings report a slightly greater reduction (average RR 0.85; 95% CI 0.77 to 0.93; participants = 79,972) in a pooled analysis of 28 studies. MMN supplementation did not have a significant effect on perinatal mortality and maternal mortality, which is similar to the findings of other systematic reviews, including the most recent update of the Cochrane review (Kawai et al., 2011; Keats et al., 2019; Ramakrishnan et al., 2012). The RR estimates for perinatal mortality and maternal mortality in this review were the same as those reported in Keats et al. (2019): 1.00 (0.90 to 1.11); studies = 16; participants = 92,769 and 1.04 (0.71 to 1.51); studies = 7; participants = 75,051, respectively. Keats et al. (2019) reported an average RR of 1.04 (95% CI 0.94 to 1.15; studies = 10), for the outcome maternal anaemia, which is similar to our finding, despite that we included more studies (average RR 1.02; 95% CI 0.82 to 1.10; studies = 16; participants = 23,556).

Subgroup analysis comparing studies based on MMN formulation showed that MMN supplementation with more components (UNIMMAP or supplements with >4 micronutrients) lend to improved birth outcomes in pregnant women compared to MMN supplementation with fewer components (non‐UNIMMAP or supplements with <or equal to 4 micronutrients), especially for outcomes such as low birthweight. Ramakrishnan et al. (2012) meta‐analyzed 16 studies that compared supplementation of women during pregnancy with five or more micronutrients compared with supplementation with three or fewer micronutrients, while Bhutta et al. (2012) compared 7 studies whose MMN supplements had >5 components versus <3 supplements. Both reviews noticed a more effective reduction in risk of low birthweight and SGA infants in studies that provide MMN supplements with >than three micronutrients, compared to studies with few components in their MMN supplements, which aligns with this review.

Although not primary outcomes in our review, commonly reported outcomes in other systematic reviews include stillbirth, preterm birth, SGA, and neonatal mortality. Our findings demonstrate a greater reduction in the risk of stillbirths (average RR 0.91; 95% CI 0.86 to 0.98; studies = 22; participants = 96,772) when compared to other reviews; Ramakrishnan et al. (2012) showed no beneficial effect, while Keats et al. (2019) showed a minimal reduction (average RR 0.95; 95% CI 0.90‐1.01; studies = 17). We found that MMN supplementation slightly improved preterm birth (average RR 0.96, 95% CI 0.91 to 1.01; studies = 29; participants = 99,855), while Keats et al. (2019) found a similar reduction (average RR 0.95, 95% CI 0.90 to 1.01; studies = 19). For SGA, our review showed an 7% reduction in the risk of SGA (average RR 0.93; 95% CI 0.88 to 0.98; studies = 19; participants = 52,965], which was similar to Keats et al. (2019) (average RR 0.92; 95% CI 0.88 to 0.97; studies = 17) and Smith et al. (2017) (average RR 0.94 95% CI 0.90 to 0.98; studies = 16). However, greater reductions in SGA with MMN supplementation were reported in Ramakrishnan et al. (2012) (average RR 0.83, 95% CI 0.73 to 0.95) and Kawai et al. (2011) (average RR 0.85, 95% CI 0.78 to 0.93). Consistent with several other reviews (Bhutta et al., 2012; Christian et al., 2005; Kawai et al., 2011; Keats et al., 2019; Margetts et al., 2009; Ramakrishnan et al., 2012; Smith et al., 2017) we found that MMN supplementation has no important effect on neonatal mortality.

There are several possible reasons for these differences. Firstly, our review included a greater number of studies. Second, in Ramakrishnan et al. (2012), MMN must have contained at least five micronutrients, whereas we included any supplement with at least three. Third, Kawai et al. (2011) included maternal populations that were undernourished, while our review only included healthy populations as participants. Kawai et al. (2011) noted that MMN supplementation resulted in greater reductions in maternal and infant outcomes, such as low birthweight, mortality and SGA in anaemic pregnant woman compared to non‐anaemic pregnant women. Therefore, it is possible that baseline maternal nutritional status may be an important modifier of the effect of MMN supplementation on maternal and infant outcomes.

#### 6.5.2 LNS vs MMN

For LNS versus MMN supplementation, the findings of this review corroborate the findings of a recent Cochrane review (Das et al., 2018). Both demonstrated little to no impact on LBW (average RR 0.92; 95% CI 0.74 to 1.13; studies = 4; participants = 2,727; Das et al. (2018) average RR 0.92; 95% CI 0.74 to 1.13; studies = 3), preterm birth (average RR 1.15; 95% CI 0.93 to 1.42; studies = 4; participants = 2,953; Das et al. (2018) average RR 1.15; 95% CI 0.93 to 1.42; studies = 3), SGA (average RR 0.96; 95% CI 0.86 to 1.07; studies = 4; participants = 2,716; Das et al. (2018) average RR 0.95; 95% CI 0.84 to 1.07; studies = 3), and neonatal mortality (average RR 0.81 95% CI 0.45 to 1.45; studies = 3; participants = 2,727; Das et al. (2018) average RR 0.88 95% CI 0.36 to 2.15; studies = 1).

#### 6.5.3 Iron vs Placebo

Our findings also corroborate those of a recent review conducted by Peña‐Rosas et al. (2015), who looked at iron supplementation compared to placebo or no treatment. Our findings show that iron or IFA supplementation, compared to no iron, reduces maternal anaemia (average RR 0.53; 95% CI 0.43, to 0.65; studies = 6; participants = 15,737) and LBW (average RR 0.88; 95% CI 0.78 to 0.99; studies = 4; participants = 17,257). Comparatively, Peña‐Rosas et al. (2015) reported a slightly greater reduction in maternal anaemia (average RR 0.29; 95% CI 0.19 to 0.47; studies = 14) and a greater reduction in LBW (average RR 0.63; 95% CI 0.30 to 1.32; studies = 6). In terms of preterm birth, Peña‐Rosas et al. (2015) indicated a greater reduction in the risk of preterm births due to iron supplementation in mothers (average RR 0.82; 95% CI 0.58 to 1.14; studies = 6); compared to this review (average RR 0.94; 95% CI 0.63 to 1.41; studies = 6; participants = 18,419). Some of these difference may be due to the fact that Peña‐Rosas et al. (2015) included studies with anaemic populations at baseline, whereas these studies would have been excluded in this review as the focus was on healthy pregnant participants.

#### 6.5.4 IFA vs FA

Our findings also corroborate those of a recent review conducted by Peña‐Rosas et al. (2015), who looked at iron‐folic acid supplementation compared to folic acid alone. Both reviews reported similar reductions in the risk of LBW infants (average RR 0.88; 95% CI 0.78 to 0.99; studies =4; participants = 17,257; Peña‐Rosas et al. (2015) average RR 0.88; 95% CI 0.78 to 1.00; studies = 4), and preterm births (average RR 0.96; 95% CI 0.64 to 1.44; studies = 5; participants = participants = 17,637; Peña‐Rosas et al. (2015) average RR 0.97; 95% CI 0.87 to 1.08; studies = 4). While perinatal mortality was not an outcome reported in Peña‐Rosas et al. (2015), our results are similar for neonatal mortality (average RR 0.85; 95% CI 0.55 to 1.31; studies = 3; participants = 15,794; Peña‐Rosas et al. (2015) average RR 0.91; 95% CI 0.71 to 1.18; studies = 4), whereby there was no significant reduction. Peña‐Rosas et al. (2015) reported a greater reduction in the risk of maternal anaemia (average RR 0.34; 95% CI 0.21 to 0.55; studies = 2) compared to our findings (average RR 0.52; 95% CI 0.41 to 0.66; studies = 5; participants = 15,540).

#### 6.5.5 Zinc vs Placebo

Compared to Ota et al. (2015), a recent review of zinc supplementation, our review found that zinc supplementation did not have an important effect on LBW (average RR 1.08 95% CI 0.94 to 1.25; studies = 10; participants = 4,633). However, Ota et al. (2015) showed a 7% reduction in the risk of LBW (average RR 0.93 95% CI 0.78 to 1.12; studies = 14). In terms of preterm births, our review found a minimal effect of zinc supplementation on the reduction of preterm births (average RR 0.97, 95% CI 0.80 to 1.17; studies = 11; participants = 5,017), while Ota et al. (2015) showed a greater reductive effect (average RR 0.86; 95% CI 0.76 to 0.97; studies = 16). Ota et al. (2015) includes additional studies, which may lend to a greater certainty in these results compared to our own. These studies were not eligible for inclusion in our review because they were conducted in high‐income settings, included wrong patient populations, or could not be retrieved.

#### 6.5.6 Vitamin A vs. Placebo

Similar to McCauley et al. (2015), there were very few studies that assessed vitamin A supplementation versus no vitamin A or placebo. In terms of maternal mortality, our findings (average RR 0.90 95% CI 0.67 to 1.18, studies = 3; participants = 124,002) are in accord with McCauley et al. (2015) (average RR 0.88, 95% CI 0.65 to 1.20; studies = 4) in that vitamin A supplementation does not improve maternal mortality. As well, both review report no effect of vitamin A supplementation on risk reduction of stillbirths (average RR 1.01; 95% CI 0.96 to 1.07; studies = 3; participants = 115,223; McCauley et al. (2015) average RR 1.04; 95% CI 0.98 to 1.10; studies = 2).

#### 6.5.7 Vitamin D vs placebo

Several recent reviews have looked at the effects of vitamin D supplementation on pregnancy outcomes, against which many of our findings are aligned. We found that vitamin D, compared to placebo, may reduce preterm birth (average RR 0.64 95% CI 0.40 to 1.04; studies = 7; participants = 1,262). This finding differs from that which were reported in Roth et al. (2017) (average RR 1.00 95% CI 0.77 to 1.30; studies = 15), and by Palacios et al. (2019), a recent update to the De‐Regil et al. (2016) Cochrane review (average RR 0.66; 95% CI 0.31 to 1.30; studies = 7). In terms of SGA, our findings (average RR 0.93, 95% CI 0.57 to 1.53; studies = 3; participants = 851) were not similar to Roth et al. (2017), which showed a significant reduction in risk of SGA (average RR 0.60, 95% CI 0.40 to 0.90; studies = 5). A possible reason for this might be that we excluded studies with patient populations that were unhealthy or had am explicitly reported micronutrient deficiency, such as vitamin D deficiency; whereas, Roth et al. (2017) included all studies that evaluated vitamin D supplementation compared to placebo, regardless of health status of the maternal populations. Including populations who are micronutrient‐deficient at baseline may modify the effect of the intervention. While not a primary outcome in this review, caesarean section as a mode of delivery is a common outcome reported across many vitamin D supplementation studies and reviews. Our results show little to no effect on this outcome (average RR 1.05, 95% CI 0.94 to 1.18; studies = 5; participants = 1,063), similar to Roth et al. (2017) (average RR 1.02; 95% CI 0.93 to 1.12; studies = 17), and Palacios et al. (2019) (average RR 0.98, 95% CI 0.80 to 1.21; studies = 10).

#### 6.5.8 Calcium vs Placebo

Our findings show that calcium supplementation compared to placebo probably improves rates of pre‐eclampsia/eclampsia among mothers (average RR 0.45; 95% CI 0.19 to 1.06; studies = 4; participants = 9,616). Hofmeyr et al. (2018) reported similar effects for pre‐eclampsia (average RR 0.45; 95% CI 0.31 to 0.65; studies = 12), though confidence intervals around our estimate are much wider, lending to more uncertainty around the pooled effect. Both this review and Hofmeyr et al. (2018) reported effects of calcium supplementation on the reduction of preterm birth; however Hofmeyr et al. (2018) reported a great reduction in preterm birth (average RR 0.76; 95% CI 0.60 to 0.97; studies = 9) compared to our findings (average RR 0.84; 95% CI 0.65 to 1.08; studies = 4; participants = 9,933). Lastly, our findings on the reduction of low birthweight as a result of calcium supplementation compared to placebo differs from that reported by a recent systematic review by Hofmeyr et al. (2018). Our findings show no effect of calcium supplementation on the reduction in the risk of low birthweight in the intervention group compared to the control group (average RR 0.99; 95% CI 0.95 to 1.04; studies = 3; participants = 9,498), while Hofmeyr et al. (2018) found a 15% reduction in the risk of low birthweight. The differences may be explained by the different inclusion criteria for participants between the reviews. While this review excluded participants who had a reported risk for a hypertensive disorder, such as pre‐eclampsia, Hofmeyr et al. (2018) included these participants, as well as, those who were reported at screening to be calcium‐deficient. These differences at baseline may have contributed to greater effects of supplementation in studies reported by Hofmeyr et al. (2018) compared to our findings.

## 7 AUTHORS' CONCLUSIONS

### 7.1 Implications for practice

The findings of this review suggest that micronutrient and vitamin supplementation improves certain maternal, fetal, infant and child outcomes. Across all comparisons, it was noted that micronutrient and vitamin supplementation have little to no effect on mortality outcomes, specifically maternal, neonatal and perinatal mortality, which is consistent with observations from other systematic reviews. Generally, the findings of this review support micronutrient and vitamin supplementation in all women irrespective of maternal nutritional status during pregnancy, especially in low‐ and middle‐income settings, as it provides improvements in maternal, fetal and infant outcomes. This review also shows that MMN supplementation should be considered as the preferred option for standard prenatal care, when compared to IFA. This is especially evident for outcomes such as stillbirths, SGA and low birthweight. MMN supplementation also showed possible effects on preterm births. Novel findings of this review are the improvements of diarrhea and serum/plasma retinol in children with MMN supplementation, although more research is required to interpret these results with greater reliability. IFA or iron supplementation seem to be better options compared to other micronutrients for improving maternal anaemia. LNS supplementation compared to MMN supplementation showed no improved effect on maternal and child outcomes; however, very few studies were included. Further study is needed to understand LNS supplementation compared to MMN supplementation. Calcium supplementation may reduce the risk of pre‐eclampsia and eclampsia in pregnancy. Vitamin A supplementation indicated a slight improved effect on serum/plasma retinol in mothers, while zinc supplementation seems to have minimal effect on maternal and infant outcomes.

### 7.2 Implications for research

Given that the benefits of micronutrient and vitamin supplementation have been well corroborated by many other systematic reviews, future research should now consider conducting more detailed analyses to identify optimal dosages and formulations of micronutrient supplements, and duration of supplementation. Similarly, consideration should be made to identifying specific groups of mothers that could benefit from different forms of supplementation, including different dosages, based on maternal nutritional status at baseline or screening (e.g. by maternal BMI, stature, or anaemia status). A specific group about which focused and disaggregated data is needed are pregnant adolescents, who may have modified supplementation effects and needs compared to pregnant adult women.

While this review notes improvements in certain child health outcomes, few studies conduct follow‐up evaluations amongst their child participants. Future research should consider evaluating longitudinal outcomes in the under‐five child and older children on health and early childhood development. It would be beneficial to evaluate the long‐term effects of micronutrient and vitamin supplementation during pregnancy, especially given that evidence indicates that certain child outcomes are indicators for chronic health issues and possible generational effects, such as stunting as an indicator of chronic malnutrition.

Future research should also consider disaggregating data by sex for infant and child outcomes, as increasing evidence has shown sex‐specific differences for certain morbidity and mortality outcomes. Better understanding of the biological mechanisms behind these sex‐specific differences should also be considered in future research. However, it is important to note that these sex‐specific differences should not be used as evidence to encourage selective supplementation of mothers during pregnancy; regardless of fetal sex, all mothers should receive the standard of care and micronutrient and vitamin supplementation provided by programmes within their setting. Lastly, future programmes and effectiveness studies should consider designs other than observational as methods of evaluation, and should consider a true comparison or control area. This may lead to more robust evidence and a stronger causality of findings in these large‐scale programmes.

## CONTRIBUTIONS OF AUTHORS

Emily Keats and Aamer Imdad have methodological, statistical, and information retrieval expertise. Zulfiqar Bhutta has content expertise. Christina Oh, Tamara Chau and Dina S. Khalifa received training in systematic review methods.

## DECLARATIONS OF INTEREST

The authors are not aware of any conflicts of interest arising from financial or researcher interests.

## DIFFERENCES BETWEEN PROTOCOL AND REVIEW

An exploratory post‐hoc analysis was included, for both primary and secondary outcomes, comparing studies that provided strictly the single micronutrient to studies that provided additional micronutrients along with the micronutrient in question (e.g. studies that strictly provided calcium and studies that provided calcium with iron‐folic acid as the standard of care). The aim of this analysis was to dissociate the true effects of the micronutrient itself from the possible modifier effects of the additional micronutrients. For the MMN vs. IFA comparison, a post‐hoc analysis was conducted comparing studies that provided MMN supplements with >4 micronutrients to studies that provided supplements with only 3 or 4 micronutrients.

## PUBLISHED NOTES

### Characteristics of studies

#### Characteristics of included studies

Ahmad 2015

**Table:** 

**Methods**	This double‐blind, randomized controlled trial was conducted in two urban slum areas, Kamrangir Char and Hazaribagh,in Dhaka, Bangladesh. Dates of study: 2011‐2013
**Participants**	Pregnant women between 11‐13 weeks of gestation were eligible for enrolment. Exclusion criteria included: systemic or chronic disease, previous complicated pregnancies or abortion and congenital anomaly. Informed written consent was obtained from participating mothers. Zinc group (n = 28), Placebo group (n = 28)
**Interventions**	1. Zinc group (n = 28) received a tablet to be consumed daily, containing 20 mg of zinc sulfate.2. Placebo group (n = 28) received a cellullose placebo tablet to be consumed daily
**Outcomes**	Primary outcomes: low birth weight (<2500 g)Secondary outcomes: preterm births and serum/plasma zinc concentrations (umol/L)
**Notes**	Compliance with tablet consumption was assessed by counting the remaining tablets in the bubble packs during weekly home visits. **Declarations of Interest:** Authors declare no conflict of interest **Funding Sources:** Nestle Foundation

Risk of bias table

**Table:** 

Bias	Authors' judgement	Support for judgement
Random sequence generation (selection bias)	Unclear risk	Quote: "placebo‐controlled, double‐blindand randomized trial" Comment: insufficient information about the sequence generation process to permit judgment
Allocation concealment (selection bias)	Unclear risk	Comment: insufficient information about the allocation concealment process to permit judgment
Blinding of participants and personnel (performance bias)	Low risk	Quote: "Zinc and placebo tablets were identical in color, shape, size,odor and test."Comment: probably done
Blinding of outcome assessment (detection bias)	Low risk	Quote: "Zinc and placebo tablets were identical in color, shape, size,odor and test."Comment: probably done
Incomplete outcome data (attrition bias)	High risk	Quote: "Seventy percentage of the mother‐infant pairs completed the study protocol; the high number lost to follow‐up could be due to the >1‐year‐long study period since enrollment and the high migration rate of the slum population in Dhaka."Comment: Reasons for attrition were reported. Attrition and exclusions were balanced across the treatment arms.
Selective reporting (reporting bias)	Low risk	Comment: All outcomes presented in the methods section were reported in the paper.
Other bias	Low risk	Comment: not other bias identified

Aminisani 2009

**Table:** 

**Methods**	This double‐blind randomized controlled trial was conducted in an urban setting of the Ardabil District (province) of Iran. Dates of study: not reported, Dates of recruitment: April 2004 to March 2005.
**Participants**	196 pregnant women between 16‐20 weeks of gestation were recruited from urban healthcare centres, andn provided informed consent. Exclusion crtieria included: hypertension, diabetes, renal disease, history of prematurity, premature rupture of membranes (PROM) or low birth weight infants. Zinc group (n = 98), Placebo group (n = 98)
**Interventions**	1. Zinc group: received a daily tablet containing 50 mg of zinc sulphate.2. Placebo group: received a daily tablet that is similar in appearance to the zinc tabletParticipants in both arms received 1 mg folic acid and 30 mg of ferrous sulphate tablets daily, to be consumed at night. The zinc sulphate and placebo tablets were consumed at midmorning.
**Outcomes**	This study was included in two comparisons in this review: 1) Zinc vs. Placebo: Outcomes analyzed included low birth weight, pre‐eclampsia and preterm births2) MMN vs. IFA: Outcomes analyzed included low birth weight, stillbirths and preterm births
**Notes**	Information on compliance was assessed and recorded monthly by trained midwives. Women whose compliance was less than 70% were excluded from analysis. **Declarations of Interest:** none reported **Funding sources:** Research Fund of Ardabil University of Medical Sciences

Risk of bias table

**Table:** 

Bias	Authors' judgement	Support for judgement
Random sequence generation (selection bias)	Unclear risk	Quote: "The study was double‐blind and the women were randomly assigned to receive either 50 mg daily elemental zinc as zinc sulphate (n = 98), or placebo (n = 98)." Comment: insufficient information about the sequence generation process to permit judgment
Allocation concealment (selection bias)	Low risk	Quote: "The zinc sulphate and placebo capsules were made by the Alhavi Company. The placebo capsules were similar to the zinc sulphate capsules in both shape and blister packing. The zinc sulphate was coded as "A" and the placebo as "B", however, both interviewers and participants remained blind of these codes until the study was completed"Comment: probably done
Blinding of participants and personnel (performance bias)	Low risk	Quote: "The zinc sulphate and placebo capsules were made by the Alhavi Company. The placebo capsules were similar to the zinc sulphate capsules in both shape and blister packing. The zinc sulphate was coded as "A" and the placebo as "B", however, both interviewers and participants remained blind of these codes until the study was completed"Comment: probably done
Blinding of outcome assessment (detection bias)	Low risk	Quote: "The zinc sulphate and placebo capsules were made by the Alhavi Company. The placebo capsules were similar to the zinc sulphate capsules in both shape and blister packing. The zinc sulphate was coded as "A" and the placebo as "B", however, both interviewers and participants remained blind of these codes until the study was completed"Comment: probably done
Incomplete outcome data (attrition bias)	Low risk	Quote: "A total of 196 subjects were recruited and randomly assigned in two groups. Six refused to participate (3 in zinc and 3 in placebo group), remaining (190) were included in the study. Subjects who consumed placebo or supplement irregularly (on fewer than 21 days per month or 70% compliance) were excluded; 11 (5.8%) were excluded (6.3% in the zinc group and 5.3% in placebo group NS), they withdrew prior to the outcome being measured, but 93.2% of the participants consumed Zinc supplement or placebo regularly."Comment: exclusion and attrition were balanced across treatment arms
Selective reporting (reporting bias)	Low risk	Comment: All outcomes presented in the methods section were reported in the paper.
Other bias	Low risk	Comment: No other bias was identified

Asemi 2013

**Table:** 

**Methods**	This was a randomized, double‐blind, placebo‐controlled clinical trial conducted in Kashan, Iran. Dates of study: March 2012 to September 2012.
**Participants**	Pregnant womem (n = 48), aged 18‐40 years old, primigravida and a singleton pregnancy, at 25 weeks of gestation were eligible for the study. Exclusion criteria included: pre‐eclampsia, hypertension, gestational diabetes, intrauterine fetal death, history of rheumatoid arthritis, hepatic or renal disease/failure, metabolic bone disease and malabsorption, thryoid, parathyroid or adrenal diseases, smoking, taking medications including nonsteroidal anti‐inflammatory drugs and aspirinVitamin D group (n = 24), placebo group (n = 24).
**Interventions**	Vitamin D group: received 400 IU of Vitamin D per day for nine weeks.Placebo group: received a placebo, micro‐crystalline cellulose tablet per day for nine weeksParticipants were asked not to alter their routine diets and physical activity regimen. They were also asked not to consume any supplements other than the ones provided to them in the study. Women in both treatment arms received 400ug of folic acid from the beginning of the study and 60 mg of iron (as ferrous sulphate) daily from the second trimester.
**Outcomes**	Outcomes examined were serum/plasma vitamin D (nmol/L), serum/plasma calcium (mg/dL) and preterm births
**Notes**	Compliance was monitored once a week through phone interviews. **Declarations of interest**: none reported **Funding sources:** none reported

Risk of bias table

**Table:** 

Bias	Authors' judgement	Support for judgement
Random sequence generation (selection bias)	Low risk	Quote: "Random assignment was performed by the use of computer‐generated random numbers" Comment: probably done
Allocation concealment (selection bias)	Low risk	Quote: "A trained midwife at the maternity clinic performed the randomized allocation sequence and assigned participants to the groups"Comment: probably done
Blinding of participants and personnel (performance bias)	Low risk	Quote: "Vitamin D supplements and placebo were provided by Shahre Daru Company. Placebo pills contained microcrystalline cellulose and were packed in identical tablets and coded by the producer to guarantee blinding"Comment: probably done
Blinding of outcome assessment (detection bias)	Low risk	Quote: Measurements in the laboratory were…"formed in a blinded fashion, in duplicate, in pairs (before/after intervention) at the same time, in the same analytical run, and in random order to reduce systematic error and interassay variability."Comment: Probably done
Incomplete outcome data (attrition bias)	Low risk	Comment: only three participants in each group were lost to follow‐up
Selective reporting (reporting bias)	Unclear risk	There is insufficient information to allow judgement
Other bias	Low risk	Comment: No other bias was identified

Asemi 2016

**Table:** 

**Methods**	This was a randomized double‐blind placeob‐controlled trial conducted in Kashan, Iran. Dates of study: March 2012‐September 2012
**Participants**	Pregnant women (n = 46) who were at 25 weeks of gestation were included in the study for nine weeks. Exclusion criteria included: those with pre‐eclampsia, placenta abruption and gestational diabetes mellitus.Calcium‐Vitamin D group (n = 28) and Placebo (n = 28).
**Interventions**	1. Calcium‐Vitamin D group (n = 28): received tablets containing approximately 475‐600 mg of calcium and 190‐240 IU of Vitamin D.2. Placebo group (n = 28): received identical tablets to the intervention group containing microcrystalline cellulose.Participants in both arms consumed 400 ug of folic acid every day and 50 mg of ferrous sulfate.
**Outcomes**	This study was Included in the MMN vs. IFA comparison. The outcome analyzed was mode of delivery ‐ Caesarean section.Note: while this study also measures calcium‐vitamin D vs. placebo, this comparison was excluded from analysis due to insufficient number of studies per outcome.
**Notes**	**Declarations of Interest:** Authors report no conflict of interest **Funding sources:** grant from the Vice Chancellor for Research, Kashan University of Medical Sciences, Kashan, Iran.

Risk of bias table

**Table:** 

Bias	Authors' judgement	Support for judgement
Random sequence generation (selection bias)	Low risk	Quote: "We used random numbers, taken from a computer program to do random assignment." Comment: probably done
Allocation concealment (selection bias)	Unclear risk	Comment: insufficient information to make judgement
Blinding of participants and personnel (performance bias)	Low risk	Quote: "double‐blind"Comment: probably done
Blinding of outcome assessment (detection bias)	Low risk	Quote: "Placebo pills…were similar in terms of shape and color to the supplements."Comment: probably done
Incomplete outcome data (attrition bias)	Low risk	Comment: 6 of 48 (12.5%) women were excluded from analysis. Reasons for exclusion were clearly identified.
Selective reporting (reporting bias)	Low risk	Comment: All outcomes presented in the methods section were reported in the paper.
Other bias	Low risk	Comment: No other bias was identified

Ashorn et al. 2015

**Table:** 

**Methods**	This was a randomised trial with 3 intervention groups conducted at 4 sites: a public district hospital (Mangochi), a semi private hospital (Malindi), and 2 public health centres (Lugwena and Namwera), in Mangochi District in southern Malawi. Dates of study: February 2011‐December 2015
**Participants**	Participants (n = 1391) were pregnant women who were <20 weeks of gestation confirmed by ultrasound, resided in the defined catchment area, were available during the study period, and signed or thumb‐printed an informed consent form.Women who were <15 years of age, needed frequent medical attention due to a chronic health condition, diagnosed with asthma treated with regular medication, had an illness warranting hospital referral, had a history of peanut allergy, anaphylaxis or serious allergic reaction to any substance, required emergency medical care, had pregnancy complications at enrolment (moderate‐severe oedema, bloodHb concentration <50 g/L, systolic BP >160 mmHg or diastolic BP >100 mmHg), participated in the iLiNS DYAD‐M trial during a previous pregnancy, or were concurrently participating in other clinical trials were excluded
**Interventions**	1. IFA group (n = 463; 391 were HIV‐ve) received a daily IFA tablet containing iron (60 mg) and folic acid (400ug) during pregnancy, and a tablet containing calcium (200 mg/day) during the first 6 months of lactation. No supplementation was given to infants born to these women. 2. MMN group (n = 466; 414 were HIV‐ve) received a daily MMN tablet during pregnancy and the first 6 months of lactation. The supplement consisted of vitamin A (800 ug retinol equivalents), vitamin B1 (2.8 mg), B2 (2.8 mg), B6 (3.8 mg), B12 (5.2ug), vitamin C (100 mg), vitamin D (400 IU), vitamin E (20 mg), vitamin K (45 ug), niacin (36 mg), folic acid (400 ug), pantothenic acid (7 mg), iron (20 mg), zinc (30 mg), copper (4 mg), selenium (130 ug), iodine (250 ug) and manganese (2.6 mg). No supplementation was given to infants born to these women.3. LNS group (n = 462; 395 were HIV‐ve) received 20 g LNS daily during pregnancy and the first 6 months of lactation. The supplement consisted of the same micronutrients as the MMN supplement, in addition to calcium, phosphorus, potassium, magnesium, energy (118 kcal/d) and macronutrients (protein and essential fatty acids). Infants born to these women received 20 g of LNS daily from 6‐18 months of age.
**Outcomes**	This study was incldued in two comparisons in this review:1) MMN vs. IFA: Outcomes analyzed were low birth weight, stillbirths, neonatal mortality, small for gestational age, maternal mortality, perinatal mortality, preterm births, maternal anaemia and miscarriage.2) LNS vs. MMN: Outcomes analyzed were low birth weight, stillbirths, neonatal mortality, small for gestational age, maternal mortality, perinatal mortality, preterm births, maternal anaemia and miscarriage.It should be noted that the data for small for gestational age were obtained from a separate report (Keats 2019) and not from the individual trial report.
**Notes**	All participants also received 2 doses of intermittent preventative malaria treatment with sulfadoxine‐pyrimethamine (3 tablets of 500 mg sulfadoxine and 25 mg pyrimethamine orally). 1 dose was given at enrolment and the other between 28 and 34weeks of gestation.In this review, the MMN group was used as the intervention group and the IFA group was used as the comparison group. HIV‐ve data included in this review was obtained from personal correspondence with trial investigators.**Declarations of interest:** One of the authors worked as a director of research for Nutriset S.A.S., a company that produces and sells lipid‐based nutrient supplements and also prepared the LNS supplements purchased for the current trial. The other authors declared no conflict of interest.**Funding sources:** supported in part by a grant from the Bill & Melinda Gates Foundation, with additional funding from the Office of Health, Infectious Diseases, and Nutrition, Bureau for Global Health, US Agency for International Development (USAID) under terms of Cooperative Agreement No. AID‐OAA‐A‐12‐00005, through the Food and Nutrition Technical Assistance III Project (FANTA), managed by FHI360. For data management and statistical analysis, the team received additional support in grants from the Academy of Finland (grant 252075) and the Medical Research Fund of Tampere University Hospital (grant 9M004). YBC was supported by the Singapore Ministry of Health's National Medical Research Council under its Clinician Scientist Award

Risk of bias table

**Table:** 

Bias	Authors' judgement	Support for judgement
Random sequence generation (selection bias)	Low risk	Quote: "A study statistician not involved in data collection generated 4 randomization code lists in blocks of 9 (one list for each of the 4 enrolment sites) Comment: probably done
Allocation concealment (selection bias)	Low risk	Quote: "a researcher not involved with the trial created individual randomisation slips (in blocks of 9) and packed them in sealed, numbered, opaque randomisation envelopes that were stored in numerical order. Eligible pregnant women were requested to choose 1 of the top 6 envelopes in the stack, and the contents of the envelope indicated her participant number and group allocation:Comment: probably done
Blinding of participants and personnel (performance bias)	Low risk	Quote: "The IFA and MMN interventions were provided by using double‐masked procedures that is the capsules looked identical, and neither the participants nor the research team members were aware of the nutrient contents of the supplement capsules."Comment: participants and caregivers were problably blinded to the treatment assignment
Blinding of outcome assessment (detection bias)	Low risk	Quote: "The data collectors who performed the anthropometric measurements or assessed other outcomes were not aware of group allocation. Rsearchers responsible for the data cleaning remained blind to the trial code until the database was fully cleaned."Comment: outcome assessors were probably blinded to the treatment assignment.
Incomplete outcome data (attrition bias)	Low risk	Attrition (until delivery) was 6.0% (and was balanced between treatment arms); reasons were not reported
Selective reporting (reporting bias)	Low risk	Comment: all outcomes presented in the methods section were reported in the paper
Other bias	Low risk	Comment: no other bias was identified

Belizán 1997

**Table:** 

**Methods**	This multi‐center double‐blind, randomized controlled trial was conductd in an urban setting in Rosario, Argentina Dates of study: January 1995 ‐ March 1996
**Participants**	1194 pregnant women who sought prenatal care before the 20th week of gestation between January 1987 and September 1989, were nulliparous, had singleton pregnancies, were less than 20 weeks pregnant at the time of the first interview, and had blood presssured below 140/90 mm Hg were eligible. Calcium group (n = 593), Placebo group (n = 601)
**Interventions**	1. Calcium group (n = 593): received 4 tablets containing 500 mg of elemental calcium and granulated starch, to be consumed daily for a total daily calcium supplementation of 2000 mg2. Placebo group (n = 601): received 4 tablets containing lactose and granulated starch, to be consumed daily.No attempts to alter dietary intake during the study were made.
**Outcomes**	Primary outcomes: Low birth weight (<2500 g).Secondary outcomes: Caesarean section, pre‐eclampsia and preterm birth and stillbirths
**Notes**	Protocol and methods were described in an earlier publication.Treatment compliance was assessed at each prenatal visit; at that time, the bottle that had been in use was collected and a new bottle was given to the woman. The tablets actually taken was divided by the number required. **Declarations of Interest:** none reported **Funding sources:** Supported by a grant (3‐P‐86‐0040) from the International Development Research Center in Canada.

Risk of bias table

**Table:** 

Bias	Authors' judgement	Support for judgement
Random sequence generation (selection bias)	Low risk	Quote: "They were randomized at each hospital by a random‐sample generator program provided by the Epistat Staistical Package." Comment: probably done
Allocation concealment (selection bias)	Low risk	Quote: "A complete set of numbered, sealed, opqaue envelopes containing the randomization codes was sent to each of the three hospitals. The series of bottles (one bottle for each scheduled prenatal visits) containing all the tablets needed for the entire pregnancy were kept at the central unit. When a woman was enrolled and the corresponding envelope was opened at the hospital, the study number was revealed to the central unit. Thus, the women, the nurses, and the physicians responsible for prenantal care were all unaware of the women's treatment status."Comment: probably done
Blinding of participants and personnel (performance bias)	Low risk	Quote: "When a woman was enrolled and the corresponding envelope was opened at the hospital, the study number was revealed to the central unit. Thus, the women, the nurses, and the physicians responsible for prenantal care were all unaware of the women's treatment status…The placebo tablets contained lactose and granulated starch and were identical to the calcium tablets with respect to weight, size, flavor, and color."Comment: probably done
Blinding of outcome assessment (detection bias)	Low risk	Quote: "When a woman was enrolled and the corresponding envelope was opened at the hospital, the study number was revealed to the central unit. Thus, the women, the nurses, and the physicians responsible for prenantal care were all unaware of the women's treatment status"Comment: probably done
Incomplete outcome data (attrition bias)	Low risk	Quote: "87.6% of the original population (86.2%, calcium group; 89.1%, placebo group) were available for follow up" and "They measured and compared data for those lost to followup, they carried out another per protocol analysis and other sensitivity analyses to see effect of attrition on final estimates. Incomplete data did not have significant effect on outcomes"Comment: attrition and exclusion were balanced across treatment arms
Selective reporting (reporting bias)	Low risk	Comment: All outcomes presented in the methods section were reported in the paper.
Other bias	Low risk	Comment: No other bias was identified

Bhutta 2009

**Table:** 

**Methods**	This cluster‐randomised trial was conducted in urban and rural areas in Pakistan Dates of study: not reported
**Participants**	Pregnant women with gestational age <16 weeks were eligible for enrolment. MMN group (n = 1148), IFA group (n = 1230)
**Interventions**	1. MMN group received vitamin A 800 mcg, D 200 IU, E 10 mg, C 70 mg, B1 1.4 mg, B2 1.4 mg, niacin 18 mg, B6 1.9 mg, B12 2.6 mg, folic acid 400 ug, iron 30 mg, zinc 15 mg, copper 2 mg, selenium 65 ug and iodine 150 ug.2. IFA group received 60 mg iron and 400 ug folic acid
**Outcomes**	Primary outcomes analyzed were low birth weight, maternal anaemia and perinatal mortalitySecondary outcomes analyzed were serum/plasma zinc (umol/L), serum/plasma retinol (umol/L), serum/plasma hemoglobin (g/L), serum/plasma ferritin (ug/L), congenital anomallies, miscarriage, preterm births, mode of delivery ‐ Caesarean section, small for gestational age, neonatal mortality and stillbirths.It should be noted that the data for SGA were obtained from a separate report (Keats 2019) and not from the individual trial report.
**Notes**	MMN and MMN + nutritional education groups were compared with IFA and IFA+ nutritional education group. IFA given to all participants. Maternal malnutrition, vitamin A deficiency, anaemia and iron deficiency were common. 2 methods of community outreach were implemented that is, basic nutrition along with antenatal care messages and quarterly community‐based group sessions conducted by CHWs and social scientist. There was no significant difference in baseline characteristics between 2 groups Data for caesarean section were presented (intervention 18/743; control 22/832). Data were not included in the analysis as this was a cluster‐RCT **Declarations of interest**: not reported **Funding sources:** United Nations Children's Fund (UNICEF)

Risk of bias table

**Table:** 

Bias	Authors' judgement	Support for judgement
Random sequence generation (selection bias)	Low risk	Quote: "a cluster‐based allocation strategy of supplements (either IFA or MMN supplementation) by respective CHWs was implemented" Comment: probably done
Allocation concealment (selection bias)	Low risk	Quote: "allocated to either the IFA or MMN supplements according to their respective location and allocation by the AKU Pharmacy"Comment: probably done
Blinding of participants and personnel (performance bias)	Low risk	Quote: "Both tables were identical in colour, shape and packaging" and "field staff (medical officers, CHWs, social scientists and data collection team) remained completely blinded as to the supplements allocation. All pregnant women were allocated a unique code and allocated a uniquely labelled and numerically coded specific supplement supply."Comment: participants and caregivers were probably blinded to the treatment assignment
Blinding of outcome assessment (detection bias)	Low risk	Quote: "Both tables were identical in colour, shape and packaging" and "field staff (medical officers, CHWs, social scientists and data collection team) remained copmletely blinded as to the supplements allocation"Comment: outcome assessors were probably blinded to the treatment assignment
Incomplete outcome data (attrition bias)	Low risk	Attrition (15.8%) and exclusion (around 1%) along with their reasons were reported. Attrition and exclusions were balanced across the treatment arms
Selective reporting (reporting bias)	Low risk	Comment: results of all outcomes mentioned in methods section were presented in the paper
Other bias	Low risk	Comment: no other bias was identified, including cluster‐design specific biases (recruitment bias, baseline imbalance, loss of clusters, incorrect analysis, and comparability with individually randomised trials)

Castillo‐Durán 2001

**Table:** 

**Methods**	This double‐blind randomized controlled trial was conducted in an urban setting in Santiago, Chile.
**Participants**	Pregnant adolescents from low socio‐economic status, and less than 20 weeks of gestation and aged <19 years at the estimated time of delivery were eligible. Exclusion critieria included chronic diseases, drug abuse, mental disabilities and delay, illiteracy or those with pregnancies due to incest or rape were not considered. Zinc group (n = 401), placebo group (n = 403)
**Interventions**	1. Zinc group: received capsules containing 20 mg of zinc sulphate to be consumed daily 2. Placebo group: received capsules containing lactose, to be consumed dailyAll women in both treatment arms received iron sulphate (40 mg) daily
**Outcomes**	Primary outcomes analyzed were low birth weight (<2500 g)Secondary outcomes analyzed were pre‐eclampsia, preterm births, serum/plasma zinc concentration (umol/L)
**Notes**	At the time of the study, Chile was considered a LMIC.Compliance with zinc intake was evaluated by counting the remaining capsules during the monthly visits. **Declarations of interest:** none reported **Funding Sources:** Partially funded by FONDECYT grant 068092

Risk of bias table

**Table:** 

Bias	Authors' judgement	Support for judgement
Random sequence generation (selection bias)	Unclear risk	Quote: "In a double blind fashion, subjects were randomly assigned to a Zinc‐Supplemented group (S) which received 20 mg Zn capsules daily (sulphate), or to a Placebo group (P), which received an equivalent capsule of a placebo containing lactose." Comment: insufficient information about the sequence generation process to permit judgment
Allocation concealment (selection bias)	Low risk	Quote: "To secure the double blind design, the group codes were changed twice during the study and were kept by the pharmacist who prepared the capsules until the end of computational analysis." Allocation codes and supplements were prepared by a source outside the study, and were kept hidden till the end of analysis"Comment: probably done
Blinding of participants and personnel (performance bias)	Low risk	Quote: "To secure the double blind design, the group codes were changed twice during the study and were kept by the pharmacist who prepared the capsules until the end of computational analysis."Comment: probably done
Blinding of outcome assessment (detection bias)	Low risk	Quote: "To secure the double blind design, the group codes were changed twice during the study and were kept by the pharmacist who prepared the capsules until the end of computational analysis."Comment: probably done
Incomplete outcome data (attrition bias)	High risk	Quote: "Eight hundred and four (804) pregnant adolescents were included in the study…297 were excluded during the follow‐up due to: failure to come to visits (n = 137), non‐compliant intake of zinc capsules (<15 capsules in any month) (n = 115), spontaneous abortion (n = 12), ultrasonographic assessment indicating that intervention began after the 20th week of gestation (n = 10), or revealing absence of pregnancy (n = 7), change of address (n = 6), apparent intolerance to zinc or placebo capsules (n = 6) and twin pregnancy (n = 4)Comment: attrition and exclusion were balanced across treatment arms
Selective reporting (reporting bias)	Low risk	Comment: All outcomes presented in the methods section were reported in the paper.
Other bias	Low risk	Comment: no other bias was identified

Caulfield 1999

**Table:** 

**Methods**	This double‐blind, randomized controlled trial as conducted in an urban shanytown, in Lima, Peru. Dates of study: 1995‐1997
**Participants**	Women with low‐risk pregnancy (uncomplicated and eligible for vaginal delivery), carrying a singleton fetus, and lived in coastal Peru for greater than or equal to 6 momnths before becoming pregnant were eligible. Signed consent was obtained from all participants. MMN group (n = 270) and Placebo group (n = 268)
**Interventions**	1. MMN group/Zinc group: received a tablet containing 60 mg of Iron, 250 ug of folic acid and 15 mg of zinc to be consumed daily2. IFA group/Placebo group: received a tablet containing 60 mg of iron and 250 ug of folic acid
**Outcomes**	This study was included in two comparisons in this review: MMN vs. IFA and Zinc vs. placebo1) MMN vs. IFA: the primary outcome measured was low birth weight (<2500 g). Secondary outomes (maternal) included: serum/plasma ferritin concentration (ug/L), serum/plasma folate (nmol/L), serum/plasma transferrin receptor (mg/L), serum/plasma hemoglobin (g/L), serum/plasma vitamin B12 (pmol/L), serum/plasma zinc concentration (umol/L). Secondary outcomes (child) included: preterm births, infection (fever), serum/plasma hemoglobin concentration (g/L)2) Zinc vs. placebo: the primary outcome measured was low birth weight, and a secondary outcome measured was preterm births
**Notes**	Compliance with supplementation was monitored monthly through the prenatal care distribution system and biweekly by health workers who interviewed women in their homes and observed the number of tablets remaining in each blisterpack. **Declarations of interest:** none reported **Funding Sources:** none reported

Risk of bias table

**Table:** 

Bias	Authors' judgement	Support for judgement
Random sequence generation (selection bias)	Unclear risk	Comment: insufficient information about the sequence generation process to permit judgment
Allocation concealment (selection bias)	Low risk	Quote: "Supplemented were contained in coded blister packs, and codes were unknown to investigators until data analysis was complete." Comment: probably done
Blinding of participants and personnel (performance bias)	Low risk	Quote: "Supplemented were contained in coded blister packs, and codes were unknown to investigators until data analysis was complete."Comment: probably done
Blinding of outcome assessment (detection bias)	Low risk	Quote: "double‐blind", "Supplemented were contained in coded blister packs, and codes were unknown to investigators until data analysis was complete."Comment: probably done
Incomplete outcome data (attrition bias)	High risk	Comment: Researchers changed blood collection methods, so only 538 of 1295 women enrolled in the study had measurements at 3 time points.
Selective reporting (reporting bias)	High risk	Quote: "Few of the final 200 women enrolled in the study were not included for analysis, since a subsample of 500 women was reached before the end of enrollment."
Other bias	Low risk	Comment: No other bias was identified

Charandabi 2015

**Table:** 

**Methods**	This was a randomized triple‐blind, controlled trial conducted in health centers in Tabriz, Iran. Dates of study: 2013‐2014
**Participants**	Pregnant women (n = 126) who were referred to Tabriz health centers, between 25‐30 weeks of gestation, and aged 18‐39 years old were eligible. Exclusion criteria included: history of chronic disease, kidney problems, osteomalacia, active thryoid and parathyroid diseases, endocrine diseases, hypertension, use of diuretics, calcium‐blockers intake, chronic hypertension, and history of allergy to the study supplements.The participants were randomized to three arms: calcium‐vitamin D arm (n = 40), vitamin D group (n = 42) and control group (n = 42). The authors noted that 85% and 96% of the participants consumed multivitamins and iron supplements, respectively.
**Interventions**	There were three arms of this study:(1) Calcium‐Vitamin D group: received 300 mg of carbonate calcium and 1000 IU of vitamin D (2) Vitamin D group: received 1000 IU of Vitamin D (3) Control group: received a placebo tablet in similar shape, size and weight as the tablets for the two intervention arms
**Outcomes**	This study was included in the vitamin D vs. placebo comparison. Secondary outcomes measured for this comparison were mode of delivery ‐ Caesarean section and preterm birth.While this study also measures calcium‐vitamin D vs. placebo, this comparison was excluded from the review due to insufficient number of studies per outcome.
**Notes**	**Declarations of Interest:** Authors declare no conflict of interest **Funding sources:** None reported

Risk of bias table

**Table:** 

Bias	Authors' judgement	Support for judgement
Random sequence generation (selection bias)	High risk	Quote: "Participants were assigned into three groups via block randomization with a block size of three and six with allocation ratio of 1:1:1." and "In the selected centers, the subjects were selected by convenience sampling method"
Allocation concealment (selection bias)	Low risk	Quote: "For allocation concealment, drugs and placebo were placed in the same closed opaque envelopes that serial numbered according to allocation sequence. Block scheduling and preparing envelopes were performed by a person not involved in the sampling and data analysis.
Blinding of participants and personnel (performance bias)	Low risk	Quote: "For allocation concealment, drugs and placebo were placed in the same closed opaque envelopes that serial numbered according to allocation sequence. Block scheduling and preparing envelopes were performed by a person not involved in the sampling and data analysis.
Blinding of outcome assessment (detection bias)	Low risk	Quote: "…provided tablets with similar shape, size, and weight. For uniformity of the weight of tablets an inert material was added to tablets of vitamin D group."
Incomplete outcome data (attrition bias)	Low risk	Quote: "The neonate anthropometric characteristics of 2 women in calcium‐vitamin D group were not available." Comment: Data for 124 of the enrolled 126 pregnant women were captured by the end of the study
Selective reporting (reporting bias)	Low risk	Comment: Outcome presented in the methods sections were reported in the paper or previous studies.
Other bias	Low risk	Comment: No other bias was identified

Choudhury 2012

**Table:** 

**Methods**	This was a cluster randomized controlled trial conducted in a densley populated rural setting in Kaliganj District, Bangladesh. This area relies primarily on agricultural labour. Dates of study: Not reported; dates of recruitment: October 2005‐March 2006
**Participants**	Pregnant women receiving care from 42 community‐based Antenatal Care Centres operated by the Bangladesh Rural Adevancement Committee (BRAC), and who were between 14 and 22 weeks of gestation were eligible. Exclusion criteria include: severe anaemia (hemoglobin <70 g/L), hemoglobin concentration greater than 140 g/L, were greater than 22 weeks of gestation, or were already taking iron supplements prior to the start of the study. MMN group (n = 243) and IFA group (n = 235)
**Interventions**	1. MMN group: received a micronutrient powder containing 60 mg of iron, 400 ug of folic acid, 30 mg of vitamin C and 5 mg of zinc, to be consumed daily2. IFA group: received a powder containing 60 mg of iron and 400ug of folic acid, to be consumed daily
**Outcomes**	Primary outcomes: maternal anaemiaSecondary outcomes: maternal serum/plasma hemoglobin (g/L)
**Notes**	Fieldworkers assessed adherence by recording the number of empty and full MNP‐P sachets remaining or the number of unused IFA tablets and asking specific questions about the sharing and general use of the supplements. Adherence was defined as the ratio of the number of tablets or sachets consumed to the total recommended during a specific period of time. At each visit, the fieldworkers also provided educational support with the use of a flip chart to maximize adherence to the interventions. **Declarations of interest:** None reported **Funding sources:** grant from the HJ Heinz Company Foundation

Risk of bias table

**Table:** 

Bias	Authors' judgement	Support for judgement
Random sequence generation (selection bias)	Unclear risk	Quote: "each cluster was randomly allocated to receive either MNP‐P…or IFA tablets" Comment: Process of random sequence generation was not clear
Allocation concealment (selection bias)	Low risk	Quote: "Out of this population, 478 women met the eligibility criteria and were randomized, at the cluster level…"Comment: probably done
Blinding of participants and personnel (performance bias)	Unclear risk	Comment: IFA and MMN supplements were not identical in appearance. The former was in tablet form, while the latter was in powder form.
Blinding of outcome assessment (detection bias)	Unclear risk	Comment: blinding of outcome was not discussed
Incomplete outcome data (attrition bias)	Low risk	Quote: "…the loss to follow‐up at 32 weeks was similar between groups (14.8% vs 15.7% in the MNP‐P and IFA groups, respectively)"
Selective reporting (reporting bias)	Low risk	Comment: Outcome presented in the methods sections were reported in the paper or previous studies.
Other bias	Low risk	Comment: No other bias was identified

Christian 2003

**Table:** 

**Methods**	This was a double‐blind cluster‐randomised trial, carried out in rural Nepal Dates of study: December 1998‐April 2001
**Participants**	A total of 4926 pregnant women were enrolled in the study. The women were randomised into 5 groups as follows: group 1 (n = 941), group 2 (n = 957), group 3 (n = 999), group 4 (n = 1050) and group 5 (n = 1051)Women who were currently pregnant or those who were breastfeeding an infant <9 months old were excluded from the study. Also excluded were menopausal, sterilised or widowed women
**Interventions**	This study had five treatment arms, as described below:Group 1: received folic acid (400 ug) dailyGroup 2: received iron‐folic acid (60 mg of iron and 400 ug of folic acid) dailyGroup 3: received iron‐folic acid and zinc (60 mg of iron, 400 ug of folic acid and 30 mg of zinc sulphate) dailyGroup 4: received multiple micronutrients: iron‐folic acid and zinc, plus 10 ug of Vitamin D, 10 mg of Vitamin E, 1.6 mg of Vitamin B1, 1.8 mg of Vitamin B2, 20 mg of niacin, 2.2 mg of vitamin B6, 2.6ug of Vitamin B12, 100 mg of Vitamin C, 65 ug of vitamin K, 2.0 mg of copper and 100 mg of magnesium, daily.Groups 1‐4 all received 1000 ug retinol equivalents of vitamin A.Group 5 (Control group): received Vitamin A only (1000 ug retinol equivalents) dailyAll supplements were given orally from the time of pregnancy detection until 12 weeks after a live birth or 5 weeks after a still birth or a miscarriageThis study was included in four comparisons in this review: MMN vs. IFA (comparing Group 2 and 4), Zinc vs. Placebo (comparing Group 2 and Group 3), IFA vs FA (comparing Group 1 and Group 2), and Iron vs placebo (Group 1 and Group 2)
**Outcomes**	For the IFA vs. FA comparison and Iron vs. placebo comparison, the primary outcomes included: perinatal mortality, low birth weight, maternal anaemia. The secondary outcomes included preterm birth and neonatal mortality and serum/plasma hemoglobin (g/L).For the MMN vs. IFA comparison, the primary outcomes included: maternal iron‐deficiency anaemia, low birth weight, maternal anaemia, and perinatal mortality. Secondary outcomes: small‐for‐gestational age, diarrhea, serum/plasma transferrin receptor, developmental outcomes in children, neonatal mortality, mode of delivery, congenital anomalies, preterm births, stillbirths, serum/plasma ferritin, and serum/plasma hemoglobin (g/L).For Zinc vs. placebo comparison, the primary outcomes included: low birth weight. Secondary outcomes included: preterm birth and small for gestational ageIt should be noted that the data for SGA were obtained from a separate report (Keats 2019) and not from the individual trial report.
**Notes**	All women were offered 2 x 400 mg single‐dose albendazole in the second and third trimester of pregnancy because of the high prevalence of hookworm infestation in this population. Hookworm infestation and vitamin A deficiency are one of the major causes of anaemia in this population. Due to this reason, vitamin A was given to all the participants including the control groupBaseline characteristics did not differ significantly among the various randomisation groups except for ethnicity and land holdingIn this review, we used the group 4 data for the MMN group and group 2 data for the control group. All the estimates were adjusted for the cluster design and provided by the trial authors**Declarations of interest**: the trial authors declared no conflict of interest.**Funding sources:** US Agency for International Development (USAID) and additional support from the Unicef Country Office, Kathmandu, Nepal, and the Bill and Melinda Gates Foundation

Risk of bias table

**Table:** 

Bias	Authors' judgement	Support for judgement
Random sequence generation (selection bias)	Low risk	Quote: "Randomisation was done in blocks of five within each village development community by the senior study investigators, who drew numbered chips from a hat" Comment: probably done
Allocation concealment (selection bias)	Unclear risk	Quote: "Randomisation was done in blocks of five within each village development community by the senior study investigators, who drew numbered chips from a hat"Comment: probably done
Blinding of participants and personnel (performance bias)	Low risk	Quote: "Participants, investigators, field staff and statisticians did not know supplement codes" and "supplements, which were of identical shape, size and color" and "code allocation was kept locked at the Johns Hopkins University, Baltimore"Comment: participants and caregivers were blinded to the treatment assignment
Blinding of outcome assessment (detection bias)	Low risk	Quote: "Participants, investigators, field staff and statisticians did not know supplement codes"Comment: participants and caregivers were blinded to the treatment assignment
Incomplete outcome data (attrition bias)	Low risk	Exclusion (1.43%) and attrition (6.9%) were reported along with their reasons
Selective reporting (reporting bias)	Low risk	Comment: results of all outcomes mentioned in methods were presented in the various publications of this trial
Other bias	Low risk	Comment: no other bias was identified, including cluster‐design specific biases (recruitment bias, baseline imbalance, loss of clusters, incorrect analysis, and comparability with individually randomised trials)

Cox 2005

**Table:** 

**Methods**	This was a randomized double‐blind controlled trial conducted in antenatal clinics at Nkoranza District Hospital and three rural health clinics in Brong Ahafo region, Central Ghana. Dates of study: not reported; Dates of recruitment: March to June 2001
**Participants**	Pregnant, primigravid women (n = 98) were eligible if they resided within the study area, in good health, and <24 weeks of gestation. The authors noted that malaria transmission is perennial and intense in this study area. Exclusion criteria included HIV and tuberculosis infections. HIV infection status and tuberculousis infection status were based on available health records; but no diagnostics tests were performed. The authors note that the estimated prevalence of HIV infection amongst antentatal attendees was 1.6% in 2000 in the Brong Ahafo region, according to the MInistry of Health.Vitamin A group (n = 48), Placebo group (n = 50)
**Interventions**	Vitamin A group (n = 48): received capsules that contained 10,000 IU of vitamin A (as retinyl palmitate in groundnut oil) plus tocopherol as a preservative. to be consumed weeklyPlacebo group (n = 50): received capsules that contained groundnut oil (without Vitamin A) and tocopherol, to be consumed weekly.
**Outcomes**	Secondary outcomes: maternal serum/plasma hemoglobin (g/L)
**Notes**	**Declarations of interest:** Not reported **Funding Sources:** MRC UK (G78/6363), University of London Central Research Fund, Nestle Foundation, Commission of the European Communities (QLK2‐CT‐2001‐01302, PAMVAC), Danish Medical Research Council (SSVF 22‐02‐0571), Denis Burkit Study Award, Jeremy Chadwick Travelling Fellowship, Parkes Foundation.

Risk of bias table

**Table:** 

Bias	Authors' judgement	Support for judgement
Random sequence generation (selection bias)	Low risk	Quote: "Using balanced block randomization 100 women were randomized (eight women per block) to one of eight sets of capsules, four of which contained vitamin A."
Allocation concealment (selection bias)	Low risk	Quote: "The coding was unknown to the investigators, until after completion of the trial and the capsules were identical in appearance."
Blinding of participants and personnel (performance bias)	Low risk	Quote: "The coding was unknown to the investigators, until after completion of the trial and the capsules were identical in appearance."
Blinding of outcome assessment (detection bias)	Low risk	Quote: "capsules were identical in appearance."
Incomplete outcome data (attrition bias)	Low risk	Comment: Only 12 (12%) women were excluded from the analysis
Selective reporting (reporting bias)	Low risk	Comment: results of all outcomes mentioned in methods section were presented in the paper
Other bias	Low risk	Comment: no other bias was identified

Darling et al. 2017

**Table:** 

**Methods**	This was a double‐blind placebo‐controlled, randomized trial conducted in 8 antenatal care clinics in the urban Temeke and Ilala districts of Dar es Salaam, Tanzania. Dates of study: July 12, 2010 to September 17, 2013. Follow‐up was completed in June 2014.
**Participants**	Participants (n = 2500) were pregnant women in their first trimester of pregnancy, primigravida or secundigravida. Eligible participants intended to stay in Dar es Salaam for at least 6 weeks after delivery, and were HIV‐negative.
**Interventions**	Individuals were randomized into the following arms:1) Vitamin A only group: 2,500 IU of vitamin A daily2) Zinc only group: 25 mg of zinc (as zinc sulfate) daily3) Combination: both 2,500 IU of vitamin A and 25 mg of zinc (as zinc sulfate) daily4) Placebo: received placebo tablets and instructed to take the regimen orally each day until deliveryAccording to standard of care, participants were also given 60 mg of iron (daily) and 5 mg of folic acid (daily), and 1500 mg of sulfadoxine and 75 mg of pyrimethamine as pregnancy malaria prophylaxis.
**Outcomes**	This study was included in two comparisons:1) Vitamin A vs placebo: Secondary outcomes analyzed were maternal serum/plasma hemoglobin (g/L)2) Zinc vs placebo: Secondary outcomes analyzed were maternal serum/plasma zinc (umol/L)Note: this study should have also been included in the MMN vs IFA comparison (using data from arm 3); however, the data for this combined group was not disaggregated. Authors were contacted requesting their data, but have not yet provided their data.
**Notes**	**Declarations of interes**t: Not reported**Funding sources**: grants from the National Institute of Child Health and Human Development (NICHD R01 HD057941‐01 and K24 HD 058795 [Christopher Duggan]).

Risk of bias table

**Table:** 

Bias	Authors' judgement	Support for judgement
Random sequence generation (selection bias)	Low risk	Quote: "Allocation to treatment groups was performed according to a computer‐generated randomization sequence using blocks of size 20 by a scientist not involved in the data collection." Comment: probably done
Allocation concealment (selection bias)	Low risk	Quote: "Allocation to treatment groups was performed according to a computer‐generated randomization sequence using blocks of size 20 by a scientist not involved in the data collection."Comment: probably done
Blinding of participants and personnel (performance bias)	Low risk	Quote: "Neither the participants nor the study personnel had access to the masking information"Comment: probably done
Blinding of outcome assessment (detection bias)	Low risk	Quote: "Each study clinic was issued regimen bottles that were prelabeled according to this sequence by study pharmacists who had no contact with participants. At enrollment, each participant was assigned to the next numbered bottle at their site. The study remained blinded until all trial assessments and database cleaning were completed, at which time study staff analyzing the data were given access to treatment assignments."Comment: probably done
Incomplete outcome data (attrition bias)	High risk	Comment: >20% loss to follow‐up. Reasons for loss to follow‐up were reported.
Selective reporting (reporting bias)	High risk	Comment: results for Vitamin A supplementation were combined with the results from the Vitamin A‐Zinc combined arm. Similarly, the results for zinc supplementation were combined with the results ffrom the Vitamin A‐Zinc combined arm. This prevented this review from reporting disaggregated results for the vitamin A only group, zinc only group and the vitamin A and zinc combined group.
Other bias	Low risk	Comment: no other bias was identified

Dewey 2009

**Table:** 

**Methods**	This was a randomised study with 3 intervention groups conducted in the Somanya‐ Kpong area of the Yilo Krobo and Lower Manya Krobo districts in southern Ghana Date of study: December 2009‐March 2014
**Participants**	Participants (n = 1320) were pregnant women who were greater than or equal to 18 years of age and <20 weeks of gestation confirmed by ultrasound. Women who were not residing in the target area, who intended to move within the next 2 years, had a known milk or peanut allergy, were unwilling to receive field workers or take the study supplement, were participating in another study, or had an antenatal card indicating HIV infection, asthma, epilepsy, tuberculosis, or any malignancy were excluded
**Interventions**	1. IFA group (n = 441) received a daily IFA tablet containing iron (60 mg) and folic acid (400 ug) during pregnancy, and a tablet containing calcium (200 mg/day) during the first 6 months of lactation. No supplementation was given to infants born to these women.2. MMN group (n = 439) received a daily MMN tablet during pregnancy and the first 6 months of lactation. The supplement consisted of vitamin A (800 ug), vitamin B1 (2.8 mg), B2 (2.8 mg), B6 (3.8 mg), B12 (5.2 ug), vitamin C (100 mg), vitamin D (400 IU), vitamin E (20 mg), vitamin K (45 ug), niacin (36 mg), folic acid (400 ug), panthothenic acid (7 mg), iron (20 mg), zinc (30 mg), copper (4 mg), selenium (130 ug), iodine (250 ug) and manganese (2.6 mg). No supplementation was given to infants born to these women.3. LNS group (n = 440) received 20 g LNS daily during pregnancy and the first 6 months of lactation. The supplement consisted of the same micronutrients as the MMN supplement, in addition to calcium, phosphorus, potassium, magnesium, energy (118 kcal/d) and macronutrients (protein and essential fatty acids). Infants born to these women received 20 g of LNS daily from 6‐18 months of age.
**Outcomes**	This study was included in two comparisons in this review: MMN vs. IFA and LNS vs. MMN (1) MMN vs. IFA: primary outcomes analyzed were low birth weight, perinatal mortality, maternal mortality, maternal anaemia and maternal iron‐deficiency anaemia. Secondary outcomes measured were serum/plasma hemoglobin (g/L), stillbirths, neonatal mortality, small for gestational age, serum/plasma transferrin receptor (mg/L), preterm births, congenital anomalies and miscarriage.(2) LNS vs. MMN: primary outcomes analyzed were low birth weight and perinatal mortality. Secondary outcomes analyzed were: miscarriage, small for gestational age, preterm births, neonatal mortality and stillbirths
**Notes**	Temporary mislabelling of IFA and MMN capsules resulted in some women in the IFA (n = 92) and MMN (n = 85) groups receiving both IFA and MMN supplements during pregnancy. These women were excluded in the analysis of pregnancy outcomes. **Declarations of interest:** MZ was an employee of Nutriset S.A.S., which is a commercial producer of LNS products. The other study authors declared no conflict of interest **Funding sources**: Bill & Melinda Gates Foundation

Risk of bias table

**Table:** 

Bias	Authors' judgement	Support for judgement
Random sequence generation (selection bias)	Low risk	Quote: "women were randomly allocated into one of 3 groups by using a computer generated scheme (SAS version 9.3; SAS Institute) in blocks of 9." Comment: probably done
Allocation concealment (selection bias)	Low risk	Quote: "Sheets bearing supplement allocations represented by 6 different color codes (3 for IFA and 3 for MMN) and an inscription "LNS" for the LNS group, and numbered 1 to 1320 were placed in opaque envelopes and stacked in increasing order. At each enrolment, the study nurse shuffled the 9 topmost envelopes in the stack, and the woman picked one to reveal allocation. Allocation information was kept by the field supervisor (HO) in a passwordprotected file, which was shared with the study statistician (JMP) at UC Davis, who designed the randomisation scheme."Comment: probably done
Blinding of participants and personnel (performance bias)	Low risk	Quote: "Two individuals in Ghana who were independent of the research team colour‐coded the capsules by placing colour stickers (which also included the letter P or L to indicate pregnancy or lactation) on the blister packs of IFA and MMN, so that no investigator, study worker or participant knew the identities of the capsules except by the colours."Comment: participants and caregivers were probably blinded to the treatment assignment
Blinding of outcome assessment (detection bias)	Low risk	Quote: "none of the maternal or newborn anthropometrists were aware of the code allocations. Likewise, data analysts remained blinded until all preliminary analyses had been completed, and the allocation codes were broken."
Incomplete outcome data (attrition bias)	Low risk	Exclusion (until delivery) was 20% (and was balanced between treatment arms); the reason was reported. Attrition (until delivery) was 4.4% and reasons were reported
Selective reporting (reporting bias)	Low risk	Comment: results of all outcomes mentioned in methods section were presented in the paper
Other bias	Low risk	Comment: no other bias was identified

Dijkhuizen 2001

**Table:** 

**Methods**	This was a randomized controlled trial condcuted in rural setting in Bogor District, West Java in Indonesia Dates of study: July 1998 ‐ January 1999
**Participants**	Pregnant women (n = 229) <20 weeks of gestation were eligible.
**Interventions**	1. MMN group: received 30 mg of zinc, 30 mg of iron as ferrous fumarate and 400ug of folic acid, daily2. IFA group: received 30 mg iron as ferrous fumarate and 400ug of folic acid, daily
**Outcomes**	This study is included in two comparisons in this review: MMN vs. IFA and Zinc vs. Placebo(1) MMN vs. IFA: primary outcomes analyzed were perinatal mortality and low birth weight. Secondary outcomes analyzed were: mode of delivery (Caesarean section), serum/plasma retinol (umol/L), serum/plasma zinc (umol/L), serum/plasma ferritin (ug/L), serum/plasma hemoglobin (g/L), congenital anomalies, preterm births, child serum/plasma retinol (umol/L), child serum/plasma zinc (umol/L) and child serum/plasma hemoglobin (g/L)(2) Zinc vs. Placebo: primary outcome analyzed was low birth weight. Secondary outcomes analyzed were serum/plasma zinc (umol/L) and preterm birthsIt should be noted that the data for small for gestational age were obtained from a separate report (Keats 2019) and not from the individual trial report.
**Notes**	**Declarations of interest:** authors declared no conflicts of interest **Funding sources:** none reported

Risk of bias table

**Table:** 

Bias	Authors' judgement	Support for judgement
Random sequence generation (selection bias)	Unclear risk	Comment: Authors indicated that women were randomly assigned to a group.
Allocation concealment (selection bias)	Low risk	Quote: "The code allocation was not known to the researchers until all observations and biochemical analyses were completed." Comment: probably done
Blinding of participants and personnel (performance bias)	Low risk	Comment: Double‐blind study design. Bottles were labled with only subject's name, number and date.
Blinding of outcome assessment (detection bias)	Low risk	Quote: "Capsules were prepared containing the micronutrients specific for each group and were indistinguishable from each other"Comment: probably done
Incomplete outcome data (attrition bias)	High risk	Comment: ~27% of the control group did not complete the study, while ~17% of the zinc group did not complete the trial.
Selective reporting (reporting bias)	Low risk	Comment: All outcomes presented in the methods section were reported in the paper.
Other bias	Low risk	Comment: No other bias was identified

Diogenes et al. 2013

**Table:** 

**Methods**	This was a randomized, single‐blind, placebo‐controlled trial conducted in an urban setting in Rio de Janeiro, Brazil Dates of study: undisclosed, dates of recruitment: September 2009 to June 2011
**Participants**	First time pregnant adolescents (n = 84), carrying a single fetus, and between 21‐29 weeks gestation, and planning to breastfeed were eligible. Exclusion criteria included chronic health problems, pregnancy complications, smokers, users of nutritional supplements besides iron and folate supplements provided during standard prenatal care, and mothers who decided not to breastfeed.Calcium + Vitamin D group (n = 43); placebo group (n = 41)
**Interventions**	1. Calcium + Vitamin D intervention group: received 600 mg calcium carbonate and 200 IU of Vitamin D, to be consumed daily2. Placebo group: received microcrystalline cellulose and corn starchWomen in both treatment arms received IFA provided during standard prenatal care
**Outcomes**	None. This comparison was removed from analysis as there were insufficient number of studies per outcome
**Notes**	**Declarations of Interest**: None of the authors had a conflict of interest **Funding Sources**: Conselho Nacional de Desenvolvimento Científico e Tecnológico [grant 471872/2008‐3] and Fundação Carlos Chagas Filho de Amparo à Pesquisa do Estado do Rio de Janeiro (grant E‐26/102.759/2008]

Risk of bias table

**Table:** 

Bias	Authors' judgement	Support for judgement
Random sequence generation (selection bias)	Low risk	Quote: "random assignment was performed by a member of the research team in a 1:1 ratio within permuted blocks of size 10" Comment: probably done
Allocation concealment (selection bias)	Unclear risk	Comment: Allocation concealment not discussed.
Blinding of participants and personnel (performance bias)	High risk	Quote: "Participants were randomly and single‐blinded assigned"
Blinding of outcome assessment (detection bias)	High risk	Quote: "Participants were randomly and single‐blinded assigned"
Incomplete outcome data (attrition bias)	High risk	Quote: "33% of enrolled participants were not analyzed at 5 wk postpartum, and ~ 44% were not analyzed at 20wk postpartum"
Selective reporting (reporting bias)	Low risk	Comment: All outcomes presented in the methods section were reported in the paper.
Other bias	Low risk	Comment: No other bias was identified

Duggan et al. 2014

**Table:** 

**Methods**	This was a randomised, double‐blind placebo‐controlled trial conducted in an urban setting of Bengaluru, India.
**Participants**	Pregnant women (n = 366), 18 years or older who presented for prenatal care before or at 14 weeks of gestational age (as judged by the date of the last menstrual period) were eligible for inclusion. Exclusion criteria included: mothers who anticipated moving out of the area before study completion, twin or multiple pregnancies, treated for infertility, tested positive for hepatitis B, HIV or syphilis infections, who were taking daily vitamin supplements in addition to the standard IFA, chronic health issues, or history of previous Caesarean section. Vitamin B12 group (n = 183); Placebo group (n = 183)
**Interventions**	1. Vitamin B12 intervention group: received 50 ug of Vitamin B12 to be taken daily2. Placebo group: received a placebo that was identical in appearance, taste and smell.As part of standard prenatal care, all mothers were given IFA (60 mg of iron and 500 ug of folic acid), diagnosis and treatment for sexually transmitted infections and prophylaxis
**Outcomes**	None analyzed. Excluded from analysis due to insufficient number of studies for the Vitam B12 vs. Placebo comparison.
**Notes**	**Declarations of Interest**: Authors report no conflict of interest **Funding Sources**: Indian Council of Medical Research grant 5/7/192/06‐RHN and Eunice Kennedy Shriver National Institute of Child Health and Human Development grants R03 HD054123 and K24HD058795

Risk of bias table

**Table:** 

Bias	Authors' judgement	Support for judgement
Random sequence generation (selection bias)	Low risk	Quote: "A randomization list from 1 to 370 was prepared by the study biostatistician using permuted blocks of variable size, and women enrolled at the study clinic were provided the next consecutive number on the list." Comment: probably done
Allocation concealment (selection bias)	Low risk	Quote: "The randomization list was provided to the pharmacy department in Bangalore, with each number corresponding to a code denoting 1 of the 2 treatment groups. Onsite study pharmacists stored the coded randomization list in a locked file cabinet and concealed allocation by only displaying the womans identification number on the label of the bottle."Comment: probably done
Blinding of participants and personnel (performance bias)	Low risk	Quote: "The placebo and vitamin B‐12 supplement were indistinguishable in terms of taste, smell, and appearance. Study physicians, research nurses, and participants were unaware of treatment groups."Comment: probably done
Blinding of outcome assessment (detection bias)	Low risk	Quote: "The placebo and vitamin B‐12 supplement were indistinguishable in terms of taste, smell, and appearance. Study physicians, research nurses, and participants were unaware of treatment groups."Comment: probably done
Incomplete outcome data (attrition bias)	High risk	Comment: 366 pregnant women were randomly assigned to a treatment group. Serum samples were collected from 284 subjects during the third trimester
Selective reporting (reporting bias)	Low risk	Comment: All outcomes presented in the methods section were reported in the paper.
Other bias	Low risk	Comment: No other bias was identified

Etheredge 2015

**Table:** 

**Methods**	This was a randomized double‐blind, placebo‐controlled trial conducted in an urban setting of Dar es Salaam in Tanzania. Dates of study: unclear, Dates of recruitment and screening: September 2010 to October 2012.
**Participants**	Pregnant women (n = 1500) who were not iron‐deficient (serum ferritin >12 ug/L), not severely anemic (hemoglobin >8.5 g/dL), primigravidae or secundigravidae, without HIV, and at or before 27 weeks gestation, and who intended to stary in Dar es Salaam until 6‐weeks post partum were eligible. Women who were excluded were provided with iron supplementation or other treatment per Tanzanian standards of care.
**Interventions**	1. Iron intervention group: received 60 mg of ferrous sulfate to be consumed daily2. placebo group: received placebo pill to be consumed dailyAll participants were provided with standard prenatal care, and received 5 mg/d of folic acid and intermittent preventive treatment of malaria during pregnancy using 1500 mg of sulfadoxine and 75 mg of pyrimethamine in the second and third trimesters.
**Outcomes**	This study was included in two comparisons in this review: IFA vs. FA and Iron vs. Placebo (1) IFA vs FA: Primary outcomes analyzed were maternal anaemia and low birth weight. Secondary outcomes analyzed were preterm births, small for gestational age, serum/plasma hemoglobin (g/L), infant mortality and serum/plasma ferritin (ug/L)(2) Iron vs. Placebo: Primary outcomes analyzed were maternal anaemia and low birth weight. Secondary outcomes analyzed were preterm births, small for gestational age, serum/plasma hemoglobin (g/L), infant mortality and serum/plasma ferritin (ug/L)
**Notes**	**Declarations of Interest:** Authors report no conflict of interest **Funding Sources:** This study was supported by grants U01 HD061232 (Dr Fawzi) and 1K24HD058795 (Dr Duggan) from the Eunice Kennedy Shriver National Institute of Child Health and Human Development

Risk of bias table

**Table:** 

Bias	Authors' judgement	Support for judgement
Random sequence generation (selection bias)	Low risk	Quote: "Participants were individually randomized. A computer‐generated randomization sequence in blocks of 20 was created by a scientist not involved in data collection." Comment: probably done
Allocation concealment (selection bias)	Low risk	Qutoe: "A computer‐generated randomization sequence in blocks of 20 was created by a scientist not involved in data collection"…" Study clinics were issued prelabeled regimen bottles, and at enrollment, a participant was assigned to the next numbered bottle in the regimen"Comment: probably done
Blinding of participants and personnel (performance bias)	Low risk	Quote: "The active and placebo tablets and packaging were indistinguishable. A computer‐generated randomization sequence in blocks of 20 was created by a scientist not involved in data collection. All participants and study personnel were masked until analyses were completed."
Blinding of outcome assessment (detection bias)	Low risk	Quote: "The active and placebo tablets and packaging were indistinguishable. All participants and study personnel were masked until analyses were completed."
Incomplete outcome data (attrition bias)	Low risk	Quote: "…for primary outcomes, although birth outcomes were obtained for 1469 of 1500 births (97.9%) and birth weights were obtained for 1363 of 1368 live birth deliveries (99.6%) (Figure), we were not always able to gather samples from mothers at delivery. Of 1469 women with delivery outcomes, it was not possible to collect placentas from 342 women who had early fetal loss (n = 59), delivered at a nonstudy hospital (n = 167), delivered outside Dar es Salaam (n = 98), or withdrew from the study (n = 18). Of the remaining 1127 women, 1003 placenta samples (89.0%) were obtained.for primary outcomes, although birth outcomes were obtained for 1469 of 1500 births (97.9%) and birth weights were obtained for 1363 of 1368 live birth deliveries (99.6%) (Figure), we were not always able to gather samples from mothers at delivery. Of 1469 women with delivery outcomes, it was not possible to collect placentas from 342 women who had early fetal loss (n = 59), delivered at a nonstudy hospital (n = 167), delivered outside Dar es Salaam (n = 98), or withdrew from the study (n = 18). Of the remaining 1127 women, 1003 placenta samples (89.0%) were obtained."
Selective reporting (reporting bias)	Low risk	Comment: All outcomes presented in the methods section were reported in the paper.
Other bias	Low risk	No other bias was identified

Falahi (2010)

**Table:** 

**Methods**	This was a randomized "triple‐blind" clinical trial conducted in Khorramabad City, Lorestan Province, in western Iran. Dates of study: not reported
**Participants**	Pregnant women (n = 148) who were nonanemic (hemoglobin concentration >110 g/L, serrum ferritin >12ug/L), <20 weeks of gestation, primigravidae, ages 20 and 35 years with a body mass index of >25 and <30 were included. Exclusion criteria included diabetes mellitus, coronary heart disease, thalessemia, renal disease, respiratory disease, use of supplementary multivitamins or minerals, drug use and those who were on a special diet.Iron group (n = 70) and placebo group (n = 78)
**Interventions**	The iron group (n = 70) received tablets containing 60 mg of iron.The placebo group (n = 78) received placebo tablets
**Outcomes**	Primary outcomes analyzed were maternal iron‐deficiency anaemiaSecondary outcomes analyzed were iron deficiency (ferritin), serum/plasma hemoglobin (g/L) and serum/plasma ferritin (ug/L)
**Notes**	During screening, women who were anemic or iron‐deficient were referred for medical evaluation and treatment.**Declarations of interest**: none reported**Funding sources**: none reported

Risk of bias table

**Table:** 

Bias	Authors' judgement	Support for judgement
Random sequence generation (selection bias)	Unclear risk	Comment: insufficient information to provide judgement
Allocation concealment (selection bias)	Unclear risk	Comment: insufficient information to provide judgement
Blinding of participants and personnel (performance bias)	Low risk	Quote: "tripe‐blind"…"The physician, laboratory analysts, and subjects were not aware of the type of tablet." Comment: probably done
Blinding of outcome assessment (detection bias)	Low risk	Quote: "The placebos were formulated to be indistinguishable from the tablets containing iron."Comment: probably done
Incomplete outcome data (attrition bias)	Unclear risk	Comment: insufficient information to provide judgement
Selective reporting (reporting bias)	Low risk	Comment: All outcomes presented in the methods section were reported in the paper.
Other bias	Low risk	No other bias was identified

Fawzi et al. 2007

**Table:** 

**Methods**	This was a double‐blind trial in Dar es Salam, Tanzania. Pregnant women who attended antenatal clinics were included Dates of study: August 2001‐July 2004
**Participants**	Pregnant women who attended antenatal clinics, had a negative test for HIV infection, planned to stay in the city until delivery and for 1 year thereafter with gestational age between 12 and 27 weeks according to LMP were included. The study groups were similar with respect to baseline characteristics
**Interventions**	1. MMN group (n = 4214) received vitamin B1 20 mg, B2 20 mg, B6 25 mg, B12 50 ug, C 500 mg, E 30 mg, niacin 100 mg, folic acid 0.8 mg2. IFA group (n = 4214) received placeboAll women irrespective of group received daily iron (60 mg) and folic acid (0.25 mg). Women were randomly assigned to receive either MM or control from the time of enrolment until 6 weeks after delivery
**Outcomes**	Primary outcomes analyzed were perinatal mortality and low birth weightSecondary outcomes analyzed were preterm birth, neonatal mortality, small‐for‐gestational age, serum/plasma hemoglobin (g/L), mode of delivery ‐ Caesarean section, miscarriage and stillbirthsIt should be noted that the data for small for gestational age were obtained from a separate report (Keats 2019) and not from the individual trial report.
**Notes**	Malaria prophylaxis (sulphadoxine‐pyrimethamine tablets) at 20 and 30 weeks of gestation was given to all. The study groups were similar with respect to baseline characteristics **Declarations of interest**: the trial authors declared no conflict of interest. **Funding sources**: National Institute of Child Health and Human Development (NICHD R01 37701)

Risk of bias table

**Table:** 

Bias	Authors' judgement	Support for judgement
Random sequence generation (selection bias)	Low risk	Quote: "A list was prepared according to a randomisation sequence in blocks of 20; at enrolment, each eligible women was assigned to the next numbered bottle and computerised random number generator was used (*personal communication)" Comment: probably done
Allocation concealment (selection bias)	Low risk	Quote: "Each eligible women was assigned to the next numbered bottle:Comment: Probably done
Blinding of participants and personnel (performance bias)	Low risk	Quote: "Active tablets and placebo were similar in shape, size and color and were packaged in identical coded bottles" and "Each eligible women was assigned to the next numbered bottle"Comment: participants and caregivers were blinded to the treatment assignment
Blinding of outcome assessment (detection bias)	Low risk	Quote: "research assistants who assessed the study outcome were unaware of the intervention group"Comment: outcome assessors were blinded
Incomplete outcome data (attrition bias)	Low risk	Exclusion (0.5%) and attrition (5.4%) were reported with reasons in each arm
Selective reporting (reporting bias)	Low risk	Comment: all outcomes mentioned in the methods section were presented in the paper
Other bias	Low risk	Comment: no other bias was identified.

Friis 2004

**Table:** 

**Methods**	This trial was carried out in Zimbabwe. Dates of study: 1996‐1997
**Participants**	Pregnant women who were between 22 and 36 weeks of gestation were eligible for enrolment. Participants (n =1669) were randomised into 2 groups, MMN group n = 837 and placebo n = 832. Out of the 1106 women that were followed, 725 were HIV‐ve and 360 were HIV+ve
**Interventions**	1. MMN group received daily supplementation of vitamin A 3000 mcg retinol equivalents, beta carotene 3.5 mg, thiamine 1.5 mg, riboflavin 1.6 mg, B6 2.2 mg, B12 4 mcg, niacin 17 mg, C 80 mg, D 10 mcg, E 10 mg, zinc 15 mg, copper 1.2 mcg and selenium 65 mcg2. Placebo: received a placebo tabletAn IFA supplement was given separately as part of the routine antenatal care and was not part of the MMN tablet.Tablets were given from the day of enrolment until delivery.
**Outcomes**	Primary outcome analyzed was low birth weightSecondary outcomes analyzed were preterm births, stillbirths, mode of delivery and small‐for‐gestational ageIt should be noted that the data for Small for gestational age were obtained from a separate report (Keats 2019) and not from the individual trial report.
**Notes**	Study intervention was approximately the RDA for pregnant or lactating women, except for vitamin A for which a higher amount was given.Out of 1106 women who were followed, 725 were HIV‐ve whereas 360 were HIV+ve and HIV status of 21 was indeterminate. We have used data of HIV‐ve women only in this review The intervention and the placebo groups were comparable at baseline except for the higher proportion of primigravida in the placebo group **Declarations of interest:** the trial authors declared no conflict of interest. **Funding sources:** Council for Development Research, Danish International Development Assistance (to HF), the Danish Council forMedical Research (to HF), Southampton Insurance, Zimbabwe, the Foundation of 1870, BASF Health and Nutrition, the Hørslev Foundation, the Dagmar Marshall Foundation, the Sophus Jacobsens Foundation, and the Lily Benthine Lunds Foundation

Risk of bias table

**Table:** 

Bias	Authors' judgement	Support for judgement
Random sequence generation (selection bias)	Low risk	Quote: "Allocation to daily supplementation with multimicronutrient or identicallooking placebo tablets was based on simple blocked randomisation. The digits 0‐5 in a computer‐generated random sequence were replaced by 6 preassigned permuted blocks of 4 AABB, ABAB, ABBA, BABA, BBAA, and BAAB; the digits 6‐9 were deleted" Comment: probably done
Allocation concealment (selection bias)	Low risk	Quote: "Containers with 110 multimicronutrient or placebo tablets, which were coded A or B, respectively, were delivered by themanufacturer togetherwith the code in 2 sealed envelopes. Duplicate containers, which corresponded to the random sequence, were consecutively numbered from 1 to 1800. The study participants were numbered consecutively at recruitment"Comment: Probably done
Blinding of participants and personnel (performance bias)	Low risk	Quote: "double blind", "multimicronutrient or identical‐looking placebo tablets"Comment: study participants and care providers were probably blinded to the treatment assignment
Blinding of outcome assessment (detection bias)	Low risk	Quote: "double blind", "multimicronutrient or identical‐looking placebo tablets"Comment: investigators were probably blinded to the treatment assignment
Incomplete outcome data (attrition bias)	High risk	Attrition was >20% and reasons for it were reported. Exclusions were not reported in the trial
Selective reporting (reporting bias)	Low risk	Comment: all outcomes in the methods section were presented in the paper
Other bias	Low risk	Comment: no other bias was identified

Gowachirapant 2017

**Table:** 

**Methods**	This randomized, double‐blind, placebo‐controlled trial was conducted in an urban setting in Bangalore, India and Bangkok, Thailand
**Participants**	Pregnant women presenting at their first antenatal visit, aged 18‐40 years, were eligible if they had a singleton pregnancy less than or equal to 14 weeks of gestation, were not lactating, had no chronic health issues, and not taking iodine‐containing supplements. Women were excluded if they had a thyroid stimulating hormone (TSH) concentration of more than 6 mIU/L at screening (these women were referred for treatment) Iodine group (n = 412), placebo group (n = 420)
**Interventions**	1. Iodine intervention group: received 200 ug of iodine orally, once a day2. Placebo group: received placebo once a day
**Outcomes**	None. Excluded from analysis. The iodine vs. placebo comparison was exlcuded from the review due to an insufficient number of studies.
**Notes**	**Declarations of interest:** Authors declare no conflict of interest **Funding sources:** Swiss National Science Foundation, Bern, Switzerland; Nestlé Foundation, Lausanne, Switzerland; Wageningen University and Research, Wageningen, Netherlands; and Swiss Federal Institute of Technology (ETH) Zurich, Switzerland.

Risk of bias table

**Table:** 

Bias	Authors' judgement	Support for judgement
Random sequence generation (selection bias)	Low risk	Quote: "We randomly assigned (1:1) women to receive either iodine supplementation or placebo by use of simple randomisation stratified by site. We used a computergenerated randomisation sequence. Masking was achieved by use of tablets with identical appearance and taste and coding of participants. An individual at Wageningen University who was not involved in the study coded the iodine and placebo supplements, and sealed envelopes with the code were sent to the data safety monitoring board members" Comment: probably done
Allocation concealment (selection bias)	Low risk	Quote: "We randomly assigned (1:1) women to receive either iodine supplementation or placebo by use of simple randomisation stratified by site. We used a computergenerated randomisation sequence. Masking was achieved by use of tablets with identical appearance and taste and coding of participants. An individual at Wageningen University who was not involved in the study coded the iodine and placebo supplements, and sealed envelopes with the code were sent to the data safety monitoring board members"Comment: probably done
Blinding of participants and personnel (performance bias)	Low risk	Quote: "Masking was achieved by use of tablets with identical appearance and taste and coding of participants… Participants, the study team assessing outcomes, and the team analysing the data remained masked to group assignment until analysis of the final data"Comment: probably done
Blinding of outcome assessment (detection bias)	Low risk	Quote: "Masking was achieved by use of tablets with identical appearance and taste and coding of participants… Participants, the study team assessing outcomes, and the team analysing the data remained masked to group assignment until analysis of the final data"Comment: probably done
Incomplete outcome data (attrition bias)	Unclear risk	Quote: "514 pregnant women in Bangkok and 318 in Bangalore (n = 832) were enrolled and randomly assigned to the two groups… We analysed data from 399 children at age 1 year, 430 children at age 2 years, and 330 children at age 5·4 years; attrition was balanced between groups and the main reason was a move away from the trial area around the time of delivery (Figure 1). Baseline characteristics of women who were lost to followup did not differ from those of women who completed the study, although women completing the study were enrolled a mean 4·2 gestational days later (appendix p 1)."
Selective reporting (reporting bias)	Low risk	Comment: All outcomes presented in the methods section were reported in the paper.
Other bias	High risk	Authors reported that measure of iodine concentration is not accurate, and particiants may have been misclassified. As well, they stated that messages about iodine nutrition might have effected participants' behaviours to consume more iodine rich foods. Lastly, allocation codes for one of the authors was broken for an interim analysis; it was indicated that results were not published.

Hafeez 2005

**Table:** 

**Methods**	This was a double blind, randomised case‐control study conducted in Pakistan, in two urban hospitals and one rural community Dates of study: April 2003‐April 2004
**Participants**	Pregnant women (n = 242) at 10‐16 weeks gestation were eligible. Exclusion criteria included any known systemic disease. Zinc group (n = 121), placebo group (n = 121)
**Interventions**	1. Zinc intervention group: received 20 mg of zinc to be consumed daily2. Placebo group: received glucoseWomen in both treatment arms received routine IFA supplementation
**Outcomes**	This study was included in two comparison in the review: MMN vs. IFA and Zinc vs. Placebo(1) MMN vs. IFA: the primary outcome analyzed was low birth weight (<2500 g) and the secondary outcome analyzed was preterm births.(2) Zinc vs. Placebo: the primary outcome analyzed was low birth weight (<2500 g) and the secondary outcome analyzed was preterm births.
**Notes**	**Declarations of interest:** none reported **Funding sources:** Saving Newborn Lives (SNL) of "Save the Children" USA

Risk of bias table

**Table:** 

Bias	Authors' judgement	Support for judgement
Random sequence generation (selection bias)	Low risk	Quote: "The patients were randomised into test and control groups by simple random sampling, and each patient was allotted a preassigned code on first contact." Comment: probably done
Allocation concealment (selection bias)	Low risk	Quote: "The patients were randomised into test and control groups by simple random sampling, and each patient was allotted a preassigned code on first contact. Both the patient and health worker were blinded to the content of the medication…"Comment: Probably done
Blinding of participants and personnel (performance bias)	Low risk	Quote: "Both the patient and health worker were blinded to the content of the medication"Comment: probably done
Blinding of outcome assessment (detection bias)	Low risk	Quote: Both the patient and health worker were blinded to the content of the medication (20 mg elemental zinc in the form of zinc sulphate powder capsule and a similar capsule as placebo), filled with glucose given from booking till the end of gestation"Comment: probably done
Incomplete outcome data (attrition bias)	Low risk	Quote: "About 15% patients were lost to follow up in the two groups which was nondifferential."
Selective reporting (reporting bias)	Low risk	Comment: All outcomes presented in the methods section were reported in the paper.
Other bias	Low risk	Comment: No other bias was identified

Hambidge 2019

**Table:** 

**Methods**	This study was a three‐arm, non‐masked, randomized controlled efficacy study conducted in rural or semirural locatiuons in the Democratic Republic of Congo (Equateur), Guatemala (Chimaltenango), India (Belagavi, North Karnataka) and Pakistan (Thatta, Sindh Province). Dates of study: Dec 2013 to Oct 2014
**Participants**	Participants (n = 7376) were recruited and enrolled from a total of 53 clusters part of the Human Development Global Network for Women's and Children's Health Research. Inclusion criteria included: 16‐35 years of age, partiy 0‐5, no current or planned contraceptive use, and expectation to conceive within the following 18 months. Exclusion criteria included: known history of obstetric complications or those unwilling to deliver in hospital. No women were excluded on the basis of weight, height or body mass index.
**Interventions**	In this study, lipid‐based nutrient supplementation (LNS) was evaluated compared to placebo or no LNS. The three arms differed in terms of the duration of the primary supplement (called Supplement 1).1) LNS provided from time of random assignment until delivery; participants were required to be on the primary supplement for >or equal to 3 months before conception2) primary supplement was provided during trimesters 2 and 3 of pregnancy, until delivery.3) participants were given no LNS throughout the studyIn addition to the supplement 1, women were provided with a second, daily, lipid‐based protein‐energy supplement (called Supplement 2). Participants were cautioned to not take any other micronutrient supplements or fortified food products while taking the supplements.
**Outcomes**	None analyzed. Excluded from analysis due to insufficient number of studies for the LNS vs placebo comparison.
**Notes**	**Declarations of interest:** Trial authors declared no conflict of interest **Funding Sources:** Supported by Bill &Melinda Gates Foundation grant OPP1055867 (KMH, NFK) and Eunice Kennedy Shriver National Institute of Child Health and Human Development and Office of Dietary Supplements, NIH grant U10 HD 076474 (NFK, KMH).

Risk of bias table

**Table:** 

Bias	Authors' judgement	Support for judgement
Random sequence generation (selection bias)	Low risk	Quote: "The DCC created the randomization scheme, centrally generating the allocation sequence for each site. Once the responsible home visitor research assistant identified an eligible participant, they received the random assignment generated by the site data manager from the centralized computerized data management system maintained by the DCC." Comment: probably done
Allocation concealment (selection bias)	Low risk	Quote: "The allocation ratio was 1:1:1 within blocks which randomly varied between sizes of 3, 6, or 9 for each site. Once the responsible home visitor research assistant identified an eligible participant, they received the random assignment generated by the site data manager from the centralized computerized data management system maintained by the DCC."Comment: probably done
Blinding of participants and personnel (performance bias)	High risk	Quote: "non‐masked"Comment: probably not blinded
Blinding of outcome assessment (detection bias)	Unclear risk	Comment: unclear
Incomplete outcome data (attrition bias)	High risk	Comment: >20% loss to follow‐up. Reasons for loss to follow‐up or exit from study were reported.
Selective reporting (reporting bias)	Low risk	Comment: all stated outcomes were reported
Other bias	Low risk	Comment: no other biases were identified

Hanieh 2013

**Table:** 

**Methods**	This was a cluster‐randomised trial comparing the impact of daily IFA, twice weekly IFA and twice weeklyMMN for pregnant women on birthweight in Ha Nam province, Vietnam Dates of study: September 2010‐2012
**Participants**	Participants (n = 1258) were pregnant women who were confirmed to be <16 weeks of gestation, resided in trial communes (n = 104), were >16 years of age, and were registered with the commune health station. Women with a high‐risk pregnancy including those with a multi‐fetal pregnancy (confirmed by palpation or ultrasound), a significant medical condition or severe anaemia (Hb concentration <80 g/L) at enrolment were excluded
**Interventions**	1. Daily IFA group (n = 426 participants; n = 34 communes) received an IFA tablet containing elemental iron (60 mg) and folic acid (0.4 mg), administered 7 days/week 2. Twice‐weekly IFA group (n = 425 participants; n = 35 communes) received an IFA capsule containing elemental iron (60 mg) and folic acid (1.5 mg), administered as 2 capsules/week 3. Twice‐weekly MMN group (n = 407 participants; n = 35 communes) received a MMN capsule containing elemental iron (60 mg), zinc (20 mg), iodine (300 ug), copper (4 mg), selenium (130 ug), vitamin A (1.6 mg), thiamine (2.8 mg), riboflavin (2.8 mg), niacin (36 mg), vitamin B6 (3.8 mg), B12 (5.2 ug), folic acid (1.5 mg), vitamin C (140 mg), vitamin D (400 IU) and vitamin E (20 mg), administered as 2 capsules/weekAll groups were provided supplements for the duration of the pregnancy until 3 months postpartum
**Outcomes**	For this review, the daily IFA group and the twice‐weekly MMN group were compared. The daily IFA group was used as the control group to maintain consistency with other studies that provided daily IFA supplementation.Primary outcomes analyzed were maternal iron‐deficiency anaemia, low birth weight, maternal anaemiaSecondary outcomes (Child) analyzed were serum/plasma ferritin, serum/plasma hemoglobin, child developmental outcomes, anaemia, neonatal mortality, stunting, preterm births, stillbirthsSecondary outcomes (maternal) analyzed were serum/plasma folate, serum/plasma transferrin receptor, serum/plasma hemoglobin, serum/plasma vitamin B12, ferritin deficiency, serum/plasma ferritin
**Notes**	**Declarations of interest:** the trial authors declared no conflict of interest.**Funding sources:** National Health and Medical Research Coucil of Australia (Grant number 628751)

Risk of bias table

**Table:** 

Bias	Authors' judgement	Support for judgement
Random sequence generation (selection bias)	Low risk	Quote: "Randomisation was performed by an independent statistician not involved in the study and blinded to the identy of the communes, using 'ralloc' in Stata (Stata‐Corp)" Comment: probably done
Allocation concealment (selection bias)	Low risk	“Supplements were received from the manufacturing company in blister packs, with a code A, B, or C embossed on each blister pack. The intermittent IFA and MMN capsules were identical in both appearance and packaging. The manufacturing company confidentially notified the chairperson of the Data Monitoring and Safety Committee at the University of Melbourne of the allocation code, and the code was kept in a locked file cabinet at the University of Melbourne Comment: probably done
Blinding of participants and personnel (performance bias)	Low risk	Quote: “The Investigators, field staff, and participants were blinded to the codes of the intermittent supplement groups throughout the study and during data analysis. Laboratory staff were unaware of the intervention groups. It was not possible to blind the field team to the daily supplementation arm, but participants were not informed about the dosing frequency of the intervention being given in other communes.”Comment: participants and caregivers were probably not blinded
Blinding of outcome assessment (detection bias)	Low risk	Quote: "The investigators, field staff, and participants were blinded to the codes of the intermittent supplement groups throughout the study and during data analysis. Laboratory staff were unaware of the intervention groups. It was not possible to blind the field teamto the daily supplementation arm" and "The allocation code was broken at the completion of data analysis. An independent teamundertook the BSID III assessments, and were blinded to the intervention arms"Comment: it is unclear whether outcome assessors were blinded to the treatment assignment
Incomplete outcome data (attrition bias)	Low risk	Attrition (until delivery) was 7.2% and (and was balanced between treatment arms); reasons were reported
Selective reporting (reporting bias)	High risk	Comment: growths outcomes including weight‐for‐age, underweight, weight‐forlength and wasted were mentioned in the methods section, however, results were not presented in the paper
Other bias	Low risk	Comment: no other bias was identified, including cluster‐design‐specific biases (recruitment bias, baseline imbalance, loss of clusters, and comparability with individually randomised trials). Any incorrect analysis was corrected by adjustment for clustering within data reported in this review

Hossain et al. 2012

**Table:** 

**Methods**	This was a single‐center, open‐label, randomized, controlled trial conducted in Karachi, Pakistan. Dates of study: September 2010 to May 2012
**Participants**	Pregnant women with a singleton pregnancy, attending the outpatient obstetric clinic at the Civil Hospital in Karachi, and who were at 20 weeks or less of gestation were eligible. Exclusion criteria included: hypertension, hypergylcemia, history of gestational diabetes, thyroid disorder, chronic liver diseases, and evidence of fetal anomaly in current pregnancy.
**Interventions**	Vitamin D group (n = 100): received 4000 IU of vitamin D (as 10 oral drops) dailyPlacebo group (n = 100): received no vitamin DParticipants in both arms received routine antenatal care which included 200 mg of ferrous sulfate (two times per day) and 600 mg of calcium lactate (daily).
**Outcomes**	Secondary outcomes analyzed were maternal serum vitamin D (nmol/L), preterm births, small for gestational age, and pre‐eclampsia
**Notes**	**Declarations of interest**: authors declare no conflict of interest **Funding sources**: grant from the Pakistan Medical Research Council

Risk of bias table

**Table:** 

Bias	Authors' judgement	Support for judgement
Random sequence generation (selection bias)	Low risk	Quote: "Sequential randomization schema was used and eligible gravidae were randomized into two groups"
Allocation concealment (selection bias)	Unclear risk	Allocation concealment not discussed. Insufficient information to make judgement.
Blinding of participants and personnel (performance bias)	Unclear risk	Blinding of participants and personnel not discussed. Insufficient information to make judgement.
Blinding of outcome assessment (detection bias)	Unclear risk	Blinding of outcome assessment not discussed. Insufficient information to make judgement.
Incomplete outcome data (attrition bias)	Low risk	Quote: "Of 207 gravidae enrolled, 193 completed the trial."
Selective reporting (reporting bias)	Low risk	Comment: Outcome presented in the methods sections were reported in the paper or previous studies.
Other bias	Low risk	Comment: No other bias was identified

Huy 2009

**Table:** 

**Methods**	This was a non‐randomized, non‐blinded, side‐by‐side effectiveness trial in three districts near Hanoi, Vietnam. Dates of study: 2000‐2005
**Participants**	(n = 1579) Pregnant women from the target populations attending antenatal care clinics, and receiving the intervention through the regular care program.
**Interventions**	1. MMN group: received UNIMMAP formulation to be consumed daily (Vitamin A 800 retinol equivalents, Vitamin D 200 IU, Vitamin E 10 mg, Vitamin C 70 mg, Vitamin B1 (thiamin) 1.4 mg, Vitamin B2 (riboflavin) 1.4 mg, vitamin B3 (niacin) 18 mg, vitamin B6 (pyridoxine) 1.9 mg, vitamin B12 (cobalamin) 2.6 mg, folic acid 400 ug, iron 30 mg, zinc 15 mg, copper 2 mg, selenium 65 ug, iodine 150 ug.)2. IFA group: received 60 mg of iron and 400ug of folic acid to be consumed dailyNutrition educationw as provided to women in both treatment arms.
**Outcomes**	Primary outcomes analyzed were low birth weight (<2500 g) and maternal anaemiaSecondary outcomes analyzed underweight (child), child anaemia, stunting and wasting
**Notes**	**Declarations of interest:** none stated **Funding sources:** none stated

Risk of bias table

**Table:** 

Bias	Authors' judgement	Support for judgement
Random sequence generation (selection bias)	High risk	Comment: non‐randomised trial
Allocation concealment (selection bias)	High risk	Comment: No allocation concealment. Control and intervention groups were assigned to districts.
Blinding of participants and personnel (performance bias)	High risk	Comment: participants were not blinded to their intervention arm; neither were the personnel
Blinding of outcome assessment (detection bias)	High risk	Comment: no blinding of the outcome assesment
Incomplete outcome data (attrition bias)	Unclear risk	Attrition is not discussed
Selective reporting (reporting bias)	Low risk	Comment: All outcomes presented in the methods section were reported in the paper.
Other bias	Low risk	Comment: No other bias was identified

Huybregts 2009

**Table:** 

**Methods**	This was a nonblinded, randomized controlled trial that was conducted in two rural villages in Burkina Faso. Dates of study: March 2006‐July 2008
**Participants**	Pregnant women (n = 1296) who were not planning to leave the area within the next 2 years were eligible. No other exclusion criteria was used.LNS group (n = 665); MMN group (n = 641)
**Interventions**	1. The MMN group received a tablet of the UNIMMAP formulation to be consumed daily. The UNIMMAP formulation includes 800 retinol equivalents of Vitamin A, 200 IU of Vitamin D, 10 mg of Vitamin E, 70 mg of Vitamin C, 1.4 mg of Vitamin B1, 1.4 mg of Vitamin B2, 18 mg of Vitamin B3, 1.9 mg of vitamin B6, 2.6 mg of vitamin B12, 400ug of folic acid, 30 mg of iron, 15 mg of zinc, 2 mg of copper, 65 ug of selenium, 150ug of iodine.2. LNS group: received a fortified food spread that consisted of 33% peanut butter, 32# soy flour, 15% vegetable oil and 20% sugar, also containing the UNIMMAP multiple micronutrient formulation.Participants were also randomly assigned to receive either a double or a triple dose of combined sulfadoxine‐pyrimethamine in the second and third trimesters as part of a different study on malaria prevention.
**Outcomes**	Primary outcomes: perinataly mortality and low birth weightSecondary outcomes: preterm births, small for gestational age, miscarriage, neonatal mortality and stillbirths
**Notes**	**Declarations of interest**: none reported **Funding sources:** none reported

Risk of bias table

**Table:** 

Bias	Authors' judgement	Support for judgement
Random sequence generation (selection bias)	Low risk	Comment: Randomisation scheme was generated by a computer program in permuted blocks of 4.
Allocation concealment (selection bias)	High risk	Comment: This was a non‐blinded randomised control trial.
Blinding of participants and personnel (performance bias)	High risk	Comment: This was a non‐blinded randomised control trial. While LNS and MMN were both packaged in plastic sachets, it Is unclear if this help to blind participants.
Blinding of outcome assessment (detection bias)	High risk	Comment: This was a non‐blinded randomised control trial.
Incomplete outcome data (attrition bias)	High risk	Comment: Of the 1296 participants enrolled into the study, outcome data was available for ~1020 pregnant women.
Selective reporting (reporting bias)	Low risk	Comment: All outcomes presented in the methods section were reported in the paper.
Other bias	Low risk	Comment: No other bias was identified

Jarjou et al. 2006

**Table:** 

**Methods**	This was a randomized double‐blind placebo‐controlled trial conducted in a rural setting, in West Iang, Gambia. Dates of study: unclear; Dates of recruitment: May 1995 to June 1999
**Participants**	Pregnant women (n = 155) with no history of any medical condition known to affect calcium or bone metabolism, who presented at the antenatal clinic at MRC Keneba before 20 weeks of gestation, who lived locally and had an uncomplicated singleton pregnancy, and were unlikely to be away from the area for prolonged periods, were eligible for the study. Calcium group (n = 77); placebo group (n = 78)
**Interventions**	1. Calcium group: received 500 mg of calcium, 3 times a day, for a total of 1500 mg of elemental calcium2. Placebo group: microcrystalline cellulose and lactose (3 placebo tablets, similar in shape, taste and texture to the calcium carbonate tablets)
**Outcomes**	None. Excluded from analysis due to no outcome of interest to pool with other studies.
**Notes**	**Declarations of interest**: authors declare no conflict of interest **Funding sources**: none reported

Risk of bias table

**Table:** 

Bias	Authors' judgement	Support for judgement
Random sequence generation (selection bias)	Low risk	Quote: "Assignment was by random permuted blocks of 4 to ensure that equal numbers of subjects were allocated to the supplement and placebo groups in each month and thereby to minimize the potential for seasonal confounding" Comment: probably done
Allocation concealment (selection bias)	Low risk	Quote: "Random assignment was stratified by clinic to take these clusters of villages into account and, thereby, minimize the potential for bias because of differences in antenatal care. Randomization was achieved by using published sets of tables.The code was held by a member of the study team (AP) who was not directly involved with the collection of data in the field or the laboratory and who had no contact with the study participants"Comment: probably done
Blinding of participants and personnel (performance bias)	Low risk	Quote: "The placebo consisted of 3 tablets of similar shape, taste, and texture in which the calcium carbonate was replaced with microcrystalline cellulose and lactose" and "randomisation codes were hidden from participants and researchers. The randomisation code was held by a member of the study team (AP) who was not directly involved with the collection of data in the field or the laboratory and who had no contact with the study participants."Comment: probably done
Blinding of outcome assessment (detection bias)	Low risk	Quote: "The randomisation code was held by a member of the study team (AP) who was not directly involved with the collection of data in the field or the laboratory and who had no contact with the study participants."Comment: probably done
Incomplete outcome data (attrition bias)	Low risk	Comment: 125 infants were available for analysis, from the 155 mothers who were enrolled. No significant different between groups that were analyzed and lost to follow‐up.
Selective reporting (reporting bias)	Low risk	Comment: All outcomes presented in the methods section were reported in the paper.
Other bias	Low risk	Comment: No other bias was identified

Kæstel et al. 2005

**Table:** 

**Methods**	This trial was conducted in Guinea‐Bissau Dates of study: January 2001‐October 2002
**Participants**	Pregnant women with <37 weeks of gestation were eligible for enrolment. A total of 2100 women were randomised into 3 groups, MMN RDA group, MMN 2 RDA group and 60 mg iron 400 mcg folic acid group
**Interventions**	15 micronutrients were included in the supplement at RDA level, except for folic acid that was included at 400 mcg level. Supplement consisted of vitamin A 800 mcg, D 200 IU, E 10 mg, C 70 mg, B1 1.4 mg, B2 1.4 mg, niacin 18 mg, B6 1.9 mg, B12 2.6 mg, folic acid 400 mcg, iron 30 mg, zinc 15 mg, copper 2 mg, selenium 65 mcg and iodine 150 mcg1. Intervention group (n = 1392) received MMN supplements (supplement RDA n= 695, supplement 2 RDA n = 697)2. IFA group received folic acid 400 mcg and iron 60 mg n = 708
**Outcomes**	Primary outcomes analyzed were low birth weight, maternal mortality and perinatal mortalitySecondary outcomes analyzed were small‐for‐gestational age, neonatal mortality, infant mortality, miscarriage, preterm births and stillbirthsIt should be noted that the data for small for gestational age were obtained from a separate report (Keats 2019) and not from the individual trial report.
**Notes**	Malaria is endemic but HIV prevalence is relatively low. IFA given to all participants.Therewas no significant difference in baseline characteristics between randomisation groups. We used the 1 RDA and control groups in this review **Declarations of interest:** the trial authors declared no conflict of interest. **Funding Sources**: the Royal Veterinary and Agricultural University, Denmark Development Assistnce (Danida), The Novo Nordisk Foundation, UNICEF, the Foundation of 1870 and Jakob and Olga Madsen's Foundation

Risk of bias table

**Table:** 

Bias	Authors' judgement	Support for judgement
Random sequence generation (selection bias)	Low risk	Quote: "Simple block randomisation with a block size of 150 was managed as follows: at entry, the project midwife randomly drew 1 piece of coloured paper corresponding to the colour code on the tablet containers from envelopes with initially 50 pieces of each of the three colours" Comment: probably done
Allocation concealment (selection bias)	Unclear risk	Quote: "at entry the project midwife randomly drew one piece of coloured paper corresponding to the colour code on the tablet containers from envelopes with initially 50 pieces of each of the three colours"Comment: insufficient evidence to determine whether allocation was concealed following generation of the randomisation sequence
Blinding of participants and personnel (performance bias)	Unclear risk	Quote: "three identical‐looking micronutrient supplements", code was kept secret from study participants, study personnel, and data analysts until data cleaning and preliminary data analysis has been carried out" and "the health workers who collected outcome data after delivery did not have any knowledge of intervention group of the women"Comment: participants and caregivers were probably blinded to the treatment assignment
Blinding of outcome assessment (detection bias)	Unclear risk	Quote: "three identical‐looking micronutrient supplements", code was kept secret from study participants, study personnel, and data analysts until data cleaning and preliminary data analysis has been carried out" and "the health workers who collected outcome data after delivery did not have any knowledge of intervention group of the women"Comment: outcome assessors were probably blinded to the treatment assignment
Incomplete outcome data (attrition bias)	Unclear risk	Exclusion (3.1%) and attrition (20.4%) data were reported along with their reasons
Selective reporting (reporting bias)	Low risk	Comment: all outcomes mentioned in the methods section were presented in the paper
Other bias	Low risk	Comment: no other bias was identified.

Khan 2016

**Table:** 

**Methods**	This was a community‐based, double blinded, randomized placebo‐controlled trial conducted in Jhelum, Pakistan. Dates of study: undisclosed
**Participants**	Pregnant women (n = 85) at 12 to 16 weeks of gestation were eligible. Exclusion criteria included participants with Type I or Type II diabetes, known high‐levels of vitamin D or those with more than 8 permanent teeth missing.
**Interventions**	Vitamin D group (n = 36): received 4000 IU of vitamin D daily for 6 monthsControl group (n = 49): received placebo tablets, daily for the same duration (6 months).
**Outcomes**	Outcome analyzed was serum/plasma vitamin D (umol/L)
**Notes**	**Declarations of interest:** Authors report no conflicts of interest **Funding sources**: USAID and a grant from Pakistan Initiative for Mothers and Newborns (PAIMAN)

Risk of bias table

**Table:** 

Bias	Authors' judgement	Support for judgement
Random sequence generation (selection bias)	Low risk	Quote: "The study participants were randomized in blocks. The block randomization was originally made for another study (aimed at different outcomes where over 400 pregnant women participated), but this study focuses on the birth weight and the periodontal outcomes only."
Allocation concealment (selection bias)	Low risk	Quote: "The investigators, study staff, and the participants were blinded about the group allocation. Allocation codes for vitamin D and placebo were kept in a sealed envelope in a locked cabinet at the Aga Khan University until the completion of the study."
Blinding of participants and personnel (performance bias)	Low risk	Quote: "The study drug and placebo were prepared by the pharmacy division at the Aga Khan University. it is an ISO certified department for the drug preparation and delivery. Both study drugs (vitamin D and placebo) were supplied in identical syrup bottles labelled either X or Y." and "The investigators, study staff, and the participants were blinded about the group allocation"
Blinding of outcome assessment (detection bias)	Low risk	Quote: "The study drug and placebo were prepared by the pharmacy division at the Aga Khan University. it is an ISO certified department for the drug preparation and delivery. Both study drugs (vitamin D and placebo) were supplied in identical syrup bottles labelled either X or Y." and "The investigators, study staff, and the participants were blinded about the group allocation"
Incomplete outcome data (attrition bias)	Low risk	Comment: Enrolled 86 pregnant women, of which 85 were included in the analysis
Selective reporting (reporting bias)	Low risk	Comment: Outcome presented in the methods sections were reported in the paper or previous studies.
Other bias	Low risk	Comment: No other bias was identified

Kirkwood 2010

**Table:** 

**Methods**	This was a cluster‐randomised, double‐blind placebo‐controlled trial (ObaapaVitA Trial) conducted in seven districts in Brong Ahafo Region in Ghana. Dates of study: December 2000‐October 2008
**Participants**	Women (n = 207,781) aged 15‐45 years living in seven predominantly rural districts in Brong Ahafo Region in Ghana, who were capable of giving informed consent and who planned to live in the trial area for at least 3 months were eligible for enrolment.Vit A group (n = 104,484); Placebo group (n = 103,297)
**Interventions**	1. Vit A group: received a soft‐gel capsule containing 25,000 IU (7500 ug) retinol equivalents in soybean oil2. Placebo group: received a soft‐gel capsule containing just soybean oil
**Outcomes**	Primary outcome analyzed was maternal mortality, and the secondary outcomes analyzed were serum/plasma retinol (umol/L) and stillbirths
**Notes**	**Declarations of interest:** authors report no conflict of interest **Funding sources:** UK Department for International Development (DFID) and USAID

Risk of bias table

**Table:** 

Bias	Authors' judgement	Support for judgement
Random sequence generation (selection bias)	Low risk	Comment: Randomisation was blocked and based on an independent, computer‐generated list of numbers.
Allocation concealment (selection bias)	Low risk	Comment: Cluster randomisation. Women were randomly assigned, according to their cluster of residence, to receive a vitamin capsule or placebo capsule orally once every week.
Blinding of participants and personnel (performance bias)	Low risk	Quote: "All participants and trial personnel (including those distributing capsules and collecting, processing, and analysing data) were masked to treatment assignment for the duration of the trial. Placebo capsules were identical in taste and appearance to the vitamin A capsules."
Blinding of outcome assessment (detection bias)	Low risk	Quote: "All participants and trial personnel (including those distributing capsules and collecting, processing, and analysing data) were masked to treatment assignment for the duration of the trial. Placebo capsules were identical in taste and appearance to the vitamin A capsules."
Incomplete outcome data (attrition bias)	Unclear risk	Comment: Women moved between treatment arms, due to migration into different clusters. Depending on the time period and number of visitation with a fieldworker determined if they were included in the analysis.
Selective reporting (reporting bias)	Unclear risk	Comment: Women were switched into different treatment arms if they migrated to a different cluster area.
Other bias	High risk	Comment: Post‐mortems were reported by close relative or friends of trial participants and infants. And included open ended histories and questions. Although authors indicated that two doctors had to code and come to a consensus about verbal post‐mortems (NOTE: primary outcome of study is on maternal mortliaty). Aside from maternal mortality outcomes, did not report modified ITT ananlysis for other outcomes. Women were switched into different treatment arms if they migrated to a different cluster area.

Korkmaz 2014

**Table:** 

**Methods**	This was a randomized double‐blind controlled trial conducted in Turkey Dates of enrolment: November 2010‐January 2012
**Participants**	Pregnant women (n = 108) with singleton pregnancies and without a risk factor for poor pregnancy outcomes, any systemic disorders, any medications or any previous surgery, were enrolled. Exclusion criteria also included iron deficiency anaemia (hemoglobin < 11gdL), pre‐existing diabetes, prior gestational diabetes, history of stillbirth, multiple gestation, active chronic systemic disease and anyone who smokes.Iron group (n = 36), Folate group (n = 36) and placebo group (n = 36)
**Interventions**	(1) Iron group received tablets containing 60 mg of iron to be consumed daily (2) Folate group received tablets containing 400 ug of folic acid to be consumed daily (3) Placebo group received placebo tablets
**Outcomes**	None. Excluded from analysis due to no outcomes to pool with other studies in the same comparison
**Notes**	**Declarations of interest:** Authors report no conflict of interest **Funding sources:** None reported

Risk of bias table

**Table:** 

Bias	Authors' judgement	Support for judgement
Random sequence generation (selection bias)	Low risk	Quote: "…according to a pregenerated randomization scheme created by the web site Randomization.com (http://www.randomization.com)."
Allocation concealment (selection bias)	Low risk	Quote: "All study medications were prepared by a clinician unaware of the patient's allocated study group in identical drug packages. Packages were given by a blinded attending physician."
Blinding of participants and personnel (performance bias)	Low risk	Quote: "Patients, all caregivers and the clinical observers who scored were blinded to the allocated treatment of the individual patient."
Blinding of outcome assessment (detection bias)	Low risk	Quote: "All study medications were prepared by a clinician unaware of the patient's allocated study group in identical drug packages. Packages were given by a blinded attending physician."
Incomplete outcome data (attrition bias)	Unclear risk	Attrition is not discussed. Insufficient information to make judgement
Selective reporting (reporting bias)	Low risk	Comment: All outcomes presented in the methods section were reported in the paper.
Other bias	Low risk	Comment: No other bias was identified

Kumar 2009

**Table:** 

**Methods**	This was a randomized, double‐blind controlled trial conducted in New Delhi, India. Dates of study: unclear; Dates of recruitment: January 2005‐December 2007
**Participants**	Healthy, primigravid women (n = 552) with normal blood pressure and 12‐25 weeks of gestation were eligible. Exclusion criteria included: multiple pregnancies, polyhdramnios, fetal malformation, diabetes, chronic hypertension, renal disease, cardiovascular disease, urolithiasis, or a blood pressure of 140/90 mm Hg or higher at the first visit or at the time of randomization.Calcium group (n = 290); Placebo group (n = 262)
**Interventions**	1. Calcium group: received 500 mg of calcium to be consumed daily2. Placebo group: received 500 mg of lactose to be consumed daily
**Outcomes**	Primary outcomes analyzed was low birth weight (<2500 g)Secondary outcomes: analyzed were preterm births, pre‐eclampsia, stillbirths and mode of delivery ‐ Caesarean section
**Notes**	**Declarations of interest:** none reported **Funding sources**: financially supported by the University Grant Commission, New Delhi, India.

Risk of bias table

**Table:** 

Bias	Authors' judgement	Support for judgement
Random sequence generation (selection bias)	Unclear risk	Quote: "The blinding of study participants and investigators was done by assigning coded numbers to the packets, each containing 60 tablets of calcium or placebo, according to a simple randomization sequence developed manually."
Allocation concealment (selection bias)	Low risk	Quote: "The blinding of study participants and investigators was done by assigning coded numbers to the packets, each containing 60 tablets of calcium or placebo, according to a simple randomization sequence developed manually."
Blinding of participants and personnel (performance bias)	Low risk	Quote: "The blinding of study participants and investigators was done by assigning coded numbers to the packets, each containing 60 tablets of calcium or placebo, according to a simple randomization sequence developed manually. The packets were then distributed to the participants using the random numbers in sequence. The randomization code was disclosed after completion of the study."
Blinding of outcome assessment (detection bias)	Low risk	Quote: "Identical‐looking tablets of calcium carbonate and placebo were prepared, each containing 500 mg of elemental calcium or lactose. The packets were then distributed to the participants using the random numbers in sequence. The randomization code was disclosed after completion of the study."
Incomplete outcome data (attrition bias)	Low risk	Attrition was <10%
Selective reporting (reporting bias)	Low risk	Comment: All outcomes presented in the methods section were reported in the paper.
Other bias	Low risk	No other bias dientified

Liu et al. 2013

**Table:** 

**Methods**	This was a double‐blind RCT conducted in 5 rural counties in Hebei Provinve, China Dates of study: May 2006‐April 2009
**Participants**	Pregnant women who recorded dates of their menstruation for greater than or equal to 2 months before they became pregnant, were nulliparous, greater than or equal to 20 years old, <20 week's gestation, legally competent, had not consumed micronutrient supplements other than folic acid in the prior 6 months, had a Hb level >10.0 g/dL, resided in and received prenatal care in 1 of 5 counties and consented to participate were eligible. 18,755 pregnant women with singleton pregnancies were randomised to group A (n = 6261), group B (n = 6252) and group C (n = 6262)
**Interventions**	The study had 3 arms. 1. Group A received folic acid 400 ug2. Group B received folic acid 400 ug and iron 30 mg 3. Group C received the UNICEF formulation containing folic acid 400 ug, Fe 30 mg, vitamin A 800ug, E 10 mg, D 5 mcg, C 70 mg, B1 1.4 mg, B2 1.4 mg, B6 1.9 mg, B12 2.6ug, niacin 18 mg, Zn 15 mg, Cu 2 mg, iodine 150ug, selenium 65ug. Supplements were take from enrolment until delivery.
**Outcomes**	This study was included in three comparisons: IFA vs FA (Group A vs. Group B), Iron vs. Placebo (Group A vs. Group B) and MMN vs. IFA (Group B vs. Group C) (1) For IFA vs FA, the primary outcomes analyzed were low birth weight, maternal anaemia and perinatal mortality. Secondary outcomes analyzed were preterm births, serum/plasma hemoglobin (g/L), infant mortality, serum/plasma transferrin receptor (mg/L), neonatal mortality and serum/plasma ferritin (ug/L) (2) For Iron vs. Placebo, the primary outcomes were low birth weight, maternal anaemia and perinatal mortality. Secondary outcomes analyzed were neonatal mortality, serum/plasma hemoglobin (g/L), preterm births, iron‐deficiency (ferritin), infant mortality, serum/plasma transferrin receptor (mg/L) and serum/plasma ferritin (ug/L) (3) For MMN vs IFA: primary outcomes analyzed were low birth weight, maternal anaemia and perinatal mortality. Secondary outcomes analyzed were neonatal mortality, serum/plasma hemoglobin (g/L), preterm births, iron‐deficiency (ferritin), infant mortality, serum/plasma transferrin receptor (mg/L) and serum/plasma ferritin (ug/L)
**Notes**	**Declarations of interest**: the trial authors declared no conflict of interest**Funding sources**: supported by a co‐operative agreement between Peking University Health Science Center and the Centers for Disease Control and Prevention

Risk of bias table

**Table:** 

Bias	Authors' judgement	Support for judgement
Random sequence generation (selection bias)	Low risk	Quote: "A statistician external to the study randomly assigned ten 4‐digit lot numbers to each of the 3 supplement types (masked to the formulation and allocation) and generated the assignment list for each county proportional to the expected number of participants;within each county and block, lot numbers were randomly assigned using RANUNI in SAS statistics software (SAS Institute Inc)" Comment: probably done
Allocation concealment (selection bias)	Low risk	Quote: "A statistician external to the study randomly assigned ten 4‐digit lot numbers to each of the 3 supplement types (masked to the formulation and allocation)"Comment: probably done
Blinding of participants and personnel (performance bias)	Low risk	Quote: "Aside from a pharmaceutical engineer who ensured allocation of lot numbers to the correct supplement formulations, all others (i.e., participants, local physicians, study personnel, and investigators) were masked to the identity of the supplements"Comment: probably done
Blinding of outcome assessment (detection bias)	Low risk	Quote: "Treatment codes were broken after completion of the study and main analyses.", "double blind"Comment: probably done
Incomplete outcome data (attrition bias)	Low risk	Attrition rate (6.2%) was <20% and reasons for attrition and exclusions were provided
Selective reporting (reporting bias)	Low risk	Comment: all outcomes mentioned were reported
Other bias	Low risk	Comment: no other bias was identified

López‐Jaramillo 1997

**Table:** 

**Methods**	This was a prospective randomized double‐blind controlled trial conducted in an urban setting in Quito, Ecuador. Dates of study: 1990‐1995
**Participants**	Pregnant teenagers (n = 260) attending antenatal outpatient clinics aged >17.5 years with nulliparity and residency in Quito (2800 m altitude) for a period of at least 1 year before conception, were eligible. Exclusion criteria included systolic/diastolic blood pressure greater than 120/80 mmHg, any chronic medical illness, history of cardiovascular, renal or endocrinologic disease, or if they were taking any type of drug, mineral or vitamin during recruitment. Calcium group (n = 125); placebo gropu (n = 135)
**Interventions**	Calcium group: received four tablets daily of 500 mg calcium, for a daily total of 2000mg of calciumPlacebo group: received four placebo tablets
**Outcomes**	Primary outcomes: Low birth weight (<2500 g)Secondary outcomes: preterm birth and pre‐eclampsia (blood pressure >140/90 mmHg and proteinuria >30 mg/dL (or one cross by dipstick on two ocassions 4‐24 hours apart)
**Notes**	**Declarations of interest**: none reported **Funding sources:** none reported

Risk of bias table

**Table:** 

Bias	Authors' judgement	Support for judgement
Random sequence generation (selection bias)	Low risk	Quote: "…used a table of random numbers to assign each patient independently in sequence to one of two treatment regimens." Comment: probably done
Allocation concealment (selection bias)	Unclear risk	Comment: Allocation conceal was not discussed. However, study indicated that tablets was prepared by a pharamaceutical group that is likely outside of the study.
Blinding of participants and personnel (performance bias)	Low risk	Quote: "Treatment assignment was double‐blind, with the composition of the tablets unknown to the patients and to all clinical personnel involved in the study."
Blinding of outcome assessment (detection bias)	Low risk	Quote: "Treatment assignment was double‐blind, with the composition of the tablets unknown to the patients and to all clinical personnel involved in the study."
Incomplete outcome data (attrition bias)	Low risk	Quote: "Fourteen patients failed to complete the protocol: three by change of residence to other towns, seven by change of marital status and change of prenatal care to private medical doctors, two by change to hospital of social insurance (Instituto Ecuatoriano de Seguridad Social), and two by noncompliance with the treatment. Of these 14 subjects, nine had been assigned to the calcium group and five to the placebo group. A total of 260 teenaged girls completed the study: 125 in the calcium‐supplemented group and 135 in the placebo group…Girls in both treatment groups were similar in age, height, marital status, and educational level."
Selective reporting (reporting bias)	Low risk	Comment: Certain outcomes, including, preterm delivery, LBW, and perinatal mortality were not reported, due to no significant association between supplementation. These outcomes were not the primary outcomes of the study.
Other bias	Low risk	No other bias was identified

Menendez 1995

**Table:** 

**Methods**	This was a randomized, double‐blind placebo‐controlled trial in a rural setting near the town of Farafenni, North Bank Division, in the Gambia. Dates of study: none reported
**Participants**	Multigravid pregnant women (n = 550) living in study villages were eligible. Women who were primigravidae or secungravidae and participating in a trial of antimalarial chemoprophylaxis were not included in the study.
**Interventions**	1. Iron group: received 60 mg of iron daily2. Placebo group: received placebo tablet dailyAll women in both treatment arms received 5 mg of folic acid weekly
**Outcomes**	This study was included in two comparisons: IFA vs FA and Iron vs. Placebo. Outcomes analyzed for both comparisons were the same: secondary outcomes analyzed were serum/plasma ferritin (ug/L) and serum/plasma hemoglobin (g/L)
**Notes**	The area is one of seasonal malaria with high levels of transmission occurring during the 4‐5 months of the rainy season; however, women in the study were not given anti‐malarials. Previous studies have shown that pregnant women with the AS genotype have normal fertility, normal abortion, prematurity and still birth rates. There is mixed opinion on the increase risk of anaemia during pregnancy for those with the AS genotype. Lastly, this genotype has shown to protect African children from clinical malaria (particularly the severe form). **Declarations of interest:** none reported **Funding sources:** none reported

Risk of bias table

**Table:** 

Bias	Authors' judgement	Support for judgement
Random sequence generation (selection bias)	Unclear risk	Comment: Process of randomisation was not discussed.
Allocation concealment (selection bias)	High risk	Comment: Intervention and control tablets were different colours to ensure correct administration.
Blinding of participants and personnel (performance bias)	High risk	Comment: Intervention and control tablets were different colours to ensure correct administration.
Blinding of outcome assessment (detection bias)	Low risk	Comment: All clinical and laboratory measurements were made by investigators who did not know the treatment code.
Incomplete outcome data (attrition bias)	Low risk	Comment: 520/550 women completed the study, and haemoglobin electrophoresis was performed for 500 of them.
Selective reporting (reporting bias)	Low risk	Comment: All outcomes presented in the methods section were reported in the paper.
Other bias	Low risk	No other bias was identified

Merialdi 2004

**Table:** 

**Methods**	This was a double‐blind randomized controlled trial conducted in Villa El Salvador (a shantytown) in Lima, Peru. Dates of study: 1998‐2000
**Participants**	Pregnant women (n = 242) between 10 and 16 weeks gestation with a singleton pregnancy, and considered to be at low risk (eligible for a vaginal delivery), and a resident of Villa El Salvador, and lived in coastal Peru for greater than or equal to 6 months before becoming pregnant were eligible.
**Interventions**	(1) MMN group/Zinc group: received a daily supplement containing 60 mg Fe (ferrous sulfate) and 250ug folic acid, with 25 mg Zn (zinc sulfate). (2) IFA group/Placebo group: received a daily supplement containing 60 mg Fe and 250ug of folic acid
**Outcomes**	This study was included in two comparisons of this review.MMN vs. IFA: Outcomes analyzed were preterm births, congenital anomalies and child developmental outcomes (general intelligence, motor function and verbal comprehension/language)Zinc vs. placebo: the outcome analyzed was preterm births
**Notes**	**Declarations of Interest:** none reported **Funding Sources:** none reported

Risk of bias table

**Table:** 

Bias	Authors' judgement	Support for judgement
Random sequence generation (selection bias)	Low risk	Quote: "The allocation sequence and randomization lists were computer generated by the investigators at the Bloomberg School of Public Health." Comment: probably done
Allocation concealment (selection bias)	Low risk	Quote: "The randomization code was made by the pharmaceutical company and maintained in a sealed and secured envelope in IIN in Lima; it was not opened until data analyses were largely completed. Samples of the supplements were analyzed by an independent laboratory to confirm the code."Comment: probably done
Blinding of participants and personnel (performance bias)	Low risk	Quote: "The supplements were manufactured in Lima, had the same appearance and taste, and both study personnel and study subjects were masked to treatment assignment."Comment: probably done
Blinding of outcome assessment (detection bias)	Low risk	Quote: "The supplements were manufactured in Lima, had the same appearance and taste, and both study personnel and study subjects were masked to treatment assignment…The randomization code was made by the pharmaceutical company and maintained in a sealed and secured envelope in IIN in Lima; it was not opened until data analyses were largely completed. Samples of the supplements were analyzed by an independent laboratory to confirm the code."Comment: probably done
Incomplete outcome data (attrition bias)	Low risk	Comment: 195/242 participants enrolled were included in the final analysis
Selective reporting (reporting bias)	Low risk	Comment: All outcomes presented in the methods section were reported in the paper.
Other bias	Low risk	Comment: No other bias was identified

Moore 2012

**Table:** 

**Methods**	This was a randomised trial with 4 intervention groups investigating the effects of prenatal and infancy nutritional supplementation on infant immune development in the West Kiang region of the Gambia Dates of study: October 2009‐September 2013
**Participants**	Participants (n = 875) were pregnant women who were 10‐20 weeks of gestation confirmed by ultrasound. Women who were pregnant >20 weeks of gestation on ultrasound assessment, enrolled in another Medical Research Council study, severely anaemic at booking (Hb <7 g/dL), or reported the onset of menopause were excluded
**Interventions**	The study had 4 pregnancy interventions given daily to participants from 12 weeks of gestation until deivery:1. IFA group received a daily tablet consisting of iron 60 mg and folate 400ug, representing the usual standard of care during pregnancy, as per Gambian Government guidelines (control group)2. MMN group received a daily tablet consisting of 15 micronutrients. The supplement provided twice the RDA of all micronutrients, except for iron (60 mg) and folic acid (400 ug). The rest of the micronutrients included vitamin A (1600 ug retinol equivalents), vitamin D (400 IU), vitamin E (20 mg), vitamin C (140 mg), vitamin B1 (2.8 mg), B3 (2.8 mg), B6 (2.8 mg), B12 (5.2 mg), niacin (36 mg), zinc (30 mg), copper (4 mg) selenium (130 ug), and iodine (300 ug).3. PE + Iron Folate group received a food‐based supplement developed by Valid International, providing a comparable level of iron and folate to the FeFol‐only arm, but with the addition of energy, protein and lipids4. PE + MMN group received a micronutrient fortified food‐based supplement also developed by Valid International, providing comparable levels of micronutrients to the MMN arm (including Iron folate), in addition to the energy and protein and lipid content From 6 months of age, infants were further randomised to receive either a lipid‐based nutritional supplement,with or without additional MMN, or placebo from 6‐12 months of age This study was included into two comparisons in this review: IFA vs. MMN (Group 1 and Group 2) and LNS vs. MMN (Group 4 and Group 2)
**Outcomes**	In the IFA vs. MMN comparison, the primary outcomes examined were low birth weight and maternal anaemia. The secondary outcomes were preterm births and small‐for‐gestational age.In the LNS vs. MMN comparison, the primary outcome was low birth weight. The secondary outcomes were preterm birth and small for gestational age.It should be noted that the data for small for gestational age were obtained from a separate report (Keats 2019) and not from the individual trial report.
**Notes**	In the review, the MMN group was used as the intervention group and the group was used as the comparison group **Declarations of interest:** the trial authors declared no conflict of interest. **Funding sources:** UK Medical Research Council (MRC) (MC‐A760‐5QX00) and the UK Department for International Development (DFID) under the MRC/DFID Concordat agreement. WJ and SEM are funded by the UK MRC programme MC UP 1005/1

Risk of bias table

**Table:** 

Bias	Authors' judgement	Support for judgement
Random sequence generation (selection bias)	Low risk	Quote: "Randomization into the trial is in blocks of 8, using an automated system, with the 8 groups reflecting the 8 combinations of prenatal and infancy supplements" Comment: probably done
Allocation concealment (selection bias)	Low risk	Quote: "Allocation of each supplement combination to a number between 1 and 8 was performed by DrMathilde Savy (IRD, France), with this information passed directly to the supplement manufacturers. Each box of supplement is then distinguished by a number between 1 and 8. An additional hard copy of the code assignment is held in the safe in Keneba, accessible by the field station senior administrator and only at the request of the trial monitors" and "Using the automated allocation system, a member of the data office at MRC Keneba, independent to the trial analysis, allocates mother‐infant pairs to their supplement codes and then generatesprinted labels for the supplement pots (including subject ID, name and the date of supplement period). Four members of the MRC Keneba field staff working on a different study then label the supplements using lists, by supplement allocation number (e.g. Group 1, women a, b, c, etc) provided by the data office:Comment: probably done
Blinding of participants and personnel (performance bias)	Unclear risk	Quote: "The antenatal arm of the trial is partly open, since it is not be possible to blind the field assistants or the women to the supplement type (tablet vs. LNS); all other investigators however will not know to which group the women belongComment: it is unclear whether participants and personnel were blinded to IFA and MMN groups
Blinding of outcome assessment (detection bias)	Low risk	Quote: "all other investigators however will not know to which group the women belongComment: outcome assessors were probably blinded to the treatment assignment
Incomplete outcome data (attrition bias)	High risk	Exclusion (until delivery) was 25.6% and reasons were reported. Attrition (until delivery) was 4.8% and reasons were not reported. Whether exclusion and attrition rates were balanced between treatment arms were not reported
Selective reporting (reporting bias)	Low risk	Comment: all outcomes in the methodssection were presented in the paper
Other bias	Low risk	Comment: no other bias was identified.

Muslimatun 2001

**Table:** 

**Methods**	This was a randomized double‐blind controlled, community‐based trial conducted in rural setting in Leuwiliang, West Java, Indonesia. Dates of study: November 1997 ‐ November 1999
**Participants**	Pregnant women (n = 243) who were between 16 and 20 weeks gestation, aged 17‐35 years, and parity <6, with a hemoglobin concentration between 80 and 140 g/L were eligible.MMN group (n = 122); IFA group (n = 121)
**Interventions**	1. MMN group: received two tablets, each containing 60 mg of iron, 250ug of folic acid and 2400 retinol equivalents of vitamin A, to be consumed on a weekly basis, for a total weekly supplementation of 120 mg of iron, 500ug of folic acid and 4800 retinol equivalents of vitamin A.2. IFA group: received two tablets, each containing 60 mg of iron and 250ug of folic acid, to be consumed on a weekly basis, for a total weekly supplementation of 120 g of iron and 250ug of folic acid.The participants were instructed not to take iron‐folic acid tablets from the national iron supplementation program, and village midwives were also advised not to give the tablets from the governmental program to the women in this study.
**Outcomes**	This study was included in two comparisons: MMN vs. IFA and Vitamin A vs. placebo (both groups received IFA)1) MMN vs. IFA: the primary outcome analyzed was maternal anaemia. Secondary outcomes analyzed were: iron deficiency (ferritin), maternal serum/plasma retinol (umol/L), maternal serum/plasma ferritin (ug/L), maternal serum/plasma hemoglobin (g/L), child developmental outcomes ‐ general intelligence, child serum/plasma retinol concentration (umol/L), child serum/plasma ferritin concentration, child serum/plasma hemoglobin concentration (g/L)2) Vit A vs. Placebo: the secondary outcomes analyzed were maternal serum/plasma retinol (umol/L) and maternal serum/plasma hemoglobin (g/L)
**Notes**	**Declarations of interest:** none reported **Funding sources:** none reported

Risk of bias table

**Table:** 

Bias	Authors' judgement	Support for judgement
Random sequence generation (selection bias)	Unclear risk	Comment: Participants were randomized on an individual basis.
Allocation concealment (selection bias)	Unclear risk	Comment: Study does not discuss allocation concealement
Blinding of participants and personnel (performance bias)	Unclear risk	Quote: "Both of the supplements given were identical in physical appearance to the iron‐folic acid tablets used for the national iron supplementation program and were provided by the same company, PT Kimia Farma, Indonesia." Comment: Every 4 weeks two authors would distribute supplements to health workers to giving to women on a weekly basis. It is likely the participants and personnel were blinded, however it is unclear if authors were blinded
Blinding of outcome assessment (detection bias)	Unclear risk	Comment: Blinding of outcomes was not discussed. Study states it was 'double‐blind'
Incomplete outcome data (attrition bias)	High risk	Comment: Initially, 366 pregnant women were enrolled in the study. 190 subjects completed biochemical data at baseline and near‐term. Subjects did not differ from the other subjects with respect to anthropometric and other biochemical variables at baseline. Compared with the other subjects, those included in the data analysis had (P < 0.05) lower serum soluble transferrin receptor concentrations at baseline.
Selective reporting (reporting bias)	Low risk	Comment: All outcomes presented in the methods section were reported in the paper.
Other bias	Low risk	Comment: No other bias was identified

Naghshineh 2016

**Table:** 

**Methods**	This was a double‐blind randomized controlled trial conducted in Isfahan, Iran. Dates of study: 2012
**Participants**	Nulliparous pregnant women (n = 140) at less than 16 weeks of gestation from outpatient clinics at "Shahid Behesti" hospital were eligible. Exclusion criteria include: vitamin D deficiency, use of aspirin and history of chronic hypertension, familial history of preterm labour, infection or other risk factors for preterm labour, gestational diabetes, renal disease or systemic lupus erythematus. As well, immigration, abruption and unwillingness to continue the study were also reasons for exlusion.
**Interventions**	Vitamin D group (n = 70): received 600 IU of vitamin D dailyControl group (n = 70): received daily supplementation, without vitamin D
**Outcomes**	Secondary outcomes reported: preterm birth and mode of delivery ‐ Caesarean section
**Notes**	**Declarations of interest:** none reported **Funding Sources:** Financial support was provided by the Isfahan University of Medical Sciences (Grant 392004)

Risk of bias table

**Table:** 

Bias	Authors' judgement	Support for judgement
Random sequence generation (selection bias)	Low risk	Quote: "Subjects meeting the inclusion criteria, using random‐maker software 'Random Allocation" were randomly divided into two groups of case and control
Allocation concealment (selection bias)	Unclear risk	Insufficient information to make judgement.
Blinding of participants and personnel (performance bias)	Low risk	Quote: "…patients were unaware of the treatment allocation and were followed up monthly by a doctor who was blinded to the study groups."
Blinding of outcome assessment (detection bias)	Low risk	Quote: "…patients were unaware of the treatment allocation and were followed up monthly by a doctor who was blinded to the study groups."
Incomplete outcome data (attrition bias)	Low risk	Quote: "Either, in case group, during the follow‐up period two subjects did not desire to continue and were excluded, finally, 138 subjects (68 cases and 70 controls) completed the study and analyzed.
Selective reporting (reporting bias)	Low risk	Comment: Outcome presented in the methods sections were reported in the paper or previous studies.
Other bias	Low risk	Comment: No other bias was identified

Osendarp et al. 2000

**Table:** 

**Methods**	This was a randomized double‐blind, placebo‐controlled trial that was conducted in an urban slum setting in Dhaka, Bangladesh Dates of study: March 1996 ‐ October 1997
**Participants**	Pregnant women (n = 559) between 12 and 16 weeks of gestation and who planned to remain at or near their residences in Dhaka for delivery, did not have an established medical risk for reduced or excessive birth weight (e.g. hypertension, renal disease or diabetes), were eligible.Zinc group (n = 269); placebo group (n = 290)
**Interventions**	1. Zinc group: received 30 mg of zinc daily2. Placebo group: received cellulose daily as a placebo
**Outcomes**	Primary outcomes: low birth weight (<2500 g)Secondary outcomes: Small for gestational age, serum/plasma zinc (umol/L), preterm births
**Notes**	Women with a hemoglobin concentration <90 g/L at 4 months and 7 months gestation were provided with iron‐folic acid supplementation, according to general practice in Bangladesh (200 mg of ferrous sulfate and 200ug of folate per day). **Declarations of interest:** none reported **Funding sources:** none reported

Risk of bias table

**Table:** 

Bias	Authors' judgement	Support for judgement
Random sequence generation (selection bias)	Low risk	Comment: The allocation sequence and randomization lists were computer generated by the investigators at the Bloomberg School of Public Health.
Allocation concealment (selection bias)	Low risk	Comment: The allocation sequence and randomization lists were computer generated by the investigators at the Bloomberg School of Public Health. Codes remained unknown to investigators and participants until the study was completed. Additionally, supplements were produced by a local manufacturer (Instituto Quimioterapico, SA, Lima, Peru), packaged as blister packs, and distributed to participants monthly.
Blinding of participants and personnel (performance bias)	Low risk	Comment: Study was a double‐blind design, and allocation codes were not broken until the end of the study.
Blinding of outcome assessment (detection bias)	Low risk	Comment: Study was a double‐blind design, and allocation codes were not broken until the end of the study.
Incomplete outcome data (attrition bias)	High risk	Comment: Of the 559 women enrolled, 113 (20.2%) were lost to follow‐up before delivery (20.4%) in the zinc‐supplemented group and 58 (20.0%) in the placebo grou. This was anticipated as the population was highly mobile and despite the restrictions at enrollment, most losses to follow‐up (n = 60) were due to out‐migration during the course of the study or to women leaving the area to deliver in their home villages.
Selective reporting (reporting bias)	Low risk	Comment: All outcomes presented in the methods section were reported in the paper.
Other bias	Low risk	Comment: No other bias was identified

Osrin et al. 2005

**Table:** 

**Methods**	This study was undertaken in Nepal. All women attending a designated antenatal clinic at Janakpur zonal hospital were considered for enrolment Dates of study: August 2002‐July 2004
**Participants**	Women were eligible for enrolment if an ultrasound examination confirmed a singleton pregnancy, a gestational age between 12‐20 completed weeks, no notable fetal abnormality, no existing maternal illness of a severity that could compromise the outcome of pregnancy; and the participant lived in an area of Dhanusha or the adjoining district of Mohattari accessible for home visits. Participants received supplements throughout pregnancy until delivery
**Interventions**	1. MMN group (n = 600) received tablets containing vitamin A 800 ug, vitamin E 10 mg, vitamin D 5 ug, B1 1.4 mg, B2 1.4 mg, niacin 18 mg, B6 1.9 mg, B12 2.6 ug, folic acid 400 ug, vitamin C 70 mg, iron 30 mg, zinc 15 mg, copper 2 mg, selenium 65 ug, and iodine 150 ug.2. IFA group (n = 600) received tablets containing iron 60 mg and folic acid 400 ug.There were 2 prespecified deviations from the protocol: if a participant Hb concentration was <70 g/L, she was given an extra 60 mg of iron daily, anthelmintic treatment, and herHb was rechecked after 1 month; and if a participant described night blindness at any time, she was given 2000 ug of vitamin A daily and referred for medical follow up
**Outcomes**	Primary outcomes: perinatal mortality, maternal anaemia and low birth weightSecondary outcomes: preterm births, stillbirths, neonatal mortality, miscarriage, small for gestational age, serum/plasma hemoglobin, serum/plasma retinol, mode of delivery, infant mortality, congenital anomalies, stunting, wasting, underweight (child), child developmental outcomes, serum/plasma ferritinIt should be noted that the data for SGA were obtained from a separate report (Keats 2019) and not from the individual trial report.
**Notes**	Infants were followed up to 3 months. Both groups of participants were comparable at baseline There is a discrepancy in the number of neonatal deaths reported. Figure: "Study Profile" in the Devakumar 2014 Lancet publication (p e655) reports 12 neonatal deaths in the control group and Osrin et al. 2005 reports 11 neonatal deaths in the control group. **Declarations of interest**: in the planning phase of the study, DO, SF, and AT attended an international investigators' meeting funded by the Micronutrient Initiative. After study completion, but before the paper was written, AV, DSM, and AT attended a second meeting funded by UNICEF. The other trial authors declared no conflict of interest **Funding sources**: Wellcome Trust, UK

Risk of bias table

**Table:** 

Bias	Authors' judgement	Support for judgement
Random sequence generation (selection bias)	Low risk	Quote: "Randomly allocated 1200 participant identification numbers by computer into two groups in permuted blocks of 50" Comment: Probably done
Allocation concealment (selection bias)	Low risk	Quote: "We did randomisation in advance of recruitment", "The allocation code was kept on fie in Kathmandu and London. We allocated every identification number a supplement container to last throughout the trial. Containers were filled with either intervention or control tablets in Kathmandu by a teammember who was otherwise uninvolved in the trial; these containers were then marked only with identification numbers and transported to the study centre in Janakpur" and "After screening, consent, and enrolment, one of us (YS) allocated participants sequential identification numbers and the corresponding supplement containers"Comment: probably done
Blinding of participants and personnel (performance bias)	Low risk	Quote: "The allocation code was kept on file in Kathmandu and London" and "Containers were filled with either intervention or control tablets in Kathmandu by a teammember who was otherwise uninvolved in the trial; these containers were then marked only with identification numbers and transported to the study centre in Janakpur. Intervention and control supplements were manufactured to look, smell and taste identical."Comment: participants and caregivers were probably blinded to the treatment assignment
Blinding of outcome assessment (detection bias)	Low risk	Quote: "The allocation code was kept on file in Kathmandu and London" and "Containers were filled with either intervention or control tablets in Kathmandu by a teammember who was otherwise uninvolved in the trial; these containers were then marked only with identification numbers and transported to the study centre in Janakpur. Intervention and control supplements were manufactured to look, smell and taste identical."Comment: outcome assessors were probably blinded to the treatment assignment
Incomplete outcome data (attrition bias)	High risk	Attrition was 5% and reasons for it were reported. Exclusion was 39.5% and reasons were not reported
Selective reporting (reporting bias)	Low risk	Comment: all outcomes mentioned in the methods section were presented in the paper
Other bias	Low risk	Comment: no other bias was identified.

Ouladsahebmadarek 2011

**Table:** 

**Methods**	This was a double‐blind randomized controlled trial conducted in Tehran, Iran. Dates of study: 2007‐2009
**Participants**	Healthy pregnant women (n = 960) who were in their first trimester of pregnancy with a singleton pregnancy, and had a hemoglobin concentration of >12 g/dL and had not taken any iron containing supplements in the last month, with a blood pressure <140/90 mmHg, were eligible. Exclusion criteria included anaemia (hemoglobin concentration <10.5 g/dL and <11 g/dL at the end of 2nd and 3rd trimesters respectively), miscarriage of current pregnancy, fetal abnormality and loss to followup.Iron group (n = 410) and placebo group (n = 372) were analyzed.
**Interventions**	Iron group: received a tablet with 30 mg of elemental iron and a multivitamin, daily.Placebo group: took one placebo and a multivitamin tablet daily.
**Outcomes**	Secondary outcomes analyzed were serum/plasma hemoglobin (g/L), serum/plasma ferritin (ug/L), pre‐eclampsia/eclampsia and preterm births
**Notes**	**Declarations of interest:** none reported **Funding sources:** none reported

Risk of bias table

**Table:** 

Bias	Authors' judgement	Support for judgement
Random sequence generation (selection bias)	Unclear risk	Insufficient information to make judgement.
Allocation concealment (selection bias)	Unclear risk	Insufficient information to make judgement.
Blinding of participants and personnel (performance bias)	Unclear risk	Quote: "double blind" However, beyond this, there is no additional information
Blinding of outcome assessment (detection bias)	Unclear risk	Insufficient information to make judgement.
Incomplete outcome data (attrition bias)	High risk	Comment: The intervention group had an attrition of 15%; however the control group had >20% loss to followup
Selective reporting (reporting bias)	Low risk	Comment: all outcomes mentioned in the methods section were presented in the paper
Other bias	Low risk	Comment: no other bias was identified.

Prawirohartono et al. 2011

**Table:** 

**Methods**	This was double‐blind, randomized controlled trial (ZIBUVITA trial) conducted in Yogyakarta, Indonesia. Dates of study: September 1995 to December 1999
**Participants**	Pregnant women who had a positive pregnancy test and was within the first 120 days of their pregnancy were eligible. Exclusion criteria included: women who were not married or did not have a life partner, gestational age >first trimester, peri‐menopausal women and women who were using hormone contraception or an intrauterine device
**Interventions**	There were four arms of this study. (1) Vitamin A only group (n = 546): received capsules that contained 2400 IU of vitamin A (as retinyl palmitate) daily (2) Zinc only group (n = 541): received 20 mg of zinc per day (3) Vitamin A + Zinc group (n = 543): received both vitamin A (2400 IU) and 20 mg of zinc (4) Placebo group (n = 543): received capsules that contained just the capsule filler (see below)All capsules contained 2 mg of dl‐alpha‐tocopherol as antioxidant, 350 mg of soybean oil, 20 mg of beeswax and 8 mg as capsule filler.
**Outcomes**	Excluded from analysis because outcomes reported in this study could not be pooled with other included studies in this review for the Vitamin A vs. placebo, and Zinc vs. placebo comparisons.
**Notes**	The authors noted that at the time this study was conducted, the Indonesian government recommended that pregnant women consume 60 mg of iron and 250 ug of folic acid daily for 90 days. The authors stated that surveys showed that the coverage of iron supplementation among pregnant women in the area varied greatly (31‐79%), and with low compliance (24% of the pregnant women in the study consumed the recommended number of iron tablets). **Declarations of interest:** None reported **Funding Sources:** Sources of financial support: MotherCare, John Snow Inc., Washington, USA and UNICEF, Jakarta, Indonesia; Gadjah Mada University and the University of Newcastle, Newcastle, Australia; and infrastructure support from the Community Health and Nutrition Research Laboratories, Medical School, Gadjah Mada University and Ministry of Health, Republic of Indonesia through the Third Community Health and Nutrition Development Project Loan from the World Bank (IBRD Loan No. 3550‐IND). The development of this manuscript was supported by the Centre for Global Health at Umeå University, FAS and the Swedish Council for Working Life and Social Research (grant number 2006‐1512)

Risk of bias table

**Table:** 

Bias	Authors' judgement	Support for judgement
Random sequence generation (selection bias)	Low risk	Quote: "Pregnant women were randomly allocated in a 1:1:1:1 ratio in blocks of twelve based on a list of treatment numbers derived from a pseudo‐random number generated with SAS software (SAS Institute, Inc., Cary, NC, USA)."
Allocation concealment (selection bias)	Low risk	"The treatment allocation sequence was prepared and held at the University of Newcastle, New South Wales, Australia."
Blinding of participants and personnel (performance bias)	Low risk	Quote: "All investigators, field and laboratory staff, and participants were blinded to the treatment code until all field data had been collected, and preliminary data analysis by coded groups had been completed"
Blinding of outcome assessment (detection bias)	Low risk	Quote: "The supplements were packed in plastic strips in identical, opaque pink capsules."…"All investigators, fi eld and laboratory staff, and participants were blinded to the treatment code until all field data had been collected, and preliminary data analysis by coded groups had been completed"
Incomplete outcome data (attrition bias)	High risk	Comment: >20% loss to follow up
Selective reporting (reporting bias)	Unclear risk	Comment: It is unclear what all of the outcomes the authors intended to measure at the start of the study
Other bias	High risk	Comment: This study was started in 1994, but the only published material from this original trial are follow‐up studies published in 2011 and 2013.

Preziosi 1997

**Table:** 

**Methods**	This was a randomized double‐blind controlled trial conducted in Niamey, Niger Dates of study: not reported
**Participants**	Pregnant women (n = 197) aged 17‐40 years at 28 weeks +/‐ 21 days of gestation were eligible. None of the participants had medical or obstetric problems at enrolment.Iron group (n = 99) and placebo group (n = 98)
**Interventions**	Iron group: received 100 mg of elemental iron per day (in two tablets of 50 mg of elemental iron)Placebo group: received placebo. It is unclear if the placebo was given a placebo tablet, or if they were given nothing at all.
**Outcomes**	Outcomes analyzed were: maternal anaemia, iron deficiency anaemia, and iron deficiency (ferritin)
**Notes**	"The double‐blind design of this study was made possible by the absence of routine administration of iron or folic acid supplementation to pregnant women in Niamey MCH centers. Although supplementation is strongly recommended by the World Health Organization and UNICEF (19), iron and folic acid tablets were neither given nor prescribed routinely to pregnant women." **Declarations of Interest**: None reported **Funding sources**: None reported

Risk of bias table

**Table:** 

Bias	Authors' judgement	Support for judgement
Random sequence generation (selection bias)	Unclear risk	Insufficient information to make judgement
Allocation concealment (selection bias)	Unclear risk	Insufficient information to make judgement
Blinding of participants and personnel (performance bias)	High risk	Quote: "The double blind design of this study was made possible by the absence of routine administration of iron or folic acid supplementation to pregnant women in Niamey MCH centers." Comment: probably done
Blinding of outcome assessment (detection bias)	Unclear risk	Insufficient information to make judgement
Incomplete outcome data (attrition bias)	Unclear risk	Insufficient information to make judgement
Selective reporting (reporting bias)	Low risk	Comment: all outcomes mentioned in the methods section were presented in the paper
Other bias	Low risk	No other bias identified

Ramakrishnan et al. 2003

**Table:** 

**Methods**	This randomized controlled trial was conducted near the city of Cuernavaca, in the state of Morelos, Mexico. Dates of study: 1997‐2000
**Participants**	Pregnant women who were <13 weeks of gestation, were not alrady receiving MMN supplementation and who agreed to participate were included in the study. A total of 873 women were randomised into the MMN group (n = 435, mean age 23.09 ± 5.48) and the iron only group (n = 438, mean age 23.00 ± 5.08)
**Interventions**	1. MMN group: tablets included the following vitamins and minerals: iron 60 mg as ferrous sulphate, folic acid 215 mcg, vitamin A 2150 IU, vitamin D3 309 IU, vitamin E 5.73 IU, thiamin 0.93 mg, riboflavin 1.87 mg, niacin 15.5 mg, vitamin B6 1.94 mg, vitamin B12 2.04 mcg, vitamin C 66.5 mg, zinc 12.9 mg, magnesium 252 mg 2. Control group: given iron‐only tablets with 60 mg of iron as iron sulphate All were given orally, from recruitment 6 days a week until delivery
**Outcomes**	Primary outcomes included maternal iron‐deficiency anaemia, maternal anaemia, low birth weight, and perinatal mortalitySecondary outcomes included small for gestational age, serum/plasma folate, serum/plasma retinol, serum/plasma hemoglobin, preterm births, stillbirths and serum/plasma ferritin.It should be noted that the data for small for gestational age were obtained from a separate report (Keats 2019) and not from the individual trial report.
**Notes**	Data on birth outcomes were only available for 656 pregnancies (MMN group n = 328 and control group, iron only n = 326). The 2 groups did not differ significantly in most of the characteristics at recruitment, except for marital status (more single mothers in MMN supplementation group) and mean BMI (significantly lower in the MMN supplementation group)**Declarations of interest**: the trial authors declared no conflict of interest. **Funding Sources**: Thrasher Research Fund, United Nationsl Children's Fund (UNICEF) New York, Conacyt, and the Instituto Nacional de Salud Pública, Mexico

Risk of bias table

**Table:** 

Bias	Authors' judgement	Support for judgement
Random sequence generation (selection bias)	Low risk	Quote: "Randomization was carried out by using 4 color‐coded groups (2 per treatment) that were assigned a priori with the use of a computer‐generated list" Comment: probably done
Allocation concealment (selection bias)	Low risk	Quote: Four colors were used to ensure masking and were assigned at random before the study began to a list of serial numbers from 1 to 1000" and "pregnant women were allocated to the pre assigned color code as they were added to this list at the time of recruitment"Comment: probably done
Blinding of participants and personnel (performance bias)	Low risk	Quote: "All study personnel and investigators were blinded to the group assignment, the details of which were kept at Emory University and the INSP in sealed envelopes that were opened only after preliminary data analysis was completed"Comment: participants and caregivers were probably blinded to the treatment assignment
Blinding of outcome assessment (detection bias)	Low risk	Quote: "All study personnel and investigators were blinded to the group assignment, the details of which were kept at Emory University and the INSP in sealed envelopes that were opened only after preliminary data analysis was completed"Comment: outcome assessors were probably blinded to the treatment assignment
Incomplete outcome data (attrition bias)	High risk	Exclusion was 5.2% but reasons for it were not reported. Attrition (26.2%) along with their reasons were reported
Selective reporting (reporting bias)	Low risk	Comment: all outcomes mentioned in the methods section were presented in the various publications of this trial
Other bias	Low risk	Comment: no other bias was identified.

Roberfroid et al. 2008

**Table:** 

**Methods**	This was a factorial, double‐blind, RCT conducted in the Hounde health district of Burkina Faso Dates of study: March 2004‐October 2006
**Participants**	Pregnant women irrespective of gestational age. Exclusion criterion was if women planned to leave area within 2 years
**Interventions**	1. Intervention group (n = 714) received vitamin A 800 mcg, D 200 IU, E 10 mg, B1 1.4 mg, B2 1.4 mg, niacin 18 mg, folic acid 400 mg, B6 1.9 mg, B12 2.6 mcg, C 70 mg, zinc 15 mg, iron 30 mg, copper 2 mg, selenium 65 mcg, iodine 150 mcg2. Placebo group (n = 712) received folic acid 400 mcg and iron 60 mg
**Outcomes**	Primary outcomes analyzed were low birth weight, maternal anaemia and perinatal mortality.Secondary outcomes analyzed were diarhhea, stillbirths, neonatal mortality, small for gestational age, preterm births, mode of delivery ‐ Caesarean section, infant mortality, miscarriage and fever.It should be noted that the data for SGA were obtained from a separate report (Keats 2019) and not from the individual trial report.
**Notes**	Supplement intake was observed directly and given till 3 months after delivery. Participants were also randomly assigned to receive either malaria chemoprophylaxis (300 mg chloroquine/week) or intermittent preventive treatment (1500 mg sulfadoxine and 75 mg pyrimethamine once in the second and third trimester) All participants received albendazole 400 mg during second and third trimester. Severely anaemic women received ferrous sulphate 200 mg and folic acid 0.25 mg twice daily for 3 months regardless of their allocation groups The study groups were similar with respect to baseline characteristics except for small difference inHb (lower in intervention group) and BMI (lower in control group). Stunting, wasting, underweight, and infant mortality during the first year of life were presented as hazard ratios and could not be included in the analysis of outcome using risk ratios **Declarations of interest**: the trial authors declared no conflict of interest. **Funding sources**: Nutrition Third World and the Belgian Ministry of Development

Risk of bias table

**Table:** 

Bias	Authors' judgement	Support for judgement
Random sequence generation (selection bias)	Low risk	Quote: "The randomisation scheme was generated by a computer program in permuted blocks of 4" Comment: probably done
Allocation concealment (selection bias)	Low risk	Quote: "Randomzation numbers were sealed in opaque envelopes. At each inclusion, the consulting physician opened the next sealed envelope and transmitted the randomisation number to a pharmacist managing the allocation sequence and, the packaging of drugs in Center Muraz. The pharmacist was also blinded to the intervention. Individual plastic zip bags contained 31 tablets each and were labelled with the participants name, address, and identification numbers only"Comment: Probably done
Blinding of participants and personnel (performance bias)	Low risk	Quote: "double blind", "Intervention and control micronutrient tablets were identical in appearance" and "code was kept secret from study participants and staff until completion of preliminary data analysis" and "Pharmacist was also blinded to the intervention"Comment: participants and caregivers were probably blinded to the treatment assignment
Blinding of outcome assessment (detection bias)	Low risk	Quote: "double blind", "Intervention and control micronutrient tablets were identical in appearance" and "code was kept secret from study participants and staff until completion of preliminary data analysis" and "Pharmacist was also blinded to the intervention"Comment: outcome assessors were probably blinded to the treatment assignment
Incomplete outcome data (attrition bias)	Low risk	Attrition was 7.5% and reason for it was provided. Only 1 woman was excluded because of therapeutic abortion
Selective reporting (reporting bias)	Low risk	Comment: all outcomes mentioned in the methods section were presented in the paper
Other bias	Low risk	Comment: no other bias was identified.

Roth 2013 (AViDD)

**Table:** 

**Methods**	This was a randomized double‐blind placebo‐controlled trial (AViDD trial) conducted in Dhaka, Bangladesh Dates of study: August 2010 ‐ April 2011
**Participants**	Pregnant women (n = 160) aged 18 to <35 years, between 26 to <30 weeks of gestation, with a current residence in Dhaka at a fixed address with plans to stay throughout the pregnancy and for at least one month past the date of delivery, and planning to deliver at the maternity center were eligible for the study. Exclusion criteria included: use of any dietary supplement containing more than 400IU per day(10 mcg/day) of vitamin D within the month prior to enrollment, or refusal to stop taking supplemental vitamin D at any dose after enrollment; curent use of anti‐convulsant or anti‐mycobacterial (tuberculosis) medications, severe anaemia (hemoglobin <70 g/L); high blood pressure (systolic blood pressure greater than or equal to 140 mmHg or diastolic blood pressure greater than or equal to 90 mmHg; positive urine dipstick of proteinuria or glycosuria; complicated medical or obstetric history, reported prior history of delivery of an infant with a major congenital anomaly, birth asphyxia or perinatal death.Vitamin D group (n = 80), placebo (n = 80)
**Interventions**	1. Vitamin D group: received an oral liquid supplement containing 35,000 IU of Vitamin D3 (20,000IU D3 per mL) per week2. Placebo group: received an oral liquid placebo containing Miglyol oil 812 per weekWomen in both treatment arms were given IFA (66 mg of iron and 350ug of folic acid) during free antental and obstetric care
**Outcomes**	This study was incldued in two comparisons in the review: MMN vs. IFA and Vit D vs. placebo (1) MMN vs. IFA: outcomes analyzed were stillbirths, neonatal mortality, preterm births and mode of delivery ‐ Caesarean section (2) Vitamin D vs. Placebo: outcomes analyzed were preterm, mode of delivery ‐ Caesarean section, serum/plasma vitamin D (nmol/L) and serum/plasma calcium (mg/dL)
**Notes**	Supplement doses were measured in disposable plastic syringes and orally administered by study personnel, beginning at the baseline visit and continuing thereafter at 7‐day intervals until delivery. Missed doses could be administered up to 7 days after the scheduled date, but otherwise were skipped. Compliance was 99.2% (2.7) and 99.4% (2.9) for placebo and vitamin D, respecitvely.**Declarations of interest:** The authors declare no conflicts of interest.**Funding sources:** The intervention trial was funded by The Thrasher Fund (Salt Lake City, UT), and the follow‐up study was funded by the Department of Pediatrics, Hospital for Sick Children, University of Toronto.

Risk of bias table

**Table:** 

Bias	Authors' judgement	Support for judgement
Random sequence generation (selection bias)	Low risk	Quote: "Participants were randomly assigned to one of two masked parallel intervention groups, with allocation concealment: vitamin D3 (cholecalciferol) 35,000 IU/week or matched placebo. Assignment was based on a computergenerated randomization list, with 1:1 allocation, using permuted blocks of size 4 and 8."
Allocation concealment (selection bias)	Low risk	Quote: "Participants were randomly assigned to one of two masked parallel intervention groups, with allocation concealment: vitamin D3 (cholecalciferol) 35,000 IU/week or matched placebo. The allocation sequence was prepared by icddr,b personnel not otherwise involved in the study, and was concealed from investigators."
Blinding of participants and personnel (performance bias)	Low risk	Quote: "Popular Pharmaceuticals Ltd. (Dhaka) prepared the supplements off‐site using individual opaque glass vials labeled with unique participant identifiers based on the randomization list…The active and placebo supplements were identical in appearance and tasteless…Participants and research staff (including lab personnel) were blinded to allocation"
Blinding of outcome assessment (detection bias)	Low risk	Quote: "Popular Pharmaceuticals Ltd. (Dhaka) prepared the supplements off‐site using individual opaque glass vials labeled with unique participant identifiers based on the randomization list…The active and placebo supplements were identical in appearance and tasteless…Participants and research staff (including lab personnel) were blinded to allocation"
Incomplete outcome data (attrition bias)	Low risk	Comment: Less than 10% loss to follow‐up from original trial.
Selective reporting (reporting bias)	Low risk	Comment: All outcomes presented in the methods section were reported in the paper
Other bias	Low risk	Comment: No other bias was identified

Roth et al., 2018 (MDIG)

**Table:** 

**Methods**	This was a randomized, double‐blind, placebo‐controlled, dose‐ranging trial conducted in Dhaka, Bangladesh. Dates of enrolment: March 2014‐September 2015
**Participants**	Healthy pregnant women (n = 1300) between 17 and 24 weeks of gestation were included in the study. Exclusion criteria included history of any medical condition or medications that may predispose them to vitamin D sensitivity, altered vitamin D metabolism and/or hypercalcemia, including active tuberculosis, current therapy for tuberculosis, sarcoidosis, history of renal/ureteral stones, parathyroid disorders, renal or liver failure, or use of anti‐convulsants. As well, any high risk pregnancy was excluded on the basis of any of the following: severe anaemia (hemoglobin <70 g/L), proteinuria of > or equal to 300 mg/dl (3+ or 4+) based on the urine dipstick, hypertension. Further, other exclusion criteria include multiple gestation, major congenital anomalies and severe oligyhydramnios
**Interventions**	There were five arms in this study: (1) One group (n = 260) received placebo throughout the prenatal period and for 26 weeks post partum. (2) One group (n = 260)received 4200 IU of vitamin D per week through the prenatal period, but none during the post partum period (3) One group (n = 260)received 16,800 IU of vitamin D per week thorugh the prenatal period, but none during the post partum period (4) One group (n = 260)received 28,000 IU of vitamin D per week thorugh the prenatal period, but none during the post partum period (5) One group (n = 260)received 28,000 IU of vitamin D per week through the prenatal period, as well as, 28,000 IU of vitamin D for 26 weeks post‐partumAll participants throughout the intervention phase were given calcium (500 mg per day), iron (66 mg per day) and folic acid (350ug per day)
**Outcomes**	For this review, group1 (placebo) and group 4 (28,000 IU of prenatal vitamin D, weekly) were compared. Group 4 was chosen as the comparison group because its daily dose of 4000 IU (28,000 IU per week/7 days) of vitamin D was consistent with other studies' supplementation routines within the comparison.Outcome analyzed were preterm births, small for gestational age, serum/plasma calcium (mg/dL), serum/plasma vitamin (nmol/L), mode of delivery ‐ Caesarean sectionThis study was also included in the comparison MMN vs. IFA. Outcomes analyzed were: preterm births, stillbirths, stunting, wasting, mode of delivery ‐ Caesarean section, neonatal mortality, congenital anomalies, SGA, low birthweight, maternal mortality and infant mortality.
**Notes**	**Declarations of Interest:** authors report no conflict of interest **Funding Sources**: the Bill and Melinda Gates Foundation (OPP1066764).

Risk of bias table

**Table:** 

Bias	Authors' judgement	Support for judgement
Random sequence generation (selection bias)	Low risk	Quote: "A computer‐generated, simple randomization scheme was created independently by the trial statistician."
Allocation concealment (selection bias)	Low risk	Quote: "The master list linking participant identifiers to supplementation groups was held by the supplement manufacturer and not accessed by any trial personnel until final group assignments were revealed. Concealment of trial‐group assignments was ensured with the use of prelabeled and sequentially numbered but otherwise identical supplement vials, which were provided to participants in accordance with the assignment sequence."
Blinding of participants and personnel (performance bias)	Low risk	Quote: "The master list linking participant identifiers to supplementation groups was held by the supplement manufacturer and not accessed by any trial personnel until final group assignments were revealed. Concealment of trial‐group assignments was ensured with the use of prelabeled and sequentially numbered but otherwise identical supplement vials, which were provided to participants in accordance with the assignment sequence."
Blinding of outcome assessment (detection bias)	Low risk	Quote: "Tablets with different doses were identical in appearance and taste."
Incomplete outcome data (attrition bias)	Low risk	Comment: loss to follow‐up was <20% in the mothers and live infant populations
Selective reporting (reporting bias)	Low risk	Comment: All outcomes presented in the methods section were reported in the paper
Other bias	Low risk	Comment: no other bias identified

Saaka 2009

**Table:** 

**Methods**	This was a prospective randomized double‐blind controlled trial conducted in an urban setting, in Upper West Region of Ghana Dates of study: September 2005 ‐ November 2006
**Participants**	Pregnant women (n = 600) who were no more than 16 weeks of gestation were eliglble. Exclusion criteria included if they were on any other form of zinc supplementation at any dosage, or had severe anaemia (hemoglobin <7.0 g/dL)MMN/Zinc group (n = 299); IFA/Placebo group (n = 301)
**Interventions**	1. MMN group/Zinc group: received 40 mg of zinc (as zinc gluconate) and 40 mg of iron (as ferrous sulphate) to be consumed every other day2. IFA group/Placebo group: received 40 mg of iron (as ferrous sulphate) to be consumed every other dayWomen in both treatment arms received 400ug of folic acid and chemoprophylaxis for malaria with sulphadoxine pyrimethamine.
**Outcomes**	This study was included in two comparisons in the review: MMN vs IFA and Zinc vs. placebo. (1) MMN vs IFA: Primary outcome analyzed was low birth weight (<2500 g). Secondary outcomes analyzed were preterm births and small for gestational age. (2) Zinc vs. Placebo: Primary outcome analyzed was low birth weight (<2500 g). Secondary outcomes analyzed were preterm births and small for gestational age.
**Notes**	**Declarations of Interest**: none reported **Funding sources**: none reported

Risk of bias table

**Table:** 

Bias	Authors' judgement	Support for judgement
Random sequence generation (selection bias)	Low risk	Quote: "Participants were randomly assigned to one of the two study groups. An independent statistician generated the allocation schedule/sequence using computer‐generated random numbers using the Excel software. After obtaining informed consent for enrollment, the midwife assigned participants in blocks of hundreds until the required sample size was met. There was no stratification during the randomization."
Allocation concealment (selection bias)	Low risk	Quote: "An independent statistician generated the allocation schedule/sequence using computer‐generated random numbers using the Excel software. After obtaining informed consent for enrollment, the midwife assigned participants in blocks of hundreds until the required sample size was met. There was no stratification during the randomization. The assignment of participants to study conditions was administered at the study centre. The treatment allocations generated were put in opaque envelopes and serially numbered. Each envelope contained a card on which the specific treatment was indicated. To safeguard the allocation schedule/sequence, envelopes were opened sequentially and only after the participant's name and other details were written on the envelope. The statistician who generated the allocation schedule kept the schedule that contained the random allocations until the collection of data was completed."
Blinding of participants and personnel (performance bias)	Low risk	Quote: "The iron‐zinc and iron‐only supplements were precoded and supplied by the Nutricaps Pharmaceutical Company, USA. The supplements (in the form of capsules) were of the same shape, colour, and taste."
Blinding of outcome assessment (detection bias)	Low risk	Quote: "The iron‐zinc and iron‐only supplements were precoded and supplied by the Nutricaps Pharmaceutical Company, USA. The supplements (in the form of capsules) were of the same shape, colour, and taste."
Incomplete outcome data (attrition bias)	Low risk	Quote: "the total loss‐to‐follow‐up rate was 9.5%; this was less than 10.0% and was, therefore, not a threat to the validity and power of the study. In total, 9.0% (n = 27) of the participants (n = 299) were lost‐to‐follow‐up in the zinc‐supplemented group compared to 9.9% (n = 30) of the participants (n = 301) in the control group. The minimum total sample size required to detect the 150 g mean birthweight difference at 80% power was calculated to be 470, and 543 participants completed the study."
Selective reporting (reporting bias)	Low risk	Comment: All outcomes presented in the methods section were reported in the paper.
Other bias	Low risk	Comment: No other bias was identified

Sabet 2012

**Table:** 

**Methods**	This was a double‐blind randomized controlled trial conducted in Tehran, Iran Dates of study: 2009 to 2010
**Participants**	Pregnant women (n = 50) shceduled to deliver at Mahdieh Hospital were eligible. Exclusion criteria included: pre‐existing sarcoidosis, renal and hepatic dysfunction and tuberculousis.
**Interventions**	Vitamin D group (n = 25): received 100,000 IU of vitamin D 3 times per monthControl group (n = 25): received placebo
**Outcomes**	Secondary outcome reported was maternal serum vitamin D (nmol/L)
**Notes**	**Declarations of interest:** authors declare there is no conflict of interest **Funding sources:** This research project has been supported by a grant from the Research Institute of Endocrine Sciences, Shahid Beheshti University of Medical Sciences.

Risk of bias table

**Table:** 

Bias	Authors' judgement	Support for judgement
Random sequence generation (selection bias)	Unclear risk	Insufficient information to make judgement.
Allocation concealment (selection bias)	Unclear risk	Insufficient information to make judgement.
Blinding of participants and personnel (performance bias)	Low risk	Quote: "double‐blind" Comment: probably done
Blinding of outcome assessment (detection bias)	Unclear risk	Insufficient information to make judgement.
Incomplete outcome data (attrition bias)	Low risk	Comment: 50 women were enrolled and till the end of the study.
Selective reporting (reporting bias)	Low risk	Comment: Outcome presented in the methods sections were reported in the paper or previous studies.
Other bias	Low risk	Comment: No other bias was identified

Sablok 2015

**Table:** 

**Methods**	This was a double‐blind randomized controlled trial conducted in Delhi, India. Dates of study: 2010‐2012
**Participants**	Pregnant women (n = 180) with a singleton pregnancy, primigravidae, and who were between 14 and 20 weeks of gestation were eligible. Women with pre‐existing osteomalacia, known hyperthyroidism, renal and liver dysfunction, tuberculosis or sarcoidosis were excluded from the study.Vitamin D group: (n = 120), placebo group:(n = 60)
**Interventions**	1. Vitamin D group: received vitamin D supplementation in different dosages depending on the participants' levels of serum 25(OH)‐D levels estimated at the start of the study. Women who had sufficient serum levels of vitamin D (>50nmol/L) received one dose of 60,000 IU of vitamin D at 20 weeks. Women who had insuffienct levels of vitamin D (25‐50 nmol/L) received two doses of 120,000IU at 20 weeks and 24 weeks. Participants with deficient levels of vitamin D (<25 nmol/L) were given four doses of 120,000 IU of vitamin D at 20, 24, 28 and 32 weeks.2. Placebo group: received a placebo
**Outcomes**	Outcomes analyzed were serum/plasma Vitamin D concentration (nmol/L), preterm births, and small for gestational age
**Notes**	**Declarations of interest:** authors report no conflicts **Funding sources:** none reported

Risk of bias table

**Table:** 

Bias	Authors' judgement	Support for judgement
Random sequence generation (selection bias)	Low risk	Quote: "Randomization was performed using computer generated random number tables, into two groups"
Allocation concealment (selection bias)	High risk	Comment: one group was assigned no intervention and the other was assigned intervention. The intervention group dosages depended on the serum/plasma vitamin D levels of the participants at the start of the study. Thus there was a selection bias at baseline.
Blinding of participants and personnel (performance bias)	High risk	Comment: The intervention group dosages depended on the serum/plasma vitamin D levels of the participants at the start of the study. Thus there was a selection bias at baseline.
Blinding of outcome assessment (detection bias)	High risk	Comment: The intervention group dosages depended on the serum/plasma vitamin D levels of the participants at the start of the study. Thus there was a selection bias at baseline.
Incomplete outcome data (attrition bias)	Unclear risk	Comment: Insufficient information to make judgement
Selective reporting (reporting bias)	Unclear risk	Comment: Unclear in the study what outcomes they had intended to measure at baseline
Other bias	Unclear risk	Comment: there is a large difference in the size of the control group vs. the intervention group

Sahu et al. 2009

**Table:** 

**Methods**	This was a randomized controlled trial condcuted in Uttar Pradesh in rural India. Dates of study: not reported
**Participants**	139 healthy pregnant women were enrolled in the study. Eligbility criteria included pregnancy of any parity. 84 pregnant women were analyzed after delivery (n = 14 in the control group and n=35 in both intervention groups).All women in all three arms were provided with calcium carbonate (1 g of elemental calcium to be taken in two divided doses with meals) and iron sulfate (as 60 mg of elemental iron per day).
**Interventions**	Participants were randomized into one of three arms: (1) Control group (Group A): did not receive any vitamin D/no treatment (n = 14) (2) Group B: received one bolus dose of 60,000 IU of vitamin D at the 5th month of gestation (n = 35) (3) Group C: received two bolus doses of 120,000 IU of vitamin D at the 5th and 7th month of gestation. (n = 35)
**Outcomes**	The main outcomes reported were serum/plasma biomarkers for vitamin D and calcium.For this review, Group A (control group) was compared to Group C (2 boluses of 120,000 IU of vitamin D) because Group C's daily dosage is most similar to other studies in this comparison group.
**Notes**	**Declarations of interest:** authors report no conflict of interest **Funding Sources:** Grant support to V Bhatia (BT/PR 3552/SPD/11/349/2002 and BT/PR 3552/SPD/11/349/2002 from the Department of Biotechnology, the Government of India

Risk of bias table

**Table:** 

Bias	Authors' judgement	Support for judgement
Random sequence generation (selection bias)	Unclear risk	Insufficient information to provide judgement
Allocation concealment (selection bias)	Unclear risk	Insufficient information to provide judgement
Blinding of participants and personnel (performance bias)	Unclear risk	Insufficient information to provide judgement
Blinding of outcome assessment (detection bias)	Unclear risk	Insufficient information to provide judgement
Incomplete outcome data (attrition bias)	High risk	>20% of the participants were lost to follow‐up at delivery
Selective reporting (reporting bias)	Low risk	All outcomes described in methods were reported
Other bias	Low risk	No other forms of bias detected

Semba et al. 2001

**Table:** 

**Methods**	This was a randomized, double‐blind, controlled trial conducted in Blantyre, Malawi Dates of study: November 1995 to December 1996
**Participants**	Pregnant women (n = 203), between 18 and 28 weeks of gestation, were enrolled in the study. All women received two doses of fansidar during pregnancy as per the guidelines of Malawi's Ministry of Health. All women received daily oral iron and folate from enrollment until delivery.
**Interventions**	Vitamin A group (n = 109): received a supplement containing 30 mg of elemental iron, 400 ug of folate and 3000 retinol equivalents of vitamin A (10,000 IU), to be consumed daily.Placebo group (n = 94): received a supplement containing 30 mg of elemental iron and 400 ug of folate, to be consumed daily.This study was also included in the MMN vs. IFA comparison in this review. The MMN arm received 3 micronutrients (10,000 IU of Vitamin A, 30 mg of elemental iron and 400ug of folate).
**Outcomes**	This study was included in two comparisons in the review: MMN vs. IFA and Vit A vs. placebo1) MMN vs. IFA: outcomes analyzed were maternal serum/plasma retinol (umol/L) and maternal serum/plasma hemoglobin (g/L)2) Vit A vs. placebo: outcomes analyzed were maternal serum/plasma retinol (umol/L) and maternal serum/plasma hemoglobin (g/L)
**Notes**	**Declarations of Interest:** None reported **Funding Sources:** National Institutes of mHealth (HD32247, HD30042, HIVNET contract N01‐AI‐ 35173±117), the Fogarty International Center, and the United States Agency for International Development (Cooperative Agreement HRN‐A‐00±97±00015±00).

Risk of bias table

**Table:** 

Bias	Authors' judgement	Support for judgement
Random sequence generation (selection bias)	Unclear risk	Quote: "After determination of HIV serostatus, HIV‐negative women were randomized…" Comment: unclear; not enough information to make judgement
Allocation concealment (selection bias)	Low risk	Quote: "Treatment assignment was determined using a computer random‐number generator, and treatment assignment was concealed by prepacking study supplements in sequentially numbered series assigned to study identifcation numbers"
Blinding of participants and personnel (performance bias)	Low risk	Quote: "Double‐blind"Comment: Probably done
Blinding of outcome assessment (detection bias)	Low risk	Quote: "Supplements containing vitamin A, iron, and folate were identical in appearance to the supplements containing iron and folate."
Incomplete outcome data (attrition bias)	High risk	Quote: "In the vitamin A and control groups, the follow‐up was 69.7% (76 of 109 women) and 64.9% (61 of 94 women), respectively."Comment: >20% loss to followup
Selective reporting (reporting bias)	High risk	Comment: Certain n values for different outcomes are unclear or missing. Information about other outcomes is unclear.
Other bias	Low risk	Comment: No other bias was identified

Sorouri 2016

**Table:** 

**Methods**	This was a randomized control trial conducted in an urban setting in Rasht (Guilan Province), in Iran. Dates of study: January 2010‐January 2012
**Participants**	Healthy pregnant women (n = 540) at 16 weeks of gestation who presented at the antenatal care clinic, and did not have high‐risk pregnacies were eligible. Exclusion criteria included age under 18, or over 40 years, height <145 cm, body mass index <15 kg/m2 or >30 kg/m2 before pregnancy, smoking, multifetal pregnancy, pregnancies assisted by reproductive technology, uterine anomalies, leiomyoma, and any established medical risk for reduced or excessive birthweight (including, hypertension, renal diseases, diabetes, and other contributing chronic disease). Eligible women also must have had no history of miscarriage, intrauterine death, stillbirth, low birth weight babies in previous pregnancies, premature birth, pre‐eclampsia and/or macrosomia.Zinc group (n = 270), placebo group (n = 270)
**Interventions**	1. Zinc group: received 15 mg of zinc in a tablet to be consumed every other day (given that the zinc tablets could not be cut in half)2. Placebo group: did not receive any tabletWomen in both treatment arms were given tablets of iron and folic acid (30 mg of iron and 400ug of folic acid).
**Outcomes**	This study was included in two comparisons in the review: MMN vs IFA and Zinc vs. Placebo. (1) MMN vs IFA: outcomes analyzed were low birth weight and preterm births. (2) Zinc vs Placebo: outcomes analyzed were low birth weight, preterm births and pre‐eclampsia
**Notes**	No placebo supplement was given. IFA was administered to to both the control and intervention group as part of the treatment. **Declarations of Interest**: authors declare no conflict of interest **Funding sources**: financial support provided by the Research Deputy at the Guilan University of Medical Sciences.

Risk of bias table

**Table:** 

Bias	Authors' judgement	Support for judgement
Random sequence generation (selection bias)	Low risk	Quote: "A computer‐generated randomized list using a size four permuted block was used. Random sequences were prepared by a researcher with no clinical involvement in the trial. A gynecologist from the clinic allocated participants to each group by using random sequences."
Allocation concealment (selection bias)	Low risk	Quote: "A computer‐generated randomized list using a size four permuted block was used. Random sequences were prepared by a researcher with no clinical involvement in the trial. A gynecologist from the clinic allocated participants to each group by using random sequences."
Blinding of participants and personnel (performance bias)	Low risk	Quote: "Outcome assessors and data analysts were kept blinded to the allocation."
Blinding of outcome assessment (detection bias)	Low risk	Quote: "Outcome assessors and data analysts were kept blinded to the allocation."
Incomplete outcome data (attrition bias)	Low risk	Comment: Sixty‐one women were lost to follow‐up because they declined to continue participating. Overall 235/270 reciving zinc, and 244/270 not receiving zinc completed the study.
Selective reporting (reporting bias)	Low risk	Comment: All outcomes presented in the methods section were reported in the paper.
Other bias	Low risk	Comment: No other bias was identified

Summit 2008

**Table:** 

**Methods**	A double‐blind cluster‐randomised trial conducted at Lombok island of Indonesia Dates of study: July 2001‐April 2004
**Participants**	Pregnant women of any gestational age assessed by physical exam and reported LMP
**Interventions**	1. MMN group (n = 15,804)) received iron 30 mg, folic acid 400 mcg, vitamin A 800 mcg, D 200 IU, E 10 mg, C 70 mg, B1 1.4 mg, B6 1.9 mg, B12 2.6 mcg, zinc 15 mg, copper 2 mg, selenium 65 mcg, iodine 150 mcg and niacin 18 mg2. Placebo group (n = 15,486) received iron 30 mg and folic acid 400 mcg
**Outcomes**	Primary outcomes: low birth weight, perinatal mortality, maternal anaemia and maternal mortalitySecondary outcomes: small for gestational age, child serum/plasma hemoglobin, neonatal mortality, miscarriage, preterm births, stillbirthsIt should be noted that the data for small for gestational age were obtained from a separate report (Keats 2019) and not from the individual trial report.
**Notes**	Women in both groups received supplements throughout pregnancy until 90 days postpartum. Intervention and placebo groups were comparable in terms of baseline characteristicsStudy was stopped early due to insufficient funds **Declarations of interest**: the trial authors declared no conflict of interest. **Funding sources**: Turner Foundation, UNICEF, the Centre for Health and Human Development, and the United States Agency for International Development‐Indonesia (grant no 497‐G‐00‐01‐00001‐00)

Risk of bias table

**Table:** 

Bias	Authors' judgement	Support for judgement
Random sequence generation (selection bias)	Low risk	Quote: "Before enrolment, midwife identification nnumbers were sequentially allocated to computer‐generated, randomly permuted blocks of groups numbered one to eight, stratified by community health centre or village health clinic" Comment: probably done
Allocation concealment (selection bias)	Low risk	Quote: "Midwives at village health centres and community health centres were assigned midwife identification numbers" and "Before enrolment, midwife identification nnumbers were sequentially allocated to computer‐generated, randomly permuted blocks of groups numbered one to eight, stratified by community health centre or village health clinic"Comment: Probably done
Blinding of participants and personnel (performance bias)	Low risk	Quote: "All study scientists and personnel, government staff and enrolees were unaware of the allocation" and "The code to indicate which strip was IFA or MMN was known only by themanufacturing production manager and a quality control officer from UNICEF, Copenhagen, neither of whom had any connection to the study or its personnel"Comment: participants and caregivers were probably blinded to the treatment assignment
Blinding of outcome assessment (detection bias)	Low risk	Quote: "All study scientists and personnel, government staff and enrolees were unaware of the allocation" and "The code to indicate which strip was IFA or MMN was known only by themanufacturing production manager and a quality control officer from UNICEF, Copenhagen, neither of whom had any connection to the study or its personnel"Comment: outcome assessors were probably blinded to the treatment assignment
Incomplete outcome data (attrition bias)	Low risk	Exclusion (25.2%) and attrition (5%) were reported along with their reasons
Selective reporting (reporting bias)	Low risk	Comment: all outcomes mentioned in the methods section were presented in the paper
Other bias	Low risk	Comment: no other bias was identified, including cluster‐design specific biases (recruitment bias, baseline imbalance, loss of clusters, incorrect analysis, and comparability with individually randomised trials)

Sunawang 2009

**Table:** 

**Methods**	A cluster‐randomised trial conducted in 2 subdistricts of Indramayu district of west Java province of Indonesia Dates of study: May 2000‐August 2003
**Participants**	Pregnant women irrespective of gestational age. Women suffering from diabetes mellitus, coronary heart disease and tuberculosis were excluded
**Interventions**	1. Intervention group (n = 432) received RDA of 15 micronutrients according to the UNICEF/UNU/WHO recommended formula, including 30 mg of ferrous fumarate2. Control group (n = 411) received ferrous sulphate 60 mg and folic acid 0.25 mg
**Outcomes**	Primary outcomes: perinatal mortality and low birth weightSecondary outcomes: neonatal mortality, small for gestational age, miscarriage, stillbirths, and preterm birthsIt should be noted that the data for SGA were obtained from a separate report (Keats 2019) and not from the individual trial report.
**Notes**	Study groups were similar with respect to baseline characteristics. Supplements were given from the time of enrolment at 12‐20 week's gestation and continued up to 30 days postpartum **Declarations of interest**: not reported **Funding sources**: UNICEF Indonesia Office, Jakarta

Risk of bias table

**Table:** 

Bias	Authors' judgement	Support for judgement
Random sequence generation (selection bias)	Unclear risk	Quote: "We restructured the 157 hamlets into 160 dwelling clusters", "these 160 clusters (and the pregnant women living within them) were randomly assigned into 4 blocks of 40 clusters each" Comment: method used for generating the randomisation sequence is not described in sufficient detail to permit judgement
Allocation concealment (selection bias)	Unclear risk	Comment: method used for allocation concealment is not described in sufficient detail to permit judgement
Blinding of participants and personnel (performance bias)	Unclear risk	Quote: "This study had a single‐blind design, since the supplement for the treatment and control group looked different physically. However, participants residing in each cluster received the same supplement, so they were not aware that other participants from other clusters received a different supplement: Comment: study participants were blinded to the treatment assignment
Blinding of outcome assessment (detection bias)	Unclear risk	Quote: "This study had a single‐blind design"Comment: blinding of outcome assessors probably not done
Incomplete outcome data (attrition bias)	Low risk	Exclusion (<1%) and attrition (10.4%) were reported along with their reasons
Selective reporting (reporting bias)	Low risk	Comment: all outcomes mentioned in the methods section were presented in the paper
Other bias	Low risk	Comment: when considering cluster‐design‐ specific biases, we found low risk of baseline imbalance, loss of clusters, and comparability with individually randomised trials. While participants may have been recruited after assignment of clusters (843 pregnant women enrolled after intensive surveillance), we found that this likely would not have led to recruitment bias because supplements were provided to both groups and pregnant women were similar (including mean gestational age at baseline). Any incorrect analysis was corrected by adjustment for clustering within data reported in this review

Taherian 2002

**Table:** 

**Methods**	This was a randomized controlled trial conducted in Iran. Dates of study: April 1998 to March 2001.
**Participants**	Healthy, nulliparous women (n = 990), <20 weeks of gestation were eligible. Exclusion criteria include hypertension and proteinuria (detected on a dipstick), history of cardiovascular, renal or endocrinologic problems, medical or obstetric complications, and multiple gestation.
**Interventions**	This study had 3 arms: (1) Group 1 received 75 mg of aspirin daily (2) Group 2 received 500 mg of calcium carbonate and 200 IU of vitamin D, daily (3) Group 3 (placebo group) received no medication at all
**Outcomes**	None. Excluded from analysis given insufficient number of studies per outcome for the calcium‐vitamin D vs. placebo comparison.
**Notes**	**Declarations of interest:** none reported **Funding sources:** Research Deputy of IUMS, grant (No: 76085)

Risk of bias table

**Table:** 

Bias	Authors' judgement	Support for judgement
Random sequence generation (selection bias)	Unclear risk	Quote: "The 990 healthy nulliparous women were randomly allocated to three equivalent groups. We used a table of random number to assign each case independently to one of three groups" Comment: insufficient information to make judgement
Allocation concealment (selection bias)	Unclear risk	Quote: "The 990 healthy nulliparous women were randomly allocated to three equivalent groups. We used a table of random number to assign each case independently to one of three groups"Comment: insufficient information to make judgement
Blinding of participants and personnel (performance bias)	Unclear risk	Comment: no mention of blinding; insufficient information to make judgement
Blinding of outcome assessment (detection bias)	Unclear risk	Comment: no mention of blinding; insufficient information to make judgement
Incomplete outcome data (attrition bias)	Unclear risk	Comment: insufficient information to make judgement
Selective reporting (reporting bias)	Low risk	Comment: outcomes mentioned in the methods were reported in the analysis
Other bias	Low risk	Comment: No other bias identified

Tanumihardjo 2002

**Table:** 

**Methods**	This was a randomized controlled trial conducted in the urban setting, Bogot, West Java, in Indonesia. Dates of study: unreported
**Participants**	Pregnant women (n = 27) in their second or early thir trimester, ages 18 to 37 years old with parity of <4 were eligible for the study.Participants were randomly assigned to four arms: Group 1: Placebo (n = 7), Group 2: Vitamin A (n = 7), Group 3: Iron (n = 5), and Group 4: Vitamin A and Iron (n = 8)
**Interventions**	This study has 4 treatment arms: Group 1: Placebo (n = 7), Group 2: Vitamin A (n = 7), Group 3: Iron (n = 5), and Group 4: Vitamin A + Iron (n = 8). In this review, the placebo arm, the Vitamin A‐only arm and the iron‐only arm were included in the analysis.The placebo arm: received a placebo dailyThe Vitamin A‐only arm: received 8.4 umol (8000 IU) of vitamin A daily as retinal palmitate and an iron placebo pillThe Iron‐only arm: received 1.07 mmol (60 mg) of iron (as ferrous sulfate) and a Vitamin A placebo pill, daily
**Outcomes**	This study was included in two comparisons in the review: iron vs. placebo and vitamin A vs. placeboFor Iron vs. placebo, outcomes analyzed were serum/plasma hemolgobin (g/L) and serum/plasma ferritin (ug/L)For Vitamin A vs. placebo, outcomes analyzed were serum/plasma retinol (umol/L) and serum/plasma hemoglobin (g/L)
**Notes**	**Declarations of interest:** none reported **Funding sources:** none reported

Risk of bias table

**Table:** 

Bias	Authors' judgement	Support for judgement
Random sequence generation (selection bias)	Unclear risk	Comment: Random sequence generation was not discussed.
Allocation concealment (selection bias)	Unclear risk	Comment: Allocation concealment was not discussed.
Blinding of participants and personnel (performance bias)	Low risk	Quote: "Subjects and village volunteers (caders) were unaware of group assignment."
Blinding of outcome assessment (detection bias)	Low risk	Comment: Blinding of outcome was not discuessed.
Incomplete outcome data (attrition bias)	Low risk	Comment: All enrolled participants were included in the final analysis.
Selective reporting (reporting bias)	Low risk	Comment: All outcomes presented in the methods section were reported in the paper.
Other bias	Low risk	Comment: No other bias was identified

Tofail et al. 2008

**Table:** 

**Methods**	The study was conducted in Matlab, a rural subdistrict in the east central plain of Bangladesh Dates of study: November 2001‐December 2003
**Participants**	Pregnant women with gestational age 6‐8 weeks, Hb > equal to 80 g/L and no serious disease were eligible for enrolment
**Interventions**	1. MMN group (n = 1224) received vitamin A 800 mcg, D 200 IU, E 10 mg, C 70 mg, B1 1.4 mg, B2 1.4 mg, niacin 18 mg, B6 1.9 mg, B12 2.6 mg, folic acid 400 mcg, iron 30 mg, zinc 15 mg, copper 2 mg, selenium 65 mcg and iodine 150 mcg2. Other group received folic acid and iron (60 mg iron 400 mcg folic acid n = 1265 and 30 mg iron 400 mcg folic acid n = 1248)
**Outcomes**	Primary outcomes analyzed were low birth weight, perinatal mortalitySecondary outcomes analyzed were small for gestational age, child serum/plasma ferritin, serum/plasma folate (maternal), child developmental outcomes, serum/plasma hemoglobin (maternal), child anaemia, neonatal mortality, serum/plasma vitamin B12, infant mortality, serum/plasma zinc, stunting, miscarriage, preterm births, stillbirths, serum/plasma ferritin (maternal), and fever.The MMN group was compared to the IFA group that received 30 mg of Iron and 400 ug of folic acid (n = 1248)It should be noted that the data for small for gestational age were obtained from a separate report (Keats 2019) and not from the individual trial report.
**Notes**	**Declarations of interest:** the trial authors declared no conflict of interest. **Funding sources:** The International Centre for Diarrheal Disease Research Bangladesh, the UK Medical Research Council, the Swedish Research Council, the UK Department for International Development, theGlobal Health Research Fund Japan, the Child Health and Nutrition Research Initiative, Uppsala University, the US Agency for International Development under the Cooperative Agreement #388‐G‐00‐02‐00125‐00, the Australian International Development Agency, the Government of Bangladesh, the Canadian International Development Agency, The Kingdom of Saudi Arabia, the Government of the Netherlands, the Government of Sri Lanka, the Swedish International Development Cooperative Agency, and the Swiss Agency for Development and Cooperation

Risk of bias table

**Table:** 

Bias	Authors' judgement	Support for judgement
Random sequence generation (selection bias)	Unclear risk	Quote: "individual randomisation was done in blocks of 12" and "after enrolment, women were randomly assigned to 6 intervention groups" Comment: method used for generating the randomisation sequence was not described in sufficient detail to permit judgment
Allocation concealment (selection bias)	Unclear risk	Comment: method used for allocation concealment was not described in sufficient detail to permit judgement
Blinding of participants and personnel (performance bias)	Low risk	Quote: "pills were identical in appearance, and monthly supplies were provided in identical bottles", "mothers were unaware of their micronutrient supplement" and "double masking was practiced"Comment: study participants and caregivers were blinded to the treatment assignment
Blinding of outcome assessment (detection bias)	Low risk	Quote: "pills were identical in appearance, and monthly supplies were provided in identical bottles", "mothers were unaware of their micronutrient supplement" and "double masking was practiced"Comment: outcome assessors were blinded to the treatment assignment
Incomplete outcome data (attrition bias)	High risk	Attrition was (26%), reported along with their reasons.
Selective reporting (reporting bias)	Low risk	Comment: all outcomes mentioned in the methods section were presented in the paper
Other bias	Low risk	Comment: no other bias was identified.

Vaziri 2016

**Table:** 

**Methods**	This was a randomized controlled trial conducted in an urban setting in Shiraz, Iran Dates of study: November 2014 ‐ October 2015
**Participants**	Pregnant women (n = 153), both nulliparous and multiparous, aged 18 yeras or older, who were attending prenatal care were eligible, and between 26 and 28 weeks of gestation with a singleton pregnancy were eligible. Exclusion criteria included: history of mental illness, history of chronic or medical illness such as hyper/hypothyroidism, addiction or substance abuse issues, divorced or widowed, pregnancy complications such as pre‐eclampsia, gestational diabetes, ruptured membranes, and suspicion of pre‐term birth, previous history of Caesarean sections. As well, any consumption of <8 weeks of vitamin D supplements was a criterion for exclusion.Vitamin D group (n = 62); placebo group (n = 65) were analyzed
**Interventions**	1. Vitamin D group: received two pills of 1000 IU of Vitamin D per day (for a total of 2000 IU of Vitamin D daily)2. Placebo group: received two placebo pills daily
**Outcomes**	Outcome: serum/plasma vitamin D (nmol/L) and serum/plasma calcium (mg/dL)
**Notes**	**Declarations of interest:** authors have no conflict of interest **Funding Sources:** financially supported by the Office of the Vicechancellor for Research, Shiraz University of Medical Sciences (code: 93‐01‐47‐8436)

Risk of bias table

**Table:** 

Bias	Authors' judgement	Support for judgement
Random sequence generation (selection bias)	Unclear risk	Comment: Random sequence generation was no discussed.
Allocation concealment (selection bias)	Unclear risk	Comment: Allocation concealment was not discussed
Blinding of participants and personnel (performance bias)	Low risk	Quote: "Mothers were not aware of the pill contents. The anthropometric measurements of infants were collected by trained midwives outside the research team who were blind to group allocations."
Blinding of outcome assessment (detection bias)	Low risk	Quote: "Mothers were not aware of the pill contents. The anthropometric measurements of infants were collected by trained midwives outside the research team who were blind to group allocations." Additionally, the lab technicians were blinded to group allocations
Incomplete outcome data (attrition bias)	Low risk	Comment: 127 participants were included in the final analysis, out of 153 recruited. Additionally, no significant difference between participants remaining and those lost to follow‐up.
Selective reporting (reporting bias)	Low risk	Comment: All outcomes presented in the methods section were reported in the paper.
Other bias	Low risk	Comment: No other bias was identified

Villar 2006

**Table:** 

**Methods**	This was a multi‐centered, randomized double‐blind controlled trial that was conducted in antenatal care clinics and hospitals in four countries: India, Peru, South Africa and Vietnam Dates of study: October 2004 to December 2006
**Participants**	Healthy nulliparous women (n = 8325) at <20 weeks gestation were recruited between November 2001 and July 2003. Exclusion critieria included: hypertension (>140/90 mmHg) at first antenatal visit, a history of chronic hypertension or renal disease, history or symptoms of nephrolithiasis, history of parathyroid disorders and any diseases that require digoxin, phenytoin, or tetracycline therapy. Women were enrolled from populations who consumed <600 mg of calcium per day.Calcium group (n = 4157); Placebo group (n = 4168)
**Interventions**	1. Calcium group: received 1.5 g of calcium per day (3 tablets of 500 mg of calcium each)2. Placebo group: received a placebo tablet containing lactose, sorbitol, cellulose and calcium‐free ingredients (1 tablet, 3 times daily to match the dose schedule of the intervention group)Only iron and folic acid were permitted along with the calcium or placebo supplementation
**Outcomes**	Primary outcomes analyzed was low birth weight (<2500 g)Secondary outcomes analyzed were pre‐eclampsia, mode of delivery ‐ Caesarean section and preterm birth
**Notes**	**Declarations of interest:** none reported **Funding sources:** none reported

Risk of bias table

**Table:** 

Bias	Authors' judgement	Support for judgement
Random sequence generation (selection bias)	Low risk	Quote: "Randomization lists for each site were produced with computer‐generated random number blocking…"
Allocation concealment (selection bias)	Low risk	Quote: "Boxes and tablet bottles were prepared and numbered by Magistra SA, Geneva, Switzerland; each box contained 7 sequentially numbered bottles (100 tablets/bottle, an approximate 4‐week supply; maximum, 700 tablets for 33 weeks). Complete sets, all of which were identical, were shipped by WHO to centers and kept in a designated area with a locker."
Blinding of participants and personnel (performance bias)	Low risk	Quote: "Boxes and tablet bottles were prepared and numbered by Magistra SA, Geneva, Switzerland; each box contained 7 sequentially numbered bottles (100 tablets/bottle, an approximate 4‐week supply; maximum, 700 tablets for 33 weeks). Complete sets, all of which were identical, were shipped by WHO to centers and kept in a designated area with a locker."
Blinding of outcome assessment (detection bias)	Low risk	Quote: "Randomization codes remained at the WHO Clinical Trial Unit until the time of analysis and were not available to any person until the analyses were completed."
Incomplete outcome data (attrition bias)	Low risk	Quote: "… 8325 women were assigned randomly to group (4157 women were assigned to the calcium group and 4168 women were assigned to the placebo group)…Thus, 4151 women in the calcium group and 4161 women in the placebo group contributed to the final analyses…"
Selective reporting (reporting bias)	Low risk	Comment: All outcomes presented in the methods section were reported in the paper.
Other bias	Low risk	Comment: No other bias was identified

West et al. 1999

**Table:** 

**Methods**	This was a double‐blind cluster‐randomized controlled trial, conducted in a rural setting in Sarlahi district, Nepal. Dates of study: April 1994 ‐ September 1997
**Participants**	Women of child bearing age, who were married, and became pregnant were eligible for the study. Newly married women were recruited throughout the trial. N=44,646 women were enrolled, of which n=20,119 women became pregnantVitamin A group (n = 15,305); placebo group (n = 14,805)
**Interventions**	1. Vitamin A group: received an opaque, gelatinous capsule containing peanut oil and 23,300 IU of pre‐formed Vitamin A (7000ug retinol equivalents) as retinyl palmitate to be consumed weekly2. Placebo group: received an opaque, gelatinous capsule containing just peanut oilWomen in both treatment arms were given 5 mg of dl‐alpha‐tocopherol as an antioxidant (included in both intervention and placebo capsules)
**Outcomes**	Primary outcome analyzed was maternal mortality, and secondary outcomes analyzed were serum retinol/vitamin A (umol/L) and stillbirths
**Notes**	**Declarations of Interest:** none reported **Funding sources:** The authors received funds to support vitamin A research in the developing world from TaskForce Sight and Life, Roche, Basle.

Risk of bias table

**Table:** 

Bias	Authors' judgement	Support for judgement
Random sequence generation (selection bias)	Low risk	Quote: "All wards were assigned in Kathmandu by a random draw of numbered chits, blocked on subdistrict, for eligible women to receive one of three identical coded supplements."
Allocation concealment (selection bias)	Low risk	Quote: "The study was a double blind, placebo controlled, cluster randomised trial…" Quote: "All wards were assigned in Kathmandu by a random draw of numbered chits [sic], blocked on subdistrict, for eligible women to receive one of three identical coded supplements."Comment: This trial randomized 270 wards at once to one of three supplements.
Blinding of participants and personnel (performance bias)	Low risk	The randomisation code was held by a member of the study team (AP) who was not directly involved with the collection of data in the field or the laboratory and who had no contact with the study participantsComment: Participant and personnel were likely blinded to each treatment, as treatment groups were randomised by ward, and supplements were identical in appearance.
Blinding of outcome assessment (detection bias)	Low risk	Comment: The two doctors who assessed 'proximate' cause of death, were blinded to treatment allocation.
Incomplete outcome data (attrition bias)	Unclear risk	Quote: "Overall, 20 119 (45%) women were pregnant 22 189 times. Maternal survival was known after all pregnancy outcomes, but 157 women were lost to follow up during the postpartum period (their median follow up time post partum was around 2 weeks in each group)."Quote: "Of the 15 987 live born infants, 15 115 (94.5%) were followed through 28 d of age."Comment: Low loss to follow‐up relevant outcomes, including all‐cause pregnancy related mortality, maternal morbidity, fetal loss, and neonatal morality.
Selective reporting (reporting bias)	Low risk	Comment: All outcomes listed in Methods were reported.
Other bias	Low risk	Comment: Other biases not identified.

West et al. 2011

**Table:** 

**Methods**	This was a cluster‐randomized, double‐blinded placeob‐controlled trial conducted in rural northern districts (Gaibandha and Rangpur districts) in Bangladesh Dates of study: August 2001 to January 2007
**Participants**	Married women of reproductive age (ages 13‐45 years) who become pregnant and were confirmed through a positive hCG urine test were eligible. There were 60,296 pregnancies identified through surveillance, of which n=59,666 pregnancies consented to be followed at 12 weeks postpartum).Vitamin A group: n=19,806Placebo group: n=19,826
**Interventions**	1. Vitamin group: received an oil capsule containing 23,300 IU or 7000ug retinol equivalents (as retinyl palmitate) to be consumed weekly.2. Placebo group: received an oil capsule containing only oil.Both treatment arms received 5IU of vitamin E in the capsules to extend shelf life.
**Outcomes**	Primary outcome analyzed was maternal mortalitySecondary outcomes analyzed were stillbirths and serum/plasma retinol (umol/L)
**Notes**	Adherence was assessed by pill count and recall, when consumption of supplement could not be directly observed by fieldworkers each week during delivery of supplementation. If women were not going to be at home, workers would give women enough supplementation for their absence. **Declarations of interest:** authors report no conflicts of interest **Funding sources:** Micronutrients for Health Cooperative Agreement HRN‐A‐00‐97‐00015‐00 and Global Research Activity GHS‐A‐00‐03‐00019‐00 between the Office of Health, Infectious Diseases and Nutrition, US Agency for International Development (USAID) and Johns Hopkins University and Global Control of Micronutrient Deficiency grant 614 from the Bill and Melinda Gates Foundation, and USAID Mission, Dhaka, Bangladesh; the Ministry of Health and Family Welfare, Government of Bangladesh, the Micronutrient Initiative/Canadian International Development Agency, the Nutrilite Health Institute, Access Business Group LLC, and the Sight and Life Research Institute.

Risk of bias table

**Table:** 

Bias	Authors' judgement	Support for judgement
Random sequence generation (selection bias)	Low risk	Comment: Sectors were listed geographically, assigned a number from 001 to 596, and randomized to 1 of 3 codes (1, 2, or 3) by random draw of numbered chip, each representing 1 of the 3 batches of supplements confidentially labeled by the supplement packer and shipper
Allocation concealment (selection bias)	Low risk	Comment: Cluster randomization by geographic location, and coded packaged prepared by manufacturers that were unknown to staff and participants.
Blinding of participants and personnel (performance bias)	Low risk	Comment: Supplements were identical in appearance (in size and color) and investigators, field staff, and data collectors were blinded to codes. Additionally, further masking was done by masking codes with sector numbers on bottles.
Blinding of outcome assessment (detection bias)	Low risk	Comment: Supplements were identical in appearance (in size and color) and investigators, field staff, and data collectors were blinded to codes. Additionally, further masking was done by masking codes with sector numbers on bottles.
Incomplete outcome data (attrition bias)	Low risk	Comment: Out of 40,072 women enrolled in the placebo and vitamin A treatment arms, 39668 of those pregnancies were included in the analysis. Of th 27,627 pregnancies that ended in live births, 27,877 of infants were included in analysis. Overall, attrition was low.
Selective reporting (reporting bias)	Low risk	Comment: All outcomes presented in the methods section were reported in the paper.
Other bias	High risk	Comment: Women were given treatment for 4‐8 weeks with high doses of vitamin A (25,000 IU/week) if they presented nightblindness, this may have biased the outcomes of vitamin A supplementation.

West 2014

**Table:** 

**Methods**	Community‐based, cluster‐randomised, double‐blind trial to examine whether a daily antenatal and postnatal MMN supplement given to women will enhance newborn and infant survival and health and other birth outcomes in a rural setting in northwestern Bangladesh Dates of study: January 2008‐August 2012
**Participants**	Pregnant women, aged 12‐45 years, consenting to participate were recruited (n = 45,000).Women not interviewed for consentwithin 12 consecutiveweeks after being ascertained as pregnant by urine testing were excluded
**Interventions**	1. Dietary supplement: MMN containing 15 micronutrients all at an RDA including: vitamin A (770 ug retinol equivalents, vitamin D (5 ug), vitamin E (15 mg), folic acid (600 ug), thiamin (1.4 mg), riboflavin (1.4 mg), niacin (18 mg), vitamin B‐12 (2.6 mg), vitamin B‐6 (1.9 mg), vitamin C (85 mg), iron (27 mg), zinc (12 mg), iodine (220 ug), copper (1000 ug), selenium (60 ug).2. Control supplement contained iron (27 mg) ‐ folic acid (600 ug) (providing the current standard of care during pregnancy).Mothers instructed to take 1 tablet per day, from the 1st trimester through 12 weeks postpartum
**Outcomes**	Primary outcomes analyzed were low birth weight, maternal anaemia, maternal mortality and perinatal mortalitySecondary outcomes analyzed were small for gestational age, developmental outcomes, neonatal mortality, stunting, miscarriage, preterm births, wasting, underweight (children), and stillbirthsIt should be noted that the data for small for gestational age were obtained from a separate report (Keats 2019) and not from the individual trial report.
**Notes**	**Declarations of interest**: Dr.West reported a grant from DSM awarded to the Program in Human Nutrition at Johns Hopkins Bloomberg School of Public Health and having given 2 scientific presentations in 2 consecutive years at DSM in Basel, Switzerland, with accommodations provided. Dr. Christian reported giving a presentation at DSM in Basel with accommodations provided. The other trial authors declared no conflict of interest **Funding sources**: grant OPP614 (Global Controlof Micronutrient Deficiency) from the Bill and Melinda Gates Foundation and additional assistance was received from the Sight and Life Global Nutrition Research Institute

Risk of bias table

**Table:** 

Bias	Authors' judgement	Support for judgement
Random sequence generation (selection bias)	Low risk	Quote: "We used an in‐house program (VBScript, Microsoft) that recognized 70 possible permutations for n=8 sectors and k=2 supplement allocations and 6 for the last block of n=4 sectors. Using this program, we randomized sectors within blocks to 1 of 2 codes such that each permutation had an equal probability of being chosen"
Allocation concealment (selection bias)	Low risk	Quote: "The resulting 2 lists of sectors were securely transmitted to field headquarters. One envelope with the code key was securely transmitted to the supplement producer and the other sealed in an envelope and secured at Johns Hopkins. At no time during the trial did study investigators or field or data management staff have access to the key."
Blinding of participants and personnel (performance bias)	Low risk	Quote: "double‐masked", "double blind (subject, caregiver, investigator, outcome assessor)", "received daily supplementation, so treatment effect (still blinded due to the ongoing trial)" Comment: probably done
Blinding of outcome assessment (detection bias)	Low risk	Quote: "double‐masked", "double blind (subject, caregiver, investigator, outcome assessor)", "received daily supplementation, so treatment effect (still blinded due to the ongoing trial)"Comment: probably done
Incomplete outcome data (attrition bias)	Low risk	Complete information was not available as the main trial has not been published; however, attrition is reported to be <20% (trial presentations)
Selective reporting (reporting bias)	Low risk	Comment: reports from the study are still being published
Other bias	Low risk	Comment: no other bias was identified, including cluster‐design specific biases (recruitment bias, baseline imbalance, loss of clusters, incorrect analysis, and comparability with individually randomised trials)

Zagré 2007

**Table:** 

**Methods**	This study was a cluster‐randomised, double‐blind controlled programmatic study in rural Niger aiming to compare MMN supplementation versus iron and folic acid Dates of study: not reported
**Participants**	Women residing in target villages and experiencing amenorrhoea for <12 weeks were eligible for recruitment. All villages within the coverage of the 17 health centres of Mayahi district were included.Women with night blindness and/or signs of severe anaemia were excluded
**Interventions**	1. MMN group (n = 1893) received vitamin A 800 mcg, D 200 IU, E 10 mg, C 70 mg, B1 1.4 mg, B2 1.4 mg, B3 18 mg, B6 1.9 mg, B12 2.6 mg, folic acid 400 mcg, iron 30 mg, zinc 15 mg, copper 2 mg, selenium 65 mcg, iodine 150 mcg.2. Control (n = 1777) received IFA (60 mg Fe, 400 ug FA)
**Outcomes**	Primary outcomes analyzed were low birth weight, maternal mortalitySecondary outcomes analyzed were small for gestational age, miscarriage, preterm births, stillbirthsIt should be noted that the data for small for gestational age were obtained from a separate report (Keats 2019) and not from the individual trial report.
**Notes**	Study participants received reproductive health services including malaria chemoprophylaxis, behaviour‐change communication activities to increase awareness and adoption of better lifestyles (feeding and rest during pregnancy). Outreach prenatal care sessions were also conducted throughout intervention villagesRandomisation resulted in comparable groups for most baseline characteristics except for households and more preventive measures against malaria (more in MMN group) and less education and more povert in IFA group **Declarations of interest:** not reported **Funding sources**: UNICEF

Risk of bias table

**Table:** 

Bias	Authors' judgement	Support for judgement
Random sequence generation (selection bias)	Unclear risk	Quote: "Villages ‐ not individuals were randomly assigned to one treatment group or the other" Comment: method used for generating the randomisation sequence was not described in sufficient detail to permit judgement
Allocation concealment (selection bias)	Unclear risk	Comment: method used for allocation concealment was not described to permit judgement
Blinding of participants and personnel (performance bias)	Low risk	Quote: "Because the two supplements did not look identical and may have been recognizable, a coding system was put in place by the SONIPHAR pharmaceutical company in Niger. Six codes were assigned to the treatments: three for iron/folic acid and three for multimicronutrient supplements. SONIPHAR packaged the supplements in boxes with identical labelling except for the supplement code. Health workers, traditional midwives, and data collectors were informed that each supplement came in two sizes and colors, so that the code letter did not distinguish which supplement was used"Comment: participants and caregivers were probably blinded to the treatment assignment
Blinding of outcome assessment (detection bias)	Low risk	Quote: "Because the two supplements did not look identical and may have been recognizable, a coding system was put in place by the SONIPHAR pharmaceutical company in Niger. Six codes were assigned to the treatments: three for iron/folic acid and three for multimicronutrient supplements. SONIPHAR packaged the supplements in boxes with identical labelling except for the supplement code. Health workers, traditional midwives, and data collectors were informed that each supplement came in two sizes and colors, so that the code letter did not distinguish which supplement was used"Comment: outcome assessors were probably blinded to the treatment assignment
Incomplete outcome data (attrition bias)	Unclear risk	Attrition was 18%. Reasons for attrition were reported, and dropout was significantly higher in the MMN (25/1893 (1.3%)) versus IFA (8/1777 (0.5%)) group. Exclusion data were not reported
Selective reporting (reporting bias)	Low risk	Comment: all outcomes mentioned in the methods section were presented in the paper
Other bias	Low risk	Comment: no other bias was identified, including cluster‐design‐specific biases (recruitment bias, baseline imbalance, loss of clusters, and comparability with individually randomised trials). Any incorrect analysis was corrected by adjustment for clustering within data reported in this review

Zeng et al. 2008

**Table:** 

**Methods**	Community‐based cluster‐randomised trial conducted in 2 poor rural counties in Shaanxi province of north west China Dates of study: August 2002‐Feburary 2006
**Participants**	Pregnant women of <28 weeks gestation between August 2002 and January 2006.Pregnancy was confirmed using LMP and urine pregnancy test
**Interventions**	1. IFA group (n = 1912) received iron 60 mg and folic acid 0.4 mg2. MMN group (n = 1899) received iron 30 mg, folic acid 0.4 mg, zinc 15 mg, copper 2 mg, selenium 0.65 mg, iodine 0.15 mg, vitamin A 0.8 mg, B1 1.4 mg, B2 1.4 mg, B6 1.9, B12 0.026 mg, D 0.05 mg, C 70 mg, E 10 mg, niacin 18 mg3. Folic acid only group A (n = 2017) received folic acid 0.4 mg
**Outcomes**	This study was included in three comparison in this review: IFA vs. FA and Iron vs. Placebo (Group 1 and Group 3) and MMN vs. IFA (Group 2 and Group 1)Primary outcomes for IFA vs. FA/iron vs. placebo: perinatal mortality, low birth weight, maternal anaemiaSecondary outcomes for IFA vs FA/iron vs. placebo: preterm births, neonatal mortality and serum/plasma hemoglobinPrimary outcomes for MMN vs. IFA: low birth weight, perinatal mortality, maternal anaemiaSecondary outcomes for MMN vs. IFA: small for gestational age, serum/plasma hemoglobin, child developmental outcomes, neonatal mortality, stunting, miscarriage, preterm births, wasting, underweight (children) and stillbirthsIt should be noted that the data for SGA were obtained from a separate report (Keats 2019) and not from the individual trial report.
**Notes**	For review purposes, we used the MMN and IFA groups. Intervention was administered until 6 weeks postpartum. Baseline characteristics at enrolment, and both cluster‐ and individual‐level baseline characteristics were balanced by treatment groups. Stunting, underweight, and wasting data presented as odds ratio and could not be included in the analysis **Declarations of interest:** MJD was consultant for UNICEFChinaUNICEF Pyongyang during the conduct of the trial. SC was nutrition consultant for UNICEF China from 2001‐2002, and is now the liaison officer for UNICEF with the Ministry of Health **Funding sources**: United Nations Children's Fund (grant No YH101‐H12/03) through a co‐operative agreement between UNICEF and the Centers for Disease Control and Prevention, Atlanta, US, and the National Natural Science of Foundation of China (grant No 30271131), Beijing, China

Risk of bias table

**Table:** 

Bias	Authors' judgement	Support for judgement
Random sequence generation (selection bias)	Low risk	Quote: "The randomisation schedule was generated off site with a pseudo‐random number generator in SAS" Comment: probably done
Allocation concealment (selection bias)	Low risk	Quote: "The randomisation schedule was generated off site with a pseudo‐random number generator in SAS version 6 (SAS Institute, Cary, NC). A treatment colour code was assigned to each village based on the treatment allocation schedule"Comment: probably done
Blinding of participants and personnel (performance bias)	Low risk	Quote: "double blind", "treatment code was assigned to each village based on the treatment allocation schedule. The treatment codes were opened only once all data had been collected and blinded analysis of the primary hypothesis was completed" and "were of identical appearance and packaged in blister packs"Comment: participants and caregivers were blinded to the treatment assignment
Blinding of outcome assessment (detection bias)	Low risk	Quote: "double blind", "treatment code was assigned to each village based on the treatment allocation schedule. The treatment codes were opened only once all data had been collected and blinded analysis of the primary hypothesis was completed"Comment: outcome assessors were blinded to the treatment assignment
Incomplete outcome data (attrition bias)	Low risk	Exclusion (4.8%) and attrition (2.3%) were reported along with their reasons
Selective reporting (reporting bias)	Low risk	Comment: all outcomes mentioned in the methods section were presented in the paper
Other bias	Low risk	Comment: no other bias was identified, including cluster‐design‐specific biases (recruitment bias, baseline imbalance, loss of clusters, and comparability with individually randomised trials). Investigators did not adjust for the cluster‐randomised design in their sample size or outcome estimations, but this was corrected

Zhao 2015

**Table:** 

**Methods**	This was a parallel assignment, randomised‐controlled trial that was conducted in rural Hebei, China. Dates of study: 2009 to 2011
**Participants**	Pregnant women (n = 2371) with uncomplicated, singleton pregnancies, and were less than or equal to 20 weeks of gestation at their first Sanhe MCHC visit, with plans to give birth at a participating hospital, were eligible. Exclusion criteria included age <18 years, not living in Sanhe, not mentally competent, history or establishment of a chronic health problem, hemoglobin <100 g/L or consumption of any medicinal iron for any duration.Iron‐folic acid group (n = 1185); Folic acid group (n = 1186)
**Interventions**	This study was included in two comparisons in the review: IFA vs FA and Iron vs. Placebo 1. Iron‐folic acid group/Iron Group: received a capsule containing 60 mg of elemental iron (as ferrous sulfate) and 400ug of folic acid. Folic acid was given as a separate capsule. 2. Folic acid group/Placebo group: received a placebo capsule and 400ug of folic acid as a separate capsule.
**Outcomes**	For Iron vs. Placebo comparison, the primary outcome analyzed was maternal anaemia. Secondary outcomes analyzed were serum/plasma hemoglobin (g/L), serum/plasma Tfr, serum/plasma ferritin, and iron deficiency (ferritin).For the IFA vs. FA comparison, the primary outcome analyzed was maternal anaemia and secondary outcomes analyzed were serum/plasma hemoglobin (g/L), serum/plasma Tfr, serum/plasma ferritin
**Notes**	**Declarations of interest:** authors declared no conflict of interest **Funding sources:** grant from Vifor Pharma Ltd. (GZ, Principal Investigator)

Risk of bias table

**Table:** 

Bias	Authors' judgement	Support for judgement
Random sequence generation (selection bias)	Low risk	Quote: "Taiyuan Satellite Pharmaceutical Co., Ltd. prepared equal quantities of iron or placebo capsules and folate capsules and assigned 4‐digit package numbers beginning with 0001 according to a random‐number chart prepared by a statistician who was not part of the study."
Allocation concealment (selection bias)	Low risk	Quote: "Taiyuan Satellite Pharmaceutical Co., Ltd. prepared equal quantities of iron or placebo capsules and folate capsules and assigned 4‐digit package numbers beginning with 0001 according to a random‐number chart prepared by a statistician who was not part of the study." Quote: "The code was not broken until the study and primary analyses were completed."
Blinding of participants and personnel (performance bias)	Low risk	Quote: "Study participants, personnel, and investigators were unaware of supplement group. The code was not broken until the study and primary analyses were completed."Quote: "Supplement packs, differing in appearance only in the number…"
Blinding of outcome assessment (detection bias)	Low risk	Quote: "Study participants, personnel, and investigators were unaware of supplement group. The code was not broken until the study and primary analyses were completed."
Incomplete outcome data (attrition bias)	High risk	Quote: "Attrition affected the groups similarly both in proportion [31.8% (377 of 1186) in the placebo/folate group and 30.5% (362 of 1185) in the iron/folate group] and reason"Comment: Complete details of those excluded and lost to follow‐up with reason were described. Timing of supplementation (for each follow‐up cycle) was also taken into account in the analytical methods. However, the number of withdrawls were high, despite being balanced between treatment groups.
Selective reporting (reporting bias)	High risk	Comment: Trial is registered as NCT02221752. Majority of outcomes listed in methods were reported. Outcomes that were not statisically significant were not mentioned, including scores for neurological integrity (Infant Neurological Internal Battery; INFANIB) and examiners' rating (Behaviour Rating Scale) on motor quality.
Other bias	Low risk	Comment: Other bias not identified.

Ziaei et al., 2007

**Table:** 

**Methods**	This was a randomized double‐blind controlled trial conducted in Iran. Dates of study: not reported
**Participants**	750 healthy pregnant women with a hemoglobin concentration of > or equal to 13.2 g/dL, and in the early stage of the second trimester, with a body mass index between 19.8 and 26 kg/m2, singleton pregnancy, age between 17 and 35 years, non smoking, no diseases related to polycythemica like asthma and chronic hypertension and no history of threatened abortion in the present pregnancy were enrolled in the study.Iron group (n = 375) and placebo group (n = 375)
**Interventions**	IFA group/Iron group: received a tablet containing 50 mg of elemental iron dailyFA group/placebo group: received a placebo tablet dailyAll participants in both arms received 1 mg of folic acid daily and were not permitted to consume other preparations with vitamins or minerals.
**Outcomes**	This study was included in two comparisons in the review: IFA vs FA and Iron vs. placebo (1) IFA vs FA: Primary outcome analyzed was perinatal mortality and secondary outcomes analyzed were preterm births, serum/plasma hemoglobin (g/L) and small for gestational age. (2) Iron vs. placebo: Primary outcome analyzed was perinatal mortality and secondary outcomes analyzed were preterm births, serum/plasma hemoglobin (g/L) small for gestational age, and pre‐eclampsia/eclampsia.
**Notes**	**Declarations of interest:** none reported **Funding Sources:** none reported

Risk of bias table

**Table:** 

Bias	Authors' judgement	Support for judgement
Random sequence generation (selection bias)	Low risk	Quote: "The method of simple randomisation was used employing the ordinary tables of random numbers. The 125 random numbers were allocated to each of six clinical centres. First 63 random numbers between 1 and 125 determined the case group whose women were to receive iron supplementation. The remaining 62 random numbers between 1 and 125 determined the placebo group."
Allocation concealment (selection bias)	Unclear risk	Comment: insufficient information to provide judgement
Blinding of participants and personnel (performance bias)	Low risk	Quote: "Both ferrous sulphate and placebo were packed in similar wrapping containing a code which was known only to the principle investigator…The study was doubleblind, and the contents were known neither to the women nor to the health centre which administered them."
Blinding of outcome assessment (detection bias)	Low risk	Quote: "Both ferrous sulphate and placebowere packed in similar wrapping containing a code which was known only to the principle investigator."
Incomplete outcome data (attrition bias)	Low risk	Comment: <20% loss to follow‐up
Selective reporting (reporting bias)	Low risk	Comment: outcomes mentioned in methods were reported in the analysis
Other bias	Low risk	Comment: no other bias identified

Ziaei et al., 2008

**Table:** 

**Methods**	This was a double‐blind, placebo‐controlled randomized trial conducted in Tehran, Iran. Dates of study: March 2005‐August 2006
**Participants**	244 healthy pregnant womenwho were all between 13 and 18 weeks of gestation, with a hemoglobin concentration >13.2 g/dL, or greater and normal serum ferritin levels (> or equal to 15ug/L), were enrolled in the study. Other inclusion criteria include ages between 17 and 35 yeras, having a body mass index between 19.8 and 26 kg/m2 and having a singleton pregnancy. Exclusion criteria include smoking, having a disease related to polycythemia (such as asthma or chronic hypertension), and having a history of threatened abortion in the present pregnancy.Iron group (n = 122) and placebo group (n = 122)
**Interventions**	Iron group received a tablet containing 50 mg of elemental iron dailyPlacebo group received a placebo tablet.Both groups received their respective tablets from the 20th week of pregnancy until the end of pregnancy.
**Outcomes**	Outcomes analyzed were: serum/plasma hemoglobin (g/L) and serum/plasma ferritin (ug/L)
**Notes**	Due to ethical concerns and the expectation that iron supplementation was excpted to be necessary after delivery and during breastfeeding period, all women received 50 mg of elemental iron daily for 6 weeks following delivery. **Declarations of interest:** none reported **Funding sources:** none reported

Risk of bias table

**Table:** 

Bias	Authors' judgement	Support for judgement
Random sequence generation (selection bias)	Low risk	Quote: "simple randomization from a table of random numbers was used to assign the women"
Allocation concealment (selection bias)	Low risk	Quote: "…and the wrapping contained a code known only to the principal investigator.
Blinding of participants and personnel (performance bias)	Low risk	Quote: "double‐blind" and "Both ferrous sulfate and placebo were packed in an identical manner, and the wrapping contained a code known only to the principal investigator.
Blinding of outcome assessment (detection bias)	Low risk	Quote: "Both ferrous sulfate and placebo were packed in an identical manner"
Incomplete outcome data (attrition bias)	Low risk	Comment: <20% loss to follow‐up
Selective reporting (reporting bias)	Low risk	Comment: outcomes mentioned in methods were reported in the analysis
Other bias	Low risk	Comment: no other bias identified

#### Characteristics of excluded studies

**Table:** 

Abu‐Saad and Fraser 2010	
**Reason for exclusion**	Wrong Study Design
Abu‐Saad and Fraser 2010a	
**Reason for exclusion**	Wrong Study Design
Adams 2010	
**Reason for exclusion**	Wrong Patient Population
Adamson 2004	
**Reason for exclusion**	Wrong Study Design
Adu‐Afarwuah 2018	
**Reason for exclusion**	Wrong Outcome
Ahmed et al., 2010	
**Reason for exclusion**	Wrong Patient Population
Ahmed et al., 2010a	
**Reason for exclusion**	Wrong Patient Population
Alaoddolehei 2015	
**Reason for exclusion**	Wrong Study Design
Allen 2001	
**Reason for exclusion**	Wrong Study Design
Allen and Gillespie 2001a	
**Reason for exclusion**	Wrong Study Design
Alvarez‐Pedrerol et al., 2010	
**Reason for exclusion**	Wrong Intervention
Alwan et al., 2010	
**Reason for exclusion**	Wrong Patient Population
Alwan et al., 2010a	
**Reason for exclusion**	Wrong Patient Population
Alwan et al., 2010b	
**Reason for exclusion**	Wrong Patient Population
Alwan 2014	
**Reason for exclusion**	Wrong Patient Population
Anzaku 2013	
**Reason for exclusion**	Wrong Intervention
Asadi et al., 2014	
**Reason for exclusion**	Wrong Outcome
Asemi 2012	
**Reason for exclusion**	Wrong outcome
Asemi et al., 2014	
**Reason for exclusion**	Wrong Intervention
Azizi and Smyth 2009	
**Reason for exclusion**	Wrong Intervention
Baltussen et al., 2004	
**Reason for exclusion**	Wrong Study Design
Bánhidy et al., 2011	
**Reason for exclusion**	Wrong Patient Population
Bassaw 1998	
**Reason for exclusion**	Wrong Patient Population
Bates 2003	
**Reason for exclusion**	Wrong Study Design
Bath et al., 2013	
**Reason for exclusion**	Wrong Patient Population
Behjat Sasan et al., 2017	
**Reason for exclusion**	Wrong Patient Population
Bennet et al., 2007	
**Reason for exclusion**	Wrong Intervention
Bentum 2011	
**Reason for exclusion**	Wrong Study Design
Betts et al., 2014	
**Reason for exclusion**	Wrong Patient Population
Bhandari and Banjara 2014	
**Reason for exclusion**	Wrong Intervention
Bhandari and Banjara 2015	
**Reason for exclusion**	Wrong Intervention
Bielderman et al., 2015	
**Reason for exclusion**	Wrong Study Design
Billah et al., 2017	
**Reason for exclusion**	Wrong Study Design
Botto et al., 1996	
**Reason for exclusion**	Wrong Study Design
Botto et al., 2000	
**Reason for exclusion**	Wrong Study Design
Campbell et al., 2010	
**Reason for exclusion**	Wrong Study Design
Casely‐Hayford et al., 2010	
**Reason for exclusion**	Wrong Outcome
Caulfield et al., 1998	
**Reason for exclusion**	Wrong Study Design
Ceesay et al., 1997	
**Reason for exclusion**	Wrong Patient Population
Ceesay et al., 1997a	
**Reason for exclusion**	Wrong Patient Population
Ceesay et al., 1997b	
**Reason for exclusion**	Wrong Patient Population
Chan et al., 2003	
**Reason for exclusion**	Wrong Patient Population
Chaparro and Dewey 2010	
**Reason for exclusion**	Wrong Patient Population
Chappell et al., 1999	
**Reason for exclusion**	Wrong Patient Population
Christian 1998	
**Reason for exclusion**	Wrong Study Design
Christian 2001	
**Reason for exclusion**	Wrong Patient Population
Christian 2001a	
**Reason for exclusion**	Wrong Outcome
Cogswell et al., 2003	
**Reason for exclusion**	Wrong Setting
Coles et al., 2015	
**Reason for exclusion**	Wrong Outcome
Cong et al., 1995	
**Reason for exclusion**	Unable to Access Article
Coobs et al., 2014	
**Reason for exclusion**	Wrong Patient Population
Crowther et al., 1999	
**Reason for exclusion**	Wrong Setting
Czeizel et al., 1996	
**Reason for exclusion**	Wrong Patient Population
Czeizel et al., 1996a	
**Reason for exclusion**	Wrong Outcome
Czeizel et al., 1996b	
**Reason for exclusion**	Wrong Study Design
Czeizel 1998	
**Reason for exclusion**	Wrong Study Design
Czeizel 2003	
**Reason for exclusion**	Wrong Study Design
Czeizel 2011	
**Reason for exclusion**	Wrong Study Design
Daggu and Lau 2015	
**Reason for exclusion**	Wrong Study Design
Dalmiya et al., 2009	
**Reason for exclusion**	Wrong Study Design
Danesh 2010	
**Reason for exclusion**	wrong patient population
Darmstadt et al., 2010	
**Reason for exclusion**	Unable to Access Article
Darnton‐Hill 2015	
**Reason for exclusion**	Wrong Study Design
Darnton‐Hill and Mkparu 2015a	
**Reason for exclusion**	Wrong Study Design
Darnton‐Hill and Mkparu 2015b	
**Reason for exclusion**	Wrong Study Design
Darnton‐Hill and Mkparu 2015c	
**Reason for exclusion**	Wrong Study Design
da Silva Lopes et al., 2017	
**Reason for exclusion**	Wrong Study Design
Dawodu et al., 2013	
**Reason for exclusion**	wrong comparison
De CB, 1989	
**Reason for exclusion**	Unable to Access Article
Delange 1996	
**Reason for exclusion**	Wrong Study Design
Delange 2007	
**Reason for exclusion**	Wrong Study Design
De‐Regil et al., 2012	
**Reason for exclusion**	Wrong Study Design
De‐Regil 2016	
**Reason for exclusion**	Wrong Study Design
De‐Regil 2016a	
**Reason for exclusion**	Wrong Study Design
Díaz et al., 2014	
**Reason for exclusion**	Wrong Intervention
Dreyfuss et al., 1997	
**Reason for exclusion**	Unable to Access Article
Dudas et al., 1995	
**Reason for exclusion**	Wrong Outcome
Duncalf et al., 2014	
**Reason for exclusion**	Wrong Intervention
Egri et al., 2009	
**Reason for exclusion**	Wrong Intervention
Ekström et al., 1996	
**Reason for exclusion**	Wrong Outcome
Eskeland et al., 1997	
**Reason for exclusion**	Wrong Patient Population
Etemadifar and Janghorbani 2015	
**Reason for exclusion**	Wrong Patient Population
Fall et al., 2003	
**Reason for exclusion**	Wrong Study Design
FAO 2006	
**Reason for exclusion**	Wrong Study Design
Finan et al., 2014	
**Reason for exclusion**	Wrong Setting
Franik et al., 2018	
**Reason for exclusion**	Wrong Study Design
Frankenberger et al., 2014	
**Reason for exclusion**	Wrong Patient Population
Frith 2015	
**Reason for exclusion**	Wrong Intervention
Fuse et al., 2011	
**Reason for exclusion**	Wrong Patient Population
Gardner et al., 2015	
**Reason for exclusion**	Wrong Patient Population
Genequand et al., 2014	
**Reason for exclusion**	Wrong Intervention
Genequand et al., 2015	
**Reason for exclusion**	Wrong Intervention
Genequand et al., 2016	
**Reason for exclusion**	Wrong Intervention
Gernand 2016	
**Reason for exclusion**	Wrong Study Design
Gernand et al., 2016a	
**Reason for exclusion**	Wrong Study Design
Glinoer 2006	
**Reason for exclusion**	Wrong Study Design
Glinoer 2007	
**Reason for exclusion**	Wrong Study Design
Goldenberg 1995	
**Reason for exclusion**	Wrong Patient Population
Gonzalez‐Casanova et al., 2017	
**Reason for exclusion**	Wrong Outcome
Gopalan and Tamber 2003	
**Reason for exclusion**	Wrong Patient Population
Grant 2013	
**Reason for exclusion**	Wrong Patient Population
Greiner 2011a	
**Reason for exclusion**	Wrong Study Design
Greiner 2011b	
**Reason for exclusion**	Wrong Study Design
Gupta et al., 2007	
**Reason for exclusion**	Wrong Patient Population
Haider et al., 2011	
**Reason for exclusion**	Wrong Study Design
Haider et al., 2011a	
**Reason for exclusion**	Wrong Study Design
Haider et al., 2011b	
**Reason for exclusion**	Wrong Study Design
Haider 2017	
**Reason for exclusion**	Wrong Study Design
Hamadani et al., 2001	
**Reason for exclusion**	Wrong Patient Population
Hambidge et al., 2014	
**Reason for exclusion**	Wrong Patient Population
Harvey 2017	
**Reason for exclusion**	Wrong Study Design
Hashemipour et al., 2013	
**Reason for exclusion**	Wrong comparison
Hernandez et al., 2008	
**Reason for exclusion**	Wrong Study Design
Herrera et al., 1998	
**Reason for exclusion**	Wrong Patient Population
Hess et al., 2001	
**Reason for exclusion**	Wrong Intervention
Hess et al., 2018	
**Reason for exclusion**	Wrong Intervention
Hininger et al., 2004	
**Reason for exclusion**	Wrong Patient Population
Hoa et al., 2005	
**Reason for exclusion**	Wrong Intervention
Hofmeyr et al., 2010	
**Reason for exclusion**	Wrong Study Design
Holden 2007	
**Reason for exclusion**	Wrong Study Design
Hollis et al., 2011	
**Reason for exclusion**	Wrong Patient Population
Hoogendoorn et al., 2014	
**Reason for exclusion**	Wrong Intervention
Horton et al., 2008	
**Reason for exclusion**	Wrong Patient Population
Huffman et al., 1999	
**Reason for exclusion**	Wrong Patient Population
Huffman and Schofield 2011	
**Reason for exclusion**	Wrong Patient Population
Hyder et al., 2002	
**Reason for exclusion**	Wrong Outcome
Hyder et al., 2008	
**Reason for exclusion**	Wrong Patient Population
Hynes et al., 2012	
**Reason for exclusion**	Wrong Study Design
Ikeogu 2018	
**Reason for exclusion**	Wrong Study Design
Imdad and Bhutta 2011	
**Reason for exclusion**	Wrong Patient Population
Imdad and Bhutta 2012	
**Reason for exclusion**	Wrong Study Design
Imdad and Bhutta 2012a	
**Reason for exclusion**	Wrong Study Design
Imdad and Bhutta 2012b	
**Reason for exclusion**	Wrong Study Design
Imhoff‐Kunsch et al., 2010	
**Reason for exclusion**	Wrong Intervention
Janakiraman et al., 2003	
**Reason for exclusion**	Wrong Outcome
Kalra et al., 2012	
**Reason for exclusion**	Wrong Comparison
Katz 2001	
**Reason for exclusion**	Wrong Comparison
Katz et al., 2002	
**Reason for exclusion**	Wrong Comparison
Khambalia et al., 2009	
**Reason for exclusion**	Wrong Intervention
Khorshid et al., 2014	
**Reason for exclusion**	Wrong Comparison
Kolsteren and De Souza 2001	
**Reason for exclusion**	Wrong Study Design
Krauss‐Etschmann et al., 2017	
**Reason for exclusion**	Wrong Patient Population
Kreysler et al., 2009	
**Reason for exclusion**	Wrong Intervention
Kung'u et al., 2018	
**Reason for exclusion**	Wrong Outcome
Lanou 2013	
**Reason for exclusion**	Wrong Study Design
Larocque et al., 2006	
**Reason for exclusion**	Wrong Intervention
Lee 2013	
**Reason for exclusion**	Wrong Study Design
Leguéné et al., 2014	
**Reason for exclusion**	Wrong Intervention
Leguéné et al., 2016	
**Reason for exclusion**	Wrong Intervention
Leguéné et al., 2017	
**Reason for exclusion**	Wrong Patient Population
Leung et al., 2011	
**Reason for exclusion**	Wrong Patient Population
Li 1995	
**Reason for exclusion**	Wrong Patient Population
Li 2000	
**Reason for exclusion**	Wrong Intervention
Li 2000a	
**Reason for exclusion**	Wrong Patient Population
Li 2013	
**Reason for exclusion**	Wrong Intervention
Li 2014	
**Reason for exclusion**	Wrong Intervention
Limbert et al., 2010	
**Reason for exclusion**	Wrong Patient Population
Ma et al., 2010	
**Reason for exclusion**	Wrong Patient Population
Makola et al., 2003	
**Reason for exclusion**	Wrong Patient Population
Manafi et al., 2008	
**Reason for exclusion**	Unable to Access Article
Mangani 2015	
**Reason for exclusion**	Wrong Patient Population
Mannar and Bohac 2009	
**Reason for exclusion**	Wrong Intervention
Martinez et al., 2018	
**Reason for exclusion**	Wrong Intervention
Mason et al., 2001	
**Reason for exclusion**	Wrong Intervention
Matias et al., 2018	
**Reason for exclusion**	Wrong Outcome
McLaren 2008	
**Reason for exclusion**	Wrong Study Design
Meier et al., 2003	
**Reason for exclusion**	Wrong Patient Population
Menon et al., 2018	
**Reason for exclusion**	Wrong Study Design
Menon et al., 2018a	
**Reason for exclusion**	Wrong Study Design
Merschrod et al., 2014	
**Reason for exclusion**	Wrong Patient Population
Mid‐Term Evaluation of Project India ‐ 2206.06	
**Reason for exclusion**	Wrong Patient Population
Millward 2017	
**Reason for exclusion**	Wrong Study Design
Milman 1997	
**Reason for exclusion**	Wrong Patient Population
Mohd 2017	
**Reason for exclusion**	Wrong Patient Population
Mojibian et al., 2015	
**Reason for exclusion**	Wrong comparison
Morley et al., 2004	
**Reason for exclusion**	Wrong Intervention
Mridha et al., 2017	
**Reason for exclusion**	Wrong Comparison
Mumtaz et al., 2000	
**Reason for exclusion**	Wrong Patient Population
Munters 2017	
**Reason for exclusion**	Wrong Study Design
Muslimatun 2001a	
**Reason for exclusion**	Wrong Outcome
Muss 2011	
**Reason for exclusion**	Wrong Study Design
Mutlu et al., 2014	
**Reason for exclusion**	Wrong comparison
Mwangi et al., 2015	
**Reason for exclusion**	Wrong Intervention
Myers et al., 2001	
**Reason for exclusion**	Wrong Outcome
Naem et al., 2014	
**Reason for exclusion**	Wrong Patient Population
Nair et al., 2016	
**Reason for exclusion**	Wrong Intervention
Neufeld 2007	
**Reason for exclusion**	Wrong Study Design
Nguyen et al., 2009	
**Reason for exclusion**	Wrong Patient Population
Nguyen et al., 2012	
**Reason for exclusion**	Wrong Study Design
Nguyen et al., 2017	
**Reason for exclusion**	Wrong Intervention
Niromanesh et al., 2001	
**Reason for exclusion**	Wrong Patient Population
Nwankpa 2015	
**Reason for exclusion**	Wrong Study Design
O'Brien et al., 1997	
**Reason for exclusion**	Unable to Access Article
Oakley et al., 2004	
**Reason for exclusion**	Wrong Study Design
Olivares et al., 2012	
**Reason for exclusion**	Wrong Intervention
Olsen 1999	
**Reason for exclusion**	Wrong Study Design
Olukemi 2006	
**Reason for exclusion**	Wrong Study Design
Omotayo et al., 2016	
**Reason for exclusion**	Wrong Intervention
Omotayo et al., 2017	
**Reason for exclusion**	Wrong Outcome
Osendarp et al., 2003	
**Reason for exclusion**	Wrong Study Design
Osendarp and Neufeld 2013	
**Reason for exclusion**	Wrong Patient Population
Osungbade and Oladunjoye 2012	
**Reason for exclusion**	Wrong Study Design
Palacios et al., 2016	
**Reason for exclusion**	Wrong Study Design
Palma et al., 2008	
**Reason for exclusion**	Wrong Patient Population
Passerini et al., 2012	
**Reason for exclusion**	Wrong Intervention
Patimah et al., 2013	
**Reason for exclusion**	Wrong Patient Population
Peacocke et al., 2015	
**Reason for exclusion**	Wrong Patient Population
Peña‐Rosas 2015	
**Reason for exclusion**	Wrong Study Design
Peña‐Rosas 2015a	
**Reason for exclusion**	Wrong Study Design
Posada et al., 2016	
**Reason for exclusion**	Wrong Intervention
Poulsen et al., 2014	
**Reason for exclusion**	Wrong Patient Population
Prado 2012	
**Reason for exclusion**	Wrong Patient Population
Prado et al., 2012a	
**Reason for exclusion**	Wrong Outcome
Prado et al., 2012b	
**Reason for exclusion**	Wrong Outcome
Prado 2016	
**Reason for exclusion**	Unable to Access Article
Prendergast 2014	
**Reason for exclusion**	Wrong Study Design
Prentice 2009	
**Reason for exclusion**	Wrong Comparison
Pritwani and Mathur 2015	
**Reason for exclusion**	Wrong Study Design
Pritwani and Mathur 2015a	
**Reason for exclusion**	Wrong Study Design
Ramakrishnan et al., 2013	
**Reason for exclusion**	Wrong Study Design
Ramakrishnan et al., 2016	
**Reason for exclusion**	Wrong Intervention
Ramakrishnan et al., 2016a	
**Reason for exclusion**	Wrong Intervention
Ridwan et al., 1996	
**Reason for exclusion**	Wrong Patient Population
Roberfroid 2010	
**Reason for exclusion**	Wrong Outcome
Roberfroid 2012	
**Reason for exclusion**	Wrong Outcome
Roberfroid et al., 2012a	
**Reason for exclusion**	Wrong Outcome
Roche 2017	
**Reason for exclusion**	Wrong Patient Population
Rogers et al., 1999	
**Reason for exclusion**	Wrong Patient Population
Saaka 2013	
**Reason for exclusion**	Wrong Study Design
Sadighian et al., 2013	
**Reason for exclusion**	Wrong Intervention
Sahoo et al., 2016	
**Reason for exclusion**	Wrong comparison
Salam et al., 2014a	
**Reason for exclusion**	Wrong Study Design
Samimi et al., 2016	
**Reason for exclusion**	Wrong Patient Population
Samuel 2013	
**Reason for exclusion**	Wrong Intervention
Sanchez‐Ramos 2015	
**Reason for exclusion**	Wrong Patient Population
Seal and Claudine 2007	
**Reason for exclusion**	Wrong Intervention
Sezikawa 2010	
**Reason for exclusion**	Wrong Patient Population
Shah and Ohlsson 2009	
**Reason for exclusion**	Wrong Study Design
Shaheen 2014	
**Reason for exclusion**	Wrong Outcome
Shaheen et al., 2014a	
**Reason for exclusion**	Wrong Outcome
Shahgheibi et al., 2016	
**Reason for exclusion**	Wrong Patient Population
Shakiba and Iranmanesh 2013	
**Reason for exclusion**	Wrong Comparison
Shankar 2009	
**Reason for exclusion**	Wrong Outcome
Shankar et al., 2009a	
**Reason for exclusion**	Wrong Outcome
Shaw 2003	
**Reason for exclusion**	Wrong Study Design
Shrimpton et al., 2009	
**Reason for exclusion**	Wrong Intervention
Shrimpton et al., 2009a	
**Reason for exclusion**	Wrong Study Design
Siega‐Riz et al., 2001	
**Reason for exclusion**	Wrong Patient Population
Siega‐Riz et al., 2006	
**Reason for exclusion**	Wrong Patient Population
Skeaff 2011	
**Reason for exclusion**	Wrong Study Design
Soheilykhah et al., 2013	
**Reason for exclusion**	Wrong Comparison
Sokpuh et al., 2014	
**Reason for exclusion**	Wrong Setting
Spies et al., 2017	
**Reason for exclusion**	Wrong Patient Population
Spies et al., 2017a	
**Reason for exclusion**	Wrong Study Design
Stagnaro‐Green et al., 2012	
**Reason for exclusion**	Wrong Study Design
Suchdev et al., 2011	
**Reason for exclusion**	Wrong Study Design
Suchdev et al., 2011a	
**Reason for exclusion**	Wrong Study Design
Suchdev et al., 2015	
**Reason for exclusion**	Wrong Study Design
Sun et al., 2010	
**Reason for exclusion**	Wrong patient population
Tan and Ng 1995	
**Reason for exclusion**	Wrong Study Design
Tasnim et al., 2007	
**Reason for exclusion**	Wrong Study Design
Tirivayi et al., 2015	
**Reason for exclusion**	Wrong Intervention
Tirivayi et al., 2015a	
**Reason for exclusion**	Wrong Intervention
Tolarova and Harris 1995	
**Reason for exclusion**	Wrong Patient Population
Trevant et al., 2014	
**Reason for exclusion**	Wrong Intervention
UNICEF, 2002	
**Reason for exclusion**	Wrong Outcome
USAID 2015	
**Reason for exclusion**	Wrong Study Design
Vanbruaene et al., 2015	
**Reason for exclusion**	Wrong Patient Population
van den Broek 1998	
**Reason for exclusion**	Unable to Access Article
van den Broek et al., 2006	
**Reason for exclusion**	Wrong Patient Population
Van der Walt 2005	
**Reason for exclusion**	Wrong Study Design
Veena et al., 2016	
**Reason for exclusion**	Wrong Study Design
Vijaya 2009	
**Reason for exclusion**	Wrong Intervention
Visser et al., 2015	
**Reason for exclusion**	Wrong Patient Population
Walters et al., 2016	
**Reason for exclusion**	Wrong Patient Population
Wibowo et al., 2012	
**Reason for exclusion**	Wrong Patient Population
Wirth et al., 2017	
**Reason for exclusion**	Wrong Patient Population
World Health Organization 1996	
**Reason for exclusion**	Wrong Study Design
Xu et al., 2014	
**Reason for exclusion**	Wrong Intervention
Yakoob et al., 2010	
**Reason for exclusion**	Wrong Study Design
Yakoob et al., 2010a	
**Reason for exclusion**	Wrong Study Design
Yakoob et al., 2010b	
**Reason for exclusion**	Wrong Study Design
Yakoob et al., 2010c	
**Reason for exclusion**	Wrong Study Design
Yakoob et al., 2010d	
**Reason for exclusion**	Wrong Study Design
Yang et al., 2012	
**Reason for exclusion**	Wrong Intervention
Yarrinton and Pearce 2011	
**Reason for exclusion**	Wrong Study Design
Yu et al., 2009	
**Reason for exclusion**	Wrong Patient Population
Zeng et al., 2011	
**Reason for exclusion**	Wrong Patient Population
Zhang 2007	
**Reason for exclusion**	Wrong Outcome
Zhou et al., 2015	
**Reason for exclusion**	Wrong Setting
Zimmermann and Delange 2004	
**Reason for exclusion**	Wrong Study Design
Zimmermann 2007	
**Reason for exclusion**	Wrong Intervention
Zimmermann 2008	
**Reason for exclusion**	Wrong Study Design
Zimmermann 2012	
**Reason for exclusion**	Wrong Study Design
Zutschi et al., 2004	
**Reason for exclusion**	Wrong Patient Population

#### Characteristics of studies awaiting classification

##### Characteristics of ongoing studies

**Summary of findings tables**

**1. Iron folic acid supplementation compared to folic acid (control) for women during pregnancy**

**Table:** 

Iron folic acid supplementation compared to folic acid (control) for women during pregnancy						
Patient or population: women during pregnancy						
Setting: low‐ and middle‐income countries						
Intervention: Iron folic acid supplementation						
Comparison: folic acid (control)						
Outcomes	Anticipated absolute effects* (95% CI)		Relative effect (95% CI)	№ of participants (studies)	Certainty of the evidence (GRADE)	Comments
	Risk with folic acid (control)	Risk with Iron folic acid supplementation				
Maternal Anemia assessed with: Hb < 110 g/L	Study population		RR 0.52 (0.41 to 0.66)	15540 (5 RCTs)	⊕⊕⊕⊝ MODERATE^1^	
	149 per 1,000	77 per 1,000 (61 to 98)				
Low Birth Weight assessed with: <2500 g	Study population		RR 0.88 (0.78 to 0.99)	17257 (4 RCTs)	⊕⊕⊕⊕ HIGH	
	55 per 1,000	48 per 1,000 (43 to 54)				
Perinatal Mortality	Study population		RR 0.88 (0.71 to 1.08)	17464 (4 RCTs)	⊕⊕⊕⊝ MODERATE^1^	
	22 per 1,000	19 per 1,000 (15 to 23)				

**CI:** Confidence interval; **RR:** Risk ratio; **OR:** Odds ratio.

**GRADE Working Group grades of evidence**

**High certainty:** We are very confident that the true effect lies close to that of the estimate of the effect

**Moderate certainty:** We are moderately confident in the effect estimate: The true effect is likely to be close to the estimate of the effect, but there is a possibility that it is substantially different

**Low certainty:** Our confidence in the effect estimate is limited: The true effect may be substantially different from the estimate of the effect

**Very low certainty:** We have very little confidence in the effect estimate: The true effect is likely to be substantially different from the estimate of effect

* **The risk in the intervention group** (and its 95% confidence interval) is based on the assumed risk in the comparison group and the **relative effect** of the intervention (and its 95% CI).

1 Evidence of funnel plot asymmetry, indicating possible publication bias.

**2. Multiple micronutrients supplementation compared to iron with or without folic acid (control) for women during pregnancy**

**Table:** 

Multiple micronutrients supplementation compared to iron with or without folic acid (control) for women during pregnancy						
Patient or population: women during pregnancy						
Setting: low‐ and middle‐income countries						
Intervention: Multiple micronutrients supplementation						
Comparison: iron with or without folic acid (control)						
Outcomes	Anticipated absolute effects* (95% CI)		Relative effect (95% CI)	№ of participants (studies)	Certainty of the evidence (GRADE)	Comments
	Risk with iron with or without folic acid (control)	Risk with Multiple micronutrients supplementation				
Maternal Mortality	Study population		RR 1.04 (0.71 to 1.51)	75051 (7 RCTs)	⊕⊕⊕⊝ MODERATE^1^	
	1 per 1,000	1 per 1,000 (1 to 2)				
Maternal Anemia assessed with: Hb < 110 g/L	Study population		RR 0.95 (0.82 to 1.10)	23556 (16 RCTs)	⊕⊕⊕⊕ HIGH	
	238 per 1,000	226 per 1,000 (195 to 262)				
Maternal Iron‐deficiency Anemia assessed with: concurrent anaemia and iron deficiency	Study population		RR 1.12 (0.62 to 2.02)	1595 (4 RCTs)	⊕⊝⊝⊝ VERY LOW^2^ ^3^ ^4^	
	129 per 1,000	144 per 1,000 (80 to 260)				
Low Birth Weight assessed with: <2500 g	Study population		RR 0.85 (0.77 to 0.93)	79972 (28 RCTs)	⊕⊕⊕⊕ HIGH^4^	
	158 per 1,000	134 per 1,000 (122 to 147)				
Perinatal Mortality	Study population		RR 1.00 (0.90 to 1.11)	92769 (16 RCTs)	⊕⊕⊕⊕ HIGH^4^	
	50 per 1,000	50 per 1,000 (45 to 56)			

**CI:** Confidence interval; **RR:** Risk ratio; **OR:** Odds ratio.

**GRADE Working Group grades of evidence**

**High certainty:** We are very confident that the true effect lies close to that of the estimate of the effect

**Moderate certainty:** We are moderately confident in the effect estimate: The true effect is likely to be close to the estimate of the effect, but there is a possibility that it is substantially different

**Low certainty:** Our confidence in the effect estimate is limited: The true effect may be substantially different from the estimate of the effect

**Very low certainty:** We have very little confidence in the effect estimate: The true effect is likely to be substantially different from the estimate of effect

* **The risk in the intervention group** (and its 95% confidence interval) is based on the assumed risk in the comparison group and the **relative effect** of the intervention (and its 95% CI).

1 Confidence interval of point estimate fails to exclude important harm.

2 Wide variance of point estimates and large I².

3 Small number of events.

4 Evidence of funnel plot asymmetry indicating possible publication bias.

**3. Lipid nutrient supplementation compared to multiple micronutrient supplementation (control) for women during pregnancy**

**Table:** 

Lipid nutrient supplementation compared to multiple micronutrient supplementation (control) for women during pregnancy						
Patient or population: women during pregnancy						
Setting:						
Intervention: Lipid nutrient supplementation						
Comparison: multiple micronutrient supplementation (control)						
Outcomes	Anticipated absolute effects* (95% CI)		Relative effect (95% CI)	№ of participants (studies)	Certainty of the evidence (GRADE)	Comments
	Risk with multiple micronutrient supplementation (control)	Risk with Lipid nutrient supplementation				
Low Birth Weight assessed with: <2500 g	Study population		RR 0.92 (0.75 to 1.13)	2727 (4 RCTs)	⊕⊕⊕⊝ MODERATE^1^	
	122 per 1,000	112 per 1,000 (92 to 138)			
Perinatal Mortality	Study population		RR 1.01 (0.62 to 1.65)	2771 (3 RCTs)	⊕⊕⊝⊝ LOW^1^ ^2^ ^3^	
	30 per 1,000	30 per 1,000 (18 to 49)				

**CI:** Confidence interval; **RR:** Risk ratio; **OR:** Odds ratio.

**GRADE Working Group grades of evidence**

**High certainty:** We are very confident that the true effect lies close to that of the estimate of the effect

**Moderate certainty:** We are moderately confident in the effect estimate: The true effect is likely to be close to the estimate of the effect, but there is a possibility that it is substantially different

**Low certainty:** Our confidence in the effect estimate is limited: The true effect may be substantially different from the estimate of the effect

**Very low certainty:** We have very little confidence in the effect estimate: The true effect is likely to be substantially different from the estimate of effect

* **The risk in the intervention group** (and its 95% confidence interval) is based on the assumed risk in the comparison group and the **relative effect** of the intervention (and its 95% CI).

1 Risk of bias for a substantial proportion of studies was high or unclear

2 Confidence interval of point estimate fails to exclude important harm

3 Small number of events

**4. Iron compared to Placebo in low‐ and middle‐income countries: a systematic review**

**Table:** 

Iron compared to Placebo in low‐ and middle‐income countries: a systematic review						
Patient or population: low‐ and middle‐income countries: a systematic review						
Setting:						
Intervention: Iron						
Comparison: Placebo						
Outcomes	Anticipated absolute effects* (95% CI)		Relative effect (95% CI)	№ of participants (studies)	Certainty of the evidence (GRADE)	Comments
	Risk with Placebo	Risk with Iron				
Maternal Anemia assessed with: Hb <110 g/L	Study population		RR 0.53 (0.43 to 0.65)	15737 (6 RCTs)	⊕⊕⊕⊝ MODERATE^1^	
	156 per 1,000	83 per 1,000 (67 to 101)			
Low Birthweight assessed with: <2500 g	Study population		RR 0.88 (0.78 to 0.99)	17257 (4 RCTs)	⊕⊕⊕⊕ HIGH	
	55 per 1,000	48 per 1,000 (43 to 54)				
Perinatal Mortality	Study population		RR 0.88 (0.71 to 1.08)	17464 (4 RCTs)	⊕⊕⊕⊕ HIGH	
	22 per 1,000	19 per 1,000 (15 to 23)			

**CI:** Confidence interval; **RR:** Risk ratio; **OR:** Odds ratio.

**GRADE Working Group grades of evidence**

**High certainty:** We are very confident that the true effect lies close to that of the estimate of the effect

**Moderate certainty:** We are moderately confident in the effect estimate: The true effect is likely to be close to the estimate of the effect, but there is a possibility that it is substantially different

**Low certainty:** Our confidence in the effect estimate is limited: The true effect may be substantially different from the estimate of the effect

**Very low certainty:** We have very little confidence in the effect estimate: The true effect is likely to be substantially different from the estimate of effect

* **The risk in the intervention group** (and its 95% confidence interval) is based on the assumed risk in the comparison group and the **relative effect** of the intervention (and its 95% CI).

1 Evidence of funnel plot asymmetry, indicating possible publication bias.

**5. Zinc compared to Placebo in low‐ and middle‐income countries: a systematic review**

**Table:** 

Zinc compared to Placebo in low‐ and middle‐income countries: a systematic review						
Patient or population: low‐ and middle‐income countries: a systematic review						
Setting:						
Intervention: Zinc						
Comparison: Placebo						
Outcomes	Anticipated absolute effects* (95% CI)		Relative effect (95% CI)	№ of participants (studies)	Certainty of the evidence (GRADE)	Comments
	Risk with Placebo	Risk with Zinc				
Low Birth Weight assessed with: <2500 g	Study population		RR 1.08 (0.94 to 1.25)	4633 (10 RCTs)	⊕⊕⊕⊝ MODERATE^1^	
	204 per 1,000	220 per 1,000 (192 to 255)			

**CI:** Confidence interval; **RR:** Risk ratio; **OR:** Odds ratio.

**GRADE Working Group grades of evidence**

**High certainty:** We are very confident that the true effect lies close to that of the estimate of the effect

**Moderate certainty:** We are moderately confident in the effect estimate: The true effect is likely to be close to the estimate of the effect, but there is a possibility that it is substantially different

**Low certainty:** Our confidence in the effect estimate is limited: The true effect may be substantially different from the estimate of the effect

**Very low certainty:** We have very little confidence in the effect estimate: The true effect is likely to be substantially different from the estimate of effect

* **The risk in the intervention group** (and its 95% confidence interval) is based on the assumed risk in the comparison group and the **relative effect** of the intervention (and its 95% CI).

1 Confidence interval of point estimate does not exclude important harm.

**6. Vitamin A supplementation compared to placebo for women during pregnancy**

**Table:** 

Vitamin A supplementation compared to placebo for women during pregnancy						
Patient or population: women during pregnancy						
Setting: low‐ and middle‐income countries						
Intervention: Vitamin A supplementation						
Comparison: placebo						
Outcomes	Anticipated absolute effects* (95% CI)		Relative effect (95% CI)	№ of participants (studies)	Certainty of the evidence (GRADE)	Comments
	Risk with placebo	Risk with Vitamin A supplementation				
Maternal Mortality	Study population		RR 0.90 (0.68 to 1.18)	124002 (3 RCTs)	⊕⊕⊝⊝ LOW^1^ ^2^ ^3^	
	354 per 100,000	318 per 100,000 (241 to 417)			

**CI:** Confidence interval; **RR:** Risk ratio; **OR:** Odds ratio.

**GRADE Working Group grades of evidence**

**High certainty:** We are very confident that the true effect lies close to that of the estimate of the effect

**Moderate certainty:** We are moderately confident in the effect estimate: The true effect is likely to be close to the estimate of the effect, but there is a possibility that it is substantially different

**Low certainty:** Our confidence in the effect estimate is limited: The true effect may be substantially different from the estimate of the effect

**Very low certainty:** We have very little confidence in the effect estimate: The true effect is likely to be substantially different from the estimate of effect

* **The risk in the intervention group** (and its 95% confidence interval) is based on the assumed risk in the comparison group and the **relative effect** of the intervention (and its 95% CI).

1 Risk of bias for a substantial proportion of studies were high or unclear.

2 Low number of events.

3 Confidence interval of point estimate crosses the threshold for decision. making.

**7. Calcium compared to Placebo in low‐ and middle‐income countries: a systematic review**

**Table:** 

Calcium compared to Placebo in low‐ and middle‐income countries: a systematic review						
Patient or population: low‐ and middle‐income countries: a systematic review						
Setting:						
Intervention: Calcium						
Comparison: Placebo						
Outcomes	Anticipated absolute effects* (95% CI)		Relative effect (95% CI)	№ of participants (studies)	Certainty of the evidence (GRADE)	Comments
	Risk with Placebo	Risk with Calcium				
Low Birth Weight assessed with: <2500 g	Study population		RR 0.99 (0.95 to 1.04)	9498 (3 RCTs)	⊕⊕⊕⊕ HIGH	
	168 per 1,000	166 per 1,000 (159 to 175)			

**CI:** Confidence interval; **RR:** Risk ratio; **OR:** Odds ratio.

**GRADE Working Group grades of evidence**

**High certainty:** We are very confident that the true effect lies close to that of the estimate of the effect

**Moderate certainty:** We are moderately confident in the effect estimate: The true effect is likely to be close to the estimate of the effect, but there is a possibility that it is substantially different

**Low certainty:** Our confidence in the effect estimate is limited: The true effect may be substantially different from the estimate of the effect

**Very low certainty:** We have very little confidence in the effect estimate: The true effect is likely to be substantially different from the estimate of effect

* **The risk in the intervention group** (and its 95% confidence interval) is based on the assumed risk in the comparison group and the **relative effect** of the intervention (and its 95% CI).

Additional tables

## DATA AND ANALYSES

1. IFA vs Folic Acid

**Table:** 

Outcome or Subgroup	Studies	Participants	Statistical Method	Effect Estimate
1.1 Maternal Anemia	5	15540	Risk Ratio (M‐H, Random, 95% CI)	0.52 [0.41, 0.66]
1.2 Low Birthweight	4	17257	Risk Ratio (M‐H, Random, 95% CI)	0.88 [0.78, 0.99]
1.3 Perinatal Mortality	4	17464	Risk Ratio (M‐H, Random, 95% CI)	0.88 [0.71, 1.08]
1.4 Serum/Plasma Hemoglobin (g/L)	7	16089	Mean Difference (IV, Random, 95% CI)	6.95 [2.80, 11.11]
1.5 Serum/plasma ferritin (ug/L)	5	3894	Mean Difference (IV, Random, 95% CI)	15.87 [2.96, 28.79]
1.6 Serum/plasma Tfr (mg/L)	3	2431	Mean Difference (IV, Random, 95% CI)	‐0.16 [‐0.96, 0.65]
1.7 Neonatal Mortality	3	15794	Risk Ratio (M‐H, Random, 95% CI)	0.85 [0.55, 1.31]
1.8 Preterm Births	5	17637	Risk Ratio (M‐H, Random, 95% CI)	0.96 [0.64, 1.44]
1.9 Small‐for‐gestational age	4	6549	Risk Ratio (M‐H, Random, 95% CI)	1.04 [0.87, 1.24]
1.10 Infant Mortality	3	14748	Risk Ratio (M‐H, Random, 95% CI)	1.10 [0.84, 1.45]

2. MMN vs IFA

**Table:** 

Outcome or Subgroup	Studies	Participants	Statistical Method	Effect Estimate
2.1 Maternal Mortality	7	75051	Risk Ratio (IV, Random, 95% CI)	1.04 [0.71, 1.51]
2.2 Maternal Anemia	16	23556	Risk Ratio (IV, Random, 95% CI)	1.02 [0.95, 1.10]
2.2.1 >4 micronutrients	13	22695	Risk Ratio (IV, Random, 95% CI)	1.02 [0.96, 1.08]
2.2.2 <or equal to 4 micronutrients	3	861	Risk Ratio (IV, Random, 95% CI)	1.03 [0.61, 1.72]
2.3 Maternal Iron‐deficiency Anemia	4	1595	Risk Ratio (IV, Random, 95% CI)	1.12 [0.62, 2.02]
2.3.1 >4 micronutrients	3	1350	Risk Ratio (IV, Random, 95% CI)	0.92 [0.37, 2.26]
2.3.2 <or equal to 4 micronutrients	1	245	Risk Ratio (IV, Random, 95% CI)	1.45 [0.80, 2.66]
2.4 Low Birthweight	28	79972	Risk Ratio (IV, Random, 95% CI)	0.85 [0.77, 0.93]
2.4.1 >4 micronutrients	19	68138	Risk Ratio (IV, Random, 95% CI)	0.79 [0.71, 0.88]
2.4.2 <or equal to 4 micronutrients	9	11834	Risk Ratio (IV, Random, 95% CI)	1.01 [0.92, 1.11]
2.5 Perinatal Mortality	16	92769	Risk Ratio (IV, Random, 95% CI)	1.00 [0.90, 1.11]
2.5.1 >4 micronutrients	14	90959	Risk Ratio (IV, Random, 95% CI)	0.97 [0.88, 1.07]
2.5.2 <or equal to 4 micronutrients	2	1810	Risk Ratio (IV, Random, 95% CI)	1.79 [0.56, 5.76]
2.6 Serum/Plasma Hemoglobin (g/L)	16	26312	Mean Difference (IV, Random, 95% CI)	‐0.34 [‐1.53, 0.86]
2.6.1 >4 micronutrients	10	24719	Mean Difference (IV, Random, 95% CI)	‐0.30 [‐1.27, 0.67]
2.6.2 <or equal to 4 micronutrients	6	1593	Mean Difference (IV, Random, 95% CI)	‐0.72 [‐4.44, 3.00]
2.7 Serum/Plasma Ferritin (ug/L)	11	5149	Mean Difference (IV, Random, 95% CI)	‐2.37 [‐7.84, 3.10]
2.7.1 >4 micronutrients	7	4016	Mean Difference (IV, Random, 95% CI)	‐3.17 [‐9.87, 3.53]
2.7.2 <or equal to 4 micronutrients	4	1133	Mean Difference (IV, Random, 95% CI)	‐0.41 [‐4.53, 3.70]
2.8 Maternal Iron Deficiency	3	1182	Risk Ratio (M‐H, Random, 95% CI)	1.39 [0.88, 2.20]
2.8.1 >4 micronutrients	2	1058	Risk Ratio (M‐H, Random, 95% CI)	1.68 [0.85, 3.32]
2.8.2 <or equal to 4 micronutrients	1	124	Risk Ratio (M‐H, Random, 95% CI)	0.97 [0.71, 1.33]
2.9 Serum/Plasma TfR (mg/L)	7	2550	Mean Difference (IV, Random, 95% CI)	0.12 [‐0.03, 0.27]
2.9.1 >4 micronutrients	4	2111	Mean Difference (IV, Random, 95% CI)	0.26 [‐0.09, 0.61]
2.9.2 <or equal to 4 micronutrients	3	439	Mean Difference (IV, Random, 95% CI)	‐0.02 [‐0.22, 0.18]
2.10 Serum/Plasma Retinol (umol/L)	7	3111	Mean Difference (IV, Random, 95% CI)	0.11 [0.05, 0.17]
2.10.1 >4 micronutrients	4	2805	Mean Difference (IV, Random, 95% CI)	0.09 [0.02, 0.16]
2.10.2 <or equal to 4 micronutrients	3	306	Mean Difference (IV, Random, 95% CI)	0.13 [0.06, 0.21]
2.11 Serum/Plasma Zinc (umol/L)	5	3028	Mean Difference (IV, Random, 95% CI)	0.40 [0.18, 0.62]
2.11.1 >4 micronutrients	3	2423	Mean Difference (IV, Random, 95% CI)	0.51 [0.11, 0.91]
2.11.2 <or equal to 4 micronutrients	2	605	Mean Difference (IV, Random, 95% CI)	0.29 [0.05, 0.53]
2.12 Serum/Plasma Vitamin B12 (pmol/L)	3	962	Mean Difference (IV, Random, 95% CI)	14.77 [5.13, 24.42]
2.12.1 >4 micronutrients	2	943	Mean Difference (IV, Random, 95% CI)	14.24 [4.36, 24.12]
2.12.2 <or equal to 4 micronutrients	1	19	Mean Difference (IV, Random, 95% CI)	25.50 [‐18.85, 69.85]
2.13 Serum/Plasma Folate (nmol/L)	5	2614	Mean Difference (IV, Random, 95% CI)	‐1.66 [‐4.39, 1.08]
2.13.1 >4 micronutrients	4	2595	Mean Difference (IV, Random, 95% CI)	‐2.21 [‐5.11, 0.69]
2.13.2 <or equal to 4 micronutrients	1	19	Mean Difference (IV, Random, 95% CI)	1.80 [‐3.91, 7.51]
2.14 Miscarriage	13	88971	Risk Ratio (IV, Random, 95% CI)	0.99 [0.94, 1.04]
2.15 Stillbirths	22	96772	Risk Ratio (IV, Random, 95% CI)	0.91 [0.86, 0.98]
2.15.1 >4 micronutrients	17	86120	Risk Ratio (IV, Random, 95% CI)	0.91 [0.85, 0.98]
2.15.2 <or equal to 4 micronutrients	5	10652	Risk Ratio (IV, Random, 95% CI)	0.93 [0.77, 1.14]
2.16 Congenital Anomalies	7	4195	Risk Ratio (IV, Fixed, 95% CI)	0.73 [0.41, 1.29]
2.16.1 >4 micronutrients	3	1995	Risk Ratio (IV, Fixed, 95% CI)	1.02 [0.30, 3.45]
2.16.2 <or equal to 4 micronutrients	4	2200	Risk Ratio (IV, Fixed, 95% CI)	0.66 [0.35, 1.26]
2.17 Preterm births	29	99855	Risk Ratio (IV, Random, 95% CI)	0.96 [0.91, 1.01]
2.17.1 >4 micronutrients	18	86957	Risk Ratio (IV, Random, 95% CI)	0.97 [0.91, 1.03]
2.17.2 <or equal to 4 micronutrients	11	12898	Risk Ratio (IV, Random, 95% CI)	0.93 [0.84, 1.02]
2.18 Small‐for‐gestational age	19	52965	Risk Ratio (IV, Random, 95% CI)	0.93 [0.88, 0.98]
2.18.1 >4 micronutrients	16	50788	Risk Ratio (IV, Random, 95% CI)	0.90 [0.85, 0.96]
2.18.2 <or equal to 4 micronutrients	3	2177	Risk Ratio (IV, Random, 95% CI)	1.07 [0.98, 1.16]
2.19 Mode of Delivery ‐ Caesarean section	11	23744	Risk Ratio (IV, Random, 95% CI)	1.00 [0.94, 1.07]
2.19.1 >4 micronutrients	5	13217	Risk Ratio (IV, Random, 95% CI)	1.13 [0.99, 1.29]
2.19.2 <or equal to 4 micronutrients	6	10527	Risk Ratio (IV, Random, 95% CI)	0.97 [0.90, 1.04]
2.20 Neonatal Mortality	17	82293	Risk Ratio (IV, Fixed, 95% CI)	0.98 [0.90, 1.06]
2.20.1 >4 micronutrients	14	80278	Risk Ratio (IV, Fixed, 95% CI)	0.97 [0.89, 1.05]
2.20.2 <or equal to 4 micronutrients	3	2015	Risk Ratio (IV, Fixed, 95% CI)	1.30 [0.83, 2.03]
2.21 Infant Mortality	10	55595	Risk Ratio (M‐H, Random, 95% CI)	0.99 [0.92, 1.08]
2.22 Wasting	5	9671	Risk Ratio (M‐H, Random, 95% CI)	1.02 [0.88, 1.18]
2.23 Stunting	7	11264	Risk Ratio (M‐H, Random, 95% CI)	0.99 [0.92, 1.07]
2.24 Underweight	4	9319	Risk Ratio (M‐H, Random, 95% CI)	0.95 [0.84, 1.07]
2.25 Developmental Outcomes ‐ General Intelligence	8	12172	Std. Mean Difference (IV, Random, 95% CI)	0.00 [‐0.06, 0.07]
2.25.1 >4 micronutrients	5	11482	Std. Mean Difference (IV, Random, 95% CI)	0.01 [‐0.05, 0.08]
2.25.2 <or equal to 4 micronutrients	3	690	Std. Mean Difference (IV, Random, 95% CI)	‐0.07 [‐0.29, 0.15]
2.26 Developmental Outcomes ‐ Motor Function	7	12057	Std. Mean Difference (IV, Random, 95% CI)	‐0.02 [‐0.17, 0.13]
2.26.1 >4 micronutrients	4	11367	Std. Mean Difference (IV, Random, 95% CI)	0.03 [‐0.11, 0.16]
2.26.2 <or equal to 4 micronutrients	3	690	Std. Mean Difference (IV, Random, 95% CI)	‐0.14 [‐0.78, 0.51]
2.27 Developmental Outcomes ‐ Verbal Comprehension & Language	4	10781	Std. Mean Difference (IV, Random, 95% CI)	0.02 [‐0.13, 0.16]
2.27.1 >4 micronutrients	3	10600	Std. Mean Difference (IV, Random, 95% CI)	0.03 [‐0.13, 0.20]
2.27.2 <or equal to 4 micronutrients	1	181	Std. Mean Difference (IV, Random, 95% CI)	‐0.09 [‐0.38, 0.20]
2.28 Developmental Outcomes ‐ Executive Function	3	2511	Std. Mean Difference (IV, Random, 95% CI)	0.09 [0.01, 0.17]
2.29 Diarrhea	4	3142	Risk Ratio (IV, Fixed, 95% CI)	0.84 [0.76, 0.92]
2.29.1 >4 micronutrients	1	1169	Risk Ratio (IV, Fixed, 95% CI)	0.84 [0.76, 0.93]
2.29.2 <or equal to 4 micronutrients	3	1973	Risk Ratio (IV, Fixed, 95% CI)	0.80 [0.52, 1.21]
2.30 Child Serum/Plasma Hemoglobin (g/L)	7	13067	Mean Difference (IV, Random, 95% CI)	0.01 [‐0.63, 0.66]
2.30.1 >4 micronutrients	4	12689	Mean Difference (IV, Random, 95% CI)	0.13 [‐0.18, 0.44]
2.30.2 <or equal to 4 micronutrients	3	378	Mean Difference (IV, Random, 95% CI)	0.42 [‐1.81, 2.65]
2.31 Child serum/plasma retinol (umol/L)	3	868	Mean Difference (IV, Random, 95% CI)	0.06 [0.02, 0.09]
2.31.1 >4 micronutrients	1	680	Mean Difference (IV, Random, 95% CI)	0.05 [0.01, 0.08]
2.31.2 <or equal to 4 micronutrients	2	188	Mean Difference (IV, Random, 95% CI)	0.05 [‐0.02, 0.13]
2.32 Child Serum/Plasma Ferritin (ug/L)	4	1443	Mean Difference (IV, Random, 95% CI)	1.85 [‐0.81, 4.50]
2.32.1 >4 micronutrients	2	1243	Mean Difference (IV, Random, 95% CI)	1.66 [‐2.75, 6.06]
2.32.2 <or equal to 4 micronutrients	2	200	Mean Difference (IV, Random, 95% CI)	2.61 [‐14.89, 20.10]
2.33 Child Serum/Plasma Zinc (umol/L)	3	944	Mean Difference (IV, Random, 95% CI)	0.04 [‐0.21, 0.30]
2.33.1 >4 micronutrients	1	712	Mean Difference (IV, Random, 95% CI)	0.00 [‐0.29, 0.29]
2.33.2 <or equal to 4 micronutrients	2	232	Mean Difference (IV, Random, 95% CI)	0.22 [‐0.35, 0.79]
2.34 Child Anemia	3	1458	Risk Ratio (M‐H, Random, 95% CI)	0.83 [0.54, 1.28]

3. LNS vs MMN

**Table:** 

Outcome or Subgroup	Studies	Participants	Statistical Method	Effect Estimate
3.1 Low birthweight	4	2727	Risk Ratio (M‐H, Random, 95% CI)	0.92 [0.75, 1.13]
3.2 Perinatal Mortality	3	2771	Risk Ratio (M‐H, Random, 95% CI)	1.01 [0.62, 1.65]
3.3 Miscarriage	3	2865	Risk Ratio (M‐H, Random, 95% CI)	1.12 [0.69, 1.80]
3.4 Stillbirths	3	2481	Risk Ratio (M‐H, Random, 95% CI)	0.47 [0.12, 1.81]
3.5 Neonatal Mortality	3	2727	Risk Ratio (M‐H, Random, 95% CI)	0.81 [0.45, 1.45]
3.6 Preterm Birth	4	2953	Risk Ratio (M‐H, Random, 95% CI)	1.15 [0.93, 1.42]
3.7 Small for gestational age	4	2716	Risk Ratio (M‐H, Random, 95% CI)	0.96 [0.86, 1.07]

4. Vitamin A vs. Placebo

**Table:** 

Outcome or Subgroup	Studies	Participants	Statistical Method	Effect Estimate
4.1 Maternal Mortality	3	124002	Risk Ratio (M‐H, Random, 95% CI)	0.90 [0.68, 1.18]
4.2 Serum/Plasma Hemoglobin (g/L)	5	1683	Mean Difference (IV, Random, 95% CI)	0.51 [‐2.42, 3.43]
4.3 Serum/Plasma Retinol (umol/L)	6	1654	Mean Difference (IV, Random, 95% CI)	0.13 [‐0.03, 0.30]
4.4 Stillbirths	3	115223	Risk Ratio (M‐H, Random, 95% CI)	1.01 [0.96, 1.07]

5. Zinc vs. Placebo

**Table:** 

Outcome or Subgroup	Studies	Participants	Statistical Method	Effect Estimate
5.1 Low Birthweight	10	4633	Risk Ratio (M‐H, Random, 95% CI)	1.08 [0.94, 1.25]
5.1.1 Zinc vs. Placebo (additional micronutrients)	8	4176	Risk Ratio (M‐H, Random, 95% CI)	1.02 [0.80, 1.31]
5.1.2 Strictly Zinc vs. Placebo	2	457	Risk Ratio (M‐H, Random, 95% CI)	1.15 [0.92, 1.44]
5.3 Pre‐eclampsia/eclampsia	3	1226	Risk Ratio (M‐H, Random, 95% CI)	1.01 [0.53, 1.93]
5.4 Preterm Births	11	5017	Risk Ratio (M‐H, Random, 95% CI)	0.97 [0.80, 1.17]
5.4.1 Zinc vs. Placebo (additional micronutrients)	9	4560	Risk Ratio (M‐H, Random, 95% CI)	0.96 [0.76, 1.21]
5.4.2 Strictly Zinc vs. Placebo	2	457	Risk Ratio (M‐H, Random, 95% CI)	1.09 [0.71, 1.66]
5.5 Small for gestational age	3	2174	Risk Ratio (M‐H, Random, 95% CI)	1.05 [0.97, 1.13]
5.5.1 Zinc vs. Placebo (additional micronutrients)	2	1764	Risk Ratio (M‐H, Random, 95% CI)	1.08 [0.98, 1.20]
5.5.2 Strictly Zinc vs. Placebo	1	410	Risk Ratio (M‐H, Random, 95% CI)	1.00 [0.90, 1.12]
5.6 Serum/Plasma Zinc (umol/L)	5	1202	Mean Difference (IV, Random, 95% CI)	0.43 [‐0.04, 0.89]
5.6.1 Zinc vs. Placebo (additional micronutrients)	3	717	Mean Difference (IV, Random, 95% CI)	0.01 [‐0.70, 0.72]
5.6.2 Strictly Zinc vs. Placebo	2	485	Mean Difference (IV, Random, 95% CI)	0.86 [0.67, 1.05]

6. Iron vs. Placebo

**Table:** 

Outcome or Subgroup	Studies	Participants	Statistical Method	Effect Estimate
6.1 Maternal Anemia	6	15737	Risk Ratio (M‐H, Random, 95% CI)	0.53 [0.43, 0.65]
6.1.1 Iron vs. Placebo (additional micronutrients)	5	15540	Risk Ratio (M‐H, Random, 95% CI)	0.52 [0.41, 0.66]
6.1.2 Strictly Iron vs. Placebo	1	197	Risk Ratio (M‐H, Random, 95% CI)	0.59 [0.45, 0.77]
6.3 Low Birthweight	4	17257	Risk Ratio (M‐H, Random, 95% CI)	0.88 [0.78, 0.99]
6.4 Perinatal Mortality	4	17464	Risk Ratio (M‐H, Random, 95% CI)	0.88 [0.71, 1.08]
6.5 Pre‐eclampsia/Eclampsia	3	2773	Risk Ratio (M‐H, Random, 95% CI)	1.55 [0.91, 2.63]
6.6 Serum/Plasma Hemoglobin (g/L)	11	17288	Mean Difference (IV, Random, 95% CI)	7.80 [4.08, 11.52]
6.6.1 Iron vs. Placebo (additional micronutrients)	8	16923	Mean Difference (IV, Random, 95% CI)	7.59 [3.43, 11.75]
6.6.2 Strictly Iron vs. Placebo	3	365	Mean Difference (IV, Random, 95% CI)	8.38 [1.16, 15.60]
6.7 Serum/Plasma Ferritin (ug/L)	9	5045	Mean Difference (IV, Random, 95% CI)	24.14 [10.83, 37.45]
6.7.1 Iron vs. Placebo (additional micronutrients)	6	4680	Mean Difference (IV, Random, 95% CI)	32.87 [15.39, 50.34]
6.7.2 Strictly Iron vs. Placebo	3	365	Mean Difference (IV, Random, 95% CI)	7.09 [4.45, 9.72]
6.8 Serum/Plasma TfR (mg/L)	3	2431	Mean Difference (IV, Random, 95% CI)	‐0.16 [‐0.96, 0.65]
6.9 Iron Deficiency	4	2522	Risk Ratio (M‐H, Random, 95% CI)	0.54 [0.40, 0.74]
6.9.1 Iron vs. Placebo (additional micronutrients)	2	2177	Risk Ratio (M‐H, Random, 95% CI)	0.67 [0.54, 0.83]
6.9.2 Strictly Iron vs. Placebo	2	345	Risk Ratio (M‐H, Random, 95% CI)	0.34 [0.23, 0.51]
6.10 Neonatal Mortality	3	15794	Risk Ratio (M‐H, Random, 95% CI)	0.85 [0.55, 1.31]
6.11 Preterm Births	6	18419	Risk Ratio (M‐H, Random, 95% CI)	0.94 [0.63, 1.41]
6.12 Small‐for‐Gestational Age	4	6549	Risk Ratio (M‐H, Random, 95% CI)	1.04 [0.87, 1.24]
6.13 Infant Mortality	3	14748	Risk Ratio (M‐H, Random, 95% CI)	1.10 [0.84, 1.45]

7. Vitamin D vs. Placebo

**Table:** 

Outcome or Subgroup	Studies	Participants	Statistical Method	Effect Estimate
7.1 Preterm births	7	1262	Risk Ratio (M‐H, Random, 95% CI)	0.64 [0.40, 1.04]
7.1.1 Vit D vs. Placebo (additional micronutrients)	5	959	Risk Ratio (M‐H, Random, 95% CI)	0.94 [0.64, 1.36]
7.1.2 Strictly Vit D vs. Placebo	2	303	Risk Ratio (M‐H, Random, 95% CI)	0.33 [0.17, 0.62]
7.2 Small‐for‐gestational age	3	851	Risk Ratio (M‐H, Random, 95% CI)	0.93 [0.57, 1.53]
7.2.1 Vit D vs. Placebo (additional micronutrients)	2	686	Risk Ratio (M‐H, Random, 95% CI)	1.18 [0.93, 1.50]
7.2.2 Strictly Vit D vs. Placebo	1	165	Risk Ratio (M‐H, Random, 95% CI)	0.43 [0.19, 0.98]
7.3 Serum/Plasma Vitamin D (nmol/L)	9	1092	Mean Difference (IV, Random, 95% CI)	44.70 [21.94, 67.45]
7.3.1 Vit D vs. Placebo (additional micronutrients)	6	805	Mean Difference (IV, Random, 95% CI)	47.36 [17.95, 76.76]
7.3.2 Strictly Vit D vs. Placebo	3	287	Mean Difference (IV, Random, 95% CI)	38.12 [2.94, 73.30]
7.4 Serum/Plasma Calcium (mg/dL)	5	759	Mean Difference (IV, Random, 95% CI)	‐0.06 [‐0.21, 0.09]
7.5 Mode of Delivery: Caesarean Section	5	1063	Risk Ratio (M‐H, Random, 95% CI)	1.05 [0.94, 1.18]
7.5.1 Vit D vs. Placebo (additional micronutrients)	4	925	Risk Ratio (M‐H, Random, 95% CI)	1.07 [0.95, 1.20]
7.5.2 Strictly Vit D vs. Placebo	1	138	Risk Ratio (M‐H, Random, 95% CI)	0.97 [0.70, 1.35]

8. Calcium vs. Placebo

**Table:** 

Outcome or Subgroup	Studies	Participants	Statistical Method	Effect Estimate
8.1 Low Birthweight	3	9498	Risk Ratio (M‐H, Random, 95% CI)	0.99 [0.95, 1.04]
8.1.1 Calcium vs. Placebo (additional micronutrients)	1	7868	Risk Ratio (M‐H, Random, 95% CI)	0.98 [0.87, 1.10]
8.1.2 Strictly Calcium vs. Placebo	2	1630	Risk Ratio (M‐H, Random, 95% CI)	0.92 [0.66, 1.29]
8.2 Pre‐eclampsia/eclampsia	4	9616	Risk Ratio (M‐H, Random, 95% CI)	0.45 [0.19, 1.06]
8.2.1 Calcium vs. Placebo (additional micronutrients)	1	8312	Risk Ratio (M‐H, Random, 95% CI)	0.92 [0.75, 1.13]
8.2.2 Strictly Calcium vs. Placebo	3	1304	Risk Ratio (M‐H, Random, 95% CI)	0.30 [0.17, 0.52]
8.3 Stillbirths	4	10287	Risk Ratio (M‐H, Random, 95% CI)	0.87 [0.70, 1.07]
8.3.1 Calcium vs. Placebo (additional micronutrients)	1	8378	Risk Ratio (M‐H, Random, 95% CI)	0.86 [0.69, 1.07]
8.3.2 Strictly Calcium vs. Placebo	3	1909	Risk Ratio (M‐H, Random, 95% CI)	0.97 [0.44, 2.15]
8.4 Preterm birth	4	9933	Risk Ratio (M‐H, Random, 95% CI)	0.84 [0.65, 1.08]
8.4.1 Calcium vs. Placebo (additional micronutrients)	1	8080	Risk Ratio (M‐H, Random, 95% CI)	0.91 [0.80, 1.04]
8.4.2 Strictly Calcium vs. Placebo	3	1853	Risk Ratio (M‐H, Random, 95% CI)	0.72 [0.44, 1.20]
8.5 Mode of delivery ‐ Caesarean Section	3	10000	Risk Ratio (M‐H, Random, 95% CI)	0.99 [0.84, 1.15]
8.5.1 Calcium vs. Placebo (additional micronutrients)	1	8378	Risk Ratio (M‐H, Random, 95% CI)	0.95 [0.87, 1.04]
8.5.2 Strictly Calcium vs. Placebo	2	1622	Risk Ratio (M‐H, Random, 95% CI)	1.10 [0.74, 1.64]

9. MMN vs. IFA: Subgroup analysis for primary outcomes

**Table:** 

Outcome or Subgroup	Studies	Participants	Statistical Method	Effect Estimate
9.1 Maternal Mortality: MMN Formulation	7		Risk Ratio (IV, Random, 95% CI)	1.04 [0.71, 1.51]
9.1.1 UNIMMAP & Adapted UNIMMAP	4		Risk Ratio (IV, Random, 95% CI)	1.05 [0.71, 1.56]
9.1.2 Non‐UNIMMAP	3		Risk Ratio (IV, Random, 95% CI)	0.95 [0.30, 3.02]
9.2 Maternal Mortality: Geographical Region	7		Risk Ratio (IV, Random, 95% CI)	1.04 [0.71, 1.51]
9.2.1 Africa	4		Risk Ratio (IV, Random, 95% CI)	0.88 [0.41, 1.87]
9.2.2 South Asia	3		Risk Ratio (IV, Random, 95% CI)	1.10 [0.71, 1.69]
9.3 Maternal Mortality: Duration of Intervention	7		Risk Ratio (IV, Random, 95% CI)	1.04 [0.71, 1.51]
9.3.1 Enrollment to Delivery	4		Risk Ratio (IV, Random, 95% CI)	1.04 [0.52, 2.09]
9.3.2 Enrollment to greater than or equal to 1 month postpartum	3		Risk Ratio (IV, Random, 95% CI)	1.04 [0.67, 1.62]
9.4 Maternal Mortality: Dose of Iron (mg)	7		Risk Ratio (IV, Random, 95% CI)	1.04 [0.71, 1.51]
9.4.1 60 mg of Iron	4		Risk Ratio (IV, Random, 95% CI)	1.04 [0.42, 2.60]
9.4.2 <60 mg of Iron	3		Risk Ratio (IV, Random, 95% CI)	1.04 [0.69, 1.57]
9.5 Maternal Anemia: MMN Formulation	16		Risk Ratio (IV, Random, 95% CI)	1.02 [0.95, 1.10]
9.5.1 UNIMMAP	7		Risk Ratio (IV, Random, 95% CI)	1.01 [0.97, 1.05]
9.5.2 Adapted UNIMMAP	3		Risk Ratio (IV, Random, 95% CI)	1.02 [0.83, 1.25]
9.5.3 Non‐UNIMMAP	6		Risk Ratio (IV, Random, 95% CI)	1.13 [0.89, 1.44]
9.6 Maternal Anemia: Geographical Region	15		Risk Ratio (IV, Random, 95% CI)	1.02 [0.94, 1.10]
9.6.1 Africa	4		Risk Ratio (IV, Random, 95% CI)	1.11 [0.92, 1.34]
9.6.2 South Asia	7		Risk Ratio (IV, Random, 95% CI)	0.97 [0.86, 1.10]
9.6.3 Western Pacific	4		Risk Ratio (IV, Random, 95% CI)	1.01 [0.89, 1.14]
9.7 Maternal Anemia: Dose of Iron (mg)	16		Risk Ratio (IV, Random, 95% CI)	1.02 [0.95, 1.10]
9.7.1 60 mg of Iron	6		Risk Ratio (IV, Random, 95% CI)	1.05 [0.79, 1.38]
9.7.2 <60 mg of Iron	10		Risk Ratio (IV, Random, 95% CI)	1.02 [0.96, 1.08]
9.8 Perinatal Mortality: MMN Formulation	16		Risk Ratio (IV, Random, 95% CI)	1.00 [0.90, 1.11]
9.8.1 UNIMMAP & Adapted UNIMMAP	10		Risk Ratio (IV, Random, 95% CI)	1.01 [0.89, 1.14]
9.8.2 Non‐UNIMMAP	6		Risk Ratio (IV, Random, 95% CI)	0.98 [0.70, 1.37]
9.9 Perinatal Mortality: Geographical Region	15		Risk Ratio (IV, Random, 95% CI)	1.00 [0.90, 1.12]
9.9.1 Africa	5		Risk Ratio (IV, Random, 95% CI)	0.92 [0.68, 1.25]
9.9.2 Western Pacific	2		Risk Ratio (IV, Random, 95% CI)	1.14 [0.78, 1.65]
9.9.3 South Asia	8		Risk Ratio (IV, Random, 95% CI)	1.01 [0.89, 1.16]
9.10 Perinatal Mortality: Dose of Iron (mg)	16		Risk Ratio (IV, Random, 95% CI)	1.00 [0.90, 1.11]
9.10.1 60 mg of Iron	3		Risk Ratio (IV, Random, 95% CI)	1.09 [0.74, 1.60]
9.10.2 <60 mg of Iron	13		Risk Ratio (IV, Random, 95% CI)	1.00 [0.88, 1.12]
9.11 Low Birthweight: MMN Formulation	26		Risk Ratio (IV, Random, 95% CI)	0.82 [0.74, 0.90]
9.11.1 UNIMMAP	11		Risk Ratio (IV, Random, 95% CI)	0.74 [0.61, 0.90]
9.11.2 Adapted UNIMMAP	3		Risk Ratio (IV, Random, 95% CI)	0.88 [0.85, 0.91]
9.11.3 Non‐UNIMMAP	12		Risk Ratio (IV, Random, 95% CI)	0.92 [0.81, 1.05]
9.12 Low Birthweight: Geographical Region	27		Risk Ratio (IV, Fixed, 95% CI)	0.87 [0.84, 0.90]
9.12.1 Africa	9		Risk Ratio (IV, Fixed, 95% CI)	0.85 [0.77, 0.95]
9.12.2 South Asia	10		Risk Ratio (IV, Fixed, 95% CI)	0.89 [0.86, 0.91]
9.12.3 Americas	2	Risk Ratio (IV, Fixed, 95% CI)	0.96 [0.64, 1.43]
9.12.4 Western Pacific	4		Risk Ratio (IV, Fixed, 95% CI)	0.46 [0.38, 0.56]
9.12.5 Eastern Mediterranean	2		Risk Ratio (IV, Fixed, 95% CI)	0.80 [0.43, 1.49]
9.13 Low Birthweight: Dose of Iron (mg)	25		Risk Ratio (IV, Random, 95% CI)	0.83 [0.75, 0.92]
9.13.1 60 mg of Iron	7		Risk Ratio (IV, Random, 95% CI)	0.96 [0.83, 1.12]
9.13.2 <60 mg of Iron	18		Risk Ratio (IV, Random, 95% CI)	0.79 [0.69, 0.89]

10. Iron vs. Placebo: Subgroup analysis for primary outcomes

**Table:** 

Outcome or Subgroup	Studies	Participants	Statistical Method	Effect Estimate
10.1 Maternal Anemia: Geographical Region	5	15463	Risk Ratio (M‐H, Random, 95% CI)	0.55 [0.44, 0.69]
10.1.1 Africa	2	1179	Risk Ratio (M‐H, Random, 95% CI)	0.60 [0.52, 0.69]
10.1.2 Western Pacific	3	14284	Risk Ratio (M‐H, Random, 95% CI)	0.52 [0.35, 0.77]
10.2 Maternal Anemia: Dose of Iron (mg)	6	15737	Risk Ratio (M‐H, Random, 95% CI)	0.53 [0.43, 0.65]
10.2.1 > or equal to 60 mg of Fe	4	3654	Risk Ratio (M‐H, Random, 95% CI)	0.51 [0.41, 0.64]
10.2.2 <60 mg of Fe	2	12083	Risk Ratio (M‐H, Random, 95% CI)	0.57 [0.34, 0.94]

11. Zinc vs. Placebo: Subgroup analysis for primary outcomes

**Table:** 

Outcome or Subgroup	Studies	Participants	Statistical Method	Effect Estimate
11.2 Low Birthweight: Geographical Region	7	2626	Risk Ratio (M‐H, Random, 95% CI)	1.10 [1.00, 1.21]
11.2.1 South‐East Asia	5	1923	Risk Ratio (M‐H, Random, 95% CI)	1.11 [1.01, 1.22]
11.2.2 Eastern Mediterranean	2	703	Risk Ratio (M‐H, Random, 95% CI)	0.39 [0.04, 4.18]

## SOURCES OF SUPPORT

### Internal sources

Centre for Global Child Health at The Hospital for Sick Children, Canada

Grant Number: OPP1137750

### External sources

Bill & Melinda Gates Foundation, Other

Feedback

## APPENDIX 1 EXISTING REVIEWS

### Iron folic acid supplementation

Peña‐Rosas JP, De‐Regil LM, Gomez Malave H, Flores‐Urrutia MC, Dowswell T. Intermittent oral iron supplementation during pregnancy. Cochrane Database Syst Rev 2015 Oct 19;(10):CD009997. doi:10.1002/14651858.CD009997.pub2.

Peña‐Rosas JP, De‐Regil LM, Garcia‐Casal MN, Dowswell T. Daily oral iron supplementation during pregnancy. Cochrane Database Syst Rev 2015, Issue 7. Art. No.: CD004736. doi:10.1002/14651858.CD004736.pub5.

Haider BA, Olofin I, Wang M, Spiegelman D, Ezzati M, Fawzi WW; Nutrition Impact Model Study Group (anaemia). Anaemia, prenatal iron use, and risk of adverse pregnancy outcomes: systematic review and meta‐analysis. BMJ 2013;21;346:f3443. doi:10.1136/bmj.f3443.

### Multiple micronutrient supplementation

Haider BA, Bhutta ZA. Multiple‐micronutrient supplementation for women during pregnancy. Cochrane Database Syst Rev. 2017 Apr 13;4:CD004905. doi: 10.1002/14651858.CD004905.pub5.

Suchdev PS, Peña‐Rosas JP, De‐Regil LM. Multiple micronutrient powders for home (point‐of‐use) fortification of foods in pregnant women. Cochrane Database Syst Rev 2015, Issue 6. Art. No: CD011158. doi:10.1002/14651858.CD011158.pub2.

### Single micronutrient supplementation

Lassi ZS, Salam RA, Haider BA, Bhutta ZA. Folic acid supplementation during pregnancy for maternal health and pregnancy outcomes. Cochrane Database Syst Rev 2013 Mar 28;(3):CD006896. doi:10.1002/14651858.CD006896.pub2.

Hodgetts VA, Morris RK, Francis A, Gardosi J, Ismail KM. Effectiveness of folic acid supplementation in pregnancy on reducing the risk of small‐for‐gestational age neonates: a population study, systematic review and meta‐analysis. BJOG 2015;122:478

Buppasiri P, Lumbiganon P, Thinkhamrop J, Ngamjarus C, Laopaiboon M, Medley N. Calcium supplementation (other than for preventing or treating hypertension) for improving pregnancy and infant outcomes. Cochrane Database Syst Rev 2015, Issue 2. Art. No.: CD007079. doi:10.1002/14651858.CD007079.pub3.

Hofmeyr GJ, Lawrie TA, Atallah ÁN, Duley L, Torloni MR. Calcium supplementation during pregnancy for preventing hypertensive disorders and related problems. Cochrane Database Syst Rev 2014, Issue 6. Art. No.: CD001059. doi:10.1002/14651858.CD001059.pub4.

Harding KB, Peña‐Rosas JP, Webster AC, Yap CMY, Payne BA, Ota E, De‐Regil LM. Iodine supplementation for women during the preconception, pregnancy and postpartum period. Cochrane Database Syst Rev 2017, Issue 3. Art. No.: CD011761. doi:10.1002/14651858.CD011761.pub2.

De‐Regil LM, Palacios C, Lombardo LK, Peña‐Rosas JP. Vitamin D supplementation for women during pregnancy. Cochrane Database Syst Rev 2016, Issue 1. Art. No: CD008873. doi:10.1002/14651858.CD008873.pub3.

Ota E, Mori R, Middleton P, Tobe‐Gai R, Mahomed K, Miyazaki C, Bhutta ZA. Zinc supplementation for improving pregnancy and infant outcomes. Cochrane Database Syst Rev 2015, Issue 2. Art. No.: CD000230. doi: 10.1002/14651858.CD000230.pub5.

### Lipid‐based nutrient supplementation

Das JK, Salam RA, Weise PZ, et al. Lipid‐based nutrient supplements for pregnant women and their impact on pregnancy, birth, and infant developmental outcomes in stable and emergency settings. 2017 Cochrane (review protocol).

## APPENDIX 2 PRELIMINARY SEARCH STRATEGY: MEDLINE

### Population

1. Pregnant women/or Mothers/or Maternal Age/or Pregnancy in Adolescence/or Pregnancy, Multiple/or exp Perinatology/or First Trimester, Pregnancy/or Second Trimester, Pregnancy/or Third Trimester, Pregnancy/or Obstetrics/or exp Maternal Behavior/or Surrogate Mothers/or Pregnancy Outcome/or Prenatal Care/or Perinatal Care/or Postnatal Care/or Postpartum Period/or Lactation/or Pregnancy Complications/or (pregnan* or mother* or mom* or surroga* or matern* or preconcept* or pre‐concept* or "pre concept*" or periconcept* or peri‐concept* or "peri concept* " or partus or lactation or obstetric* or labo#r or childbear* or "child‐bear*" or gestation* or antenatal or ante‐natal or "ante natal " or pre‐natal or "pre natal " or prenatal or perinatal or peri‐natal or "peri natal" or prepartum or pre‐partum or "pre partum" or perinatology or peripartum or peri‐partum or "peri partum" or puerper* or postpartum or post‐partum or "post partum" or postnatal or post‐natal or "post natal" or "child birth" or child‐birth or childbirth or "term birth" or paturiency or child‐carrying or "child carrying" or (pregnan* and ("reproductive age" or "wom#n of reproductive age" or WRA))).tw,kf

### Single or Multiple Micronutrient Supplementation

2. Micronutrients/or vitamins/or minerals/or exp iron/or exp iron compounds/or iron, dietary/or vitamin A/or exp iodine/or exp zinc or exp zinc compounds/or exp vitamin D/or (micronutrient* or multinutrient* or multi‐nutrient* or "multi*nutrient" or "multimicro‐nutrient*" or "multimicronutrient*" or multivitamin* or "multi‐vitamin*" or multimineral* or "multi‐mineral*" or "multiple micro nutrient*" or "multiple micronutrient" or micro‐nutrient* or MMN or "essential vitamins*" or mineral* or "m.v.i. pediatric" or "trace element*" or "trace mineral*" or "trace metal" or vitamin* or "vitamin d" or "hydroxyvitamin d" or vitamin‐d or "25 hydroxyvitamin d" or "25 hydroxy‐vitamin d" or "25‐hydroxyvitamin d" or "25‐hydroxy‐vitamin d" or "25‐hydroxyvitamin d" or 25ohd or "25‐oh‐vitamin d" or 25‐ohd or "vitamin d2" or vitamin‐d2 or "25‐hydroxyvitamin d2" or "25‐hydroxy‐vitamin d2" or "vitamin d3" or vitamin‐d3 or "25 hydroxyvitamin d3" or "25 hydroxyvitamin d3" or "25‐hydroxy‐vitamin d3" or calcidiol or calcifediol or calcium or retinol* or retinal* or Retinaldehyde or retinoid or Retinoids or retinoic or beta‐carotene or "beta carotene" or iron or "Fe(III)" or "Fe3+" or "iron(III) " or "Ferrous ion" or "Fe(II)" or "iron(II)" or "Fe2+" or "ferr* compounds" or zinc or "zn" or "zn acetate" or "zn sulfate" or "zn oxide" or iodine or "iod* compounds" or "ferr* compounds" or "folic acid" or "ergocalciferol derivative" or "ergocalciferol‐D2" or cholecalciferol‐D3 or "colecalciferol derivative" or iodiz* or "beta carotene" or "b‐tene" or "beta carotin" or betacarotene).tw,kf 3.

Exp Dietary supplements/or tablets/or syrup/or capsules/or powders/or(supplement* or nutraceutical* or nutriceutical* or neutraceutical* or capsule* or tablet* or syrup* or drop* or Sprinkles or powder* or foodlet* or "foodlet‐based" or "crushable nutritabs" or "micronutrient powder*" or "multiple‐micronutrient powder" or mnp).tw,kf

4. 1 AND 2 AND 3

### Lipid‐Nutrient Supplementation

5. exp Lipids/or (lipid* or oil* or soy* or peanut* or whey* or sesame* or cashew* or chickpea* or protein* or butter* or fat or fats or fatty or "dairy product*" or "omega‐3" or "omega 3" or "alpha‐linolenic acid" or "docosahexaenoic acids" or "eicosapentaeonic acid" or "n‐3 pufa" or "n3 pufa" or glyceride*).tw,kf

6. Dietary supplements/or ("lipid based" or "lipid‐based nutri*" or enrich* or emuls* or "Lipid Emulsions" or "Fat Emulsions" or "Intravenous Fat Emulsions" or powder* or spread* or paste* or LNS or iLiNS or supplement* or neutraceutical* or nutraceutical* or nutriceutical* or Nutributter* or Plumpy* or PlumpyNut or "ready to use" or "ready‐to‐use therapeutic food" or "ready‐to‐use supplementary food" or RUFF or RUSF or RUTF).tw,kf

7. 1 AND 5 AND 6

### Low or Middle‐Income Country

8. Developing Countries/or developing country*.tw, kf or Afghanistan/or Guinea/or Rwanda/or Benin/or Guinea‐Bissau/or Senegal/or "Burkina Faso"/or Haiti/or "Sierra Leone"/or Burundi/or "Democratic People's Republic of Korea"/or Somalia/or "Central African Republic"/or Liberia/or "South Sudan"/or Chad/or Madagascar/or Tanzania/or Comoros/or Malawi/or Togo/or "Democratic Republic of the Congo"/or Mali/or Uganda/or Eritrea/or Mozambique/or Zimbabwe/or Ethiopia/or Nepal/or "The Gambia"/or Niger/or Angola/or Indonesia/or Philippines/or Armenia/or Jordan/or "São Tomé and Principe"/or Bangladesh/or Kenya/or "Solomon Islands"/or Bhutan/or Kiribati/or "Sri Lanka"/or Bolivia/or Kosovo/or Sudan/or "Cabo Verde"/or "Kyrgyz Republic"/or Swaziland/or Cambodia/or "Lao PDR"/or "Syrian Arab Republic"/or Cameroon/or Lesotho/or Tajikistan/or "Republic of the Congo"/or Mauritania/or Timor‐Leste/or "Côte d'Ivoire"/or "Federated States of Micronesia"/or Tunisia/or Djibouti/or Moldova/or Ukraine/or "Arab Republic of Egypt"/or Mongolia/or Uzbekistan/or "El Salvador"/or Morocco/or Vanuatu/or Georgia/or Myanmar/or Vietnam/or Ghana/or Nicaragua/or "West Bank and Gaza"/or Guatemala/or Nigeria/or "Republic of Yemen"/or Honduras/or Pakistan/or Zambia/or India/or "Papua New Guinea"/or Albania/or Ecuador/or Nauru/or Algeria/or Fiji/or Panama/or "American Samoa"/or Gabon/or Paraguay/or Argentina/or Grenada/or Peru/or Azerbaijan/or Guyana/or Romania/or Belarus/or "Islamic Republic of Iran"/or "Russian Federation"/or Belize/or Iraq/or Samoa/or "Bosnia and Herzegovina"/or Jamaica/or Serbia/or Botswana/or Kazakhstan/or "South Africa"/or Brazil/or Lebanon/or "St. Lucia"/or Bulgaria/or Libya/or "St. Vincent and the Grenadines"/or China/or "Republic of Macedonia"/or Suriname/or Colombia/or Malaysia/or Thailand/or "Costa Rica"/or Maldives/or Tonga/or Croatia/or "Marshall Islands"/or Turkey/or Cuba/or Mauritius/or Turkmenistan/or Dominica/or Mexico/or Tuvalu/or "Dominican Republic"/or Montenegro/or "Bolivarian Republic of Venezuela"/or "Equatorial Guinea"/or Namibia/or ("developing country" or "developing countries" or "developing nation" or "developing nations" or "developing population" or "developing populations" or "developing world" or "less developed country" or "less developed countries" or "less developed nation" or "less developed nations" or "less developed population" or "less developed populations" or "less developed world" or "lesser developed country" or "lesser developed countries" or "lesser developed nation" or "lesser developed nations" or "lesser developed population" or "lesser developed populations" or "lesser developed world" or "under developed country" or "under developed countries" or "under developed nation" or "under developed nations" or "under developed population" or "under developed populations" or "under developed world" or "underdeveloped country" or "underdeveloped countries" or "underdeveloped nation" or "underdeveloped nations" or "underdeveloped population" or "underdeveloped populations" or "underdeveloped world" or "middle income country" or "middle income countries" or "middle income nation" or "middle income nations" or "middle income population" or "middle income populations" or "low income country" or "low income countries" or "low income nation" or "low income nations" or "low income population" or "low income populations" or "lower income country" or "lower income countries" or "lower income nation" or "lower income nations" or "lower income population" or "lower income populations" or "underserved country" or "underserved countries" or "underserved nation" or "underserved nations" or "underserved population" or "underserved populations" or "underserved world" or "under served country" or "under served countries" or "under served nation" or "under served nations" or "under served population" or "under served populations" or "under served world" or "deprived country" or "deprived countries" or "deprived nation" or "deprived nations" or "deprived population" or "deprived populations" or "deprived world" or "poor country" or "poor countries" or "poor nation" or "poor nations" or "poor population" or "poor populations" or "poor world" or "poorer country" or "poorer countries" or "poorer nation" or "poorer nations" or "poorer population" or "poorer populations" or "poorer world" or "developing economy" or "developing economies" or "less developed economy" or "less developed economies" or "lesser developed economy" or "lesser developed economies" or "under developed economy" or "under developed economies" or "underdeveloped economy" or "underdeveloped economies" or "middle income economy" or "middle income economies" or "low income economy" or "low income economies" or "lower income economy" or "lower income economies" or "low gdp" or "low gnp" or "low gross domestic" or "low gross national" or "lower gdp" or "lower gnp" or "lower gross domestic" or "lower gross national" or lmic or lmics or "third world" or "lami country" or "lami countries" or "transitional country" or "transitional countries" or Africa or Asia or Caribbean Region or West Indies or South America or Latin America or Central America or Afghanistan or Albania or Algeria or Angola or Argentina or Armenia or Armenian or Azerbaijan or Bangladesh or Benin or Byelarus or Byelorussian or Belarus or Belorussian or Belorussia or Belize or Bhutan or Bolivia or Bosnia or Herzegovina or Hercegovina or Botswana or Brazil or Bulgaria or Burkina Faso or Burkina Fasso or Upper Volta or Burundi or Urundi or Cambodia or Khmer Republic or Kampuchea or Cameroon or CameroonsOR Cameron or Camerons or Cape Verde or Central African Republic or Chad or China or Colombia or Comoros or Comoro Islands or Comores or Mayotte or Congo or Zaire or Costa Rica or Cote d'Ivoire or Ivory Coast or Cuba or Djibouti or French Somaliland or Dominica or Dominican Republic or East Timor or East Timur or Timor Leste or Ecuador or Egypt or United Arab Republic or El Salvador or Eritrea or Ethiopia or Fiji or Gabon or Gabonese Republic or Gambia or Gaza or Georgia Republic or Georgian Republic or Ghana or Gold Coast or Grenada or Guatemala or Guinea or Guiana or Guyana or Haiti or Honduras or India or Maldives or Indonesia or Iran or Iraq or Isle of Man or Jamaica or Jordan or Kazakhstan or Kazakh or Kenya or Kiribati or Korea or Kosovo or Kyrgyzstan or Kirghizia or Kyrgyz Republic or Kirghiz or Kirgizstan or "Lao PDR" or Laos or Lebanon or Lesotho or Basutoland or Liberia or Libya or Macedonia or Madagascar or Malagasy Republic or Malaysia or Malaya or Malay or Sabah or Sarawak or Malawi or Nyasaland or Mali or Marshall Islands or Mauritania or Mauritius or Agalega Islands or Mexico or Micronesia or Middle East or Moldova or Moldovia or Moldovian or Mongolia or Montenegro or Morocco or Ifni or Mozambique or Myanmar or Myanma or Burma or Namibia or Nepal or Nicaragua or Niger or Nigeria or Muscat or Pakistan or Palau or Palestine or Panama or Paraguay or Peru or Philippines or Philipines or Phillipines or Phillippines or Romania or Rumania or Roumania or Russia or Russian or Rwanda or Ruanda or Saint Lucia or St Lucia or Saint Vincent or St Vincent or Grenadines or Samoa or Samoan Islands or Navigator Island or Navigator Islands or Sao Tome or Senegal or Serbia or Montenegro or Seychelles or Sierra Leone or Sri Lanka or Ceylon or Solomon Islands or Somalia or Sudan or Suriname or Surinam or Swaziland or Syria or Tajikistan or Tadzhikistan or Tadjikistan or Tadzhik or Tanzania or Thailand or Togo or Togolese Republic or Tonga or Tunisia or Turkey or Turkmenistan or Turkmen or Uganda or Ukraine or USSR or Soviet Union or Union of Soviet Socialist Republics or Uzbekistan or Uzbek or Vanuatu or New Hebrides or Venezuela or Vietnam or Viet Nam or West Bank or Yemen or Yugoslavia or Zambia or Zimbabwe or Rhodesia).tw,kf

9. 4 OR 7

10. 8 AND 9

3 Final Search Strategy: MEDLINE

### Population

1. Pregnant women/or Mothers/or Maternal Age/or Pregnancy in Adolescence/or Pregnancy, Multiple/or exp Perinatology/or First Trimester, Pregnancy/or Second Trimester, Pregnancy/or Third Trimester, Pregnancy/or Obstetrics/or exp Maternal Behavior/or Surrogate Mothers/or Pregnancy Outcome/or Prenatal Care/or Perinatal Care/or Postnatal Care/or Postpartum Period/or Lactation/or Pregnancy Complications/or (pregnan* or pregnan* wom#n* or mother* or mom* or surroga* or matern* or preconcept* or pre‐concept* or "pre concept*" or periconcept* or peri‐concept* or "peri concept* " or "multi*pregnan*" or "child birth " or child‐birth or childbirth or "term birth or trimester" or (pregnan* and (expecting or reproductive age or women of reproductive age or WRA)) or partus or lactation or fetal or foetal or fetus or foetus or obstetric* or labo#r or childbear* or "child‐bear*" or "gestation*" or (("reproductive age" or "wom#n of reproductive age") and pregnan*) or antenatal or ante‐natal or "ante natal " or pre‐natal or "pre natal " or prenatal or perinatal or peri‐natal or "peri natal" or prepartum or pre‐partum or "pre partum" or perinatology or peripartum or peri‐partum or "peri partum " or puerper* or postpartum or post‐partum or "post partum " or postnatal or post‐natal or "post natal " or surrogate or "pregnant wom#n" or paturiency or child‐carrying or "child carrying").mp. or ((pregnan* wom#n* or matern* or pregnan*) and ("postnatal care" or "post‐partum")).tw,kf

### Intervention 1: Single and Multiple Micronutrient

2. Micronutrients/or vitamins/or minerals/or exp iron/or exp iron compounds/or iron, dietary/or vitamin A/or exp iodine/or exp zinc or exp zinc compounds/or exp vitamin D/or (micronutrient* or multinutrient* or multi‐nutrient* or "multi*nutrient" or "multimicro‐nutrient*" or "multimicronutrient*" or multivitamin* or "multi‐vitamin*" or multimineral* or "multi‐mineral*" or "multiple micro nutrient*" or "multiple micronutrient" or micro‐nutrient* or MMN or "essential vitamins*" or minerals* or "m.v.i. pediatric" or "trace element*" or "trace mineral*" or "trace metal" or vitamin* or davitamin or "vitamin d" or "hydroxyvitamin d" or vitamin‐d or "25 hydroxyvitamin d" or "25 hydroxy‐vitamin d" or "25‐hydroxyvitamin d" or "25‐hydroxy‐vitamin d" or "25‐hydroxyvitamin d" or 25ohd or "25‐oh‐vitamin d" or 25‐ohd or "vitamin d2" or vitamin‐d2 or "25‐hydroxyvitamin d2" or "25‐hydroxy‐vitamin d2" or "vitamin d3" or vitamin‐d3 or "25 hydroxyvitamin d3" or "25 hydroxyvitamin d3" or "25‐hydroxy‐vitamin d3" or calcidiol or calcifediol or calcium or retinol* or retinal* or Retinaldehyde or retinoid or Retinoids or retinoic or beta‐carotene or "beta carotene" or iron or "Fe(III)" or "Fe3+" or "iron(III) " or "Ferrous ion" or "Fe(II)" or "iron(II)" or "Fe2+" or "ferr* compounds" or zinc or "zn" or "zn acetate" or "zn sulfate" or "zn oxide" or iodine or "iod* compounds" or "ferr* compounds" or "folic acid" or "ergocalciferol derivative" or "ergocalciferol‐D2" or cholecalciferol‐D3 or "colecalciferol derivative" or iodiz* or "beta carotene" or "b‐tene" or "beta carotin" or betacarotene).tw,kf

3. Exp Dietary supplements/or tablets/or syrup/or capsules/or (supplement* or nutraceutical* or nutriceutical* or neutraceutical* or capsule* or tablet* or syrup* or drop*).tw,kf

4. 1 AND 2 AND 3

### Intervention 2: Lipid‐nutrient supplementation

5. exp Lipids/or (lipid* or oil* or soy* or peanut* or whey* or sesame* or cashew* or chickpea* or protein* or butter* or fat or fats or fatty or "dairy product*" or "omega‐3" or "omega 3" or "alpha‐linolenic acid" or "docosahexaenoic acids" or "eicosapentaeonic acid" or "n‐3 pufa" or "n3 pufa" or glyceride*).tw,kf

6. Dietary supplements/or ("lipid based" or "lipid‐based nutri*" or enrich* or emuls* or "Lipid Emulsions" or "Fat Emulsions" or "Intravenous Fat Emulsions" or powder* or spread* or paste* or LNS or iLiNS or supplement* or neutraceutical* or nutraceutical* or nutriceutical* or Nutributter* or Plumpy* or PlumpyNut or "ready to use" or "ready‐to‐use therapeutic food" or "ready‐to‐use supplementary food" or RUFF or RUSF or RUTF).tw,kf

7. 1 AND 5 AND 6

### Intervention 3: Point of use fortification with micronutrient powders

8. Exp Vitamins/or exp minerals/or exp micronutrients/

9. Powders/or (Sprinkle* or powder* or foodlet* or "foodlet‐based" or "crushable nutritabs" or "micronutrient powder" or "multiple micronutrient powder" or mnp or "drug dosage form" or "micronutrient powder*" or "MNP").tw,kf

10. ("point‐of‐use" or enrich* or fortif* or "diet therapy" or supplement* or "diet treatment" or "diet additive" or "dietary therapy" or "dietary treatment" or nutraceutical* or nutriceutical* or neutraceutical*).tw,kf

11. 1 AND 8 AND 9 AND 10

12. Developing Countries/or developing country*.tw, kf or ("developing country" or "developing countries" or "developing nation" or "developing nations" or "developing population" or "developing populations" or "developing world" or "less developed country" or "less developed countries" or "less developed nation" or "less developed nations" or "less developed population" or "less developed populations" or "less developed world" or "lesser developed country" or "lesser developed countries" or "lesser developed nation" or "lesser developed nations" or "lesser developed population" or "lesser developed populations" or "lesser developed world" or "under developed country" or "under developed countries" or "under developed nation" or "under developed nations" or "under developed population" or "under developed populations" or "under developed world" or "underdeveloped country" or "underdeveloped countries" or "underdeveloped nation" or "underdeveloped nations" or "underdeveloped population" or "underdeveloped populations" or "underdeveloped world" or "middle income country" or "middle income countries" or "middle income nation" or "middle income nations" or "middle income population" or "middle income populations" or "low income country" or "low income countries" or "low income nation" or "low income nations" or "low income population" or "low income populations" or "lower income country" or "lower income countries" or "lower income nation" or "lower income nations" or "lower income population" or "lower income populations" or "underserved country" or "underserved countries" or "underserved nation" or "underserved nations" or "underserved population" or "underserved populations" or "underserved world" or "under served country" or "under served countries" or "under served nation" or "under served nations" or "under served population" or "under served populations" or "under served world" or "deprived country" or "deprived countries" or "deprived nation" or "deprived nations" or "deprived population" or "deprived populations" or "deprived world" or "poor country" or "poor countries" or "poor nation" or "poor nations" or "poor population" or "poor populations" or "poor world" or "poorer country" or "poorer countries" or "poorer nation" or "poorer nations" or "poorer population" or "poorer populations" or "poorer world" or "developing economy" or "developing economies" or "less developed economy" or "less developed economies" or "lesser developed economy" or "lesser developed economies" or "under developed economy" or "under developed economies" or "underdeveloped economy" or "underdeveloped economies" or "middle income economy" or "middle income economies" or "low income economy" or "low income economies" or "lower income economy" or "lower income economies" or "low gdp" or "low gnp" or "low gross domestic" or "low gross national" or "lower gdp" or "lower gnp" or "lower gross domestic" or "lower gross national" or lmic or lmics or "third world" or "lami country" or "lami countries" or "transitional country" or "transitional countries" or Africa or Asia or Caribbean Region or West Indies or South America or Latin America or Central America or Afghanistan or Albania or Algeria or Angola or Argentina or Armenia or Armenian or Azerbaijan or Bangladesh or Benin or Byelarus or Byelorussian or Belarus or Belorussian or Belorussia or Belize or Bhutan or Bolivia or Bosnia or Herzegovina or Hercegovina or Botswana or Brazil or Bulgaria or Burkina Faso or Burkina Fasso or Upper Volta or Burundi or Urundi or Cambodia or Khmer Republic or Kampuchea or Cameroon or CameroonsOR Cameron or Camerons or Cape Verde or Central African Republic or Chad or China or Colombia or Comoros or Comoro Islands or Comores or Mayotte or Congo or Zaire or Costa Rica or Cote d'Ivoire or Ivory Coast or Cuba or Djibouti or French Somaliland or Dominica or Dominican Republic or East Timor or East Timur or Timor Leste or Ecuador or Egypt or United Arab Republic or El Salvador or Eritrea or Ethiopia or Fiji or Gabon or Gabonese Republic or Gambia or Gaza or Georgia Republic or Georgian Republic or Ghana or Gold Coast or Grenada or Guatemala or Guinea or Guiana or Guyana or Haiti or Honduras or India or Maldives or Indonesia or Iran or Iraq or Isle of Man or Jamaica or Jordan or Kazakhstan or Kazakh or Kenya or Kiribati or Korea or Kosovo or Kyrgyzstan or Kirghizia or Kyrgyz Republic or Kirghiz or Kirgizstan or "Lao PDR" or Laos or Lebanon or Lesotho or Basutoland or Liberia or Libya or Macedonia or Madagascar or Malagasy Republic or Malaysia or Malaya or Malay or Sabah or Sarawak or Malawi or Nyasaland or Mali or Marshall Islands or Mauritania or Mauritius or Agalega Islands or Mexico or Micronesia or Middle East or Moldova or Moldovia or Moldovian or Mongolia or Montenegro or Morocco or Ifni or Mozambique or Myanmar or Myanma or Burma or Namibia or Nepal or Nicaragua or Niger or Nigeria or Muscat or Pakistan or Palau or Palestine or Panama or Paraguay or Peru or Philippines or Philipines or Phillipines or Phillippines or Romania or Rumania or Roumania or Russia or Russian or Rwanda or Ruanda or Saint Lucia or St Lucia or Saint Vincent or St Vincent or Grenadines or Samoa or Samoan Islands or Navigator Island or Navigator Islands or Sao Tome or Senegal or Serbia or Montenegro or Seychelles or Sierra Leone or Sri Lanka or Ceylon or Solomon Islands or Somalia or Sudan or Suriname or Surinam or Swaziland or Syria or Tajikistan or Tadzhikistan or Tadjikistan or Tadzhik or Tanzania or Thailand or Togo or Togolese Republic or Tonga or Tunisia or Turkey or Turkmenistan or Turkmen or Uganda or Ukraine or USSR or Soviet Union or Union of Soviet Socialist Republics or Uzbekistan or Uzbek or Vanuatu or New Hebrides or Venezuela or Vietnam or Viet Nam or West Bank or Yemen or Yugoslavia or Zambia or Zimbabwe or Rhodesia).tw,kf

13. 4 AND 12

14. 7 AND 12

15. 11 AND 12

16. 13 OR 14 OR 15

17. Limit 16 to yr="1995‐current"
